# Photocatalysis and Kinetic Resolution by Lithiation to Give Enantioenriched 2-Arylpiperazines

**DOI:** 10.1021/acs.orglett.3c00074

**Published:** 2023-02-03

**Authors:** Anjan Das, Anthony Choi, Iain Coldham

**Affiliations:** Department of Chemistry, University of Sheffield, Brook Hill, Sheffield S3 7HF, U.K.

## Abstract

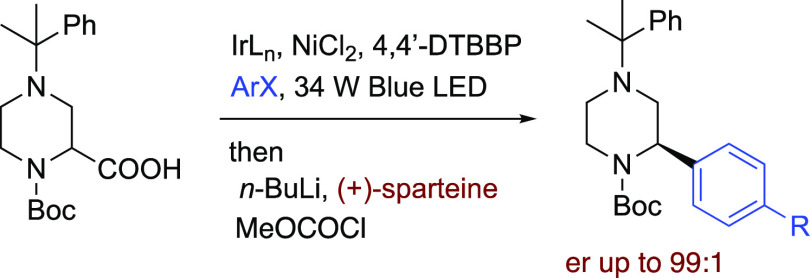

Piperazines are important heterocycles in drug compounds. We report the asymmetric synthesis of arylpiperazines by photocatalytic decarboxylative arylation (metallaphotoredox catalysis) then kinetic resolution using *n*-BuLi/(+)-sparteine. This gave a range of piperazines with very high enantioselectivities. Further functionalizations gave enantioenriched 2,2-disubstituted piperazines, and either N-substituent can be removed selectively. Late-stage functionalizations of enantioenriched piperazine derivatives were demonstrated, including synthesis of a drug compound with glycogen synthase kinase (GSK)-3β inhibitor activity with potential for treating Alzheimer’s disease.

Piperazines are one of the most important saturated nitrogen heterocycles found in small-molecule pharmaceuticals.^1^ Their druglike properties and synthetic versatility make them excellent candidates in drug discovery programs. For example, imatinib (marketed as Gleevec), a BCR-Abl tyrosine kinase inhibitor, is used in the treatment of multiple cancers with high response rate,^2^ and gatifloxacin is an important fluoroquinolone antibiotic (Figure 1).^3^ The majority of the piperazines in pharmaceuticals contain substituents only at the two nitrogen atoms. However, having substitution at a carbon atom of the piperazine ring is very important in drug discovery.^4^ Examples of substituted piperazines occur in vestipitant, a neurokinin-1 antagonist which is currently in clinical trials for the treatment of anxiety and tinnitus,^5^ and indinavir, a protease inhibitor used to treat HIV/AIDS.^6^ Development of methods to access *C*-substituted piperazines, particularly with control of absolute configuration, would promote structural diversity in small-molecule collections to aid medicinal chemistry.

**Figure 1:**
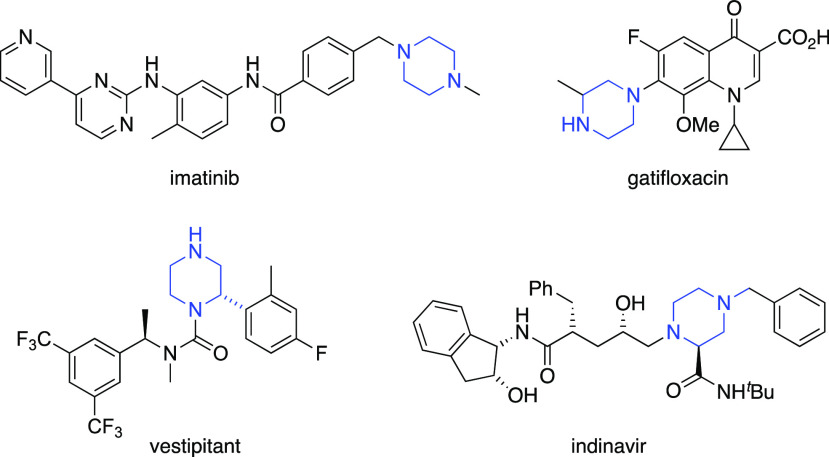
Bioactive piperazines.

The most common methods for the formation of 2-substituted piperazines use amino acid starting materials and cyclization chemistry.^7^ The direct functionalization of the intact piperazine ring is an attractive alternative strategy, allowing late-stage introduction of the substituent.^8^ One such approach uses lithiation chemistry.^9−11^ This lithiation–trapping chemistry has provided access to a small selection of 2-substituted piperazines, but extension via transmetalation and Negishi coupling to give 2-arylpiperazines was very low-yielding.^11^ A recent report of photoredox catalysis has allowed direct arylation or vinylation but there are very few examples, and this is limited to the formation of racemic *N*-aryl piperazines.^12^ The direct C–H functionalization of six-membered saturated heterocycles by photocatalysis is problematic due to the poor yields of the product,^13,14^ so we were attracted to a decarboxylative arylation strategy.^15,16^ Here we report the successful synthesis of 2-arylpiperazines using photoredox chemistry followed by kinetic resolution using asymmetric lithiation^17^ to provide highly enantioenriched substituted piperazines. This chemistry allows a new route to 2-arylpiperazines with control of absolute configuration and is showcased with an application to the preparation of a GSK-3β inhibitor with excellent yield and enantioselectivity.

Initial work investigated the possibility of carrying out a direct C–H activation using Ir[dF(CF_3_)ppy]_2_(dtbbpy)PF_6_ as the photocatalyst.^13^ However, this resulted in low yields of the 2-arylpiperazine products (up to 35% yield using methyl 4-bromobenzoate as the aryl halide) (see the SI). Therefore, the piperazine **1** was formed (see the SI) from the parent piperazine^11^ using *sec*-BuLi, TMEDA, and ground dry ice.^18^ We then explored decarboxylative arylation for the synthesis of a series of 2-arylpiperazines using photocatalysis (Scheme 1).

**Scheme 1:**
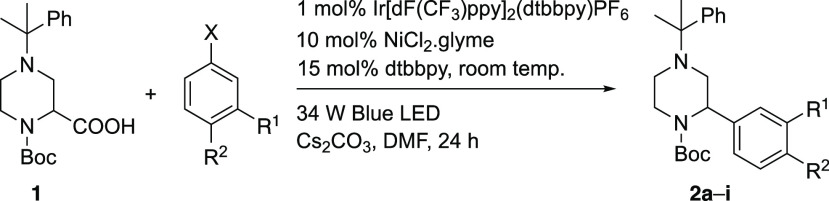
Photocatalysis to Give Piperazines **2**

As anticipated from the literature,^15,16^ the presence of an electron-withdrawing group, such as an acyl or cyano group, gave the best results (Table 1, entries 1, 2, and 5). In the absence of such a group, relatively low yields of the desired products were obtained when starting with the aryl bromide precursor (entries 3, 4, and 6). However, switching to the aryl iodide gave improved results (entries 8 and 9) and even allowed formation of the 2-phenyl derivative **2g** (entry 7) and more electron-rich derivatives (entries 11 and 12). Compared with entry 9, a similar yield (48%) of the product **2f** was obtained using (instead of the iridium catalyst) the organophotocatalyst 4CzIPn^16b^ and the aryl iodide (entry 10). The reactions were generally performed using 0.6 mmol of acid **1** but could be scaled to 5.7 mmol (2 g) without a significant reduction in yield (**2f**, 50% yield after 72 h).

**Table 1:** Yields of Isolated Piperazines **2**

Entry	X	R^1^	R^2^	Product	Yield (%)
1	Br	H	COMe	**2a**	80
2	Br	H	CO_2_^*t*^Bu	**2b**	55
3	Br	H	F	**2c**	30
4	Br	H	CF_3_	**2d**	34
5	Br	H	CN	**2e**	70
6	Br	H	Cl	**2f**	25
7	I	H	H	**2g**	30
8	I	H	F	**2c**	55
9	I	H	Cl	**2f**	58
10	I	H	Cl	**2f**	48^a^
11	I	OMe	H	**2h**	42
12	I	H	Me	**2i**	35

a Using 4CzIPn as photocatalyst.

With the 2-arylpiperazines **2** in hand, we were interested in exploring a kinetic resolution protocol developed in our laboratories.^17^ This chemistry relies on coordination of the chiral base to the carbonyl oxygen atom of the Boc group, so it is important to determine the ratio of rotamers and the rate of rotation of the carbonyl group. From variable-temperature (VT) NMR spectroscopy with the piperazine **2c** in *d*_8_-THF (Figure 2), the ratio of rotamers is approximately 1:1 and the activation parameters for the rotation were determined as Δ*H*^⧧^ ≈ 44 kJ/mol and Δ*S*^⧧^ ≈ – 27 J/K·mol. These parameters equate to a barrier to rotation, Δ*G*^^⧧^^ ≈ 49 kJ/mol at −78 °C, and this gives a half-life for rotation of about 2 s at this temperature. These results were supported by density functional theory (DFT) calculations using the B3LYP functional including dispersion interactions and the def2TZVP basis set.^19^ These calculations indicated a 53:47 ratio of rotamers of **2g** and a rotation barrier Δ*G*^⧧^ ≈ 50 kJ/mol at −78 °C (see the SI). Hence, despite the presence of both rotamers, the lithiation should be possible due to the relatively fast rate of rotation of the Boc group.

**Figure 2:**
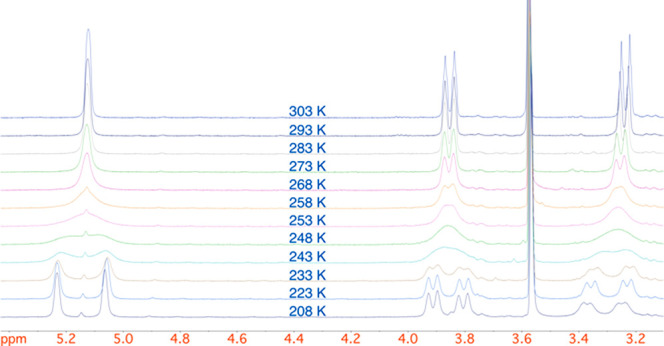
^1^H VT-NMR spectroscopy of piperazine **2c** (400 MHz, *d*_8_-THF) showing 5.40–3.10 ppm.

Kinetic resolution of the piperazine derivatives was investigated using (+)-sparteine as the chiral ligand in toluene and adding *n*-BuLi to this mixture. After optimization, we found very good results for the kinetic resolution using 0.6 equiv of *n*-BuLi along with 0.8 equiv (+)-sparteine for 30 min (Scheme 2). Trapping the resulting mixture with methyl chloroformate gave the disubstituted products **3b**,**c**,**f**,**g**,**i**, together with recovered piperazines **2b**,**c**,**f**,**g**,**i** with enantiomer ratios (er) up to 99:1. The results indicate a selectivity factor *S* ≈ 17.^20^ The resolution tolerated a bulky ester substituent at C-4 of the aromatic ring (**2b**) but not the nitrile **2e**. The 4-fluoro-, 4-chloro-, and 4-methyl-substituted piperazines **2c**, **2f**, and **2i** were excellent substrates. The absolute configuration of the major enantiomer was assigned on the basis of the known preference for BuLi/(+)-sparteine to remove the pro-(*R*) proton on the carbon atom attached to the *N*-Boc group,^21,22^ and this aligns with all previous related examples.^17^ A model to illustrate the preference is shown (Figure 3), in which the (+)-enantiomer of sparteine when coordinated to BuLi favors lithiation of the (*S*) enantiomer of the piperazines **2**. To favor the other enantiomer, (−)-sparteine could be used.^17^

**Scheme 2:**
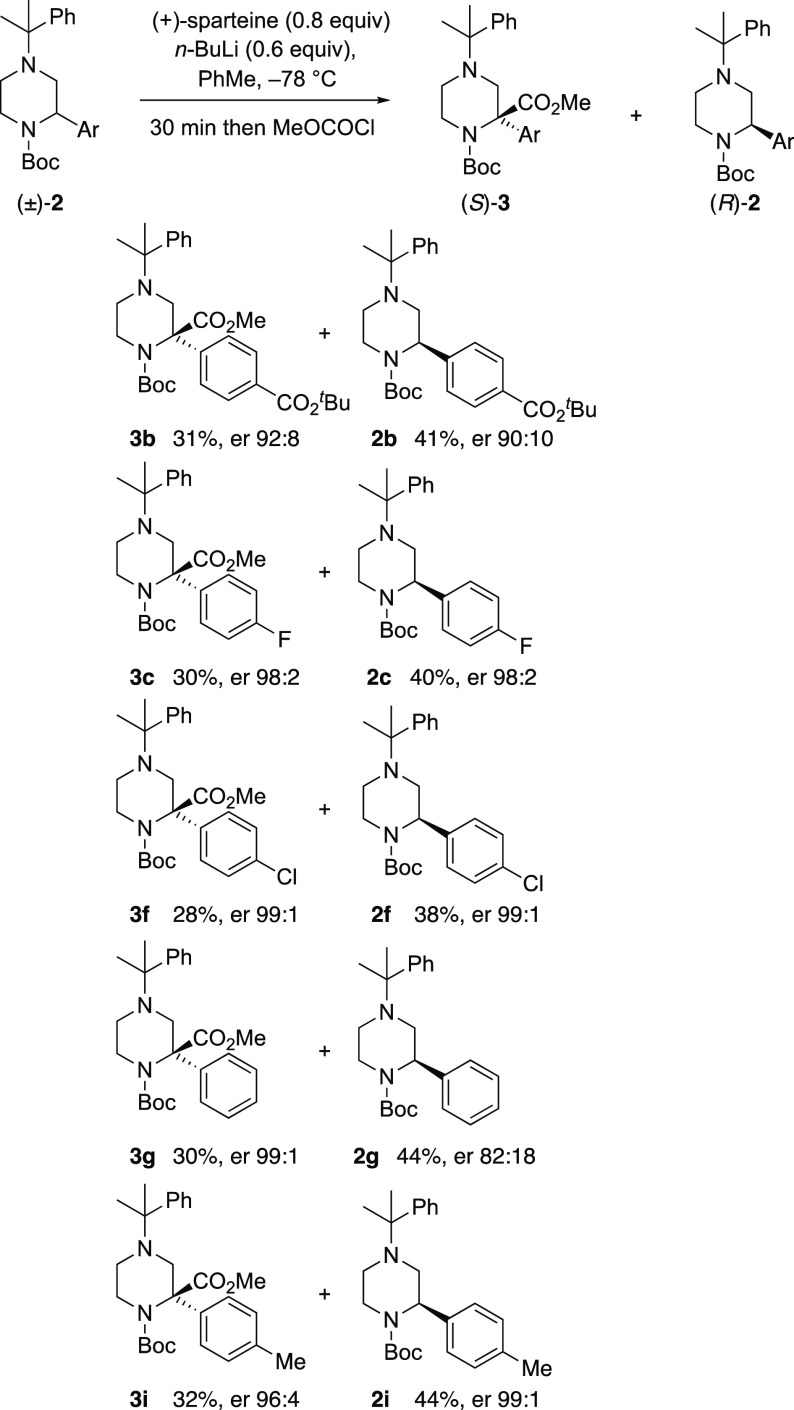
Kinetic Resolution of Selected Piperazines **2**

**Figure 3:**
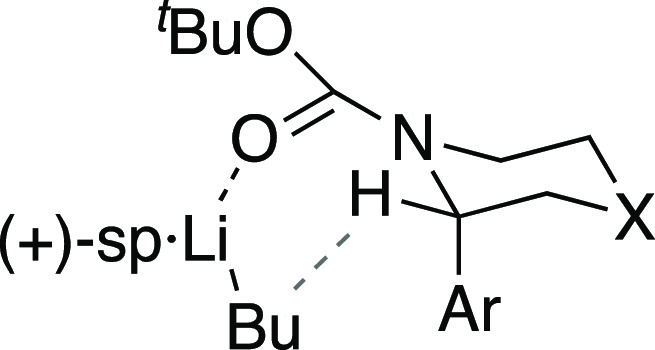
Working model to show preferential abstraction of the proton at C-2 using (+)-sparteine (sp) (X = *N*-cumyl, this work).

The kinetic resolution reactions resulted in recovered 2-arylpiperazines (38–44% yields) and 2,2-disubstituted piperazines (28–32% yields) with excellent enantioselectivities. The remaining material was, at least in part, the product of ring-opening from β-elimination of the intermediate organolithium species. This side reaction has been noted before with lithiated piperazines, particularly after addition of the electrophile that could coordinate to the distal nitrogen atom.^10,11^ The bulky *N*-cumyl group should minimize such an interaction although in our substrates there is a higher propensity for elimination due to formation of a conjugated alkene (styrene). Despite this, we were able to demonstrate successful lithiation–trapping of the enantioenriched recovered piperazines (Scheme 3). For example, treatment of piperazine (*R*)-**2c** (er 98:2) with *n*-BuLi in THF at low temperature for 10 min followed by addition of iodomethane gave the piperazine **4** with high yield and only a slight drop in enantiopurity. The cumyl protecting group on the distal nitrogen atom could be removed easily by hydrogenolysis to give the piperazine **5**. To demonstrate the orthogonality of the protecting groups, the *N*-cumyl and *N*-Boc groups were cleaved selectively from the piperazine (*R*)-**2f** to give the piperazines **6** and **7**, respectively, without loss of enantiopurity (Scheme 4).

**Scheme 3:**
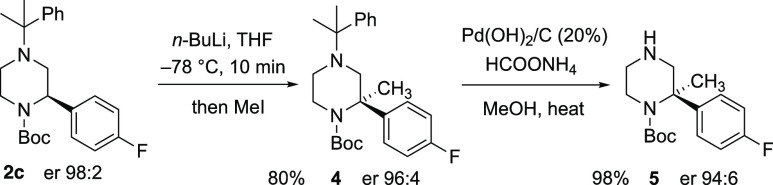
Lithiation–Trapping of Piperazine **2c**

**Scheme 4:**
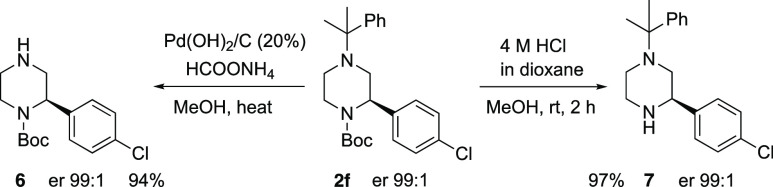
Deprotection of Piperazine **2f**

To illustrate the application of this chemistry in synthesis, we prepared the glycogen synthase kinase (GSK)-3β inhibitor **9** (Scheme 5).^23^ The chloride **8** was prepared according to the literature^24^ and was coupled with the piperazine **6**. This was followed directly by acid-promoted deprotection of the Boc group to give the desired bioactive compound **9**. This drug has potential for the treatment of Alzheimer’s disease, and either enantiomer will be accessible depending on the choice of enantiomer of the chiral ligand sparteine in the kinetic resolution chemistry.

**Scheme 5:**
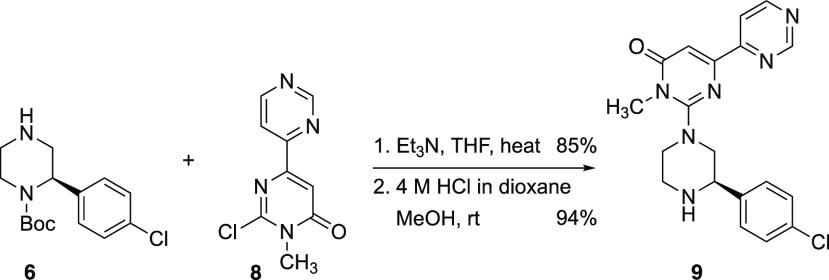
Preparation of the GSK-3β Inhibitor **8**

In conclusion, we have developed a short synthesis of 2-arylpiperazines by photocatalysis. These products are amenable to kinetic resolution with sparteine as the chiral ligand to provide the recovered 2-arylpiperazine and 2,2-disubstituted piperazines with high enantioselectivities. The lithiated intermediates are configurationally stable at low temperature, and this allows the incorporation of electrophiles at C-2 to give other substituted products. The regioselective and stereoselective lithiation–trapping of the 2-arylpiperazines provides a useful way to prepare uncommon, but potentially valuable, 2,2-disubstituted piperazines. The choice of a cumyl group on one nitrogen atom and a Boc group on the other nitrogen atom of the piperazine ring means that the protecting groups are orthogonal and either *N*-substituent can be cleaved selectively. The chemistry can be applied to the synthesis of enantiomerically enriched bioactive piperazine drug compounds.

## Data Availability

The data underlying this study are available in the published article and its Supporting Information.

## Supporting Information Available

Experimental procedures and characterization data, NMR spectra, HPLC traces, and DFT calculations (PDF)
