# Supplementary material for: Photocatalysis and Kinetic Resolution by Lithiation to Give Enantioenriched 2-Arylpiperazines
Source: Org Lett. 2023 Feb 3;25(6):987–91. doi: 10.1021/acs.orglett.3c00074 (PMC9942196; doi:10.1021/acs.orglett.3c00074)
Supplement: Supplementary file 1 — ol3c00074_si_001.pdf [file ol3c00074_si_001.pdf]

Photocatalysis and Kinetic Resolution by Lithiation to give Enantioenriched 2-Arylpiperazines

Anjan Das, Anthony Choi, Iain Coldham*

Department of Chemistry, University of Sheffield, Brook Hill, Sheffield, S3 7HF, UK

Contents

1. General procedures S-2
2. Experimental procedures and data S-3
   2.1 Preparation of starting material S-3
   2.2 General procedure for the arylation S-5
   2.3 Preparation of substrates by photocatalysis S-6
   2.4 Racemic lithiation–trapping S-12
   2.5 Kinetic resolution S-16
   2.6 Further functionalization S-19
   2.7 Synthesis of GSK-3β inhibitor S-23
3. VT-NMR spectra S-24
4. NMR spectra S-26
5. HPLC traces S-54
6. DFT calculations S-67
7. References S-120

1. General procedures

All the reagents were obtained from commercial suppliers and were used without further purification or after distillation; n-BuLi was titrated before use. Dry solvents were obtained from a Grubbs dry solvent system or obtained from commercial suppliers. Thin layer chromatography was performed on Merck silica gel 60F254 plates and visualised by UV irradiation at 254 nm or by staining with an alkaline $KMnO_4$ dip. Column chromatography was performed using DAVISIL or Geduran silica gel (40-63 micron mesh). Heating was performed using aluminum heating blocks on a hotplate stirrer. InfraRed spectra were recorded on a Perkin Elmer Spectrum RX Fourier Transform – IR System and only selected peaks are reported. ^{1}H NMR spectra were recorded on a Bruker AC400 (400 MHz) instrument. Chemical shifts are reported in ppm with respect to the residual solvent peaks, with multiplicities given as s = singlet, d = doublet, t = triplet, q = quartet, m = multiplet, br = broad. Coupling constants (J values) are quoted to the nearest 0.5 Hz with values in Hertz (Hz). ^{13}C NMR spectra were recorded on the above instrument at 100 MHz. Low and high resolution (accurate mass) mass spectra were recorded on an Agilent Technologies LC-MS quadrupole time-of-fight (Q-TOF) instrument for ElectroSpray (ES).

Light source: Kessil A160WE Tuna Blue, 34 W, blue LED, 460 nm wavelength. The light was placed 6 cm away from the reaction vessel (standard glass, round bottom flask) and no filter was used.

2. Experimental procedures and data

2.1 Preparation of starting material

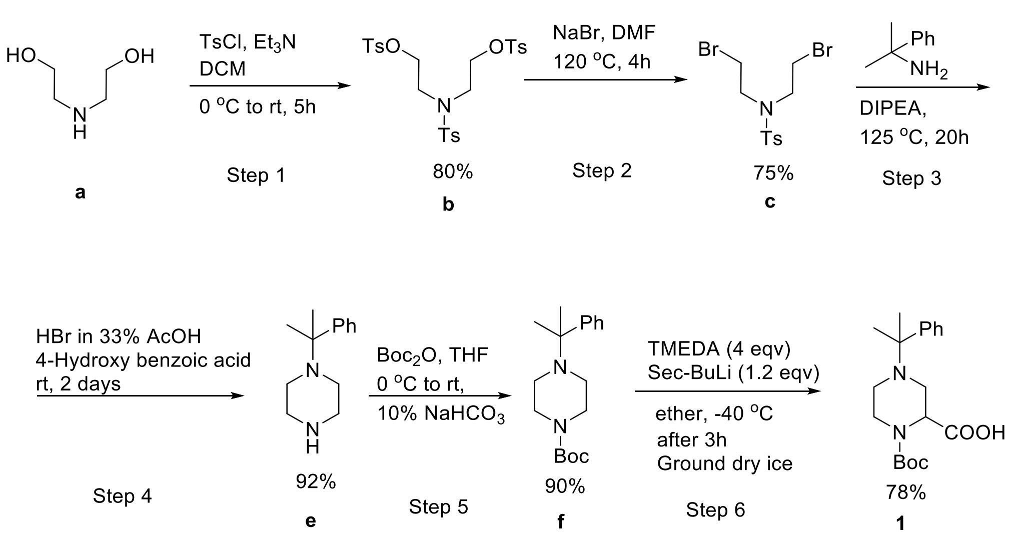

Preparation of ***b***:

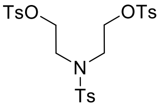

Triethylamine (45 mL, 0.44 mol) was added to diethanolamine **a** (13.75 g, 0.13 mol) in CH_2Cl_2 (250 mL). The mixture was cooled to 0 °C and p-toluenesulfonyl chloride (78.5 g, 0.41 mol) was added portion wise with vigorous stirring over 2 h. The mixture was allowed to warm to room temperature over 16 h. The mixture was filtered and the filtrate was washed with aqueous HCl (1 M) (3 x 100 mL), water (5 × 200 mL), and saturated $NaHCO_3$ solution (5 × 200 mL). The organic layer was dried ($MgSO_4$) and the solvent was evaporated to give the product **b** (60 g, 80%) as an amorphous white solid; m.p. 98 °C, lit.[1] m.p. 97.5 °C; ^1H NMR (400 MHz, $CDCl_3$) δ 7.81–7.77 (m, 4H), 7.64–7.62 (m, 2H), 7.44–7.30 (m, 6H), 4.13 (t, 4H, *J* = 8 Hz), 3.39 (t, 4H, *J* = 8 Hz), 2.48 (s, 6H), 2.45 (s, 3H). Data as reported.[1]

Preparation of ***c***:

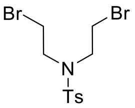

Tosylate **b** (43 g, 0.076 mol) and NaBr (40 g, 0.39 mol) in DMF (250 mL) were heated at 120 °C. After 4 h, the mixture was cooled to room temperature and concentrated to 50 mL under reduced pressure. The mixture was poured into a rapidly stirred ice–water mixture (1 L). The product was filtered and was washed with water (200 mL), hexane (200 mL), and then was air-dried to give product **c** (23.5 g, 75%) as an amorphous white solid; m.p. 56–58 °C, lit.[1] m.p. 58.2 °C; ^1H NMR (400 MHz, $CDCl_3$) δ 7.66 (d, 2H, *J* = 8 Hz), 7.37 (d, 2H, *J* = 8 Hz), 3.76 (t, 4H, *J* = 4 Hz), 3.00 (t, 4H, *J* = 4 Hz), 2.47 (s, 3H). Data as reported.[1]

*Preparation of **d***:

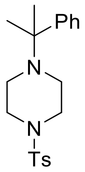

Dibromide **c** (9.2 g, 0.023 mol), cumylamine (3 g, 0.022 mol) and N,N-diisopropylethylamine (9 mL, 0.05 mol) were heated at 125 °C under nitrogen for 20 h. The mixture was allowed to cool to room temperature and EtOAc (100 mL) was added. The mixture was washed with water (2 x 200 mL). The organic layer was dried ($MgSO_4$), evaporated and purified by column chromatography, eluting with hexane:EtOAc 9:1) to give the product **d** (7 g, 82%) as a yellow oil; ^{1}H NMR (400 MHz, $CDCl_3$) δ 7.66 (d, 2H, *J* = 8 Hz), 7.43 (d, 2H, *J* = 8 Hz), 7.36 (d, 2H, *J* = 8 Hz), 7.30–7.26 (m, 2H), 7.22–7.18 (m, 1H), 2.98 (br, 4H), 2.59–2.56 (m, 4H), 2.48 (s, 3H), 1.33 (s, 6H). Data as reported.[2]

*Preparation of **e***:

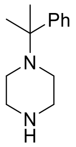

Hydrogen bromide solution (155 mL, 33 wt % in acetic acid) was added to N-Ts-piperazine **d** (13.5 g, 0.037 mol) and 4-hydroxybenzoic acid (13.5 g, 0.097 mol) and the mixture was stirred under nitrogen at room temperature. After 2 d, water (150 mL) was added slowly and the mixture was stirred for 2 h. The precipitate was filtered and was washed with water (2 x 100 mL). The solution was cooled to 0 °C and KOH pellets were added portion wise until pH > 10, and the mixture was extracted with EtOAc (3 x 150 mL). The combined organic layers were washed with brine (200 mL), dried ($MgSO_4$), and evaporated to give the product **e** (6.9 g, 90%) as a colourless oil; ^{1}H NMR (400 MHz, $CDCl_3$) δ 7.56 (d, 2H, *J* = 4Hz), 7.34–7.30 (m, 2H), 7.24–7.20 (m, 1H), 2.87 (t, 4H, *J* = 4 Hz), 2.47 (t, 4H, *J* = 4 Hz), 1.91 (s, 1H), 1.36 (s, 6H). Data as reported.[2]

*Preparation of **f***:

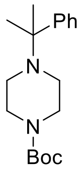

Compound **e** (9.1 g, 30 mmol) in THF (50 mL) was dropwise added to di-tert-butyl dicarbonate (9.5 g, 43.5 mmol) in THF (200 mL) at 0 °C. After 10 min the mixture was warmed to room temperature. After 2 h, 10% $NaHCO_3$(aq) solution (150 mL) was added and the mixture was extracted with EtOAc (2 × 250 mL). The combined organic extracts were washed with brine (200 mL), dried ($MgSO_4$), and evaporated. Purification by column chromatography on silica gel, eluting with EtOAc–hexane (1:9), gave the product **f** (12.2 g, 90%) as a yellow oil; ^{1}H NMR (400 MHz, $CDCl_3$) δ 7.55 (d, 2H, *J* = 8Hz), 7.34–7.31 (m, 2H), 7.25–7.21 (m, 1H), 3.40 (t, 4H, *J* = 4Hz),

2.44 (t, 4H, J = 4Hz), 1.47 (s, 9H), 1.36 (s, 6H); ^{13}C NMR (100 MHz, $CDCl_3$) δ 154.8, 148.8, 128.0, 126.2, 126.0, 79.3, 59.7, 46.0 (2 x CH_2), 28.4, 24.1. Data as reported.[3]

*Preparation of **1***:

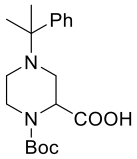

sec-BuLi (16.5 mL, 19.8 mmol, 1.2 M in cyclohexane) was added to piperazine **f** (5 g, 16.5 mmol) and TMEDA (10.0 mL, 65.8 mmol) in Et_2O (75 mL) at –40 °C under nitrogen. After 3 h, dry ice (100 g), which had been ground under a continuous flow of nitrogen and rinsed with dry THF,[4] was added. The mixture was allowed to warm to room temperature and the solvent was evaporated. Water (200 mL) was added and aqueous HCl (2 M) was added at 0 °C to maintain pH 2. The mixture was extracted with EtOAc (3 x 150 mL) and the combined organic layers were dried ($MgSO_4$) and the solvent was evaporated to give the acid **1** (4.4 g, 78%) as an amorphous white solid; m.p. 98–99 °C; FT-IR ν_{max} (ATR)/cm^{-1} 2974, 2934, 2820, 1701, 1597, 1450, 1388, 1366, 1170, 1116, 966, 764, 770; ^{1}H NMR (400 MHz, $CDCl_3$) (~55:45 mixture of rotamers) δ 7.71 (br s, 1H), 7.50 (d, 2H, J = 8 Hz), 7.36–7.32 (m, 2H), 7.26–7.22 (m, 1H) 4.79 (br s, 0.45H), 4.63 (br s, 0.55H), 3.90 (br d, 0.55H, J = 12 Hz), 3.78 (br d, 0.45H, J = 12 Hz), 3.52 (br d, 1H, J = 12 Hz), 3.38 (td, 1H, J = 12 Hz, 4 Hz), 2.95 (br d, 0.55H, J =12 Hz) 2.80 (br d, 0.45H, J = 12 Hz), 2.50–2.45 (m, 1H), 2.32–2.23 (m, 1H), 1.51 (s, 3H), 1.48–1.46 (m, 12H) ; ^{13}C NMR (100 MHz, $CDCl_3$, rotamers) δ 175.3, 174.8, 155.7, 155.4, 128.4, 128.2, 127.4, 126.9, 126.6, 126.3, 80.4, 80.3, 61.9, 60.9, 55.4, 54.5, 47.7, 46.0, 42.1, 40.6, 28.4, 28.3, 25.0, 24.7, 22.8, 22.6; HRMS (ESI-TOF) m/z: $[M+H]^+$ Calcd for $C_{19}H_{29}N_2O_4$ 349.2127. Found 349.2148.

2.2 General procedure for the arylation

(A) Direct C–H activation:[5] In a 10 mL dry Schlenk tube with a magnetic stirring bar were added the aryl bromide (0.5 mmol), *N*-Boc-*N'*-cumylpiperazine **f** (1.0 mmol), and $Ir[dF(CF_3)ppy]_2(dtbbpy)PF_6$ (5.6 mg, 5.0 µmol, 1.0 mol%). Anhydrous DMSO (1.0 mL) and 3-acetoxyquinuclidine (85 µL, 0.55 mmol) were added. A solution (1 mL) taken from a 0.5 M stock solution in anhydrous DMSO of 4,7-dimethoxy-1,10-phenanthroline (1.2 mg, 5.0 µmol, 1.0 mol%) and nickel(II) bromide trihydrate (1.4 mg, 5.0 µmol, 1.0 mol%) was added. After 10 min, water (360 µL, 20 mmol) was added. The mixture was degassed via two cycles of freeze-pump-thaw using liquid nitrogen. The Schlenk tube was placed ~6 cm away from a 34 W blue LED and stirred, with cooling by a fan. After 20 h, aq. $NaHCO_3$ (30 mL) was added, and the aqueous layer was extracted with EtOAc (3 x 50 mL). The combined organic layers were dried ($MgSO_4$) and evaporated. Purification by column chromatography, as described below, gave the product.

***(B) Decarboxylative arylation*:**[6] In an oven-dried 40 mL Schlenk tube with a magnetic stir bar were added Ir[dF(CF_3)ppy]$_2$(dtbbpy)PF_6 (4.0 µmol, 0.01 equiv), $NiCl_2$·glyme (0.04 mmol, 0.1 equiv), 4,4'-di-tert-butyl-2,2'-bipyridyl (0.06 mmol, 0.15 equiv), the aryl halide (1. 0 mmol, 2.5 equiv), the carboxylic acid **1** (0.60 mmol, 1.5 equiv), Cs_2CO_3 (0.60 mmol, 1.5 equiv), and DMF (20 mL). The mixture was degassed by freeze-pump-thaw using liquid nitrogen. The Schlenk tube was placed ~6 cm away from a 34 W blue LED and stirred, with cooling by a fan. After 24 h, saturated aqueous $NaHCO_3$ solution (30 mL) was added, and the aqueous layer was extracted with EtOAc (3 × 100 mL). The combined organic layers were washed with water and brine, dried ($MgSO_4$) and evaporated. Purification by column chromatography, as described below, gave the product.

2.3 Preparation of substrates by photocatalysis

1-(tert-Butoxycarbonyl)-2-(4-methoxycarbonylphenyl)-4-(2-phenylpropan-2-yl)piperazine (**A**)

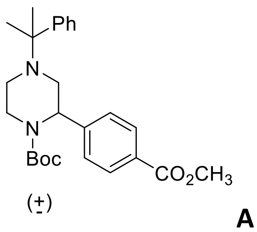

A

Using general procedure A, *N*-Boc-*N'*-cumylpiperazine (**f**) (see page S-4) (305 mg, 1 mmol) and methyl 4-bromobenzoate (108 mg, 0.5 mmol) gave, after purification by column chromatography on silica gel, eluting with hexane–EtOAc (9:1), the piperazine **A** (153 mg, 35%) as a colourless oil; R_f = 0.65 [hexane–EtOAc (4:1)]; FT-IR ν_{max} (ATR)/cm^{-1} 2972, 2816, 2249, 1721, 1690, 1611, 1277, 1110, 1012, 858; ^{1}H NMR (400 MHz, $CDCl_3$) δ 8.01 (d, 2H, *J* = 8 Hz), 7.33–7.30 (m, 4H), 7.23–7.19 (m, 3H), 5.19 (br s, 1H), 3.96–3.93 (m, 4H) 3.24 (d, 1H, *J* = 12 Hz), 3.10 (td, 1H, *J* = 12 Hz, 4 Hz), 2.76 (d, 1H, *J* = 12 Hz), 2.65 (dd, 1H, *J* = 12 Hz, 4 Hz), 2.32 (td, 1H, *J* = 12 Hz, 4 Hz), 1.44 (s, 9H), 1.35 (s, 3H), 1.33 (s, 3H); ^{13}C NMR (100 MHz, $CDCl_3$) δ 167.1, 155.1, 148.1, 146.5, 129.4, 128.3, 127.9, 127.1, 126.4, 126.1, 80.0, 59.7, 54.4, 52.0, 49.4, 46.2, 41.3, 28.3, 23.8, 23.4; HRMS (ESI-TOF) m/z: [M+H]$^+$ Calcd for $C_{26}H_{35}N_2O_4$ 439.2597. Found 439.2612.

1-(tert-Butoxycarbonyl)-2-(4-acetylphenyl)-4-(2-phenylpropan-2-yl)piperazine (**2a**)

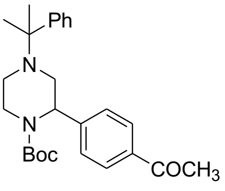

(±)-2a

Using general procedure B, carboxylic acid **1** (209 mg, 0.6 mmol), Ir[dF(CF_3)ppy]$_2$(dtbbpy)PF_6 (4.5 mg, 4 µmol), 4'-bromoacetophenone (199 mg, 1.0 mmol),

nickel(II) chloride·glyme (9 mg, 0.04 mmol), 4,4'-di-tert-butyl-2,2'-dipyridyl (16.1 mg, 0.06 mmol), cesium carbonate (196.5 mg, 0.6 mmol) and DMF (20 mL) gave, after purification by column chromatography on silica gel, eluting with hexane–EtOAc (9:1), the piperazine **2a** (203 mg, 80%) as a colourless oil; R_f = 0.70 [hexane–EtOAc (4:1)]; FT-IR ν_{max} (ATR)/cm^{-1} 2975, 2928, 2817, 1681, 1608, 1413, 1363, 1270, 1170, 770; ^{1}H NMR (400 MHz, $CDCl_3$) δ 7.93 (d, 2H, *J* = 8 Hz), 7.36–7.34 (m, 2H), 7.29–7.22 (m, 2H), 7.21–7.20 (m, 3H), 5.19 (br s, 1H), 3.96 (br d, 1H, *J* = 12 Hz), 3.25 (br d, 1H, *J* = 12 Hz), 3.08 (td, 1H, *J* = 12 Hz, 4 Hz), 2.76 (br d, 1H, 12 Hz), 2.67–2.65 (m, 4H), 2.32 (td, 1H, *J* = 12 Hz, 4 Hz), 1.44 (s, 9H), 1.36 (s, 3H), 1.33 (s, 3H); ^{13}C NMR (100 MHz, $CDCl_3$) δ 197.9, 155.1, 148.1, 146.8, 135.5, 128.2, 127.9, 127.3, 126.4, 126.2, 80.0, 59.9, 54.4, 49.4, 46.2, 41.3, 28.3, 26.6, 23.8, 23.4; HRMS (ESI-TOF) m/z: [M+H]$^+$ Calcd for $C_{26}H_{35}N_2O_3$ 423.2648. Found 423.2652.

1-(tert-Butoxycarbonyl)-4-(2-phenylpropan-2-yl)-2-(4-tert-butoxycarbonylphenyl)piperazine (**2b**)

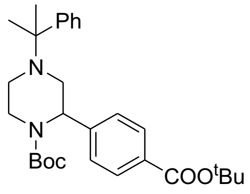

(±)-2b

Using general procedure B, carboxylic acid **1** (209 mg, 0.6 mmol), Ir[dF(CF_3)ppy]$_2$(dtbbpy)PF_6 (4.5 mg, 4 μmol), tert-butyl 4-bromobenzoate (258 mg, 1.0 mmol), nickel(II) chloride·glyme (9 mg, 0.04 mmol), 4,4'-di-tert-butyl-2,2'-dipyridyl (16.1 mg, 0.06 mmol), cesium carbonate (196.5 mg, 0.6 mmol) and DMF (20 mL) gave, after purification by column chromatography on silica gel, eluting with hexane–EtOAc (9:1), the piperazine **2b** (158 mg, 55%) as a yellow oil; R_f = 0.74 [hexane–EtOAc (4:1)]; FT-IR ν_{max} (ATR)/cm^{-1} 2975, 2930, 1697, 1611, 1455, 1415, 1392, 1366, 1299, 1167, 1114, 764, 701; ^{1}H NMR (400 MHz, $CDCl_3$) δ 7.96 (d, 2H, *J* = 8 Hz), 7.33–7.29 (m, 4H), 7.26–7.20 (m, 3H), 5.19 (br s, 1H), 3.94 (br d, 1H, *J* = 12 Hz), 3.28 (br d, 1H, *J* = 12 Hz), 3.07 (td, 1H, *J* = 12 Hz, 4 Hz), 2.74 (br d, 1H, *J* = 12 Hz), 2.66 (dd, 1H, *J* = 12 Hz, 4 Hz), 2.31 (td, 1H, *J* = 12 Hz, 4 Hz), 1.64 (s, 9H), 1.45 (s, 9H), 1.36 (s, 3H), 1.34 (s, 3H); ^{13}C NMR (100 MHz, $CDCl_3$) δ 165.8, 155.1, 148.1, 145.9, 130.2, 129.2, 128.0, 126.9, 126.4, 126.2, 80.8, 79.9, 59.7, 54.3 49.3, 46.3, 41.1, 28.3, 28.2, 23.9, 23.3; HRMS (ESI-TOF) m/z: [M+H]$^+$ Calcd for $C_{29}H_{41}N_2O_4$ 481.3066. Found 481.3070. The enantiomers were resolved using HPLC (Cellulose–2, n-hexane–isopropanol = 99:1, flow rate = 0.5 mL/min, l = 254 nm), t_R = 16.7 min and 19.3 min.

1-(tert-Butoxycarbonyl)-2-(4-fluorophenyl)-4-(2-phenylpropan-2-yl)piperazine (**2c**)

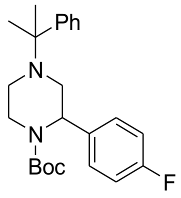

(±)-2c

Using general procedure B, carboxylic acid **1** (209 mg, 0.6 mmol), Ir[dF(CF_3)ppy]$_2$(dtbbpy)PF_6 (4.5 mg,4 µmol), 1-fluoro-4-iodobenzene (0.12 mL, 1.0 mmol), nickel(II) chloride·glyme (9 mg, 0.04 mmol), 4,4'-di-tert-butyl-2,2'-dipyridyl (16.1 mg, 0.06 mmol), cesium carbonate (196.5 mg, 0.6 mmol) and DMF (20 mL) gave, after purification by column chromatography on silica gel, eluting with hexane–EtOAc (9:1), the piperazine **2c** (131 mg, 55%) as a colourless oil; R_f = 0.84 [hexane–EtOAc (4:1)]; FT-IR ν_{max} (ATR)/cm^{-1} 2974, 2930, 2816, 1690, 1602, 1508, 1415, 1364, 1158, 1013, 701; ^{1}H NMR (400 MHz, $CDCl_3$) δ 7.37–7.35 (m, 2H), 7.33–7.20 (m, 5H), 7.04–7.00 (m, 2H), 5.16 (br s, 1H), 3.92 (br d, 1H, *J* = 12 Hz), 3.22 (br d, 1H, *J* = 12 Hz), 3.08 (td, 1H, *J* = 12 Hz, 4 Hz), 2.76 (br d, 1H, *J* = 12 Hz), 2.64 (dd, 1H, *J* = 12 Hz, 4 Hz), 2.32 (td, 1H, *J* = 12 Hz, 4 Hz), 1.47 (s, 9H), 1.38 (s, 3H), 1.37 (s, 3H); ^{13}C NMR (100 MHz, $CDCl_3$) δ 161.6 (d, *J* = 243 Hz), 155.0, 148.2, 136.7, 128.8 (d, *J* = 16 Hz), 127.9, 126.4, 126.2, 114.7 (d, *J* = 21 Hz), 79.8, 59.8, 53.6, 49.4, 46.4, 40.9, 28.4, 23.7, 23.4; ^{19}F NMR (377 MHz, $CDCl_3$) δ –116.87; HRMS (ESI-TOF) m/z: [M+H]$^+$ Calcd for $C_{24}H_{32}FN_2O_2$ 399.2448. Found 399.2447. The enantiomers were resolved using HPLC (Amylose–2, n-hexane–isopropanol = 95:5, flow rate = 0.5 mL/min, l = 254 nm), t_R = 11.4 min and 14.3 min.

1-(tert-Butoxycarbonyl)-4-(2-phenylpropan-2-yl)-2-(4-trifluoromethylphenyl)piperazine (**2d**)

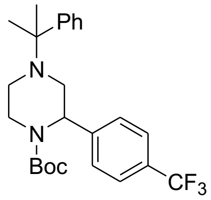

(±)-2d

Using general procedure B, carboxylic acid **1** (209 mg, 0.6 mmol), Ir[dF(CF_3)ppy]$_2$(dtbbpy)PF_6 (4.5 mg, 4 µmol), 4-bromobenzotrifluoride (0.14 mL, 1.0 mmol), nickel(II) chloride·glyme (9 mg, 0.04 mmol), 4,4'-di-tert-butyl-2,2'-dipyridyl (16.1 mg, 0.06 mmol), cesium carbonate (196.5 mg, 0.6 mmol) and DMF (20 mL) gave, after purification by column chromatography on silica gel, eluting with hexane–EtOAc (9:1), the piperazine **2d** (91 mg, 34%) as a colourless oil; R_f = 0.80 [hexane–EtOAc (4:1)]; FT-IR ν_{max} (ATR)/cm^{-1} 2975, 2931, 2814, 1693, 1620, 1322, 1265, 1118, 1068, 759; ^{1}H NMR (400 MHz, $CDCl_3$) δ 7.59 (d, 2H, *J* = 8 Hz), 7.37 (d, 2H, *J* = 8 Hz), 7.31–7.29 (m, 2H), 7.24–7.22 (m, 3H), 5.20 (br s, 1H), 3.98 (br d, 1H, *J* = 12 Hz), 3.20 (br d, 1H, *J* = 12 Hz), 3.12 (td, 1H, *J* = 12 Hz, 4 Hz), 2.82 (br d, 1H, *J* = 12 Hz), 2.67 (dd, 1H, *J* = 12 Hz, 4 Hz), 2.34 (td, 1H, *J* = 12 Hz, 4 Hz), 1.46 (s, 9H), 1.38 (s, 3H), 1.35 (s,

3H); ^{13}C NMR (100 MHz, $CDCl_3$) δ 155.1, 148.0, 145.2, 128.7 (q, *J* = 32 Hz), 127.9, 127.4, 126.4, 126.2, 124.9 (q, *J* = 40 Hz), 124.3 (q, *J* = 270 Hz), 80.0, 59.8, 54.1, 49.5, 46.2, 41.3, 28.3, 24.3, 22.8; ^{19}F NMR (377 MHz, $CDCl_3$) δ –62.3; HRMS (ESI-TOF) m/z: $[M+H]^+$ Calcd for $C_{25}H_{32}F_3N_2O_2$ 449.2416. Found 449.2413.

1-(tert-Butoxycarbonyl)-2-(4-cyanophenyl)-4-(2-phenylpropan-2-yl)piperazine (**2e**)

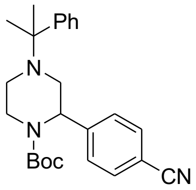

(±)-2e

Using general procedure B, carboxylic acid **1** (209 mg, 0.6 mmol), Ir[dF(CF_3)ppy]$_2$(dtbbpy)PF_6 (4.5 mg, 4 µmol), 4-bromobenzonitrile (182 mg, 1.0 mmol), nickel(II) chloride·glyme (9 mg, 0.04 mmol), 4,4'-di-tert-butyl-2,2'-dipyridyl (16.1 mg, 0.06 mmol), cesium carbonate (196.5 mg, 0.6 mmol) and DMF (20 mL) gave, after purification by column chromatography on silica gel, eluting with hexane–EtOAc (9:1), the piperazine **2e** (170 mg, 70%) as a colourless oil; R_f = 0.82 [hexane–EtOAc (4:1)]; FT-IR νmax (ATR)/cm^{-1} 2975, 2818, 2228, 1608, 1687, 1493, 1164, 769; ^{1}H NMR (400 MHz, $CDCl_3$) δ 7.62 (d, 2H, *J* = 8 Hz), 7.33 (d, 2H, *J* = 8 Hz), 7.27–7.26 (m, 2H), 7.24–7.21 (m, 3H), 5.17 (br s, 1H), 3.96 (br d, 1H, *J* = 12 Hz), 3.16 (br d, 1H, *J* = 12 Hz), 3.07 (td, 1H, *J* = 12 Hz, 4 Hz), 2.80 (br d, 1H, *J* = 12 Hz), 2.65 (dd, 1H, *J* = 12 Hz, 4 Hz), 2.34 (td, 1H, *J* = 12 Hz, 4 Hz), 1.44 (s, 9H), 1.36 (s, 3H), 1.34 (s, 3H); ^{13}C NMR (100 MHz, $CDCl_3$) δ 155.0, 147.9, 146.8, 131.9, 127.9, 127.8, 126.5, 126.1, 119.0, 110.3, 80.2, 59.7, 54.3, 49.5, 46.1, 41.4, 28.3, 24.4, 22.6; HRMS (ESI-TOF) m/z: $[M+H]^+$ Calcd for $C_{25}H_{32}N_3O_2$ 406.2495. Found 406.2495.

1-(tert-Butoxycarbonyl)-2-(4-chlorophenyl)-4-(2-phenylpropan-2-yl)piperazine (**2f**)

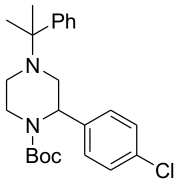

(±)-2f

Using general procedure B, carboxylic acid **1** (209 mg, 0.6 mmol), Ir[dF(CF_3)ppy]$_2$(dtbbpy)PF_6 (4.5 mg, 4 µmol), 1-chloro-4-iodobenzene (240 mg, 1.0 mmol), nickel(II) chloride·glyme (9 mg, 0.04 mmol), 4,4'-di-tert-butyl-2,2'-dipyridyl (16.1 mg, 0.06 mmol), cesium carbonate (196.5 mg, 0.6 mmol) and DMF (20 mL) gave, after purification by column chromatography on silica gel, eluting with hexane–EtOAc (9:1), the piperazine **2f** (138 mg, 58%) as a colourless oil; R_f = 0.7 [hexane–EtOAc (4:1)]; FT-IR ν_{max} (ATR)/cm^{-1} 2975, 2933,

2821, 1768, 1737, 1695, 1468, 1365, 1239, 1163, 964, 763; ^{1}H NMR (400 MHz, $CDCl_3$) δ 7.35–7.23 (m, 2H), 7.31–7.26 (m, 2H), 7.24–7.21 (m, 5H), 5.13 (br s, 1H), 3.92 (br d, 1H, *J* = 12 Hz), 3.20 (br d, 1H, *J* = 12 Hz), 3.05 (td, 1H, *J* = 12 Hz, 4 Hz), 2.76 (br d, 1H, *J* = 12 Hz), 2.63 (dd, 1H, *J* = 12 Hz, 4 Hz), 2.31 (td, 1H, *J* = 12 Hz, 4 Hz), 1.45 (s, 9H), 1.37 (s, 3H), 1.35 (s, 3H); ^{13}C NMR (100 MHz, $CDCl_3$) δ 155.0, 148.1, 139.5, 132.1, 128.7, 128.1, 127.9, 126.4, 126.2, 79.9, 59.8, 53.7, 49.3, 46.3, 41.0, 28.4, 23.6, 23.5; HRMS (ESI-TOF) m/z: $[M+H]^+$ Calcd for $C_{24}H_{32}{}^{35}ClN_2O_2$ 415.2152. Found 415.2151. The enantiomers were resolved using HPLC (Amylose–2, n-hexane–isopropanol = 95:5, flow rate = 0.5 mL/min, l = 254 nm), t_R = 5.8 min and 7.2 min.

For reaction on a larger scale:

Using general procedure B (reaction time 72 h), carboxylic acid **1** (2 g, 5.7 mmol), $Ir[dF(CF_3)ppy]_2(dtbbpy)PF_6$ (42.7 mg, 38 µmol), 1-chloro-4-iodobenzene (2.3 g, 9.5 mmol), nickel(II) chloride·glyme (85.5 mg, 0.4 mmol), 4,4'-di-tert-butyl-2,2'-dipyridyl (153 mg, 0.6 mmol), cesium carbonate (1.8 g, 5.7 mmol) and DMF (150 mL) gave, after purification by column chromatography on silica gel, eluting with hexane–EtOAc (9:1), the piperazine **2f** (1.2 g, 50%) as a colourless oil; data as above.

1-(tert-Butoxycarbonyl)-2-phenyl-4-(2-phenylpropan-2-yl)piperazine (**2g**)

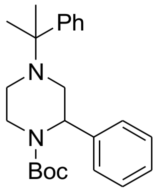

(±)-2g

Using general procedure B, carboxylic acid **1** (209 mg, 0.6 mmol), $Ir[dF(CF_3)ppy]_2(dtbbpy)PF_6$ (4.5 mg, 4 µmol), iodobenzene (0.12 mL, 1.0 mmol), nickel(II) chloride·glyme (9 mg, 0.04 mmol), 4,4'-di-tert-butyl-2,2'-dipyridyl (16.1 mg, 0.06 mmol), cesium carbonate (196.5 mg, 0.6 mmol) and DMF (20 mL) gave, after purification by column chromatography on silica gel, eluting with hexane–EtOAc (9:1), the piperazine **2g** (68 mg, 30%) as a colourless oil; R_f = 0.9 [hexane–EtOAc (4:1)]; FT-IR ν_{max} (ATR)/cm^{-1} 2973, 2927, 2814, 1690, 1413, 1164, 698; ^{1}H NMR (400 MHz, $CDCl_3$) δ 7.38–7.29 (m, 6H), 7.26–7.19 (m, 4H), 5.20 (br s, 1H), 3.94 (d, 1H, *J* = 12 Hz), 3.29 (d, 1H, *J* = 12 Hz), 3.12 (td, 1H, *J* = 12, 4 Hz), 2.75 (br d, 1H, *J* = 12 Hz), 2.67 (dd, 1H, *J* = 12 Hz, 4 Hz), 2.33 (td, 1H, *J* = 12 Hz, 4 Hz), 1.47 (s, 9H), 1.39 (s, 3H), 1.36 (s, 3H); ^{13}C NMR (100 MHz, $CDCl_3$) δ 155.2, 148.4, 140.9, 128.0, 127.9, 127.2, 126.4, 126.32, 126.3, 79.7, 59.8, 54.3, 49.2, 46.4, 41.0, 28.4, 24.0, 23.3; HRMS (ESI-TOF) m/z: $[M+H]^+$ Calcd for $C_{24}H_{33}N_2O_2$ 381.2542. Found 381.2542. The enantiomers were resolved using HPLC (Cellulose–2, n-hexane–isopropanol = 99:1, flow rate = 0.5 mL/min, l = 254 nm), t_R = 13.0 and 19.0 min.

1-(tert-Butoxycarbonyl)-2-(3-methoxyphenyl)-4-(2-phenylpropan-2-yl)piperazine (**2h**)

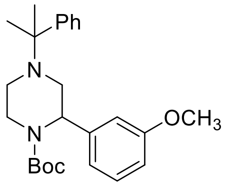

(±)-**2h**

Using general procedure B, carboxylic acid **1** (627 mg, 1.8 mmol), Ir[dF(CF_3)ppy]$_2$(dtbbpy)PF_6 (13.5 mg, 12 µmol), 3-iodoanisole (0.38 mL, 3.0 mmol), nickel(II) chloride·glyme (27 mg, 0.12 mmol), 4,4'-di-tert-butyl-2,2'-dipyridyl (48.3 mg, 0.18 mmol), cesium carbonate (589 mg, 1.8 mmol) and DMF (40 mL) gave, after purification by column chromatography on silica gel, eluting with hexane–EtOAc (9:1), the piperazine **2h** (310 mg, 42%) as a colourless oil; R_f = 0.75 [hexane–EtOAc (4:1)]; FT-IR ν_{max} (ATR)/cm^{-1} 2973, 2936, 2812, 1693, 1602, 1414, 1169, 766; ^{1}H NMR (400 MHz, $CDCl_3$) δ 7.38–7.36 (m, 2H), 7.27–7.18 (m, 4H), 6.93–6.91 (m, 2H), 6.84–6.81 (m, 1H), 5.17 (br s, 1H), 3.92 (d, 1H, *J* = 12 Hz), 3.81 (s, 3H), 3.28 (d, 1H, *J* = 12 Hz), 3.12 (td, 1H, *J* = 12, 4 Hz), 2.72 (br d, 1H, *J* = 12 Hz), 2.65 (dd, 1H, *J* = 12 Hz, 4 Hz), 2.30 (td, 1H, *J* = 12 Hz, 4 Hz), 1.46 (s, 9H), 1.39 (s, 3H), 1.35 (s, 3H); ^{13}C NMR (100 MHz, $CDCl_3$) δ 159.5, 155.1, 148.4, 142.7, 128.9, 127.9, 126.29, 126.26, 119.7, 112.9, 112.0, 79.7, 59.8, 55.1, 54.2, 49.2, 46.4, 41.0, 28.4, 24.4, 23.0; HRMS (ESI-TOF) m/z: [M+H]$^+$ Calcd for $C_{25}H_{35}N_2O_3$ 411.2648. Found 411.2650.

1-(tert-Butoxycarbonyl)-2-(4-methylphenyl)-4-(2-phenylpropan-2-yl)piperazine (**2i**)

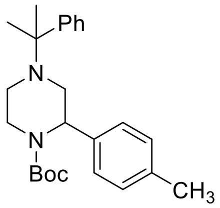

(±)-**2i**

Using general procedure B, carboxylic acid **1** (627 mg, 1.8 mmol), Ir[dF(CF_3)ppy]$_2$(dtbbpy)PF_6 (13.5 mg, 12 µmol), 4-iodotoluene (654 mg, 3.0 mmol), nickel(II) chloride·glyme (27 mg, 0.12 mmol), 4,4'-di-tert-butyl-2,2'-dipyridyl (48.3 mg, 0.18 mmol), cesium carbonate (589 mg, 1.8 mmol) and DMF (40 mL) gave, after purification by column chromatography on silica gel, eluting with hexane–EtOAc (9:1), the piperazine **2i** (248 mg, 35%) as a colourless oil; R_f = 0.9 [hexane–EtOAc (4:1)]; FT-IR ν_{max} (ATR)/cm^{-1} 2973, 2814, 1688, 1414, 1390, 1111, 1163, 700; ^{1}H NMR (400 MHz, $CDCl_3$) δ 7.41–7.39 (m, 2H), 7.29–7.22 (m, 5H), 7.16 (d, 2H, *J* = 8 Hz), 5.18 (br s, 1H), 3.92 (d, 1H, *J* = 12 Hz), 3.29 (d, 1H, *J* = 12 Hz), 3.08 (td, 1H, *J* = 12, 4 Hz), 2.72 (dd, 1H, *J* = 12, 4Hz), 2.65 (dd, 1H, *J* = 12 Hz, 4 Hz), 2.40 (s, 3H), 2.31 (td, 1H, *J* = 12 Hz, 4 Hz), 1.48 (s, 9H), 1.39 (s, 3H), 1.37 (s, 3H); ^{13}C NMR (100 MHz, $CDCl_3$) δ 155.1, 148.4, 137.8, 135.8, 128.7, 127.9, 127.2, 126.35, 126.3, 79.6, 59.8, 53.9, 49.0, 46.5, 40.8, 28.4, 24.3, 23.0, 21.0; HRMS

(ESI-TOF) m/z: $[M+H]^+$ Calcd for $C_{25}H_{35}N_2O_2$ 395.2699. Found 395.2700. The enantiomers were resolved using HPLC (Amylose–2, n-hexane–isopropanol = 95:5, flow rate = 0.5 mL/min, l = 254 nm), t_R = 12.9 and 18.7 min

2.4 Racemic lithiation–trapping

Table S1 shows the lithiation of substrates **2b**, **2c**, **2f**–**g** with trapping after different times with methyl chloroformate. In all cases, yields of the disubstituted products were similar, indicating that lithiation is rapid at low temperature.

Table S1. Optimization of the lithiation–trapping

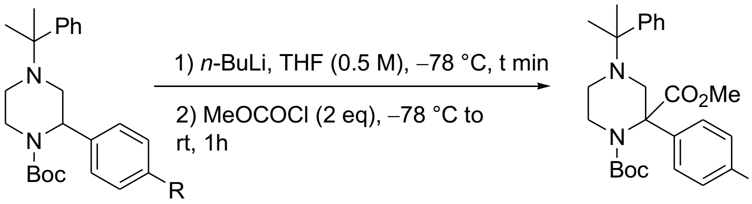

Entry	R	n-BuLi (equiv)	t (min)	Yield (%)
1	Cl	1.2	5	52
2	Cl	1.2	10	56
3	Cl	1.2	20	55
4	Cl	1.6	10	52
5	F	1.2	10	58
6	H	1.2	10	60
7	^{t}BuCOO	1.2	10	57
8	CH_3	1.2	10	62

2-(4-tert-Butoxycarbonyl-phenyl)-4-(1-methyl-1-phenylethyl)-piperazine-1,2-dicarboxylic acid 1-tert-butyl ester 2-methyl ester (**3b**)

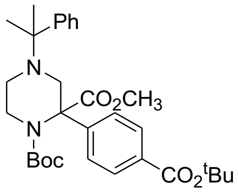

(±)-**3b**

n-BuLi (0.11 mL, 0.26 mmol, 1.2 eqv, 2.4 M in hexanes) was added to piperazine **2b** (100 mg, 0.22 mmol) in dry THF (1 mL) at –78 °C. After 10 min, MeOCOCl (0.04 mL, 0.44 mmol) was added. The mixture was allowed to warm to room temperature. After 1 h, MeOH (2 mL) was

added. The solvent was evaporated and the residue was purified by column chromatography on silica gel, eluting with hexane–EtOAc (9:1) to give the piperazine **3b** (62 mg, 55%) as a yellow oil; R_f = 0.6 [hexane–EtOAc (4:1)]; FT-IR ν_{max} (ATR)/cm^{-1} 2973, 2924, 2877, 2829, 1748, 1709, 1366, 1117, 701; ^{1}H NMR (400 MHz, $CDCl_3$) δ 7.93 (d, 2H, *J* = 8 Hz), 7.43 (d, 2H, *J* = 8 Hz), 7.32–7.30 (m, 2H), 7.27–7.20 (m, 3H), 3.90 (s, 3H), 3.72 (br s, 1H), 3.40 (br s, 1H), 3.32 (d, 1H, *J* = 12 Hz), 2.67-2.61 (m, 2H), 2.46 (td, 1H, *J* = 12 Hz, 4 Hz), 1.62 (s, 9H), 1.30 (s, 3H), 1.27 (s, 3H), 1.14 (br s, 9H); ^{13}C NMR (100 MHz, $CDCl_3$, two C could not be observed) δ = 171.1, 165.6, 156.6, 147.8, 130.2, 128.9, 128.0, 126.5, 126.1, 125.9, 80.9, 80.5, 59.5, 58.8, 52.4, 46.3, 44.4, 28.2, 27.8, 26.4, 21.2); HRMS (ESI-TOF) m/z: $[M+H]^+$ Calcd for $C_{31}H_{43}N_2O_6$ is 539.3121. Found 539.3143. The enantiomers were resolved using HPLC (Cellulose–2, n-hexane–isopropanol = 95:5, flow rate = 1.0 mL/min, l = 254 nm), t_R = 5.1 min and 7.5 min.

2-(4-Fluorophenyl)-4-(1-methyl-1-phenylethyl)-piperazine-1,2-dicarboxylic acid 1-tert-butyl ester 2-methyl ester (**3c**)

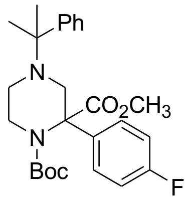

(±)-**3c**

In the same way as piperazine **3b**, n-BuLi (0.12 mL, 0.30 mmol, 1.2 eqv, 2.4 M in hexanes), piperazine **2c** (100 mg, 0.25 mmol) and MeOCOCl (0.041 mL, 0.46 mmol) gave, after purification by column chromatography on silica gel, eluting with hexane–EtOAc (9:1), the piperazine **3c** (72 mg, 63%) as a colourless oil; R_f = 0.6 [hexane–EtOAc (4:1)]; FT-IR ν_{max} (ATR)/cm^{-1} 2975, 2880, 2822, 1746, 1698, 1508, 1365, 1294, 1246, 1165, 766, 701; ^{1}H NMR (400 MHz, $CDCl_3$) δ 7.34–7.31 (m, 4H), 7.27–7.20 (m, 3H), 7.03–6.97 (m, 2H), 3.89 (s, 3H), 3.70 (br s, 1H), 3.42 (br s, 1H), 3.30 (d, 1H, *J* = 12 Hz), 2.67-2.64 (m, 2H), 2.48 (td, 1H, *J* = 12 Hz, 4 Hz), 1.31 (s, 3H), 1.29 (s, 3H), 1.19 (br s, 9H); ^{13}C NMR (100 MHz, $CDCl_3$, two C could not be observed) δ 171.5, 161.5 (d, *J* = 243 Hz), 156.7, 147.9, 128.1, 128.0, 126.4, 125.9, 114.4 (d, *J* = 21 Hz), 80.3, 59.5, 58.8, 52.3, 46.2, 44.4, 27.8, 26.4, 21.2; ^{19}F NMR (377 MHz, $CDCl_3$) δ – 116.73; HRMS (ESI-TOF) m/z: $[M+H]^+$ Calcd for $C_{26}H_{34}N_2O4$ is 457.2503. Found 457.2521. The enantiomers were resolved using HPLC (Amylose–2, n-hexane–isopropanol = 98:2, flow rate = 0.5 mL/min, l = 254 nm), t_R = 17.6 min and 19.8 min. In addition, a small amount of the alkene **B** was obtained (9 mg, 8%) as an oil, which was partially characterized as follows: R_f = 0.4 [hexane–EtOAc (4:1)]; ^{1}H NMR (400 MHz, $CDCl_3$) δ 7.89–7.86 (m, 3H), 7.61–7.59 (m, 2H), 7.13–7.08 (m, 4H), 5.91 (br s, 1H), 5.89 (br s, 1H), 3.92 (s, 3H), 3.07–3.03 (m, 2H), 2.70–2.67 (m, 2H), 1.47 (s, 3H), 1.43 (s, 3H), 1.28 (s, 9H).

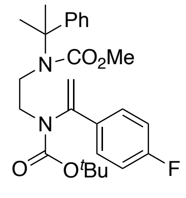

B

2-(4-Chlorophenyl)-4-(1-methyl-1-phenylethyl)-piperazine-1,2-dicarboxylic acid 1-tert-butyl ester 2-methyl ester (**3f**)

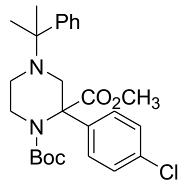

(±)-**3f**

In the same way as piperazine **3b**, n-BuLi (0.12 mL, 0.29 mmol, 1.2 eqv, 2.4 M in hexanes), piperazine **2f** (100 mg, 0.24 mmol) and MeOCOCl (0.042 mL, 0.48 mmol) gave, after purification by column chromatography on silica gel, eluting with hexane–EtOAc (9:1), the piperazine **3f** (68 mg, 60%) as a colourless oil; R_f = 0.6 [hexane–EtOAc (4:1)]; FT-IR ν_{max} (ATR)/cm^{-1} 2980, 2925, 1853, 1688, 1592, 1457, 1392, 1367, 1249, 1163, 765, 700; ^1H NMR (400 MHz, $CDCl_3$) δ 7.32–7.30 (m, 3H), 7.27–7.20 (m, 6H), 3.88 (s, 3H), 3.69 (br s, 1H), 3.42 (br s, 1H), 3.30 (d, 1H, *J* = 12 Hz), 2.67-2.59 (m, 2H), 2.48 (td, 1H, *J* = 12 Hz, 4 Hz), 1.30 (s, 3H), 1.28 (s, 3H), 1.18 (br s, 9H); ^{13}C NMR (100 MHz, $CDCl_3$) δ 171.3, 156.6, 147.8, 132.3, 128.3, 128.0, 127.85, 127.8, 126.4, 125.9, 80.5, 59.5, 58.8, 53.5, 52.5, 46.2, 44.4, 27.9, 26.5, 21.1; HRMS (ESI-TOF) m/z: $[M+H]^+$ Calcd for $C_{26}H_{34}{}^{35}ClN_2O_4$ is 473.2207. Found 473.2209. The enantiomers were resolved using HPLC (Amylose–2, n-hexane–isopropanol = 95:5, flow rate = 1.0 mL/min, l = 254 nm), t_R = 14.5 min and 18.8 min.

4-(1-Methyl-1-phenylethyl)-2-phenyl-piperazine-1,2-dicarboxylic acid 1-tert-butyl ester 2-methyl ester (**3g**)

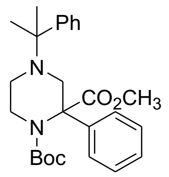

(±)-**3g**

In the same way as piperazine **3b**, n-BuLi (0.14 mL, 0.31 mmol, 1.2 eqv, 2.4 M in hexanes), piperazine **2g** (100 mg, 0.26 mmol) and MeOCOCl (0.046 mL, 0.52 mmol) gave, after purification by column chromatography on silica gel, eluting with hexane–EtOAc (9:1), the piperazine **3g** (69 mg, 60%) as a colourless oil; R_f = 0.7 [hexane–EtOAc (4:1)]; FT-IR ν_{max} (ATR)/cm^{-1} 2975, 2930, 2877, 1744, 1695, 1493, 1390, 1364, 1345, 1245, 1165, 1021, 766, 699; ^1H NMR (400 MHz, $CDCl_3$) δ 7.33–7.22 (m, 8H), 7.02–6.98 (m, 2H), 3.89 (s, 3H), 3.70 (br s, 1H), 3.42 (br s, 1H), 3.31 (d, 1H, *J* = 12 Hz), 2.66-2.64 (m, 2H), 2.52–2.45 (m, 1H), 1.31 (s, 3H), 1.29 (s, 3H), 1.19 (br s, 9H); ^{13}C NMR (100 MHz, $CDCl_3$) δ 171.4, 157.9, 148.1, 128.0, 127.7, 126.5, 126.4, 126.2, 126.0, 80.1, 59.5, 58.9, 53.9, 52.2, 46.4, 44.4, 27.8, 26.7, 21.1; HRMS (ESI-TOF) m/z: $[M+H]^+$ Calcd for $C_{26}H_{35}N_2O_4$ is 439.2597; Found 439.2587. The enantiomers were resolved using HPLC (Chiralpak-AD, n-hexane–isopropanol = 95:5, flow rate = 0.5 mL/min, l = 254 nm), t_R = 10.1 min and 13.2 min.

2-(4-Methylphenyl)-4-(1-methyl-1-phenylethyl)-piperazine-1,2-dicarboxylic acid 1-tert-butyl ester 2-methyl ester (**3i**)

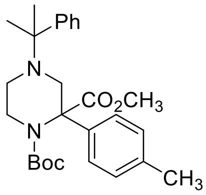

(±)-**3i**

In the same way as piperazine **3b**, n-BuLi (0.14 mL, 0.30 mmol, 1.2 eqv, 2.4 M in hexanes), piperazine **2i** (100 mg, 0.25 mmol) and MeOCOCl (0.044 mL, 0.50 mmol) gave, after purification by column chromatography on silica gel, eluting with hexane–EtOAc (9:1), the piperazine **3i** (71 mg, 62%) as a colourless oil; R_f = 0.85 [hexane–EtOAc (4:1)]; FT-IR ν_{max} (ATR)/cm^{-1} 2972, 2932, 2877, 1744, 1698, 1364, 1345, 1244, 1166, 1020, 766, 701; ^{1}H NMR (400 MHz, $CDCl_3$) δ 7.36–7.34 (m, 2H), 7.29–7.20 (m, 5H), 7.12 (d, 2H, *J* = 8 Hz), 3.90 (s, 3H), 3.75–3.71 (m, 1H), 3.38–3.35 (m, 2H), 2.66–2.63 (m, 2H), 2.46 (td, 1H, *J* = 12, 4 Hz), 2.37 (s, 3H), 1.31 (s, 3H), 1.29 (s, 3H), 1.16 (br s, 9H); ^{13}C NMR (100 MHz, $CDCl_3$, one C could not be observed) δ 171.6, 156.9, 148.2, 136.1, 128.4, 128.0, 126.4, 126.2, 126.0, 80.0, 70.2, 59.5, 58.8, 52.2, 46.4, 44.5, 27.9, 26.7, 21.1, 20.9; HRMS (ESI-TOF) m/z: $[M+H]^+$ Calcd for $C_{27}H_{37}N_2O_4$ is 453.2753; Found 453.2795. The enantiomers were resolved using HPLC (Amylose–2, n-hexane–isopropanol = 95:5, flow rate = 0.5 mL/min, l = 254 nm), t_R = 13.4 min and 15.1 min.

2.5 Kinetic resolution

Table S2. Optimization of kinetic resolution

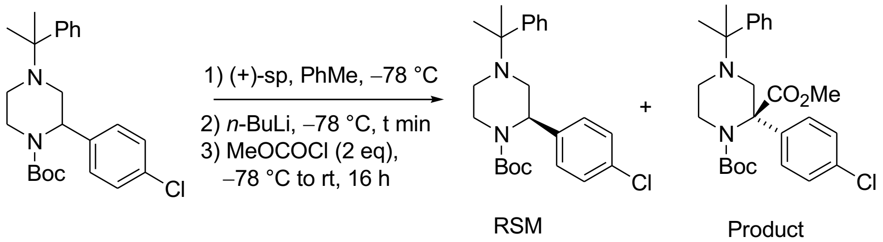

Entry	n-BuLi (eqv)	(+)-Sp (eqv)	t min	RSM % Yield	RSM er	Product % Yield	Product er
1	0.6	0.8	5	52	66:34	22	77:23
2	0.6	0.8	10	50	78:22	28	80:20
3	0.6	0.8	20	40	94:6	30	92:8
**4**	**0.6**	**0.8**	**30**	**38**	**99:1**	**28**	**99:1**
5	0.8	0.8	30	32	95:5	22	82:18

Kinetic resolution of piperazine **2b**

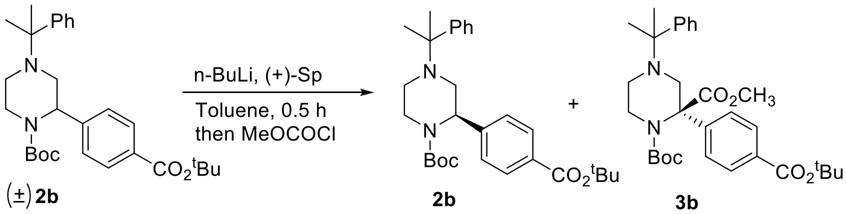

(±) **2b** **2b** **3b**

n-BuLi (0.10 mL, 0.25 mmol, 2.4 M in hexanes) was added to a mixture of (+)-sparteine (78 mg, 0.33 mmol) and the racemic piperazine **2b** (200 mg, 0.42 mmol) in dry PhMe (5.3 mL) at –78 °C. After 0.5 h, MeOCOCl (0.07 mL, 0.84 mmol) was added and the mixture was allowed to warm to room temperature. After 1 h, MeOH (1 mL) was added. The solvent was evaporated and the residue was purified by column chromatography on silica gel, eluting with n-hexane–EtOAc (90:10), to give enantioenriched piperazine **2b** (82 mg, 41%) as a yellow oil; data as above; er 90:10 by CSP-HPLC as described above (major component eluted at 19.2 min); $[\alpha]_D^{20}$ –64 (*c* 1.0, $CHCl_3$). In addition, the ester **3b** (69 mg, 31%) was isolated as a yellow oil, data as above; er 92:8 by CSP-HPLC (major component eluted at 5.1 min); $[\alpha]_D^{20}$ +75 (*c* 1.0, $CHCl_3$).

Kinetic resolution of piperazine **2c**

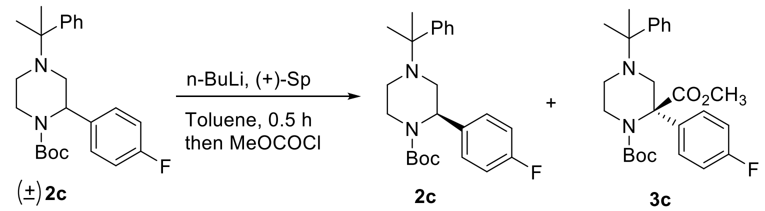

n-BuLi (0.12 mL, 0.30 mmol, 2.4 M in hexanes) was added to a mixture of (+)-sparteine (94 mg, 0.40 mmol) and the racemic piperazine **2c** (200 mg, 0.50 mmol) in dry PhMe (6.2 mL) at –78 °C. After 0.5 h, MeOCOCl (0.08 mL, 1.0 mmol) was added and the mixture was allowed to warm to room temperature. After 1 h, MeOH (1 mL) was added. The solvent was evaporated and the residue was purified by column chromatography on silica gel, eluting with n-hexane–EtOAc (90:10), to give enantioenriched piperazine **2c** (80 mg, 40%) as a colourless oil; data as above; er 98:2 by CSP-HPLC as described above (major component eluted at 11.2 min); $[\alpha]_{\mathrm{D}}^{20}$ –52 (*c* 1.0, $CHCl_3$). In addition, the ester **3c** (68 mg, 30%) was isolated as a colourless oil, data as above; er 98:2 by CSP-HPLC (major component eluted at 16.9 min); $[\alpha]_{\mathrm{D}}^{20}$ +82 (*c* 1.0, $CHCl_3$).

Kinetic resolution of piperazine **2f**

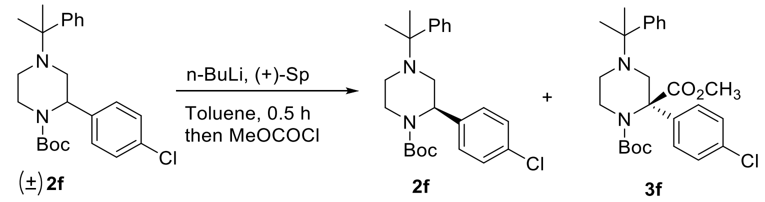

n-BuLi (0.12 mL, 0.29 mmol, 2.4 M in hexanes) was added to a mixture of (+)-sparteine (90 mg, 0.38 mmol) and the racemic piperazine **2f** (200 mg, 0.48 mmol) in dry PhMe (6 mL) at –78 °C. After 0.5 h, MeOCOCl (0.08 mL, 0.96 mmol) was added and the mixture was allowed to warm to room temperature. After 1 h, MeOH (1 mL) was added. The solvent was evaporated and the residue was purified by column chromatography on silica gel, eluting with n-hexane–EtOAc (90:10), to give enantioenriched piperazine **2f** (76 mg, 38%) as a colourless oil; data as above; er 99:1 by CSP-HPLC as described above (major component eluted at 4.0 min); $[\alpha]_{\mathrm{D}}^{20}$ –46 (*c* 1.0, $CHCl_3$). In addition, the ester **3f** (64 mg, 28%) was isolated as a colourless oil, data as above; er 99:1 by CSP-HPLC (major component eluted at 14.9 min); $[\alpha]_{\mathrm{D}}^{20}$ +28 (*c* 1.0, $CHCl_3$).

Kinetic resolution of piperazine **2g**

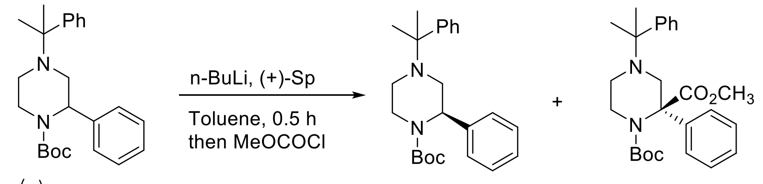

(±)**2g** **2g** **3g**

n-BuLi (0.13 mL, 0.32 mmol, 2.4 M in hexanes) was added to a mixture of (+)-sparteine (98 mg, 0.42 mmol) and the racemic piperazine **2g** (200 mg, 0.53 mmol) in dry PhMe (6.6 mL) at –78 °C. After 0.5 h, MeOCOCl (0.09 mL, 1.06 mmol) was added and the mixture was allowed to warm to room temperature. After 1 h, MeOH (1 mL) was added. The solvent was evaporated and the residue was purified by column chromatography on silica gel, eluting with n-hexane–EtOAc (90:10), to give enantioenriched piperazine **2g** (88 mg, 44%) as a colourless oil; data as above; er 82:18 by CSP-HPLC as described above (major component eluted at 12.5 min); $[\alpha]_{\mathrm{D}}^{20}$ –32 (*c* 1.0, $CHCl_3$). In addition, the ester **3g** (69 mg, 30%) was isolated as a colourless oil, data as above; er 99:1 by CSP-HPLC (major component eluted at 12.3 min); $[\alpha]_{\mathrm{D}}^{20}$ +62 (*c* 1.0, $CHCl_3$).

Kinetic resolution of piperazine **2i**

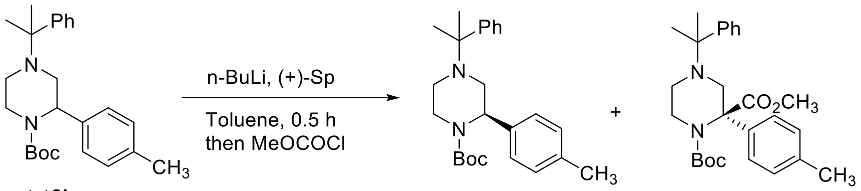

(±)**2i** **2i** **3i**

n-BuLi (0.13 mL, 0.30 mmol, 2.4 M in hexanes) was added to a mixture of (+)-sparteine (97 mg, 0.41 mmol) and the racemic piperazine **2i** (200 mg, 0.51 mmol) in dry PhMe (6.4 mL) at –78 °C. After 0.5 h, MeOCOCl (0.10 mL, 1.2 mmol) was added and the mixture was allowed to warm to room temperature. After 1 h, MeOH (1 mL) was added. The solvent was evaporated and the residue was purified by column chromatography on silica gel, eluting with n-hexane–EtOAc (90:10), to give enantioenriched piperazine **2i** (88 mg, 44%) as a colourless oil; data as above; er 99:1 by CSP-HPLC as described above (major component eluted at 13.4 min); $[\alpha]_{\mathrm{D}}^{20}$ –56 (*c* 1.0, $CHCl_3$). In addition, the ester **3i** (73 mg, 32%) was isolated as a colourless oil, data as above; er 96:4 by CSP-HPLC (major component eluted at 13.4 min); $[\alpha]_{\mathrm{D}}^{20}$ +36 (*c* 1.0, $CHCl_3$).

2.6 Further functionalization

2-(4-Fluorophenyl)-2-methyl-4-(1-methyl-1-phenylethyl)-piperazine-1-carboxylic acid tert-butyl ester **4** (racemic)

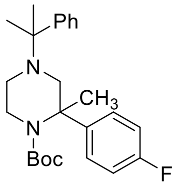

n-BuLi (0.25 mL, 0.35 mmol, 2.4 M in hexanes) was added to racemic piperazine **2c** (115 mg, 0.29 mmol) in dry THF (1.2 mL) at –78 °C. After 10 min, methyl iodide (0.10 mL, 1.45 mmol) was added. After 1 h, MeOH (1 mL) was added and the mixture was allowed to warm to room temperature. The solvent was evaporated and the mixture was purified by column chromatography on silica gel, eluting with hexane–EtOAc (4:1), to give the piperazine (±)-**4** (95 mg, 80%) as a colourless oil; R_f = 0.7 [hexane–EtOAc (7:3)]; FT-IR ν_{max} (ATR)/cm^{-1} 2975, 2935, 2877, 2816, 1687, 1602, 1231, 833; ^{1}H NMR (400 MHz, $CDCl_3$) δ 7.49 (d, 2H, *J* = 8 Hz), 7.32–7.29 (m, 4H), 7.24–7.21 (m, 1H), 7.00–6.95 (m, 2H), 3.90 (dt, 1H, *J* = 12 Hz, 4 Hz), 3.30 (td, 1H, *J* = 12 Hz, 4 Hz), 2.86 (dt, 1H, *J* = 12 Hz, 4 Hz), 2.50–2.39 (m, 2H), 2.30 (d , 1H, *J* = 12 Hz), 1.73 (s, 3H), 1.33 (s, 3H), 1.29 (s, 3H), 1.12 (s, 9H); ^{13}C NMR (100 MHz, $CDCl_3$) δ 161.1 (d, *J* = 242 Hz), 156.2, 148.5, 144.0 (d, *J* = 3 Hz), 127.9, 126.6 (d, *J* = 7 Hz), 126.4, 126.1, 114.3 (d, *J* = 21 Hz), 79.6, 61.7, 60.5, 59.3, 46.4, 43.3, 28.0, 24.0, 23.5, 20.1; ^{19}F NMR (377 MHz, $CDCl_3$) δ –118.13; HRMS (ESI-TOF) m/z: $[M+H]^+$ Calcd for $C_{25}H_{34}FN_2O_2$ is 413.2604. Found 413.2604. The enantiomers were resolved using HPLC (Amylose–2, n-hexane–isopropanol = 80:20, flow rate = 0.8 mL/min, l = 254 nm), t_R = 22.5 min and 24.7 min.

2-(4-Fluorophenyl)-2-methyl-4-(1-methyl-1-phenylethyl)-piperazine-1-carboxylic acid tert-butyl ester **4** (enantioenriched)

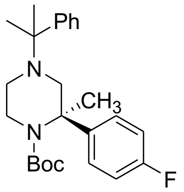

In the same way as racemic **4,** piperazine (*R*)-**2c** (80 mg, 0.20 mmol, er 98:2), n-BuLi (0.17 mL, 0.24 mmol, 2.4 M in hexanes) and MeI (0.07 mL, 1.0 mmol) gave, after purification on silica gel, eluting with hexane–EtOAc (4:1), the piperazine **4** (66 mg, 80%) as a colourless oil; data as above; er 96:4 by CSP-HPLC as described above (major component eluted at 22.8 min); $[\alpha]_D^{20}$ +24 (*c* 1.0, $CHCl_3$).

2-(4-Fluorophenyl)-2-methyl-piperazine-1-carboxylic acid tert-butyl ester **5** (racemic)

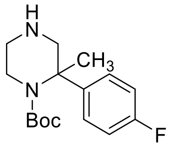

$Pd(OH)_2/C$ (20%) (8 mg), ammonium formate (48 mg, 0.75 mmol) and MeOH (10 mL) were added to piperazine **4** (racemic) (60 mg, 0.15 mmol) and the mixture was heated under reflux. After 2 h, the mixture was cooled to room temperature, filtered through celite, and evaporated. EtOAc (50 mL) was added and the mixture was washed with water (100 mL), brine (100 mL), dried ($MgSO_4$) and evaporated to give the piperazine **5** (42 mg, 98%) as a colourless oil; FT-IR ν_{max} (ATR)/cm^{-1} 2976, 2932, 2871, 1679, 1602, 1509, 1454, 1160, 857; ^{1}H NMR (400 MHz, $CDCl_3$) δ 7.32–7.30 (m, 2H), 7.02–6.98 (m, 2H), 3.84–3.78 (m, 1H), 3.36 (dt, 1H, *J* = 12 Hz, 4 Hz), 3.16–3.11 (m, 1H), 3.03–2.96 (m, 1H), 2.84 (d, 1H, *J* = 12 Hz), 2.74 (d, 1H, *J* = 12 Hz), 1.78 (s, 1H), 1.72 (s, 3H), 1.15 (s, 9H); ^{13}C NMR (100 MHz, $CDCl_3$) δ 162.2 (d, *J* = 243 Hz), 156.0, 143.3 (d, *J* = 17 Hz), 126.4 (d, *J* = 7 Hz), 114.7 (d, *J* = 21 Hz), 79.9, 59.7, 50.5, 45.2, 41.5, 28.0, 20.9; ^{19}F NMR (377 MHz, $CDCl_3$) δ –117.55; HRMS (ESI-TOF) m/z: $[M+H]^+$ Calcd for $C_{16}H_{24}FN_2O_2$ 295.1822. Found 295.1820. The enantiomers were resolved using HPLC (Cellulose–1, n-hexane–isopropanol = 85:15, flow rate = 0.8 mL/min, l = 254 nm), t_R = 7.0 min and 8.6 min.

2-(4-Fluorophenyl)-2-methyl-piperazine-1-carboxylic acid tert-butyl ester **5** (enantioenriched)

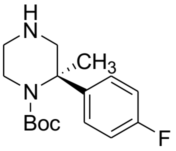

In the same way as racemic **5,** piperazine (*R*)-**4** (50 mg, 0.12 mmol, er 96:4) gave the piperazine **5** (35 mg, 98%) as a colourless oil; data as above; er 94:6 by CSP-HPLC as described above (major component eluted at 7.1 min); $[\alpha]_D^{20}$ +36 (*c* 1.0, $CHCl_3$).

2-(4-Chlorophenyl)-piperazine-1-carboxylic acid tert-butyl ester **6** (Racemic)

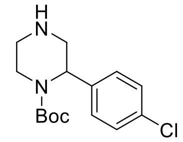

$Pd(OH)_2/C$ (20%) (20 mg), ammonium formate (114 mg, 1.8 mmol) and MeOH (20 mL) were added to piperazine **2f** (racemic) (150 mg, 0.36 mmol) and the mixture was heated under reflux. After 2 h, the mixture was cooled to room temperature, filtered through celite, and evaporated. The mixture was purified by column chromatography on silica gel, eluting with CH_2Cl_2–MeOH (9:1), to give the piperazine **6** (96 mg, 90%) as a colourless oil; R_f 0.8 [CH_2Cl_2–MeOH (4:1)], FT-IR ν_{max} (ATR)/cm^{-1} 2974, 2928, 1678, 1476, 1364, 861; ^{1}H NMR (400 MHz, $CDCl_3$) δ 7.39–7.35 (m, 2H), 7.29–7.24 (m, 2H), 5.20 (br s, 1H), 3.94 (br d, 1H, *J* = 12 Hz), 3.56 (br d, 1H, *J* = 12 Hz), 3.16 (dd, 1H, *J* = 12 Hz, 4 Hz), 3.02–2.95 (m, 2H), 2.84 (dd, 1H, *J* = 12 Hz, 4 Hz), 2.04 (br s, 1H), 1.47 (s, 9H); ^{13}C NMR (100 MHz, $CDCl_3$) δ 155.4, 139.4, 128.7, 126.8, 80.0, 53.4, 48.5, 45.7, 40.6, 28.4; HRMS (ESI-TOF) m/z: $[M+H]^+$ Calcd for $C_{15}H_{22}{}^{35}ClN_2O_2$ 297.1370. Found 297.1386. The enantiomers were resolved using HPLC (Amylose–2, n-hexane–isopropanol = 85:15, flow rate = 1.0 mL/min, l = 254 nm), t_R = 16.2 min and 20.6 min.

2-(4-Chlorophenyl)-piperazine-1-carboxylic acid tert-butyl ester **6** (enantioenriched)

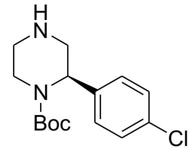

In the same way as racemic **6,** piperazine (*R*)-**2f** (50 mg, 0.12 mmol, er 99:1) gave the piperazine **6** (33 mg, 94%) as a colourless oil; data as above; er 99:1 by CSP-HPLC as described above (major component eluted at 16.4 min); $[\alpha]_D^{20}$ –42 (*c* 1.0, $CHCl_3$).

3-(4-Chlorophenyl)-1-(1-methyl-1-phenylethyl)-piperazine **7** (racemic)

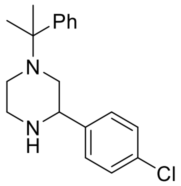

A solution of HCl in dioxane (2 mL, 4 M) was added to racemic piperazine **2f** (50 mg, 0.12 mmol) in dry MeOH (2 mL). After 18 h, the solvent was evaporated. Water (50 mL) and saturated aqueous $NaHCO_3$ solution (30 mL) were added, and the mixture was extracted with EtOAc (3 x 20 mL). The organic layers were washed with brine (50 mL), dried ($MgSO_4$) and evaporated to give the piperazine **7** (37 mg, 97%) as a colourless oil; FT-IR ν_{max} (ATR)/cm^{-1} 2972, 2818, 1601, 1490, 1090, 765; ^{1}H NMR (400 MHz, $CDCl_3$) δ 7.57 (d, 2H, *J* = 8 Hz), 7.35–7.29 (m, 5H), 7.27–7.21 (m, 2H), 3.83 (dd, 1H, *J* = 12 Hz, 4Hz), 3.10–2.97 (m, 2H), 2.83–2.74 (m, 2H), 2.34 (td, 1H, *J* = 12 Hz, 4Hz), 2.18-2.13 (m, 1H), 1.81 (br s, 1H), 1.38 (s, 3H), 1.36 (s, 3H); ^{13}C NMR (100 MHz, $CDCl_3$) δ 149.0, 141.6, 132.9, 128.4, 128.4, 128.0, 126.2, 126.1, 60.6, 59.8, 54.5, 46.9, 46.2, 24.4, 23.8; HRMS (ESI-TOF) m/z: [M+H]$^+$ Calcd for $C_{19}H_{24}{}^{35}ClN_2$ 315.1628. Found 315.1624. The enantiomers were resolved using HPLC (Amylose–2, n-hexane–isopropanol = 85:15, flow rate = 1.0 mL/min, l = 254 nm), t_R = 16.2 min and 20.6 min.

3-(4-Chlorophenyl)-1-(1-methyl-1-phenylethyl)-piperazine **7** (enantioenriched)

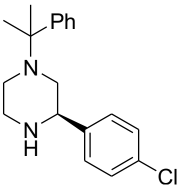

In the same way as racemic **7,** piperazine (*R*)-**2f** (80 mg, 0.19 mmol, er 99:1) gave the piperazine **7** (58 mg, 97%) as a colourless oil; data as above; er 99:1 by CSP-HPLC as described above (major component eluted at 16.4 min); $[\alpha]_D^{20}$ –54 (*c* 1.0, $CHCl_3$).

2.7 Synthesis of GSK-3β inhibitor

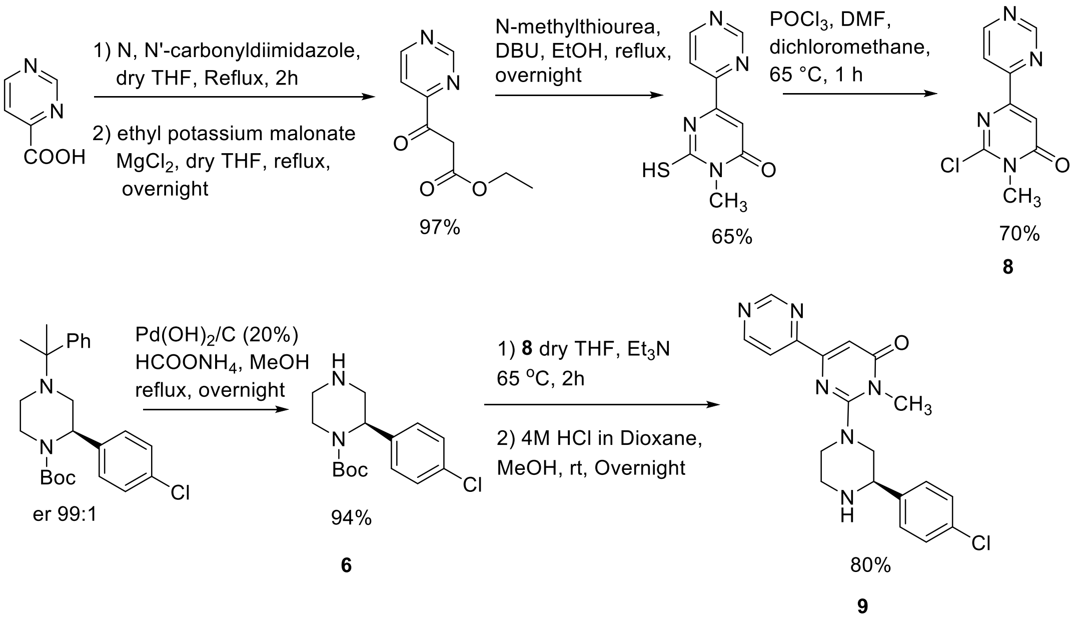

The pyrimidine **8** was prepared according to the literature (see scheme above).[7]

*Piperazine **9***

Pyrimidine **8** (112 mg, 0.50 mmol) in dry THF (5 mL) was added to piperazine **6** (150 mg, 0.50 mmol) and Et_3N (0.22 mL, 1.5 mmol) in dry THF (20 mL) and the mixture as heated under reflux. After 2 h, the solvent was evaporated. EtOAc (50 mL) was added, and the mixture was washed with water (100 mL), brine (100 mL), dried ($MgSO_4$) and evaporated. Purification by column chromatography on silica gel, eluting with hexane–EtOAc (2:3) gave the *N*-Boc-piperazine (207 mg, 85%) as a white amorphous solid; m.p. 62–64 °C; R_f = 0.5 [hexane–EtOAc (3:2)]; ^{1}H NMR (400 MHz, $CDCl_3$) δ 9.27 (d, 1H, *J* = 1 Hz), 8.88 (d, 1H, *J* = 5 Hz), 8.16 (dd, 1H, *J* = 5, 1 Hz), 7.36–7.30 (m, 2H), 7.26–7.19 (m, 3H), 5.45 (br d, 1H), 4.20 (br d, 1H, *J* = 12 Hz), 4.04 (br d, 1H, *J* = 12 Hz), 3.61–3.57 (m, 2H), 3.46 (td, 1H, *J* = 12 Hz, 4 Hz), 3.33 (s, 3H), 3.12 (td, 1H, *J* = 12 Hz, 4 Hz), 1.46 (s, 9H); ^{13}C NMR (100 MHz, $CDCl_3$) δ 164.7, 160.8, 159.4, 158.7, 158.3, 155.5, 154.9, 139.4, 128.6, 127.3, 126.4, 117.8, 106.2, 80.7, 53.9, 51.7, 49.6, 39.5, 32.8, 28.3.

To this *N*-Boc-piperazine (125 mg, 0.26 mmol) in MeOH (2 mL) was added slowly a solution of HCl in dioxane (2 mL, 4 M) at room temperature. After 18 h, the solvent was evaporated. Water (50 mL) and saturated aqueous $NaHCO_3$ (30 mL) was added. The mixture was extracted with EtOAc (3 x 50 mL) and evaporated to give the piperazine **9** (93 mg, 94%) as a white amorphous solid; m.p. 56–58 °C; $[\alpha]_D^{20}$ –42 (*c* 1.0, $CHCl_3$); ^{1}H NMR (400 MHz, $CDCl_3$) δ 9.27 (d, 1H, *J* = 1 Hz), 8.86 (d, 1H, *J* = 5 Hz), 8.17 (dd, 1H, *J* = 5, 1 Hz), 7.48–7.46 (m, 2H), 7.42–7.32 (m, 3H), 4.05 (dd, 1H, *J* = 12 Hz, 4 Hz), 3.68–3.63 (m, 2H), 3.58 (s, 3H), 3.29–3.20 (m, 3H), 3.09–3.03 (m, 1H), 2.08 (br s, 1H); ^{13}C NMR (100 MHz, $CDCl_3$) δ 165.1, 161.1, 159.2, 158.6, 158.2, 155.6, 141.1, 128.6, 128.1, 127.0, 117.9, 105.7, 60.0, 56.5, 49.6, 45.7, 33.3.

3. VT-NMR spectra

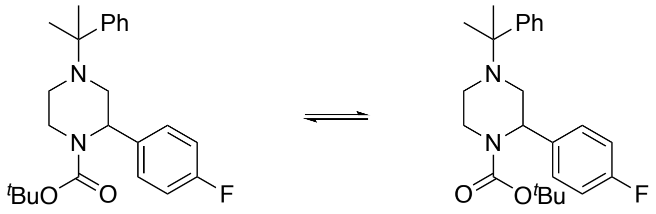

2c, rotamer a   **2c**, rotamer b

Coalescence of signals in the ^{1}H NMR spectrum of carbamate **2c** in D_8-THF was followed by taking spectra at various temperatures. The ratio of the rotamers is ~ 1:1. The ^{1}H NMR spectra in the region 5.40–3.10 ppm are shown below. The peak at 3.57 ppm is undeuterated THF.

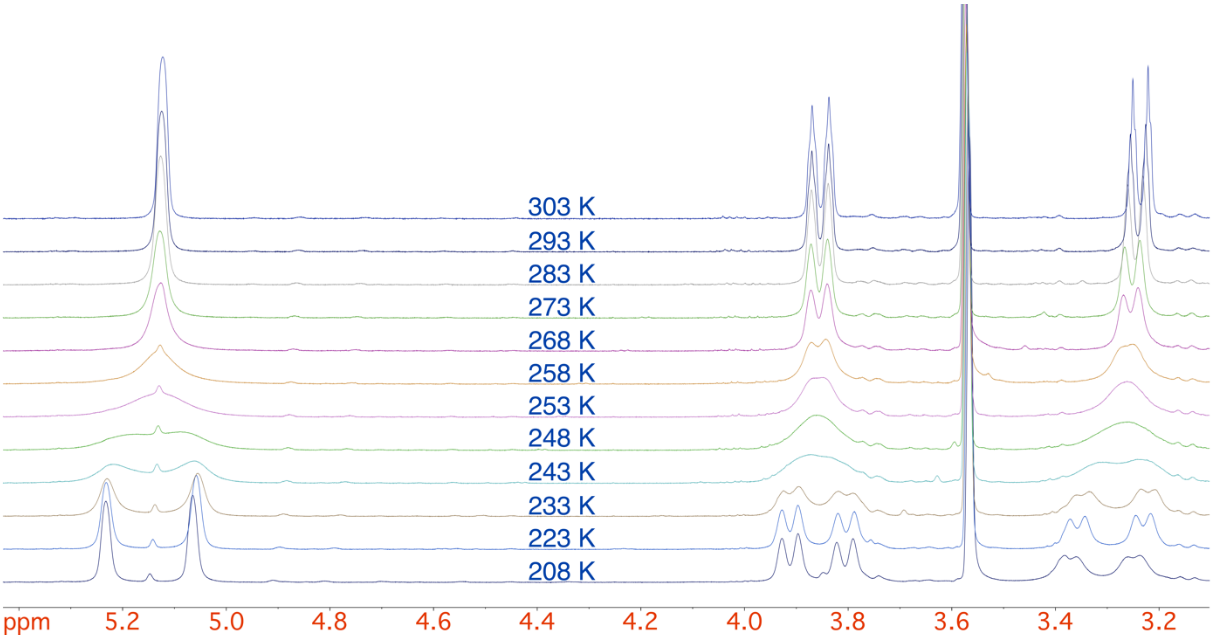

Assuming an equal rate between rotamers, the following rate constants can be estimated using line shape analysis (DNMR with the software iNMR)[8]:

Table S-3. Estimated rate constants from VT-NMR spectroscopy of carbamate **2c**.

T/K	1/T	*k*	ln(*k*/T)
208	0.004808	1	-5.33754
223	0.004484	16	-2.63458
233	0.00429	35	-1.89569
243	0.00411	80	-1.11103
248	0.00403	100	-0.90826
253	0.00395	180	-0.34043
258	0.00388	300	0.15082
268	0.00373	700	0.96009
273	0.00366	900	1.19292
283	0.00353	1700	1.79294
293	0.00341	3000	2.32619
303	0.0033	7000	3.13993

Eyring plot:

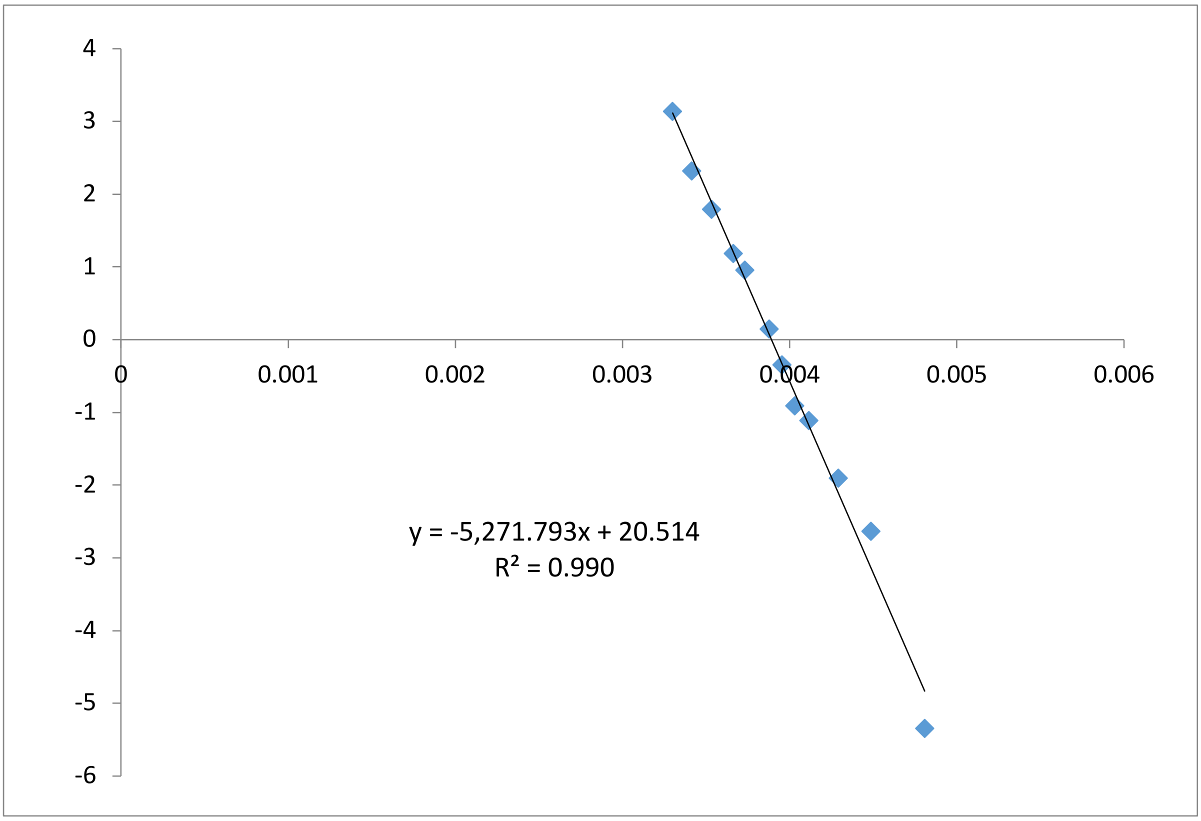

By using the Eyring equation, this gave the following data:

slope –5272, intercept 20.5

Approximate activation parameters for Boc rotation in THF:

$\Delta H^{\ddagger} \approx 43.8$ kJ/mol

$\Delta S^{\ddagger} \approx -27.0$ J/mol·K

Hence the barrier to rotation $\Delta G^{\ddagger} \approx 49.1$ kJ/mol at –78 °C.

The half-life for rotation is about 2.4 sec at –78 °C.

These data are very approximate as small variations in the rate contants affect the values significantly, although the overall barrier is in line with expectations and with the DFT calculations (see Section 6, page S-67).

4. NMR spectra

^{1}H NMR spectrum ($CDCl_3$, 400 MHz) of **b:**

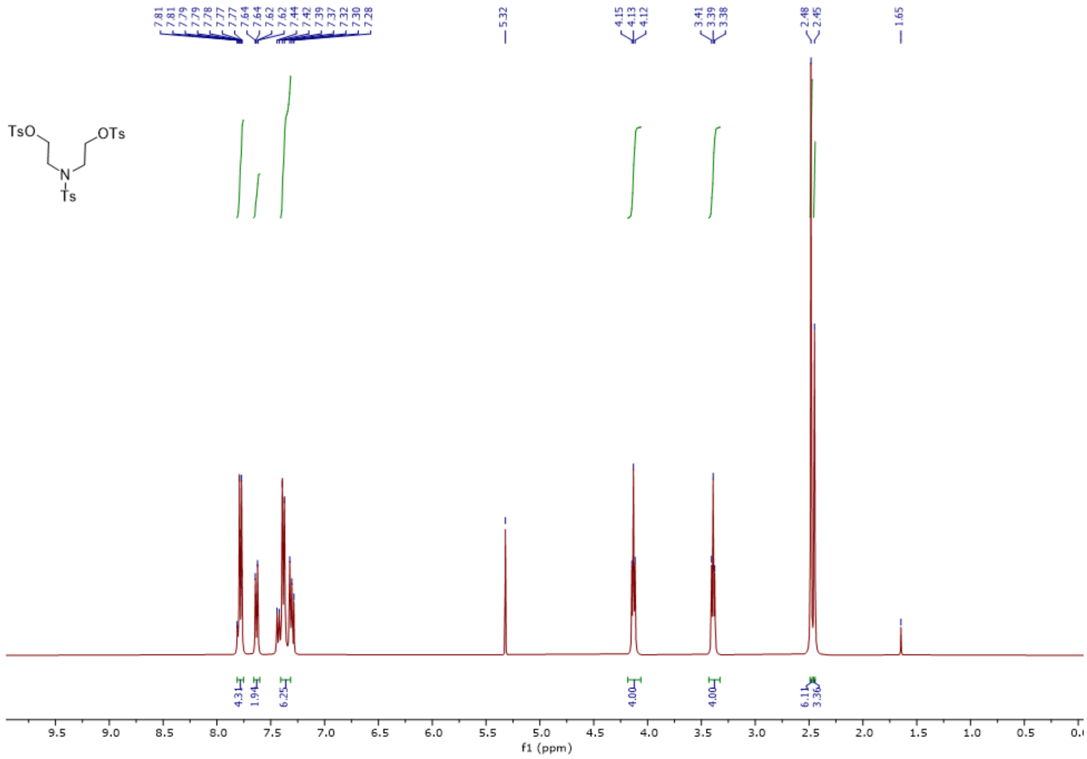

^{1}H NMR spectrum ($CDCl_3$, 400 MHz) of **c:**

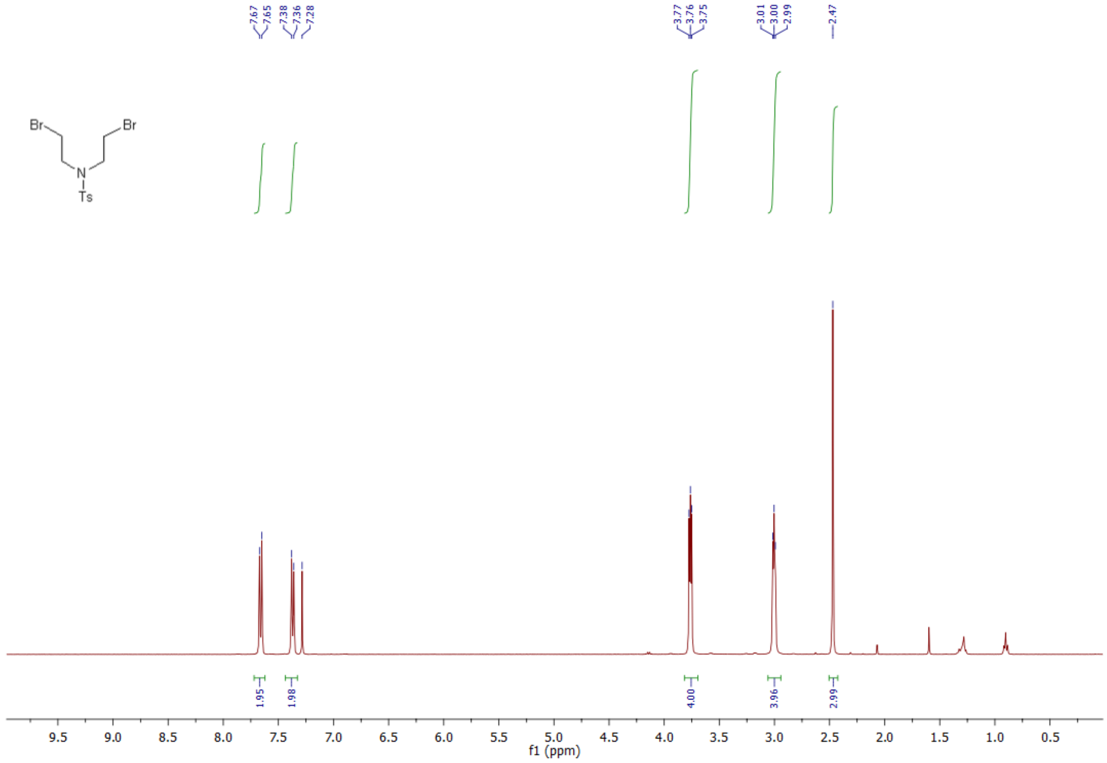

^{1}H NMR spectrum ($CDCl_3$, 400 MHz) of **d:**

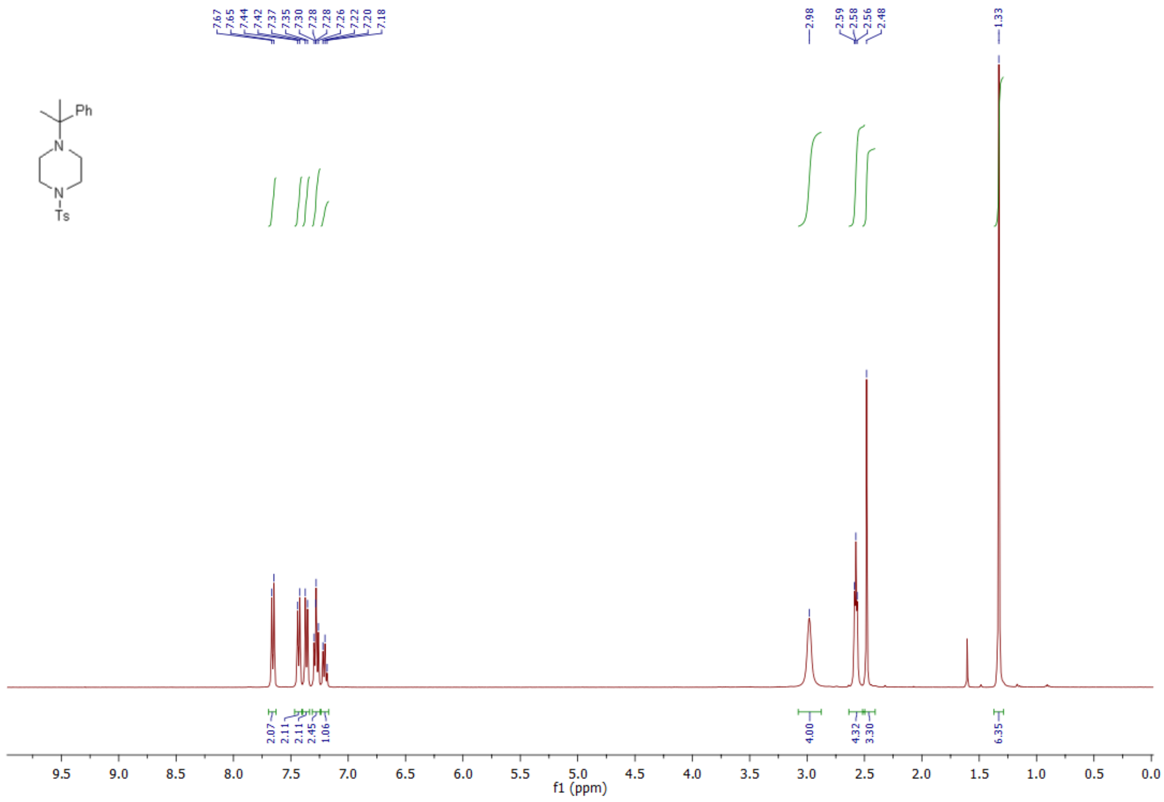

^{1}H NMR spectrum ($CDCl_3$, 400 MHz) of **e:**

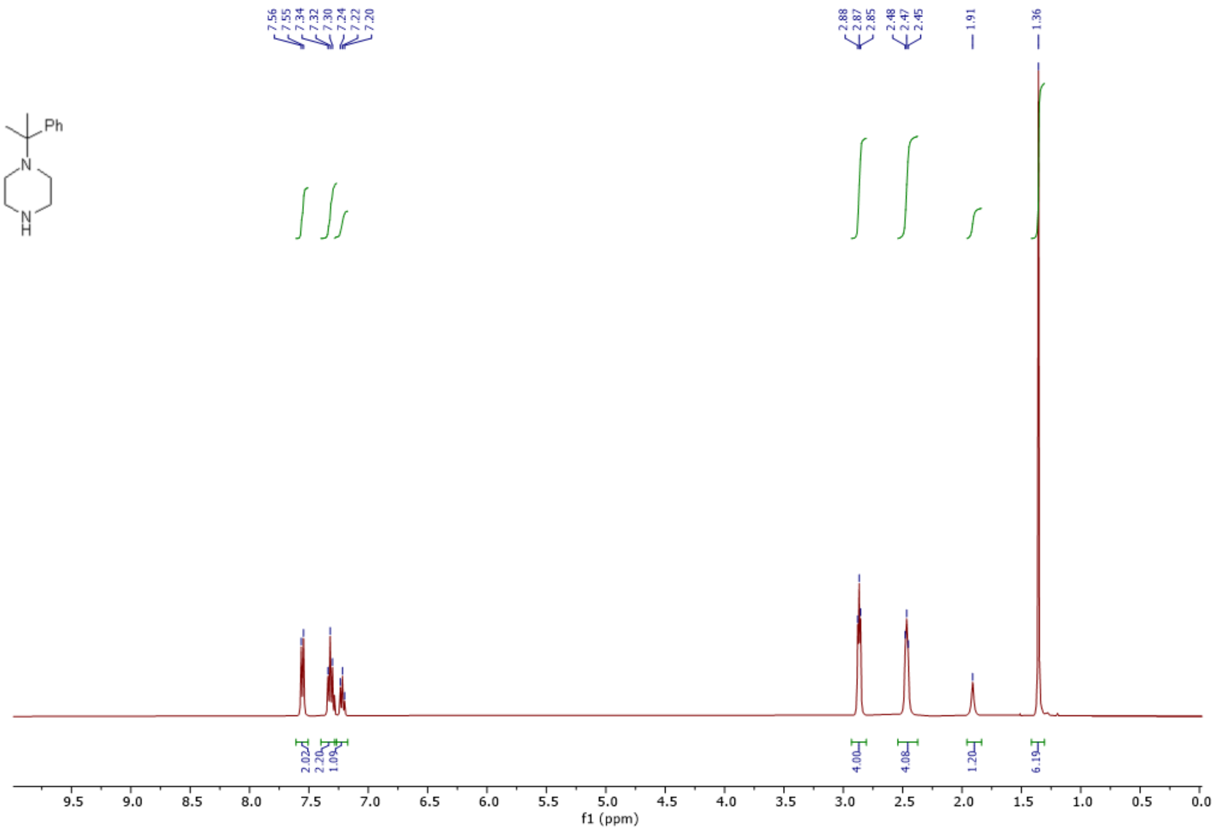

^{1}H NMR spectrum ($CDCl_3$, 400 MHz) of **f:**

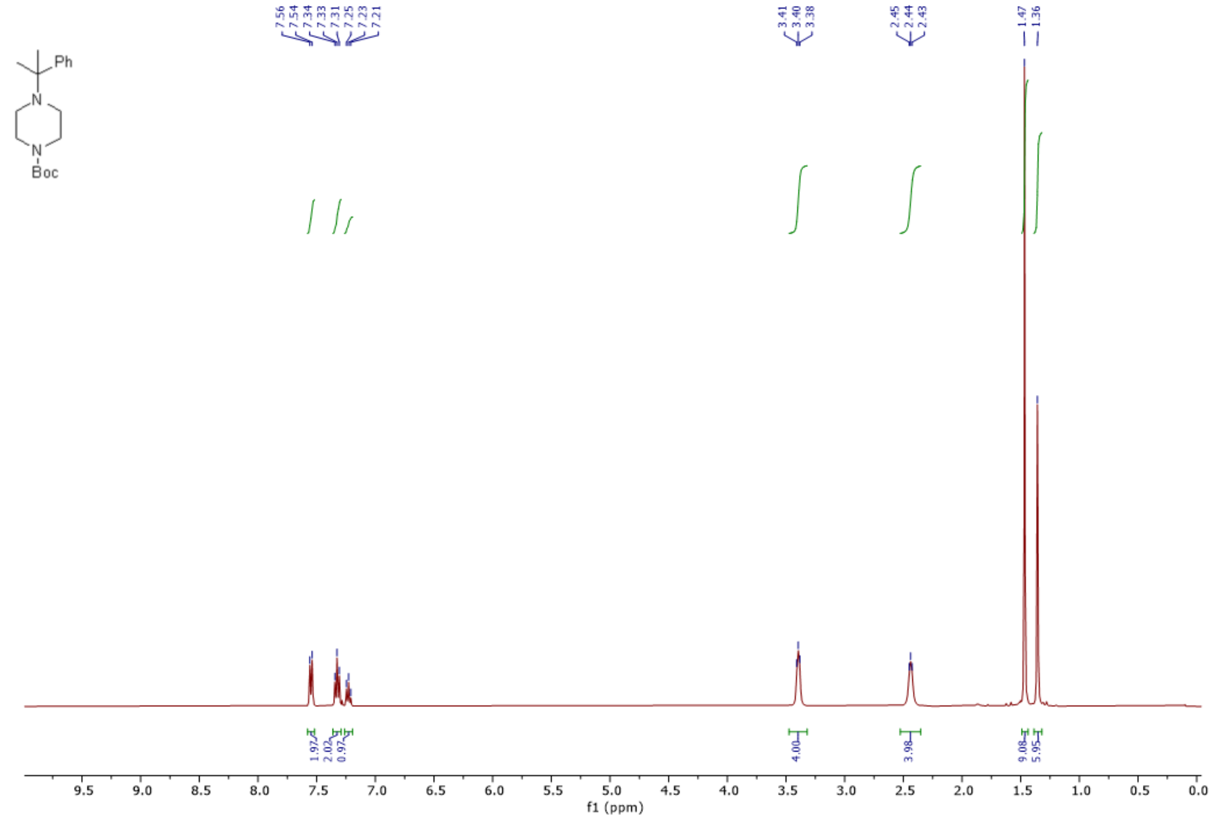

^{13}C {^{1}H} NMR spectrum ($CDCl_3$, 100 MHz) of **f:**

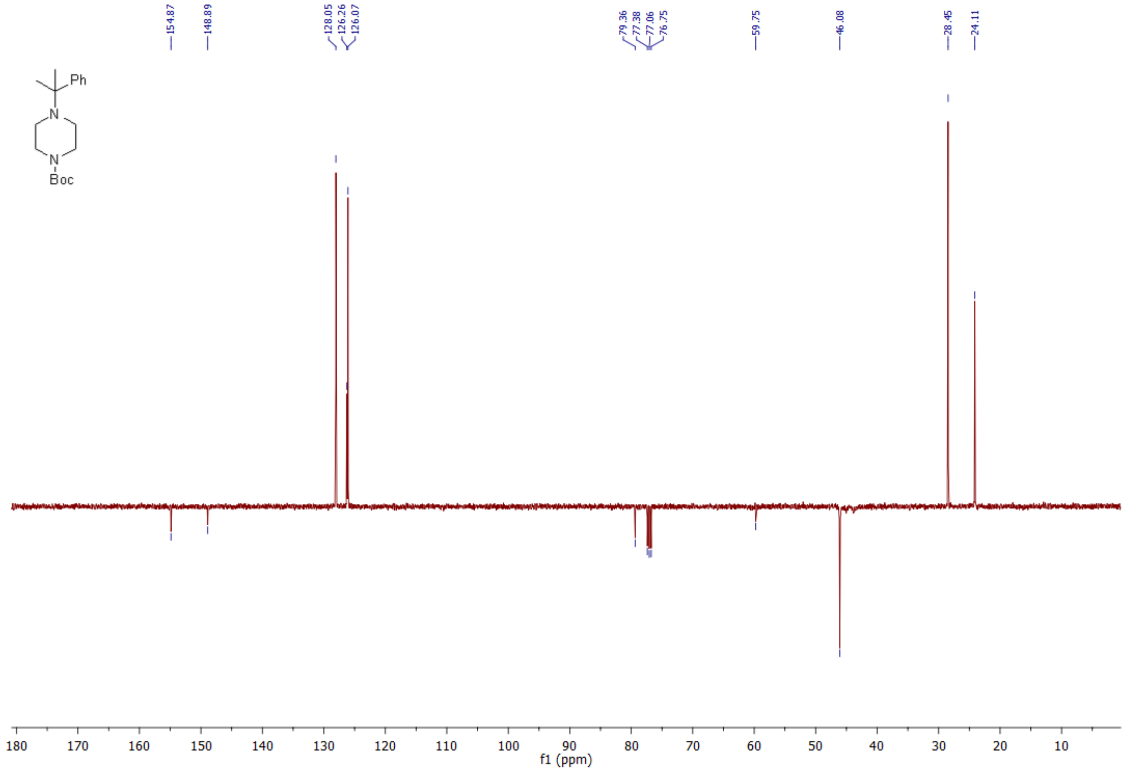

^{1}H NMR spectrum ($CDCl_3$, 400 MHz) of **1:**

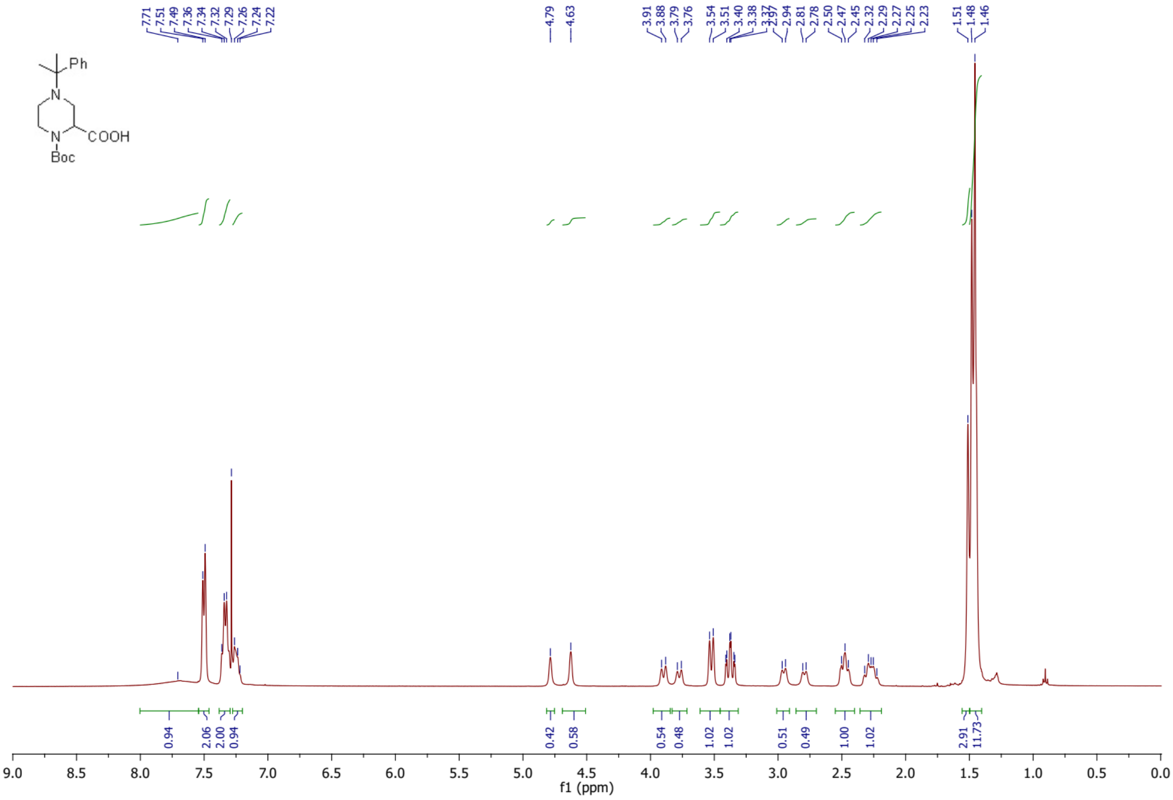

^{13}C {^{1}H} NMR spectrum ($CDCl_3$, 100 MHz) of **1:**

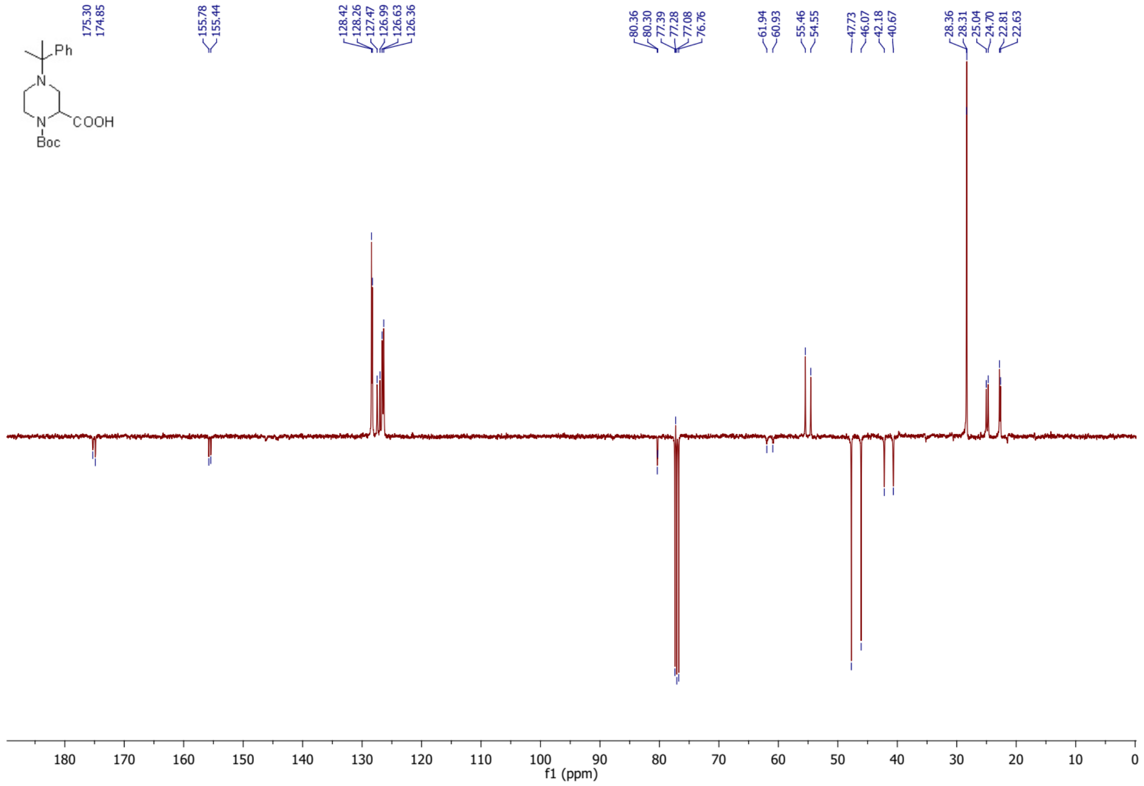

^{1}H NMR spectrum ($CDCl_3$, 400 MHz) of **A:**

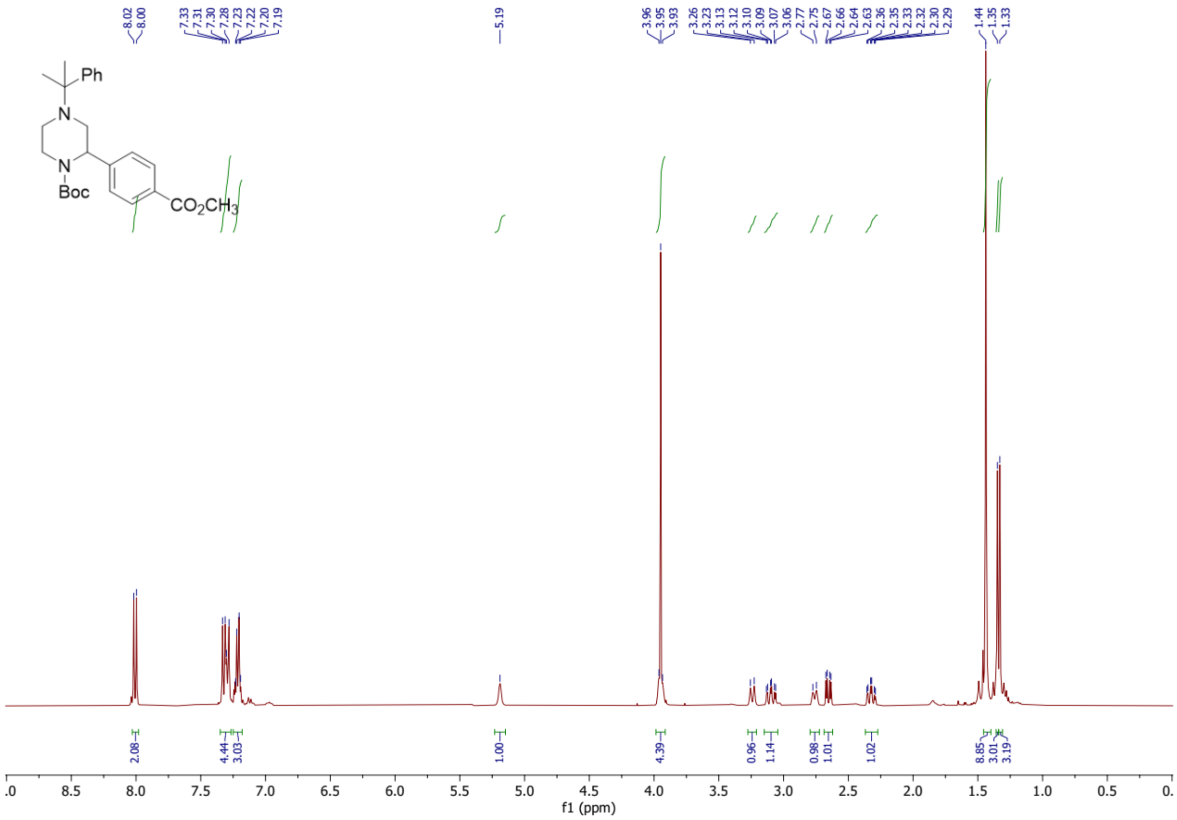

^{13}C {^{1}H} NMR spectrum ($CDCl_3$, 100 MHz) of **A:**

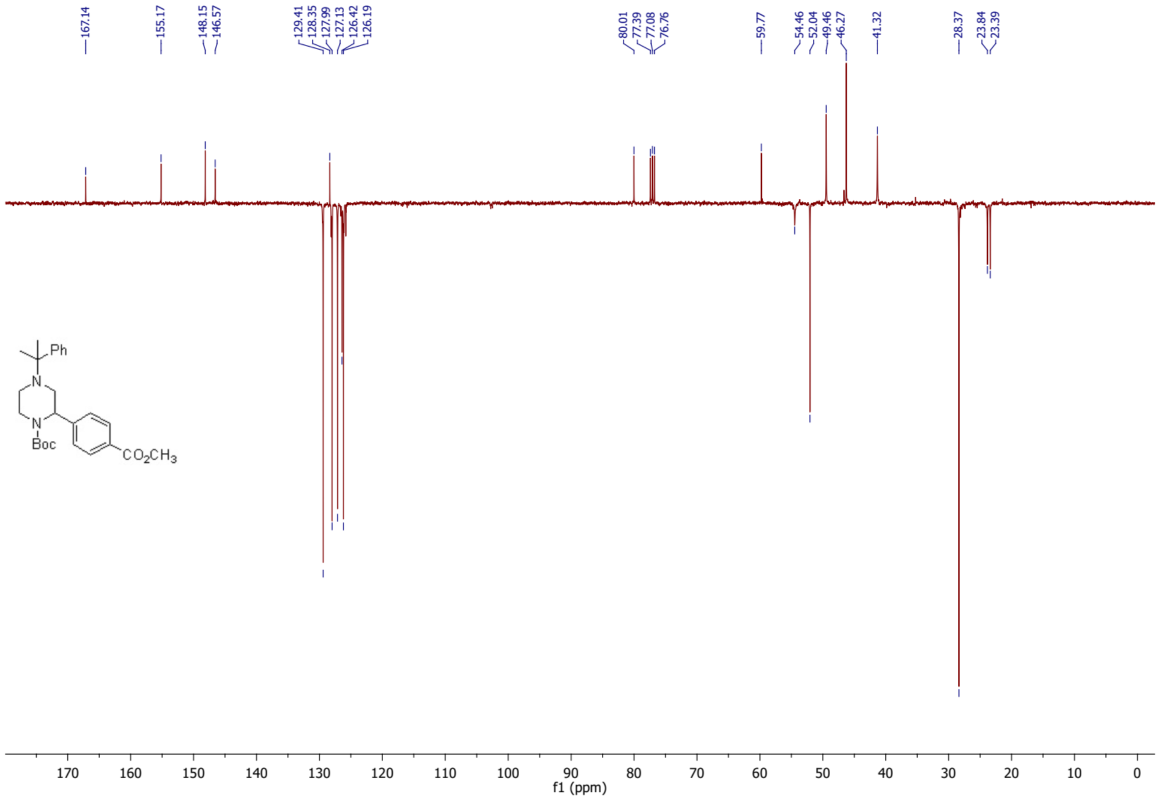

^{1}H NMR spectrum ($CDCl_3$, 400 MHz) of **2a:**

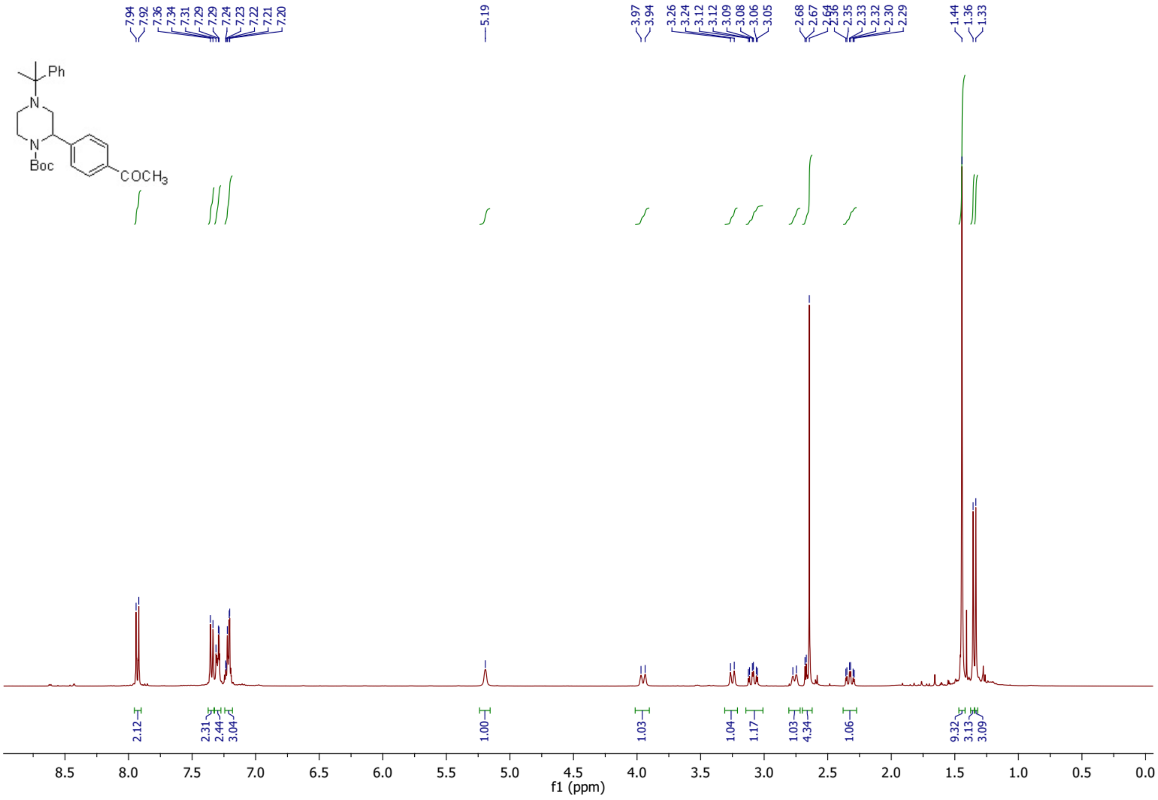

^{13}C {^{1}H} NMR spectrum ($CDCl_3$, 100 MHz) of **2a:**

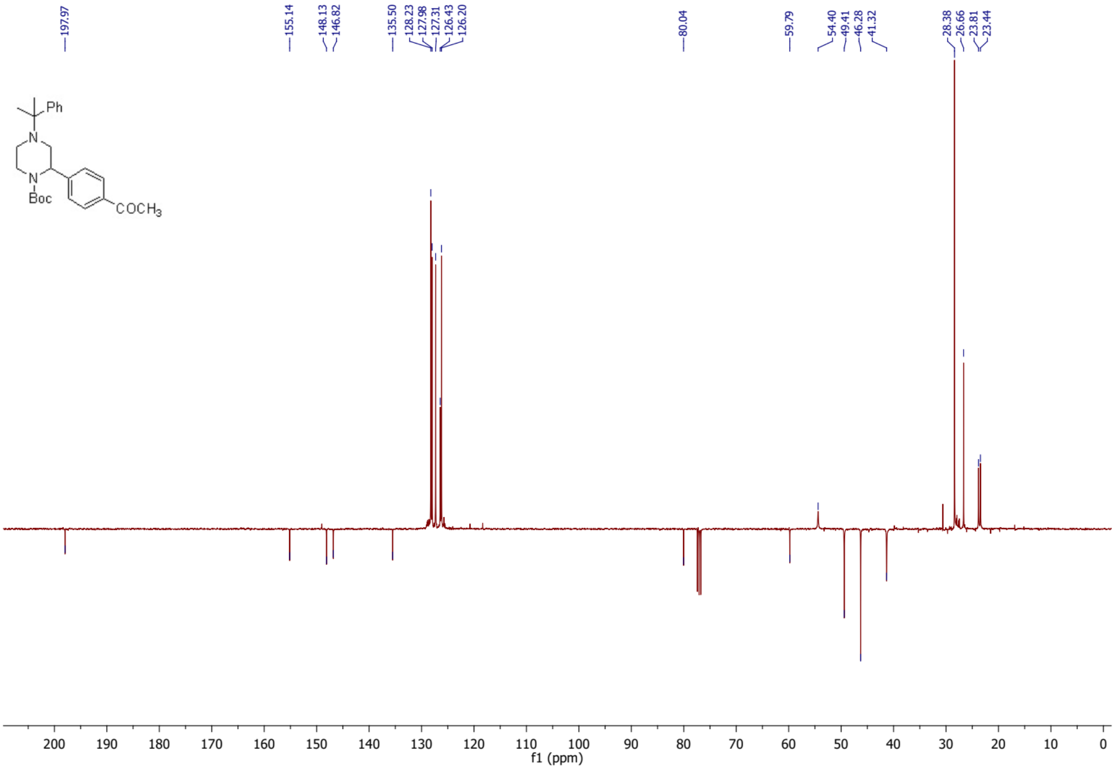

^{1}H NMR spectrum ($CDCl_3$, 400 MHz) of **2b:**

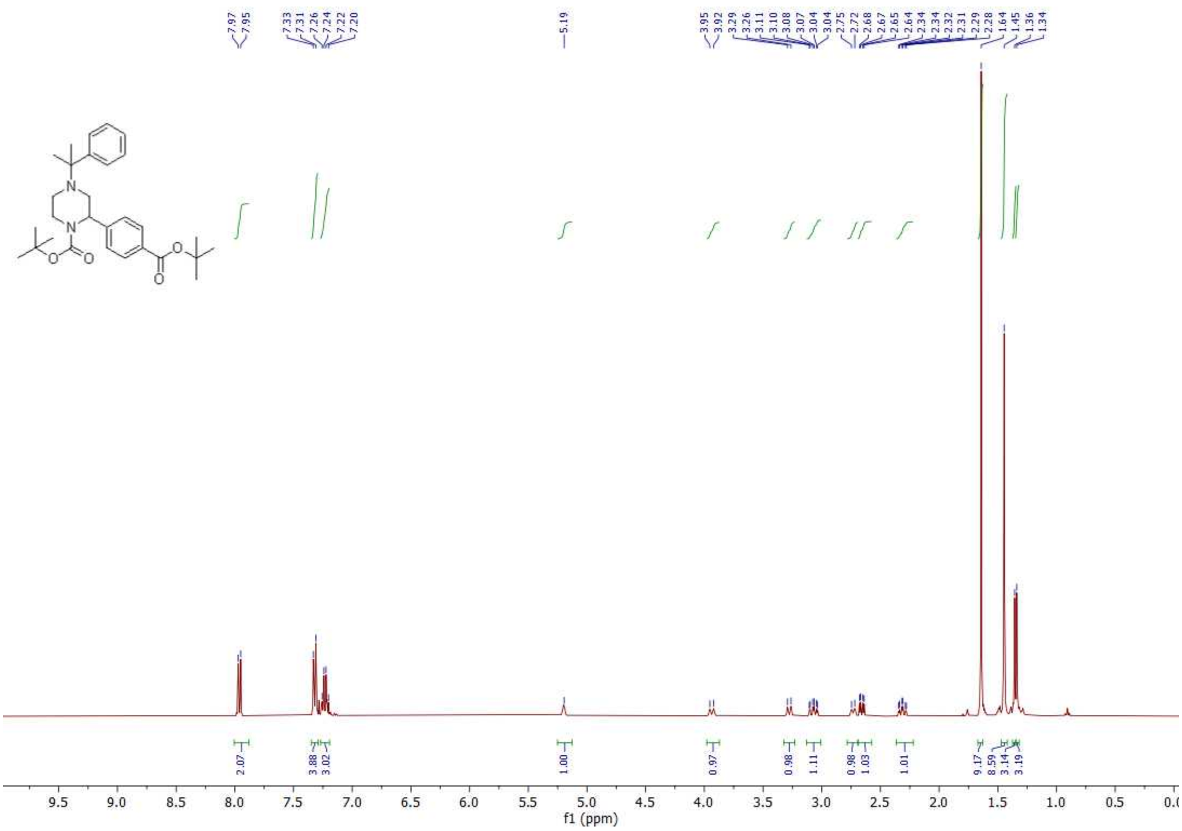

^{13}C {^{1}H} NMR spectrum ($CDCl_3$, 100 MHz) of **2b:**

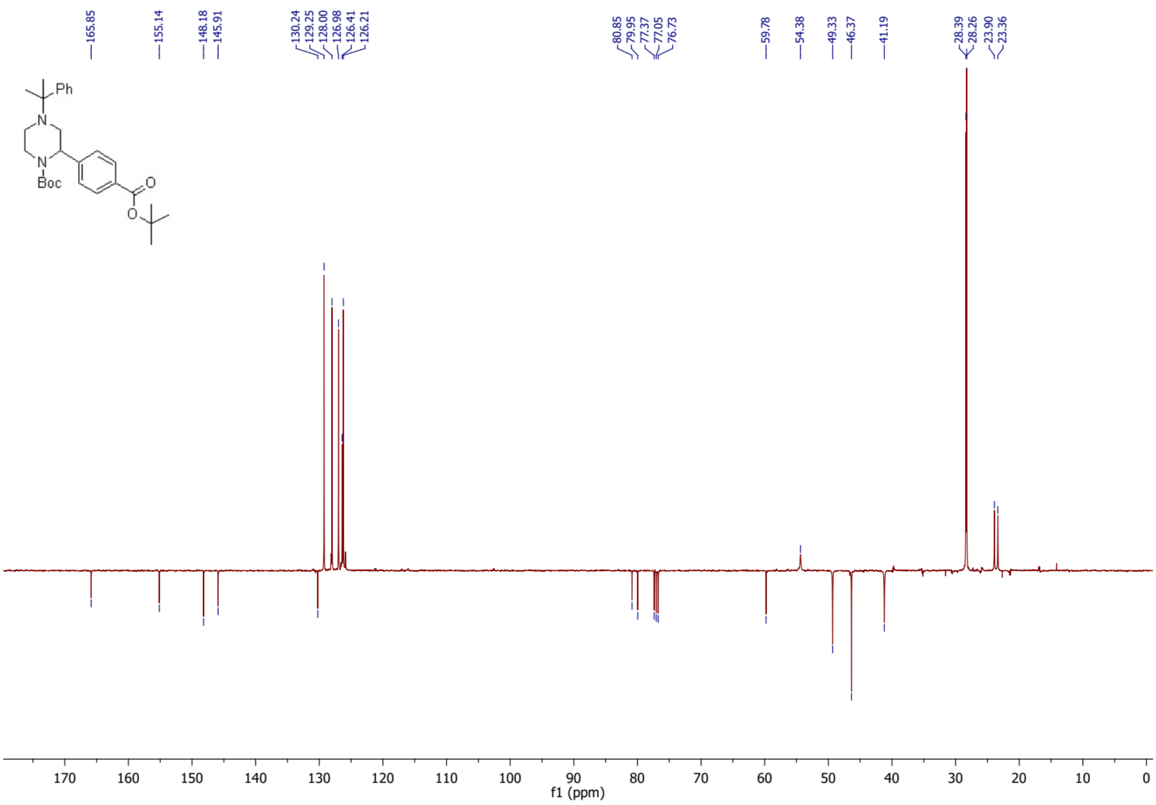

^{1}H NMR spectrum ($CDCl_3$, 400 MHz) of **2c:**

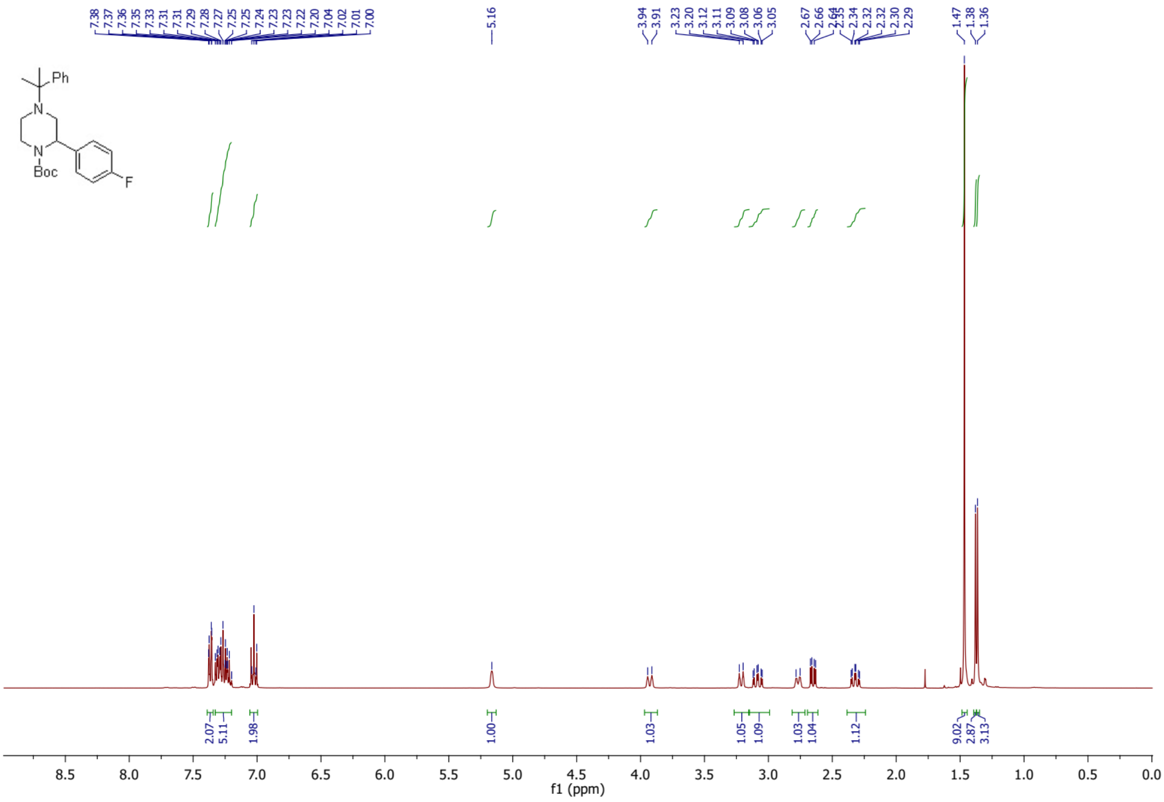

^{13}C {^{1}H} NMR spectrum ($CDCl_3$, 100 MHz) of **2c:**

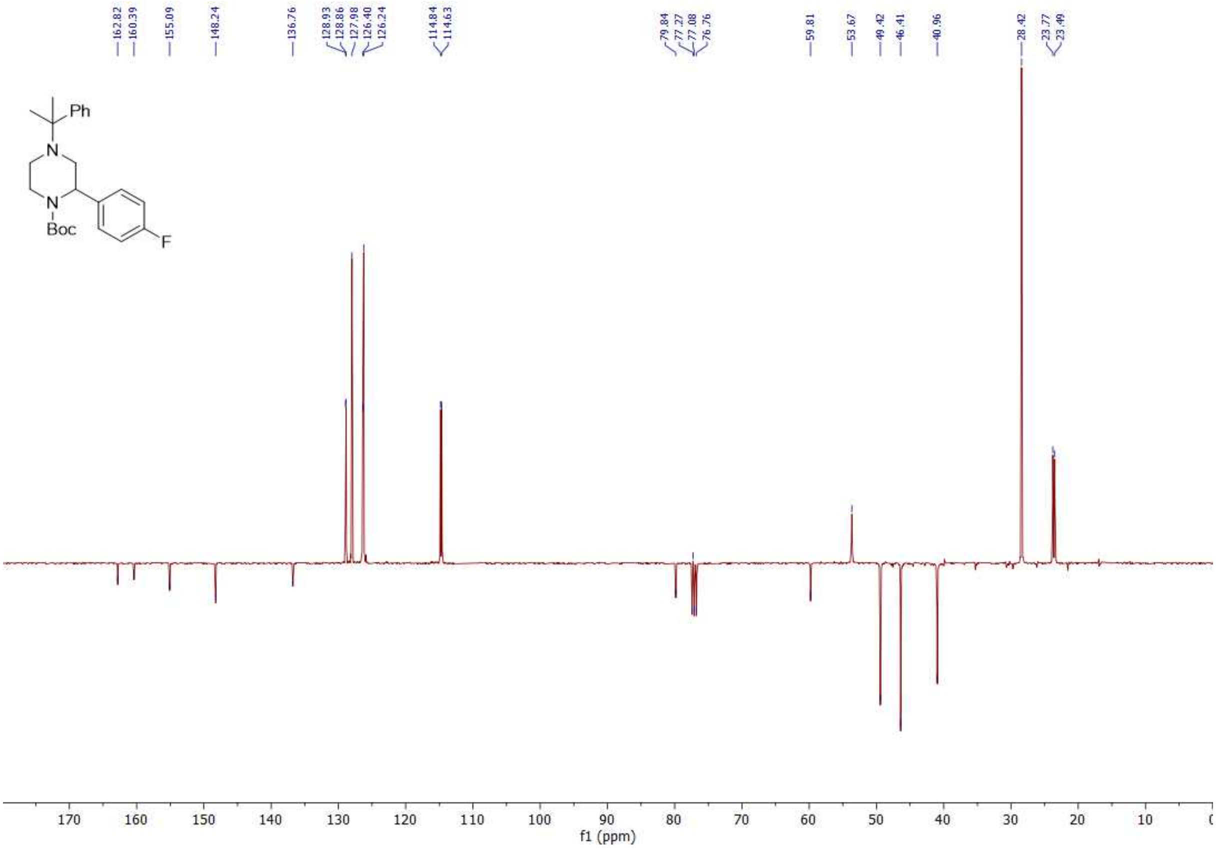

^{19}F NMR spectrum ($CDCl_3$, 377 MHz) of **2c**:

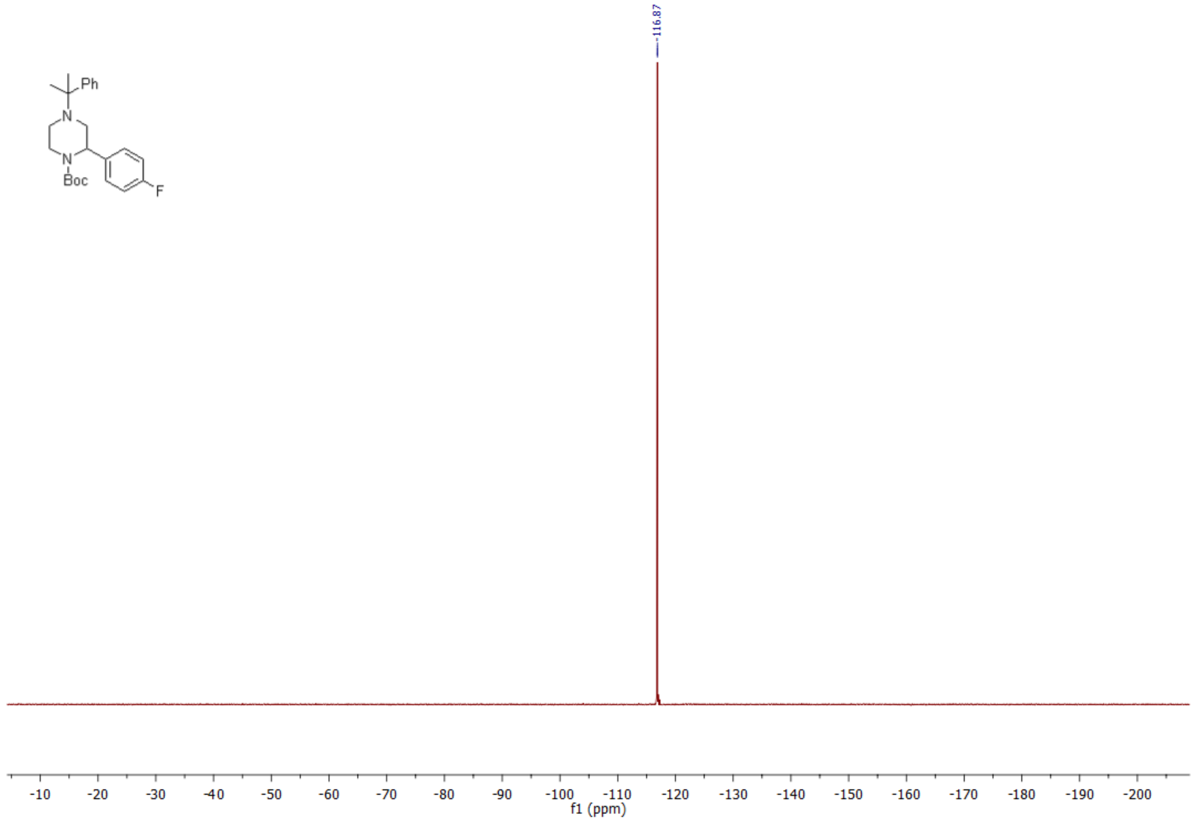

^{1}H NMR spectrum ($CDCl_3$, 400 MHz) of **2d:**

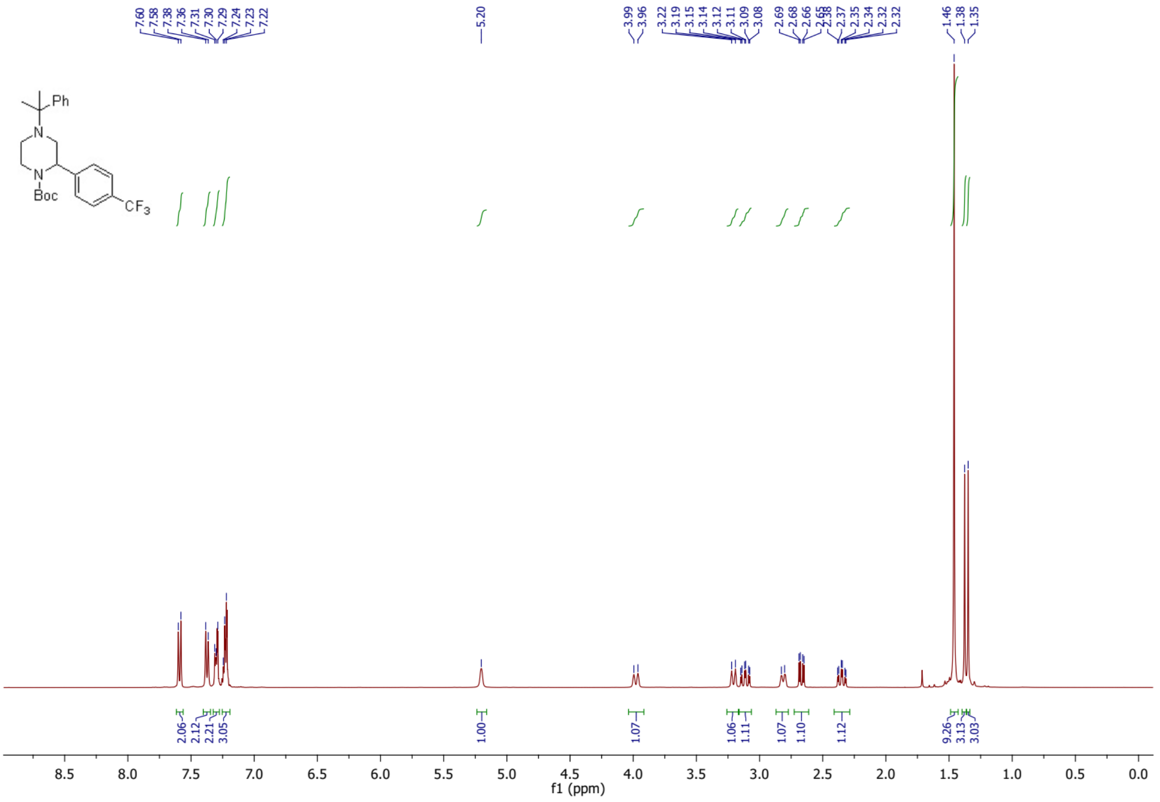

^{13}C {^{1}H} NMR spectrum ($CDCl_3$, 100 MHz) of **2d:**

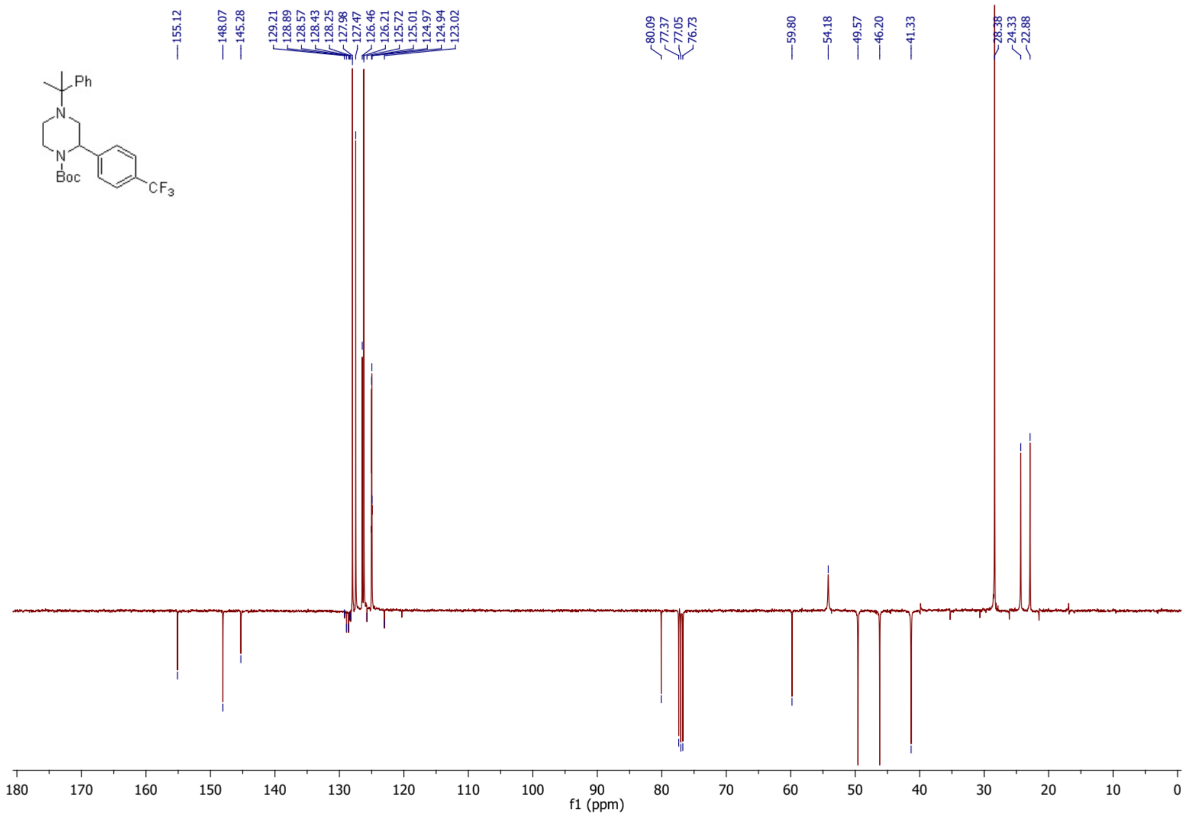

^{19}F NMR spectrum ($CDCl_3$, 377 MHz) of **2d**:

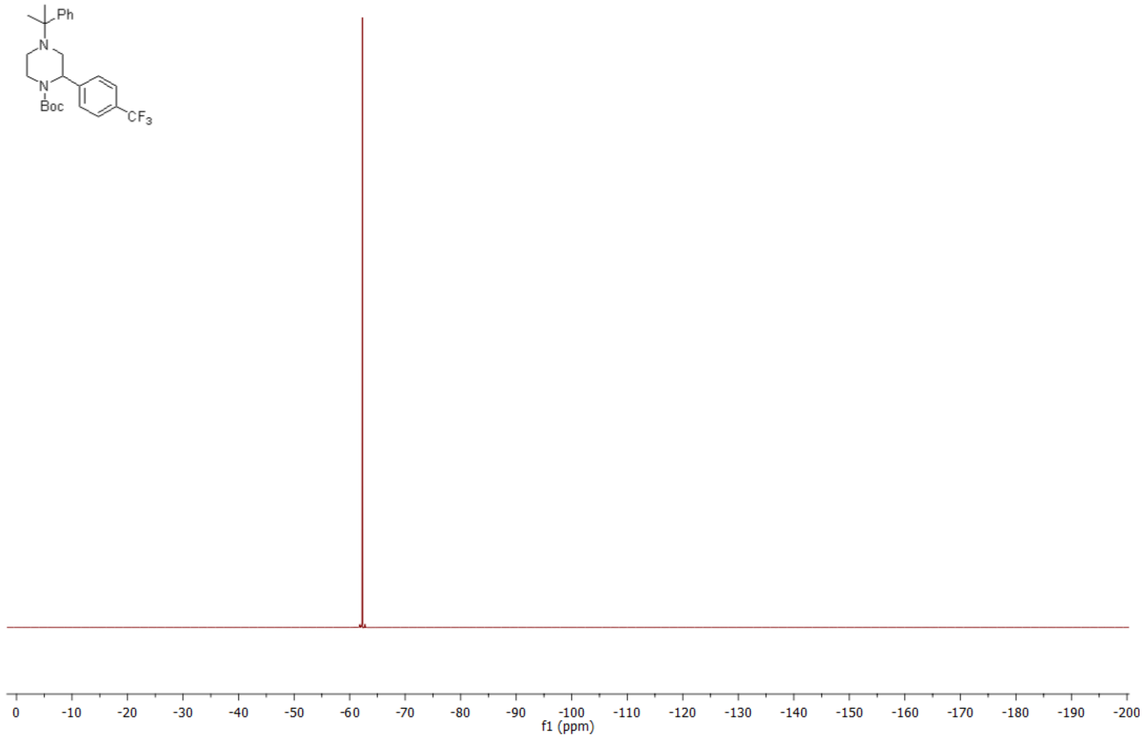

^{1}H NMR spectrum ($CDCl_3$, 400 MHz) of **2e:**

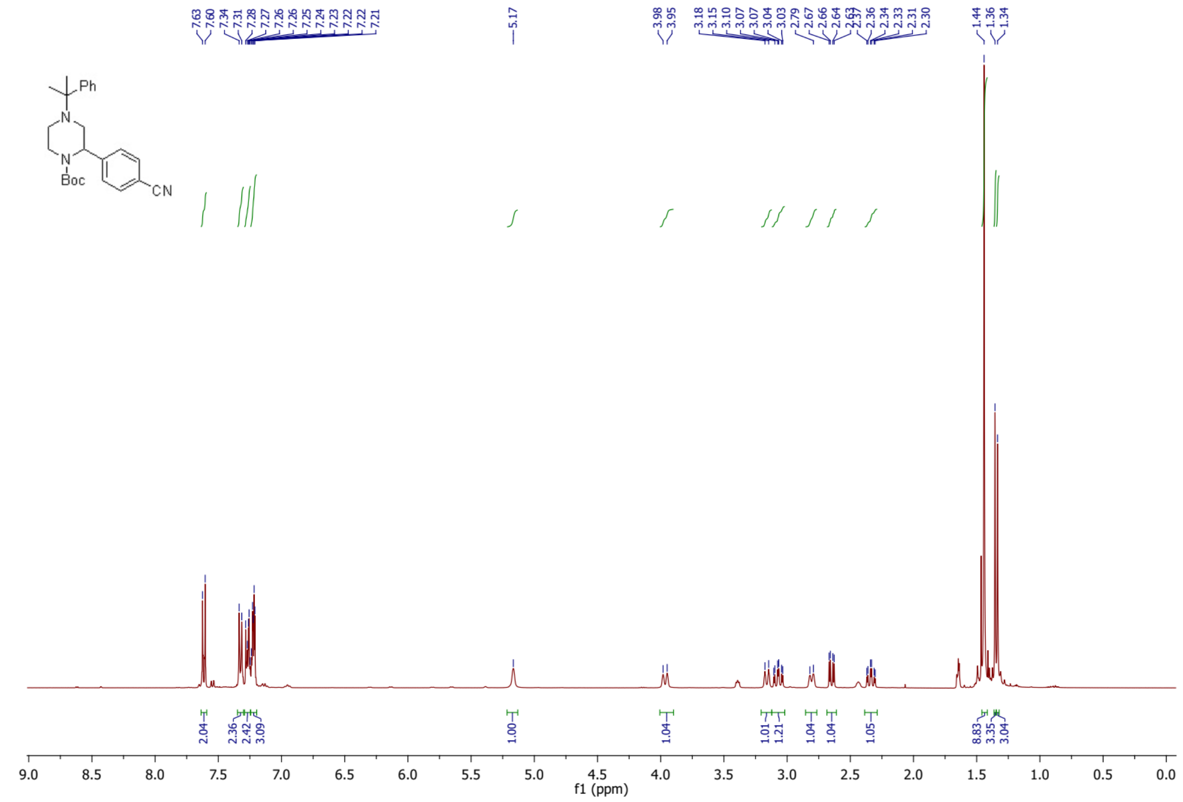

^{13}C {^{1}H} NMR spectrum ($CDCl_3$, 100 MHz) of **2e:**

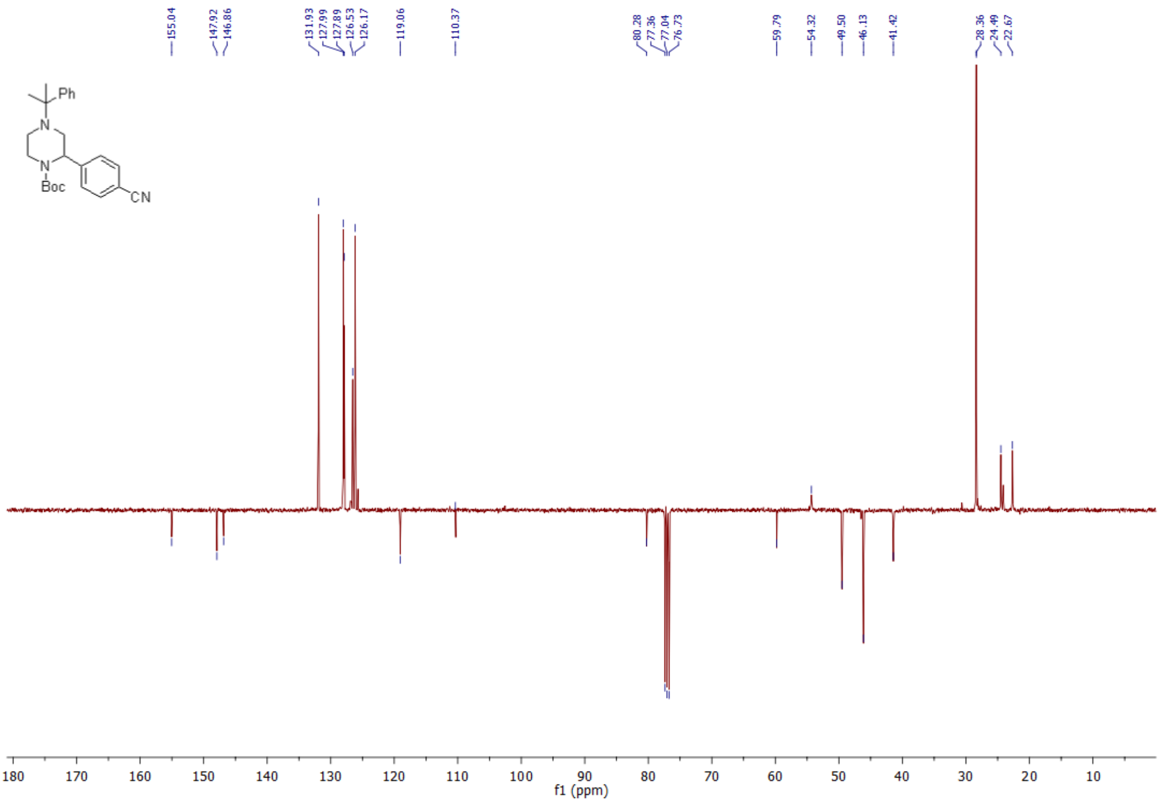

^{1}H NMR spectrum ($CDCl_3$, 400 MHz) of **2f:**

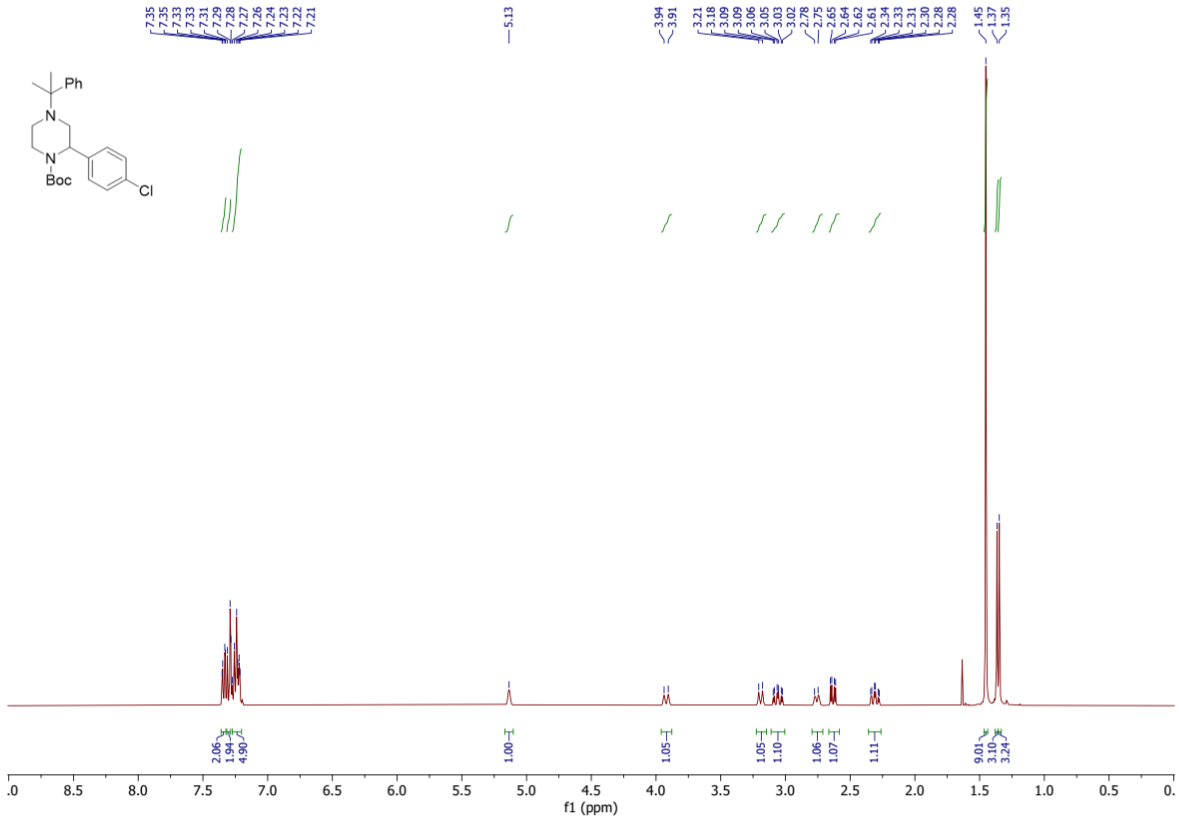

^{13}C {^{1}H} NMR spectrum ($CDCl_3$, 100 MHz) of **2f:**

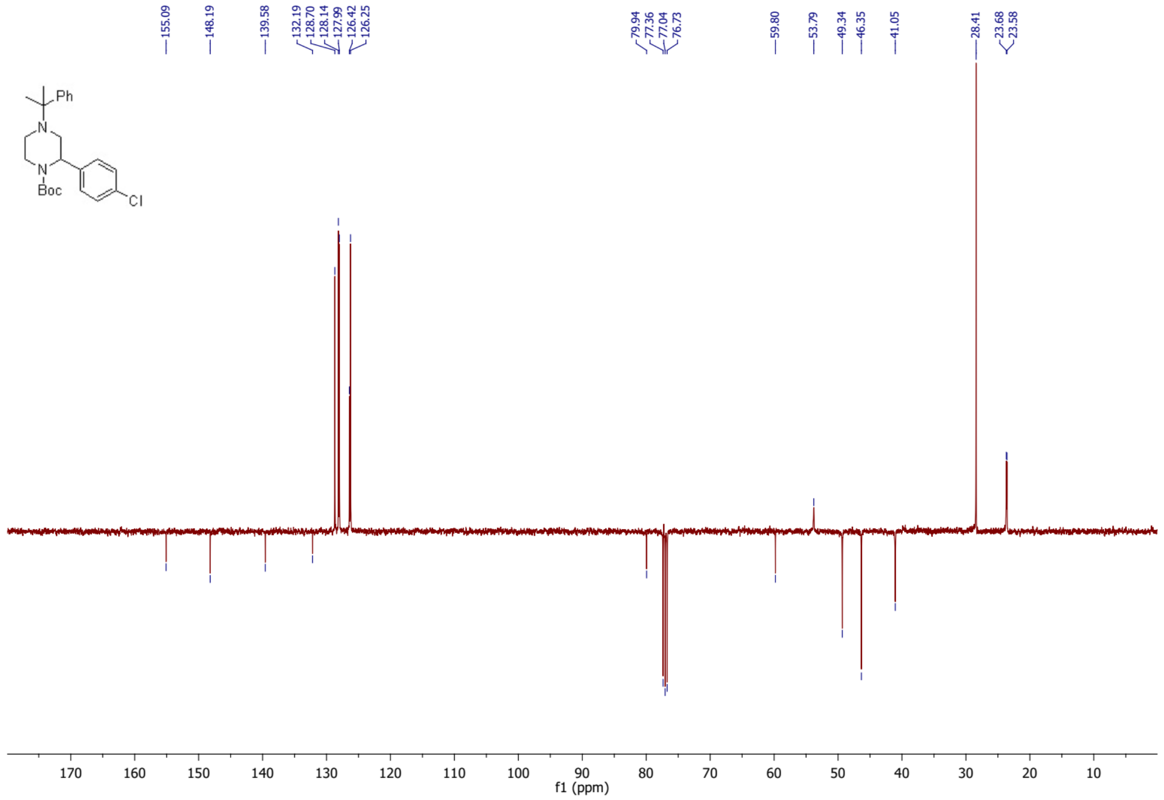

^{1}H NMR spectrum ($CDCl_3$, 400 MHz) of **2g:**

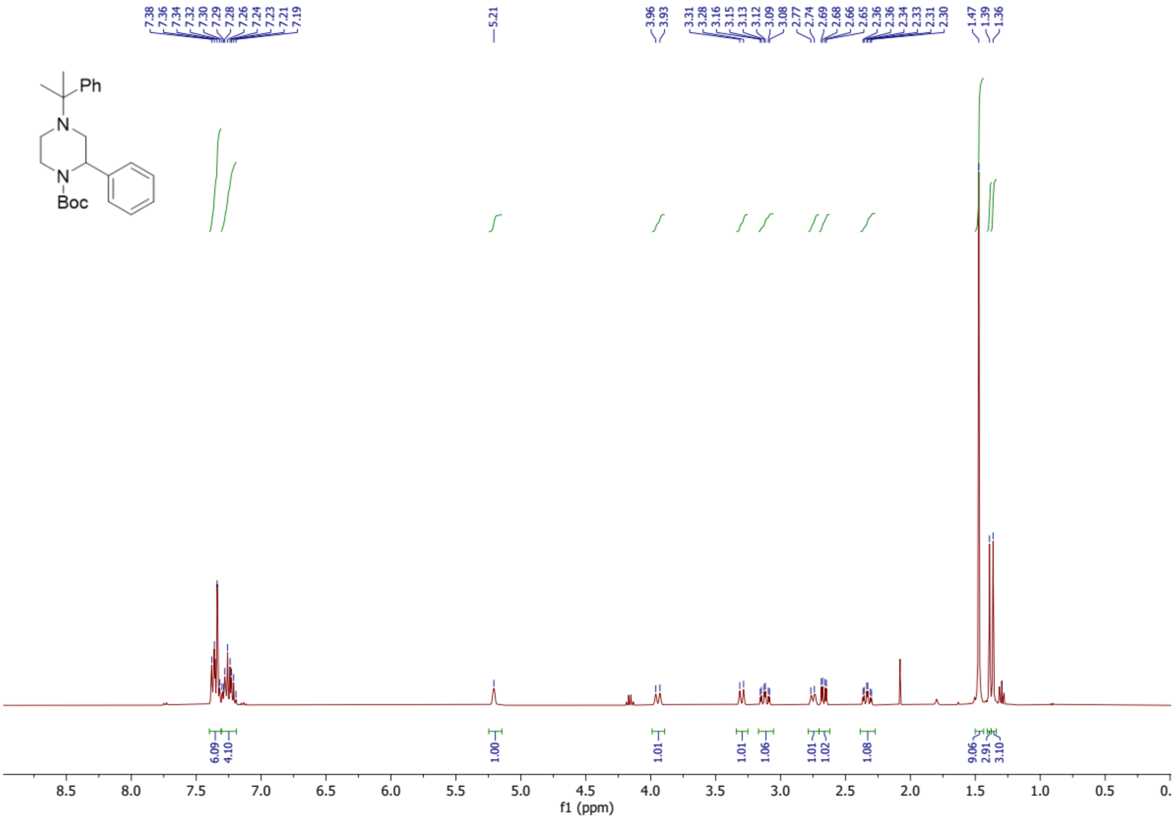

^{13}C {^{1}H} NMR spectrum ($CDCl_3$, 100 MHz) of **2g:**

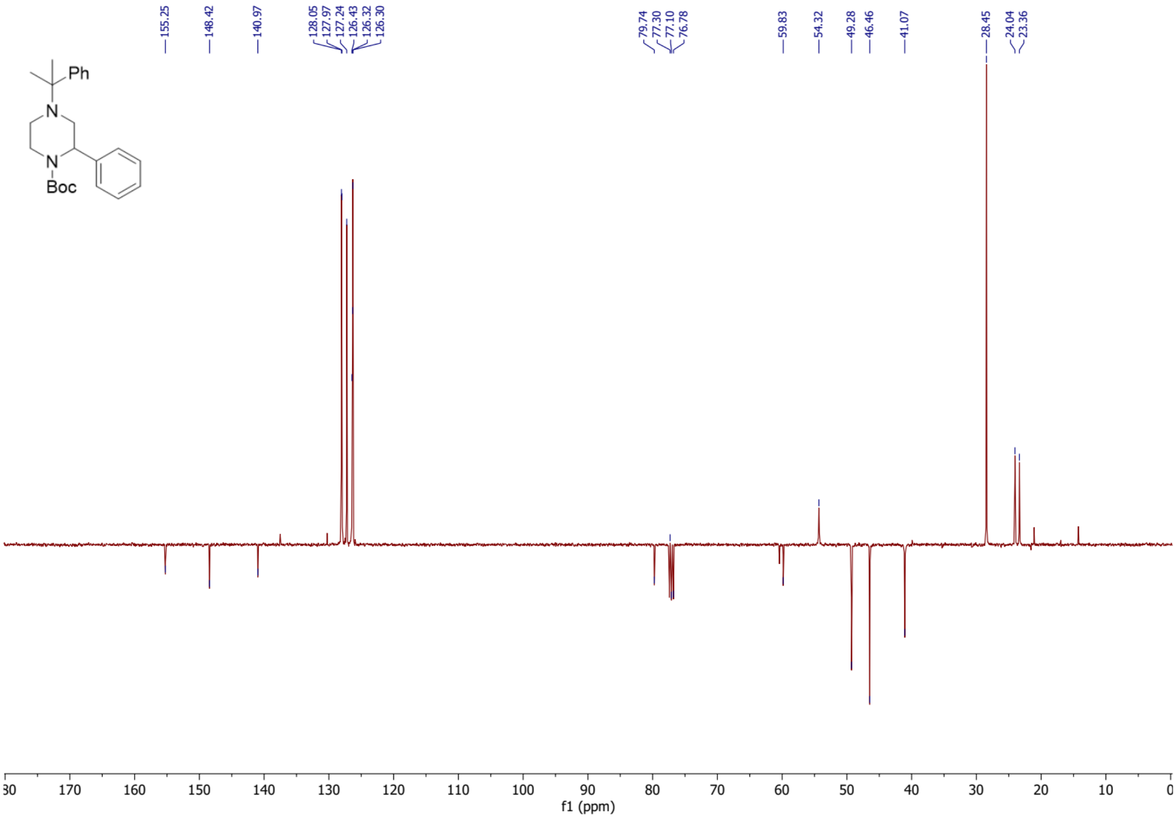

^{1}H NMR spectrum ($CDCl_3$, 400 MHz) of **2h:**

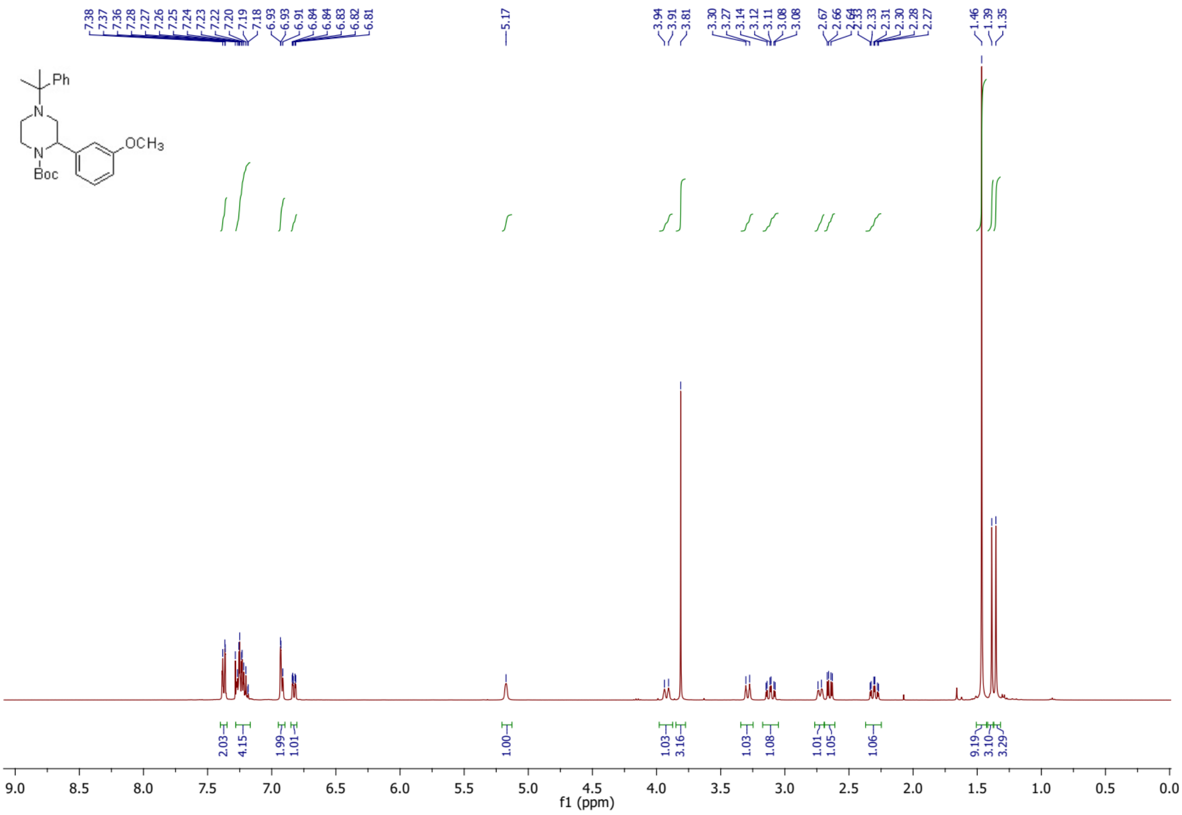

^{13}C {^{1}H} NMR spectrum ($CDCl_3$, 100 MHz) of **2h:**

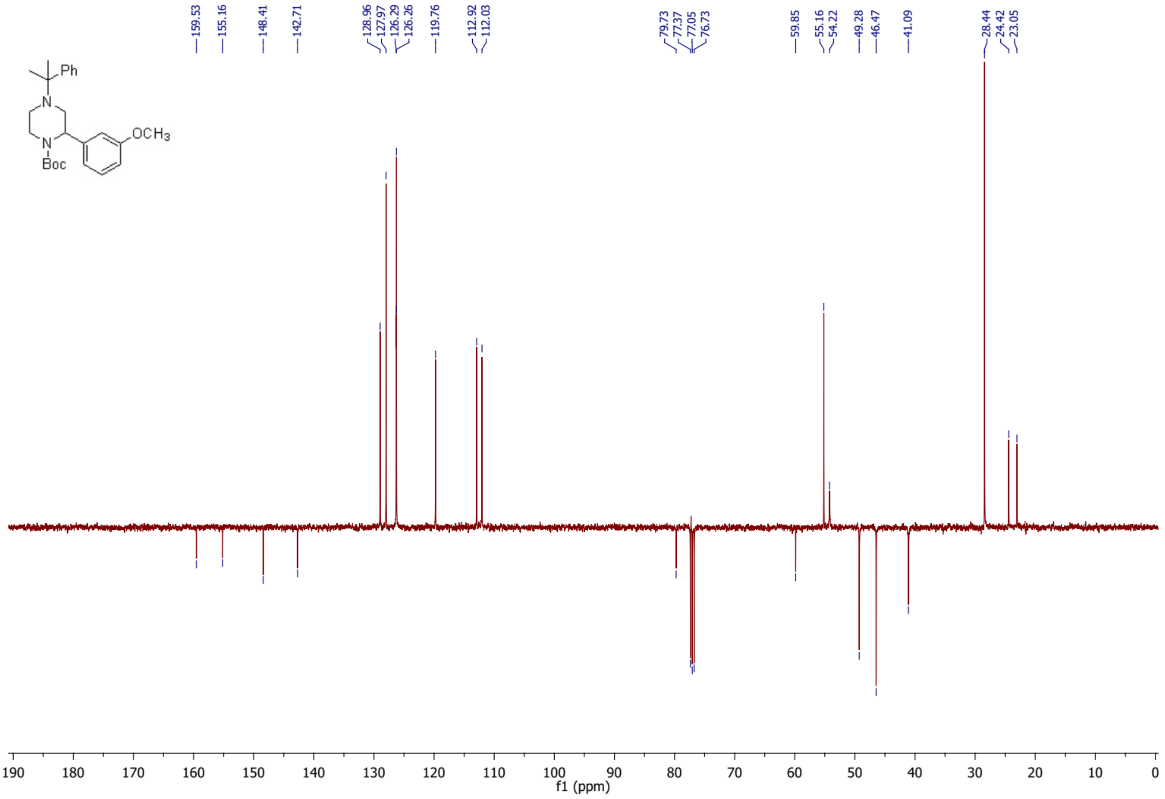

^{1}H NMR spectrum ($CDCl_3$, 400 MHz) of **2i:**

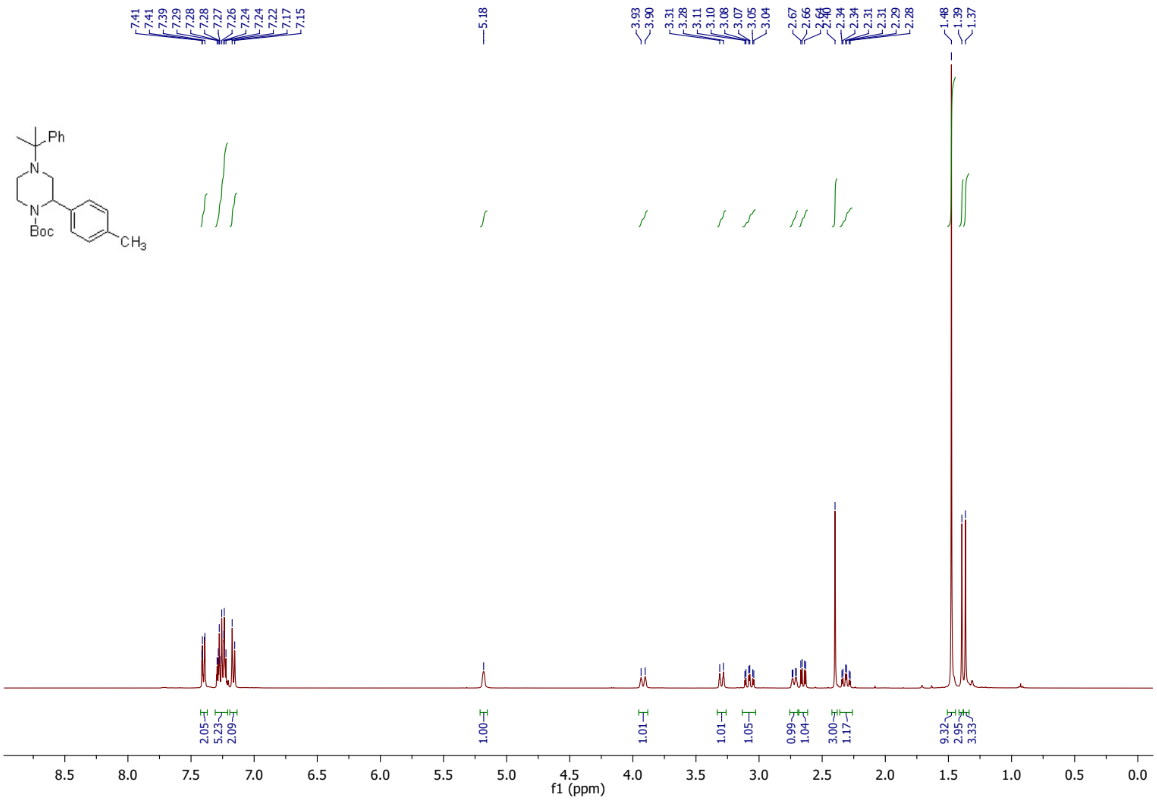

^{13}C {^{1}H} NMR spectrum ($CDCl_3$, 100 MHz) of **2i:**

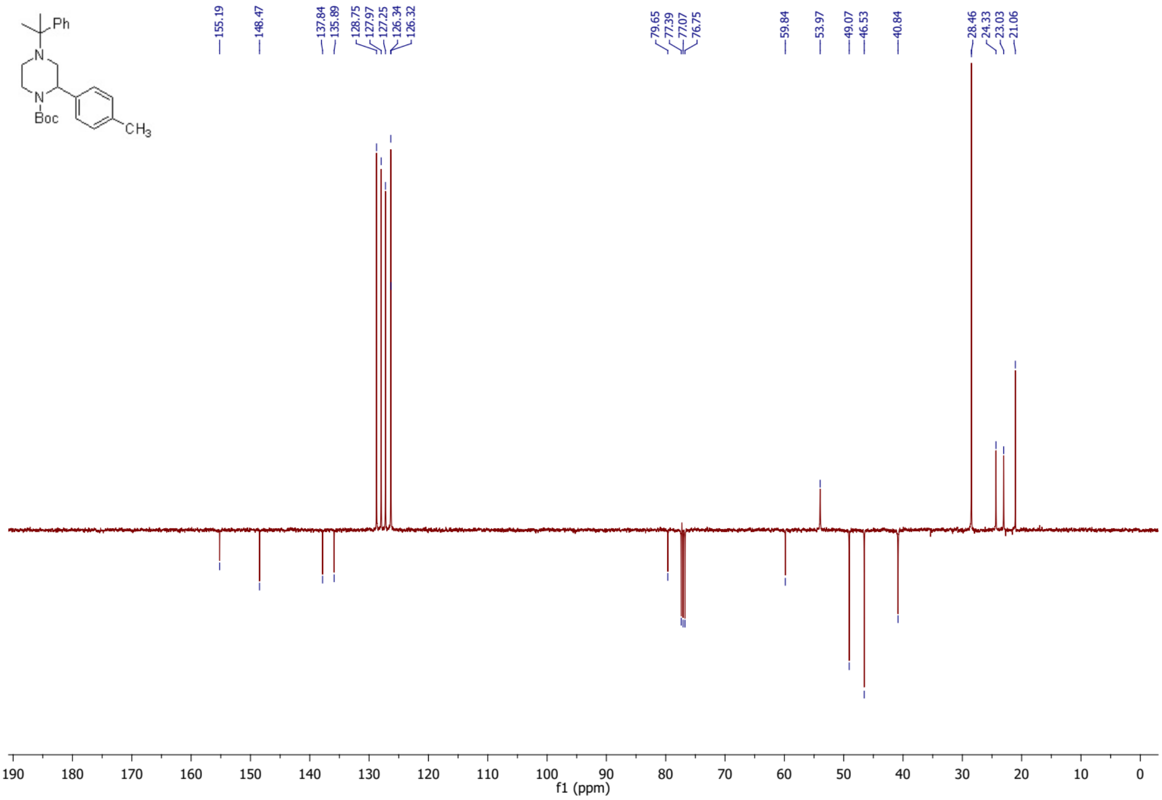

^1H NMR spectrum ($CDCl_3$, 400 MHz) of **3b:**

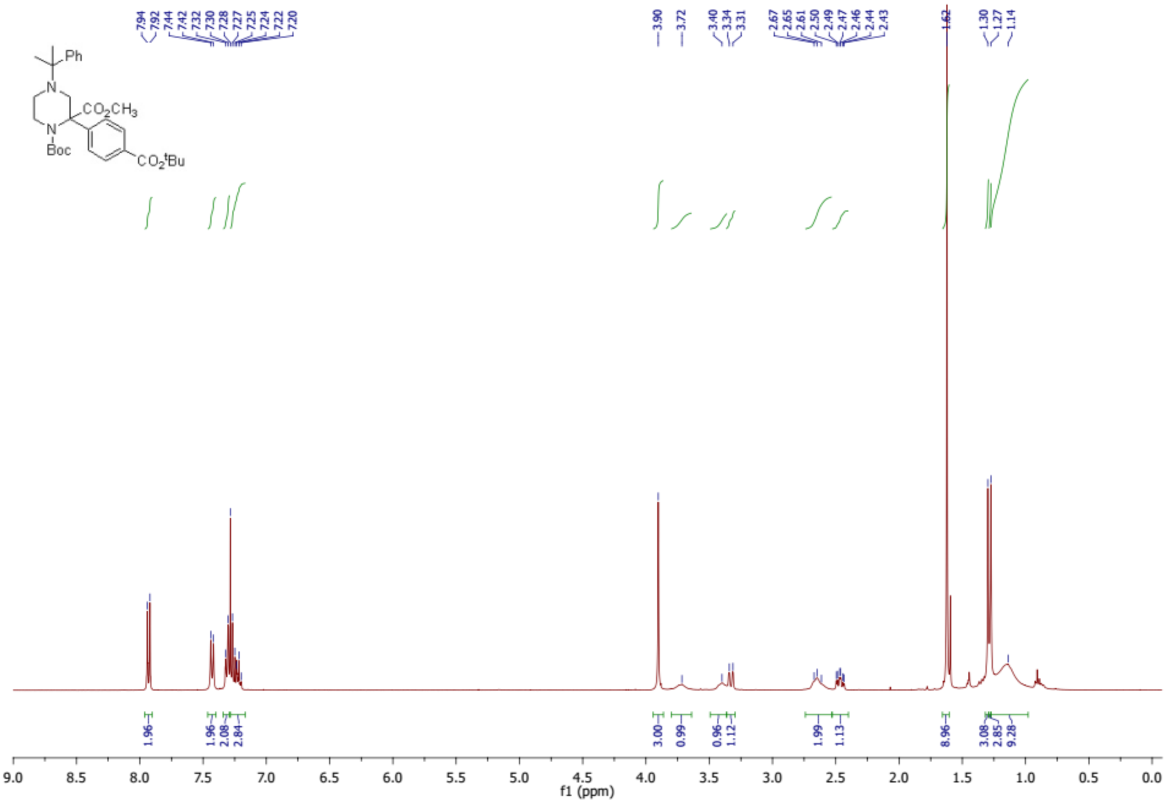

^{13}C {^1H} NMR spectrum ($CDCl_3$, 100 MHz) of **3b:**

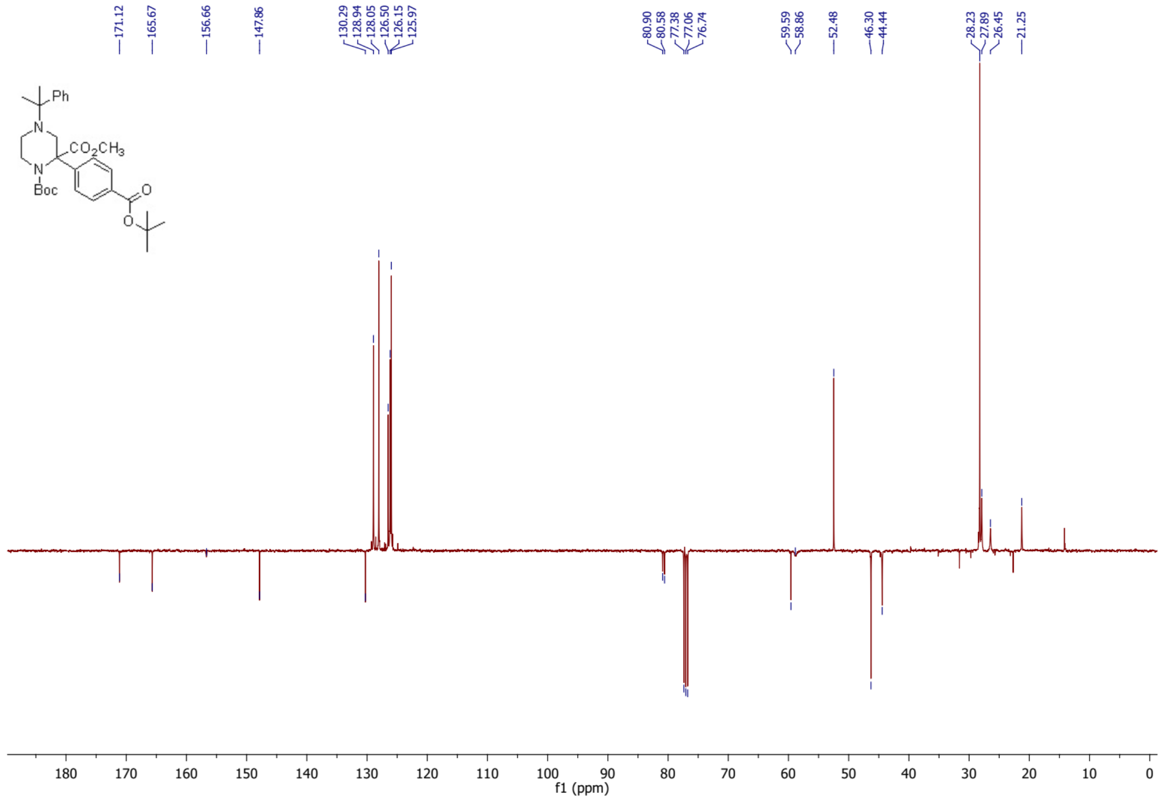

^{1}H NMR spectrum ($CDCl_3$, 400 MHz) of **3c:**

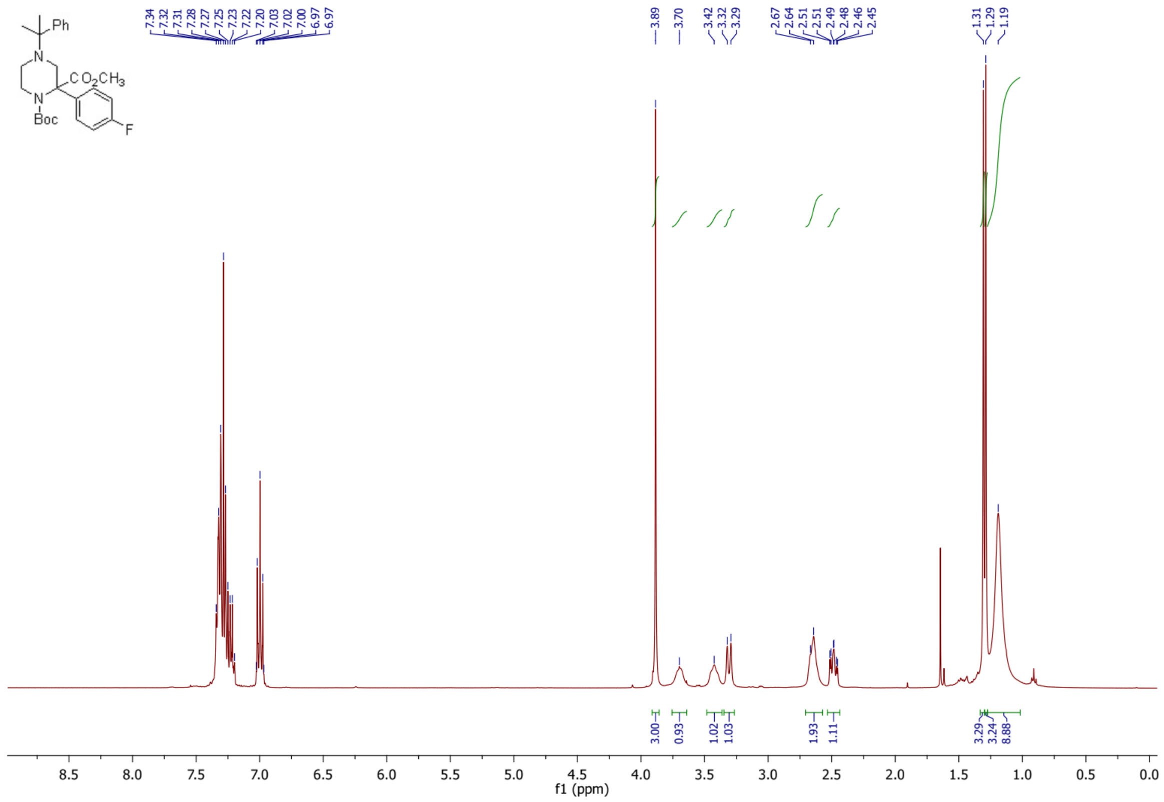

^{13}C {^{1}H} NMR spectrum ($CDCl_3$, 100 MHz) of **3c:**

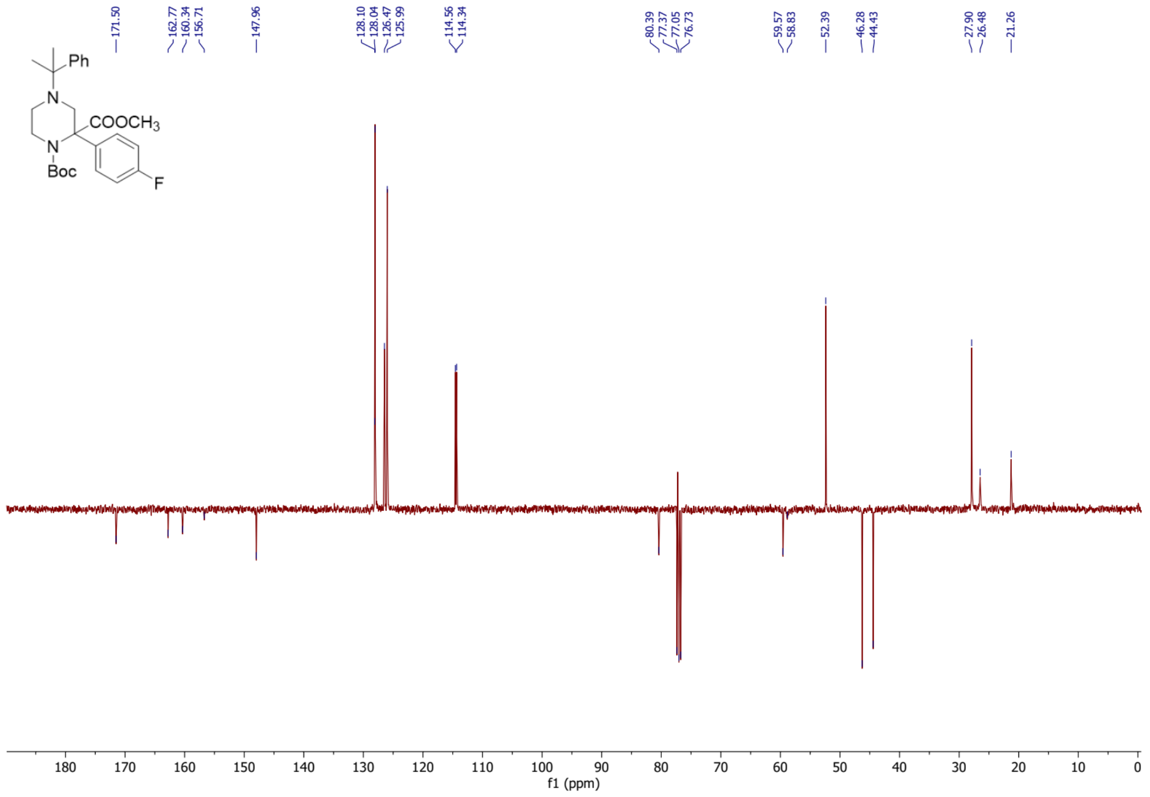

^{19}F NMR spectrum ($CDCl_3$, 377 MHz) of **3c**:

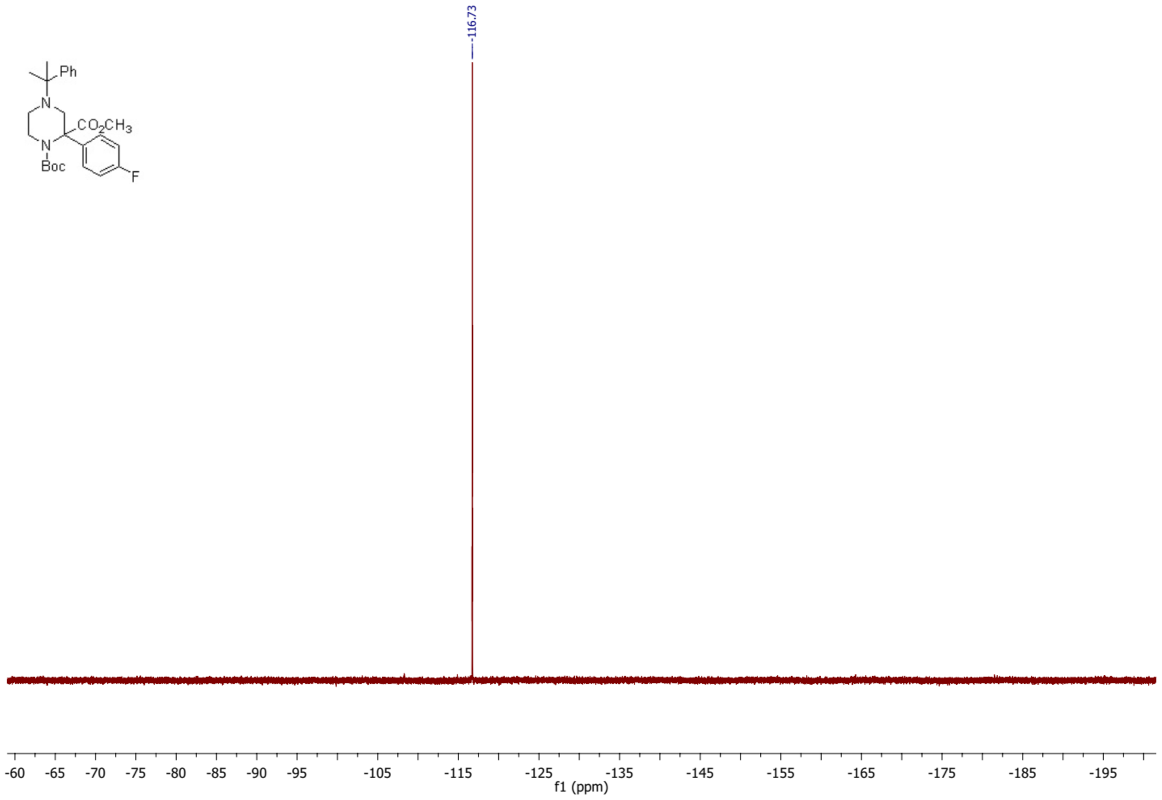

^{1}H NMR spectrum ($CDCl_3$, 400 MHz) of **3f:**

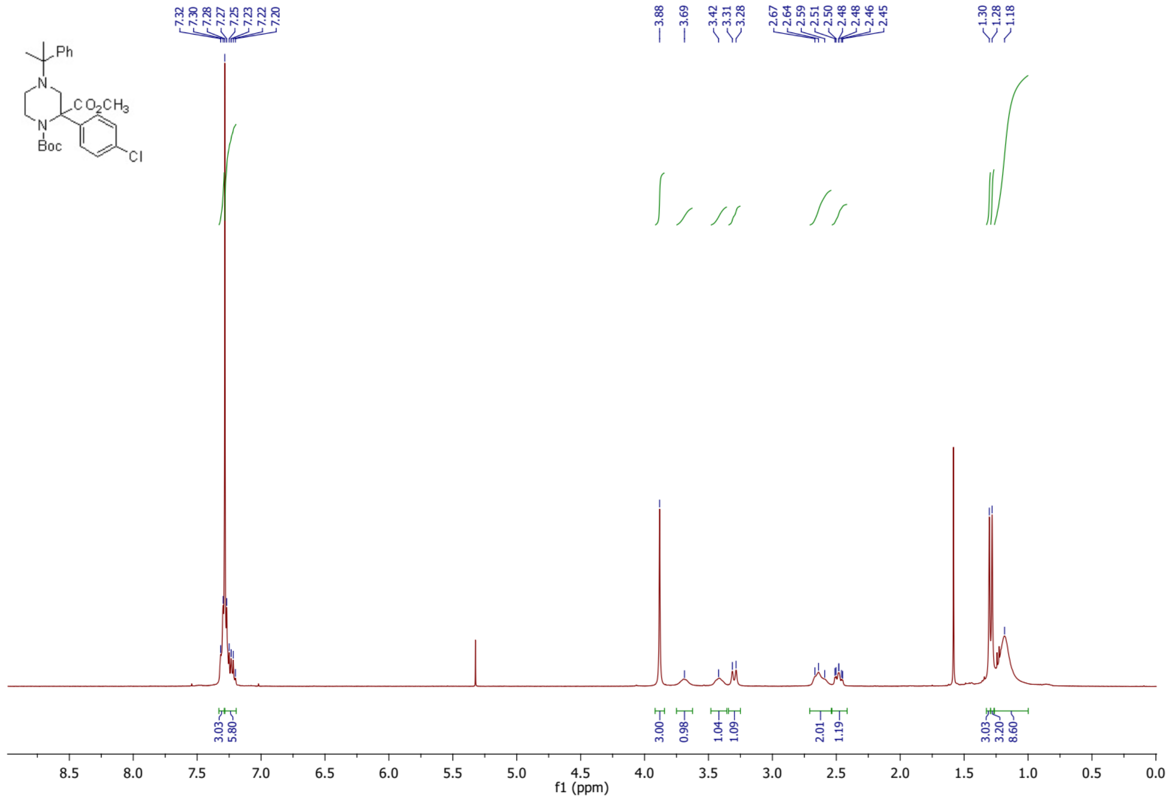

$^{13}C\{^1H\}$ NMR spectrum ($CDCl_3$, 100 MHz) of **3f:**

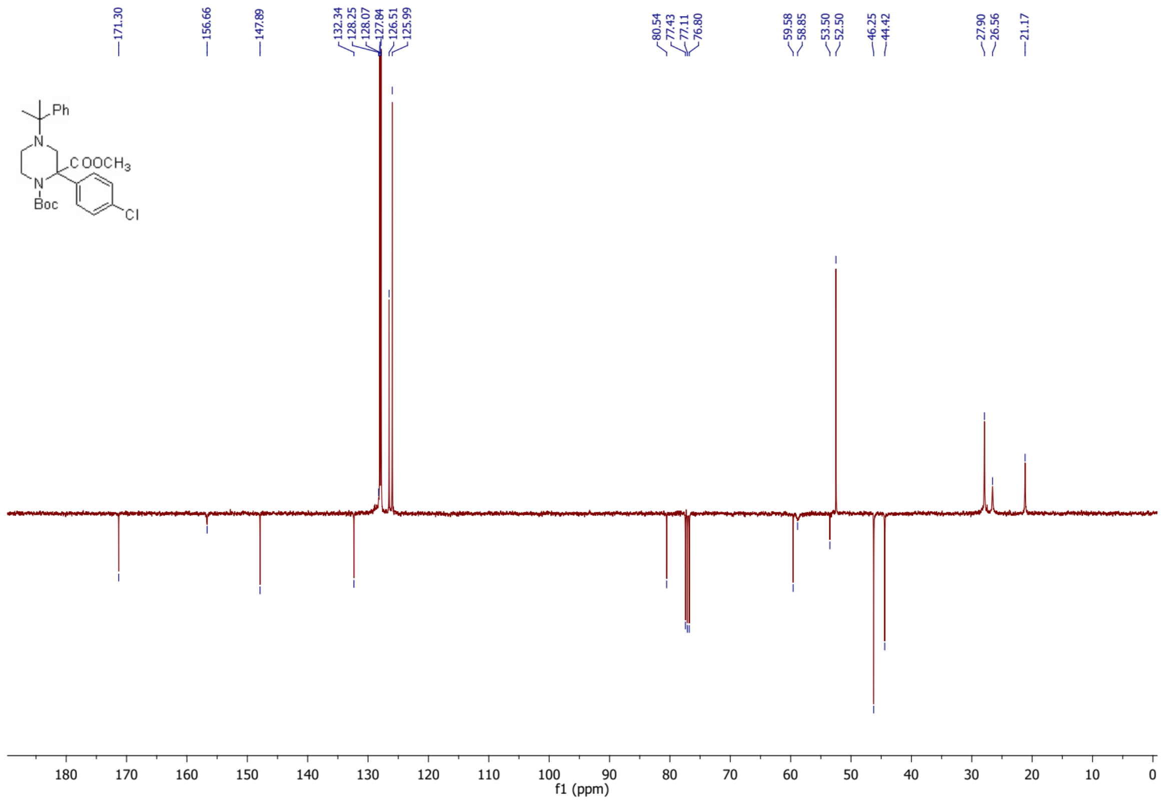

^1H NMR spectrum ($CDCl_3$, 400 MHz) of **3g:**

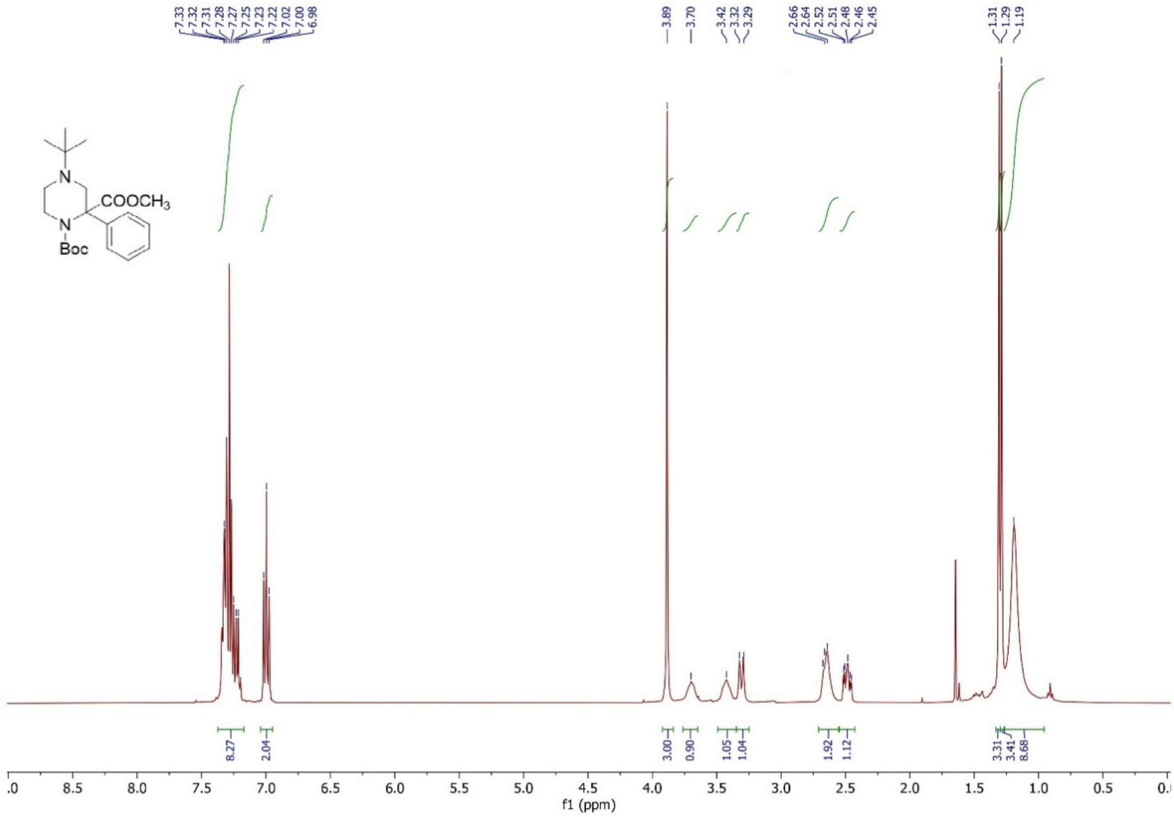

^{13}C {^{1}H} NMR spectrum ($CDCl_3$, 100 MHz) of **3g:**

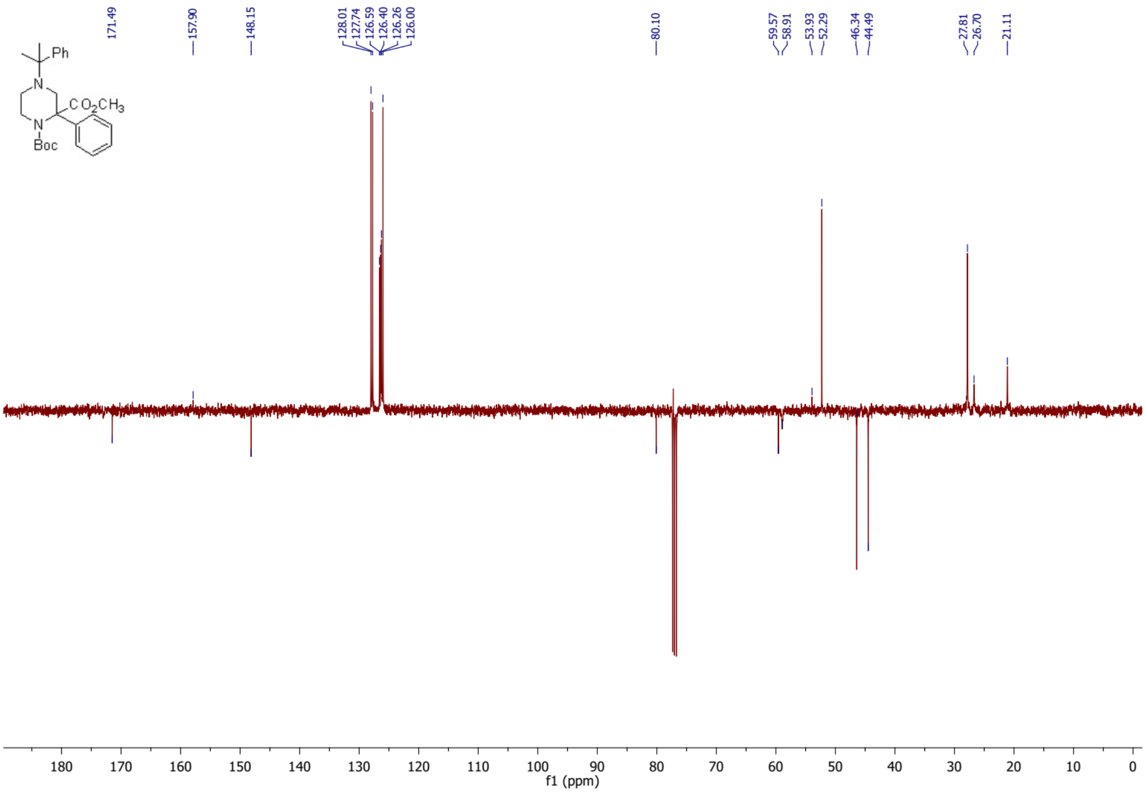

^{1}H NMR spectrum ($CDCl_3$, 400 MHz) of **3i:**

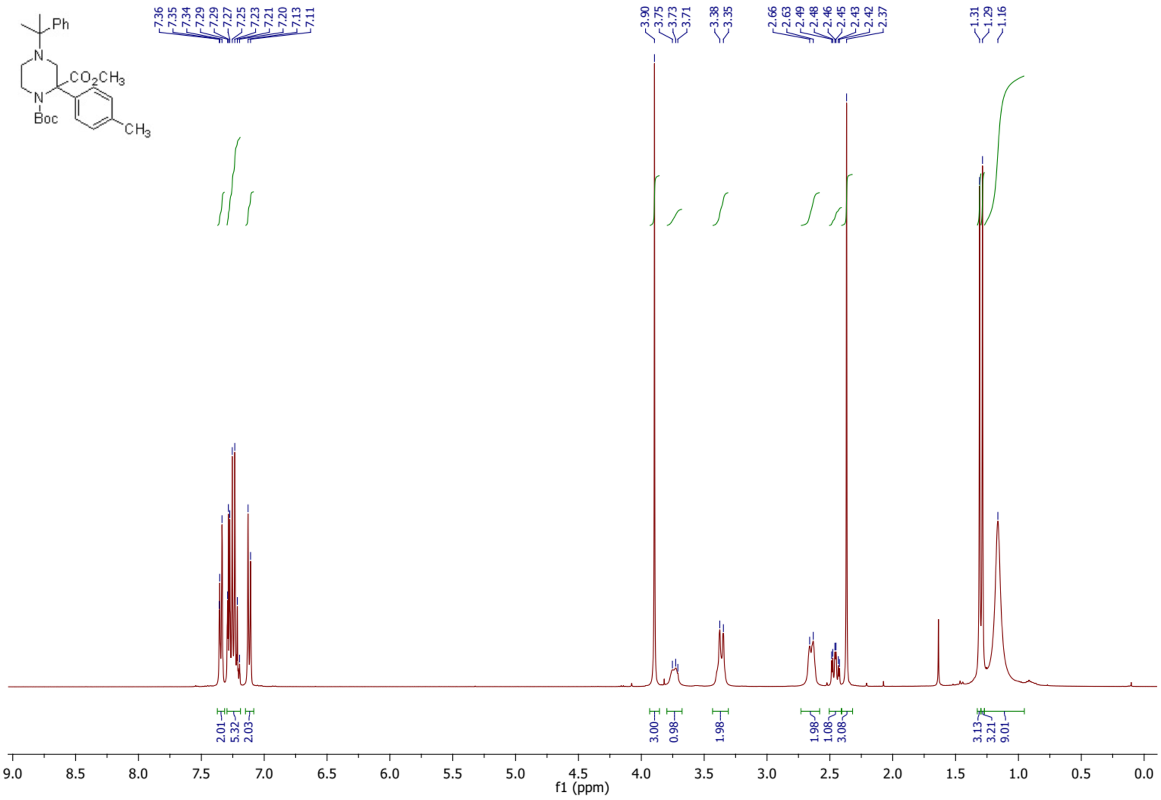

$^{13}C\ \{^{1}H\}$ NMR spectrum ($CDCl_3$, 100 MHz) of **3i:**

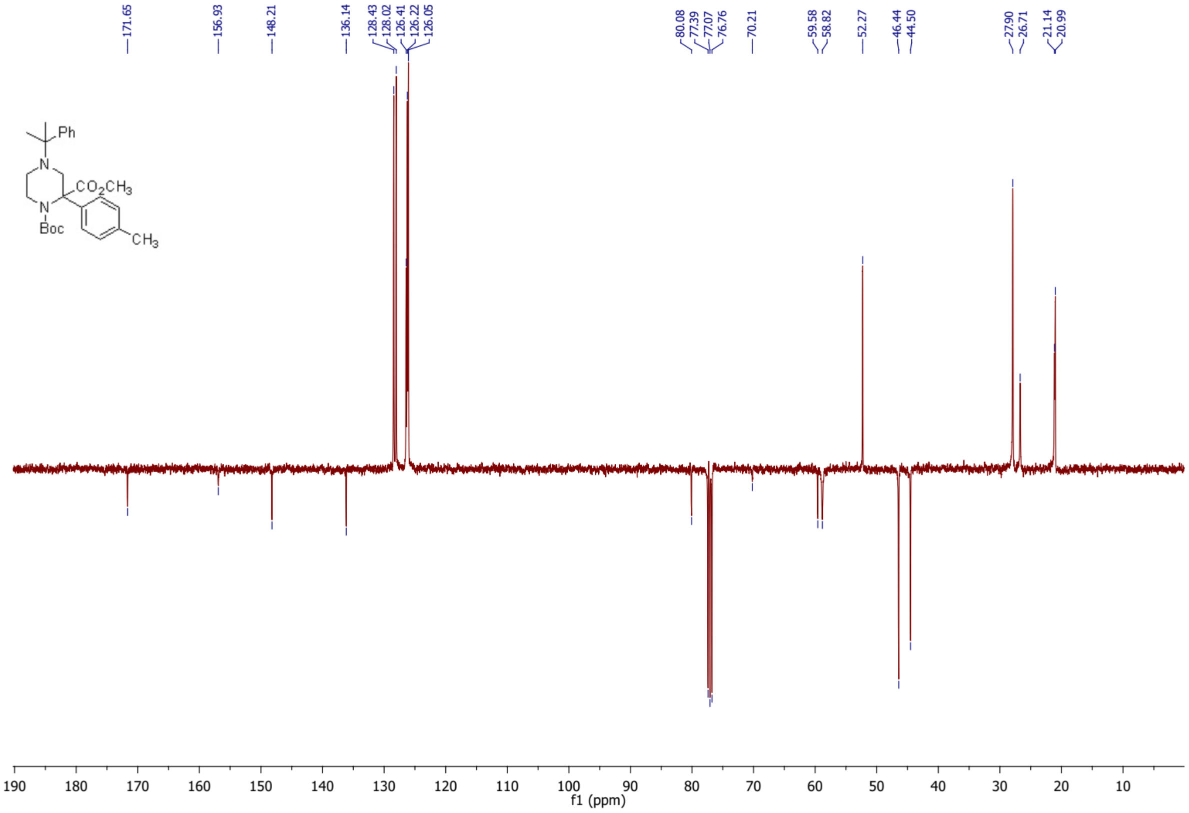

^{1}H NMR spectrum ($CDCl_3$, 400 MHz) of **4:**

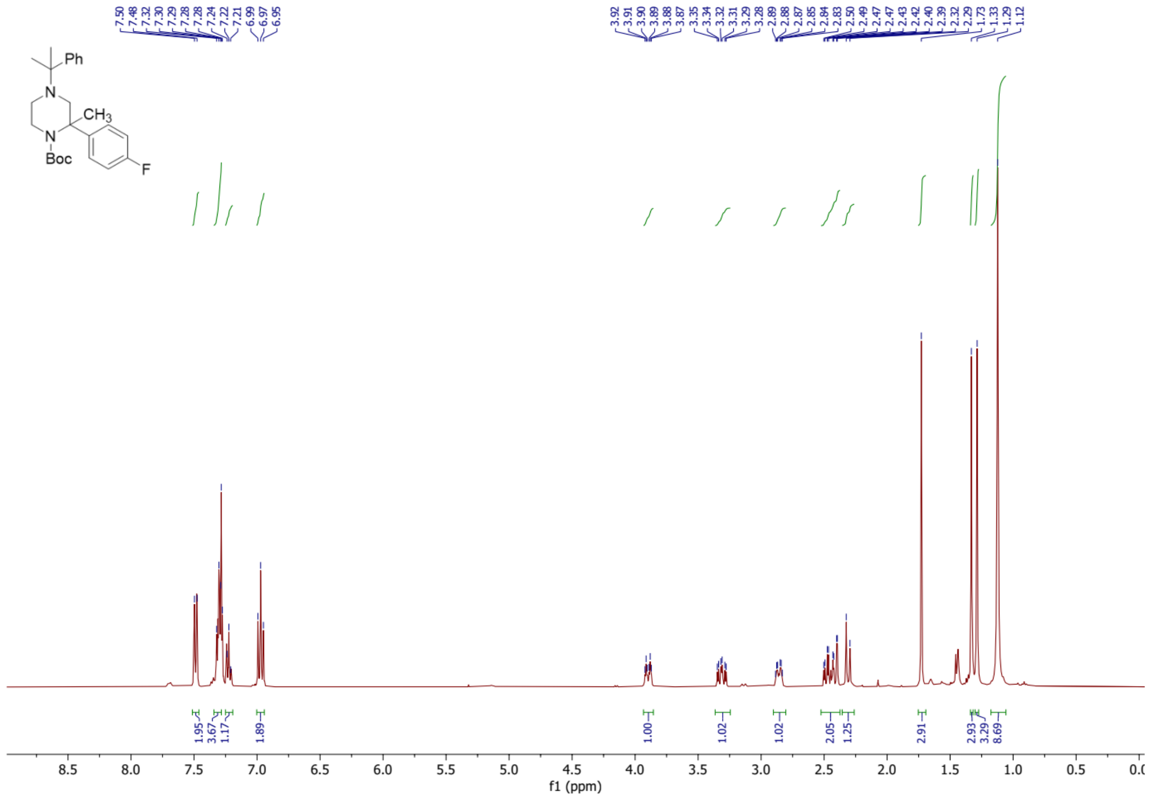

$^{13}C\ \{^1H\}$ NMR spectrum ($CDCl_3$, 100 MHz) of **4:**

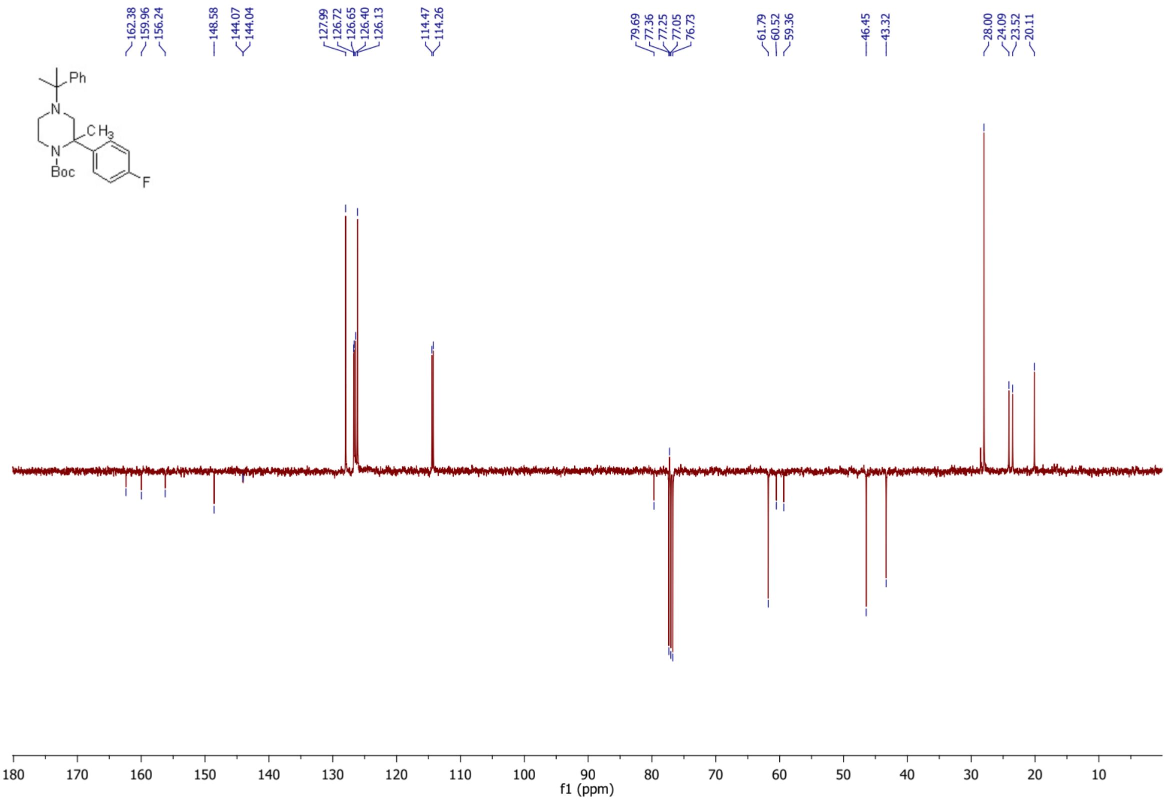

^{19}F NMR spectrum ($CDCl_3$, 377 MHz) of **4**:

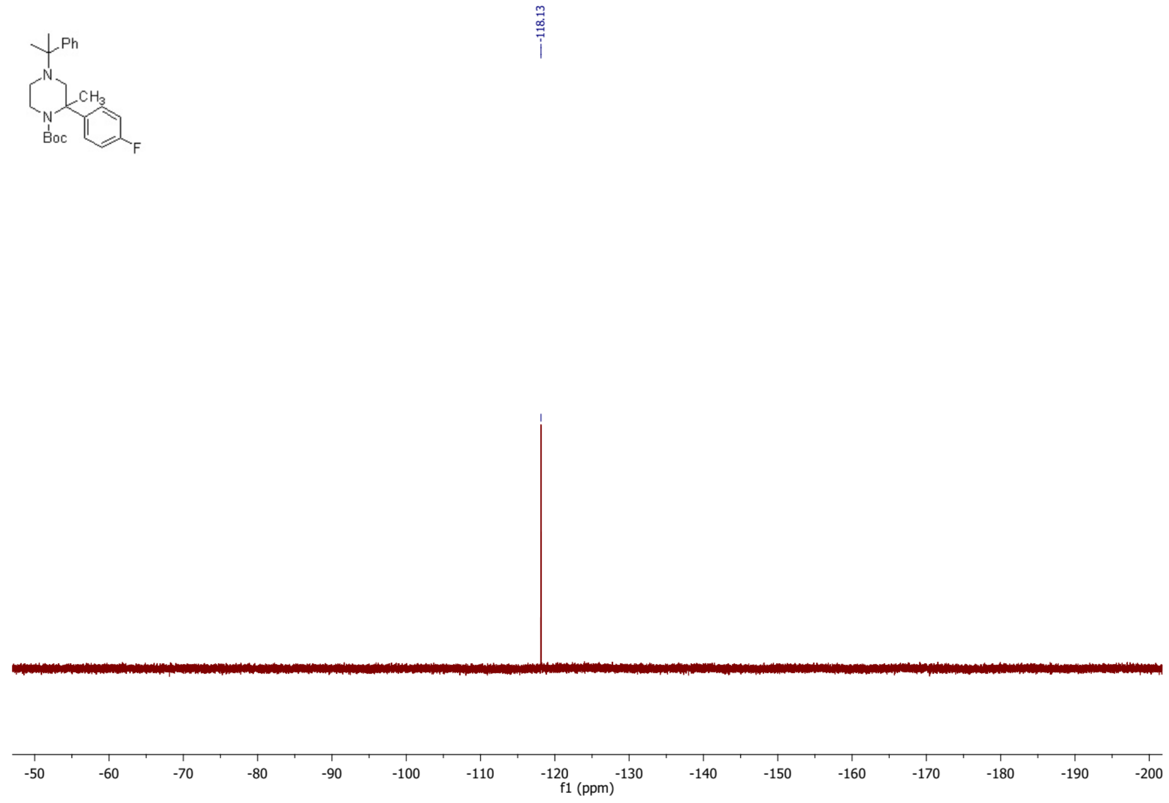

^{1}H NMR spectrum ($CDCl_3$, 400 MHz) of **5:**

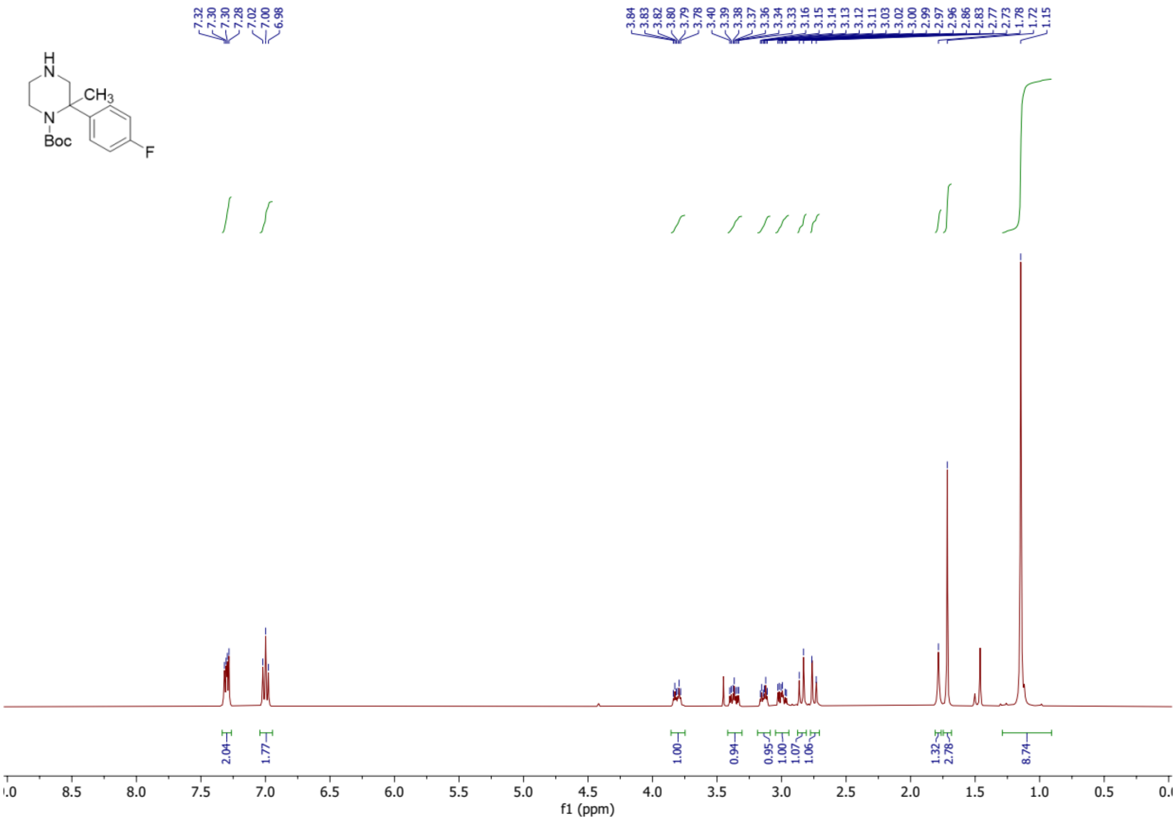

^{13}C {^{1}H} NMR spectrum ($CDCl_3$, 100 MHz) of **5:**

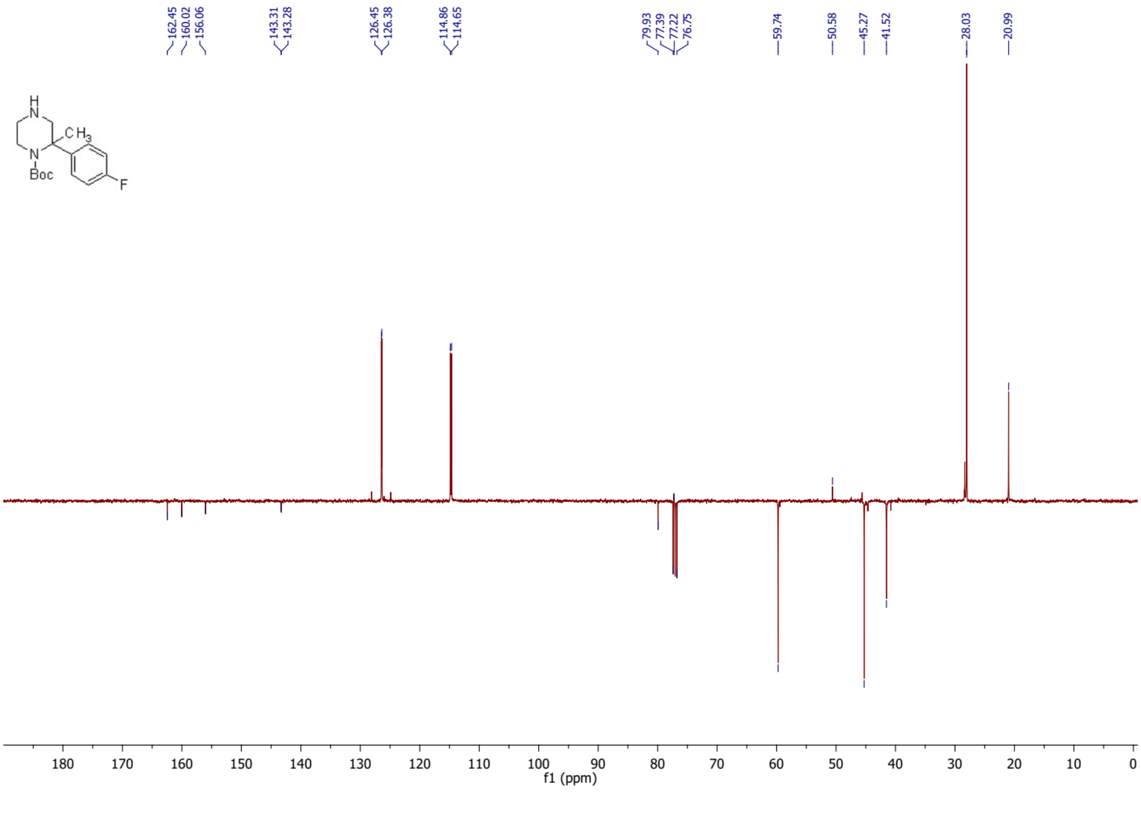

^{19}F NMR spectrum ($CDCl_3$, 377 MHz) of **5**:

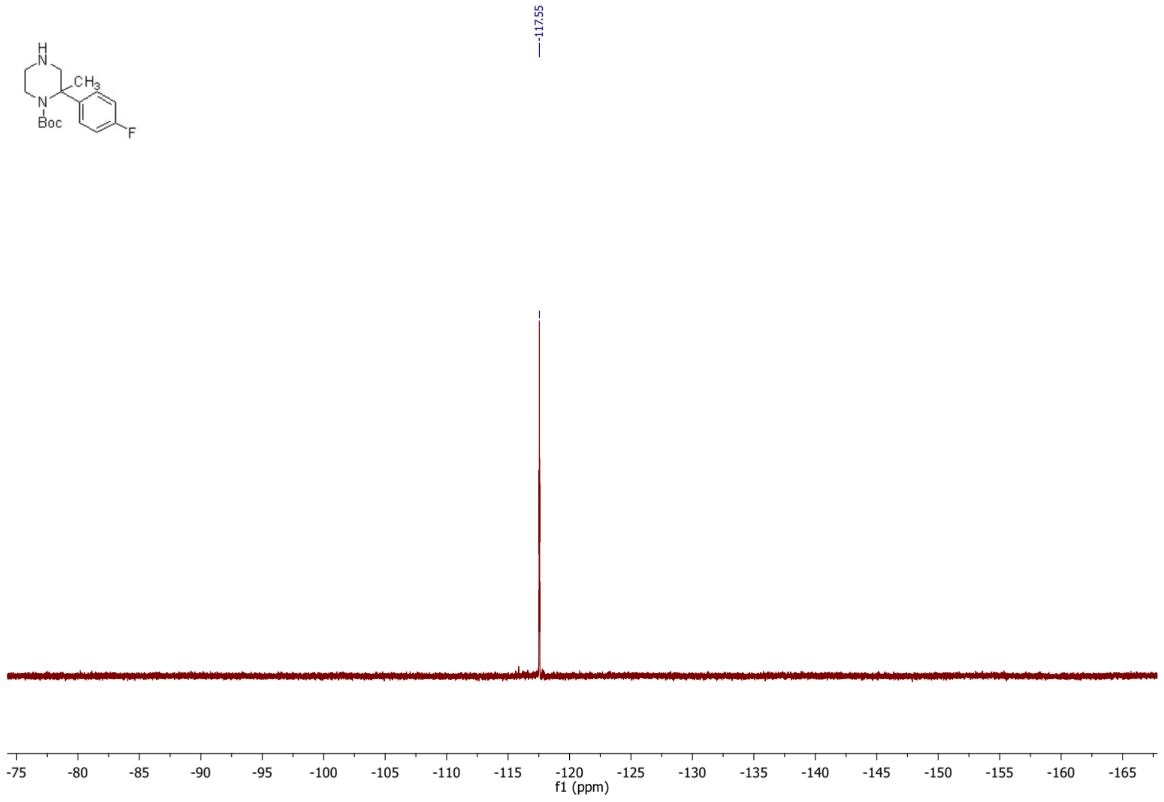

^{1}H NMR spectrum ($CDCl_3$, 400 MHz) of **6:**

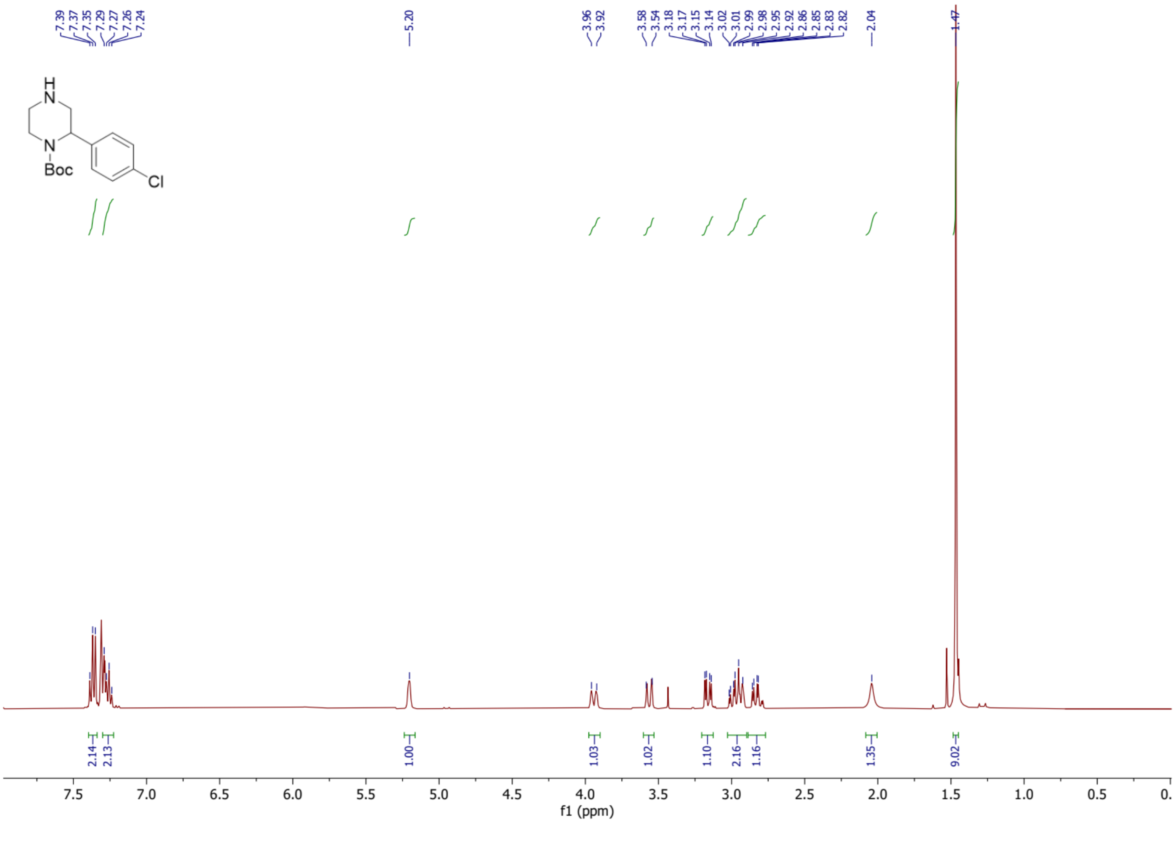

^{13}C {^{1}H} NMR spectrum ($CDCl_3$, 100 MHz) of **6:**

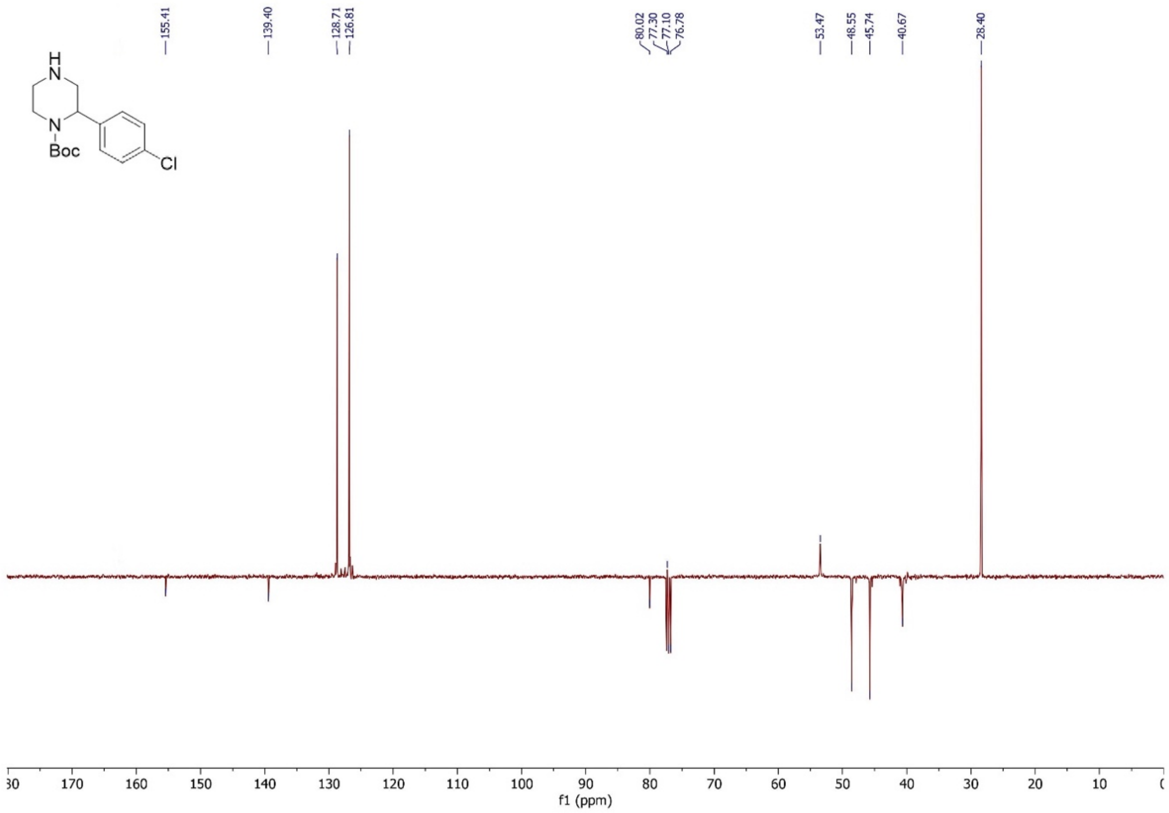

^{1}H NMR spectrum ($CDCl_3$, 400 MHz) of **7:**

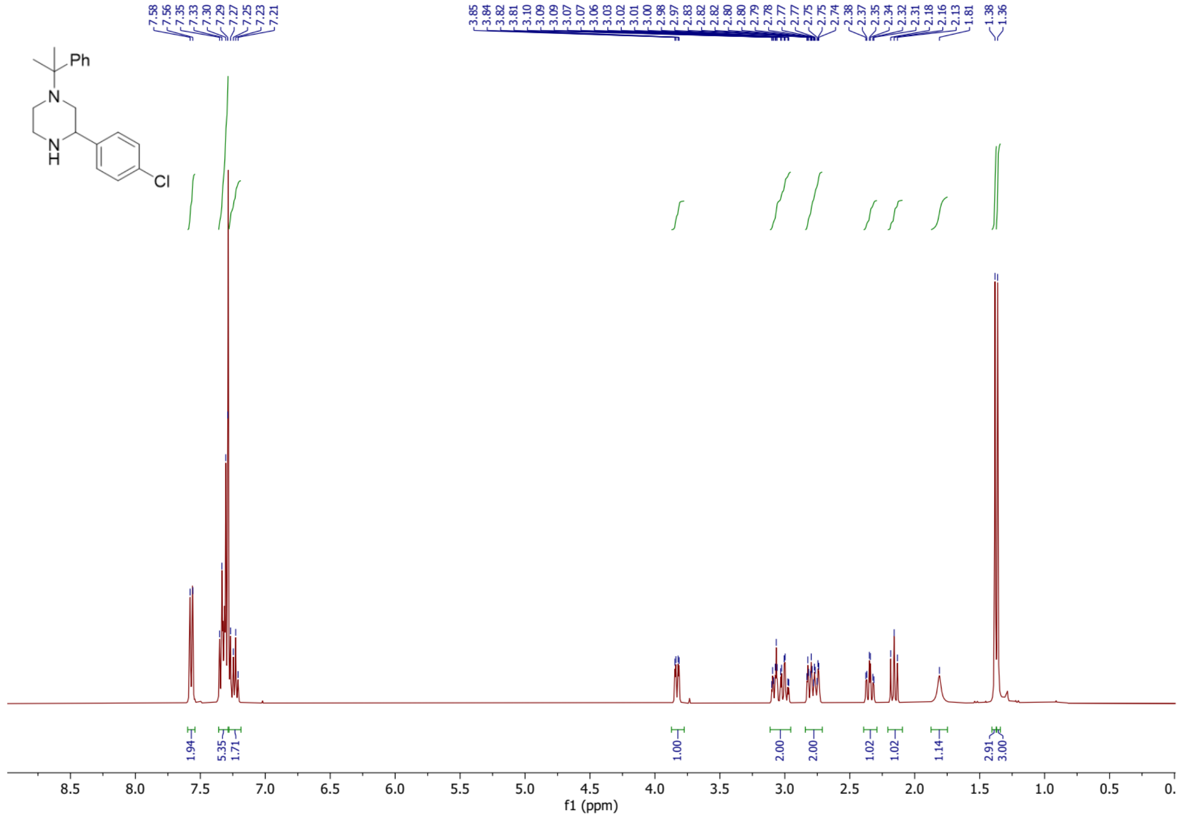

$^{13}C\ \{^1H\}$ NMR spectrum ($CDCl_3$, 100 MHz) of **7:**

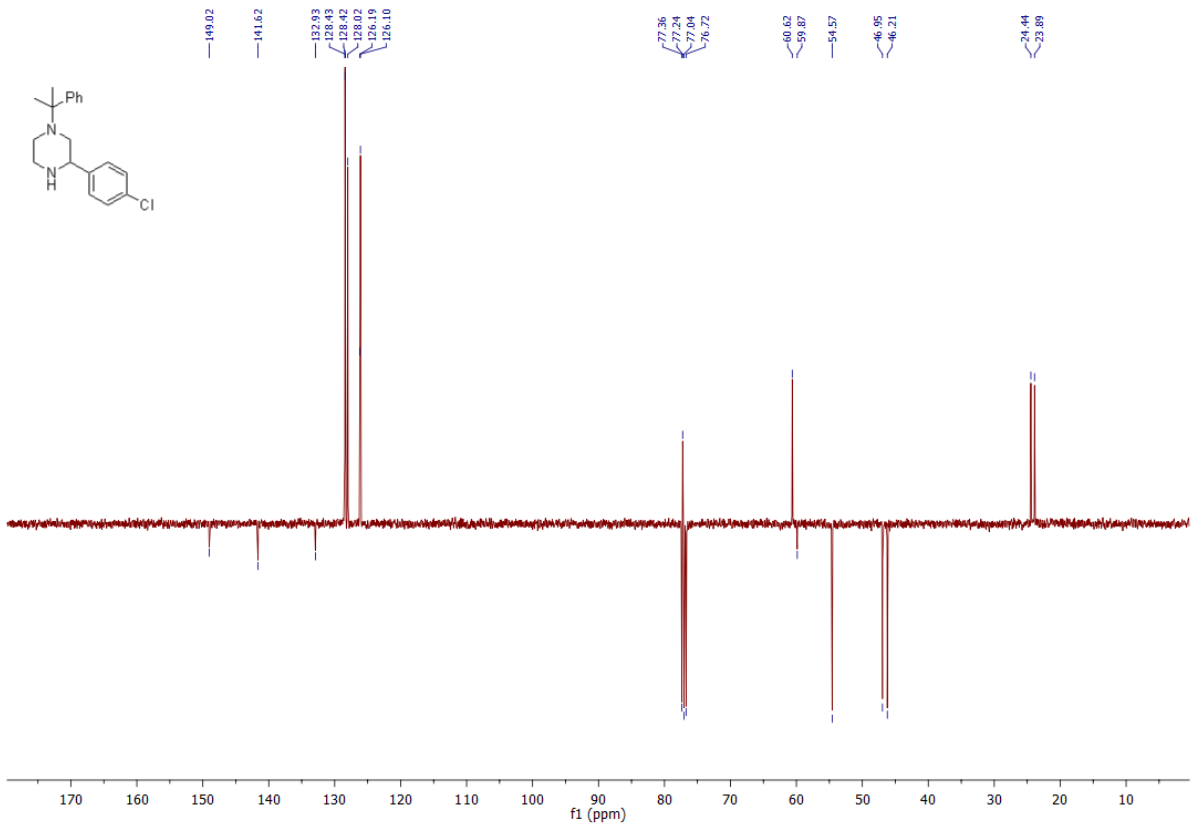

^1H NMR spectrum ($CDCl_3$, 400 MHz) of **8:**

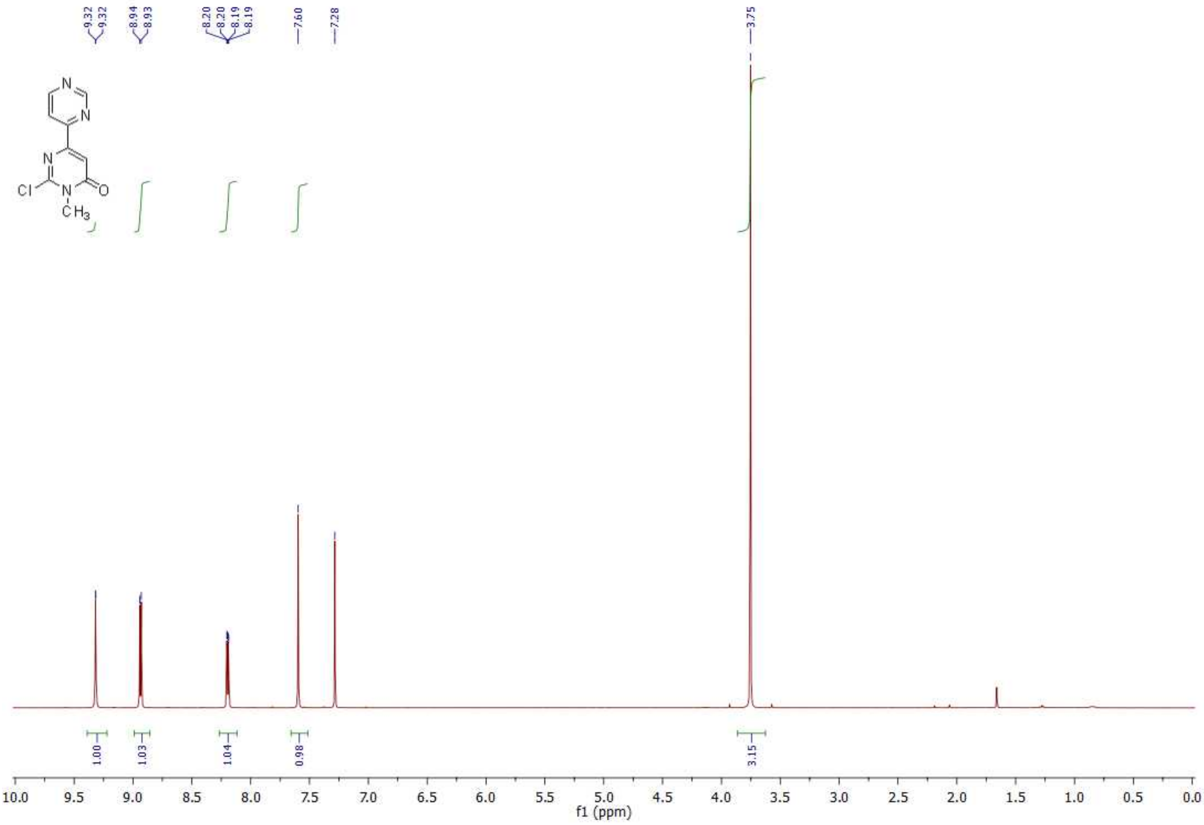

^{1}H NMR spectrum ($CDCl_3$, 400 MHz) of N-Boc-**9:**

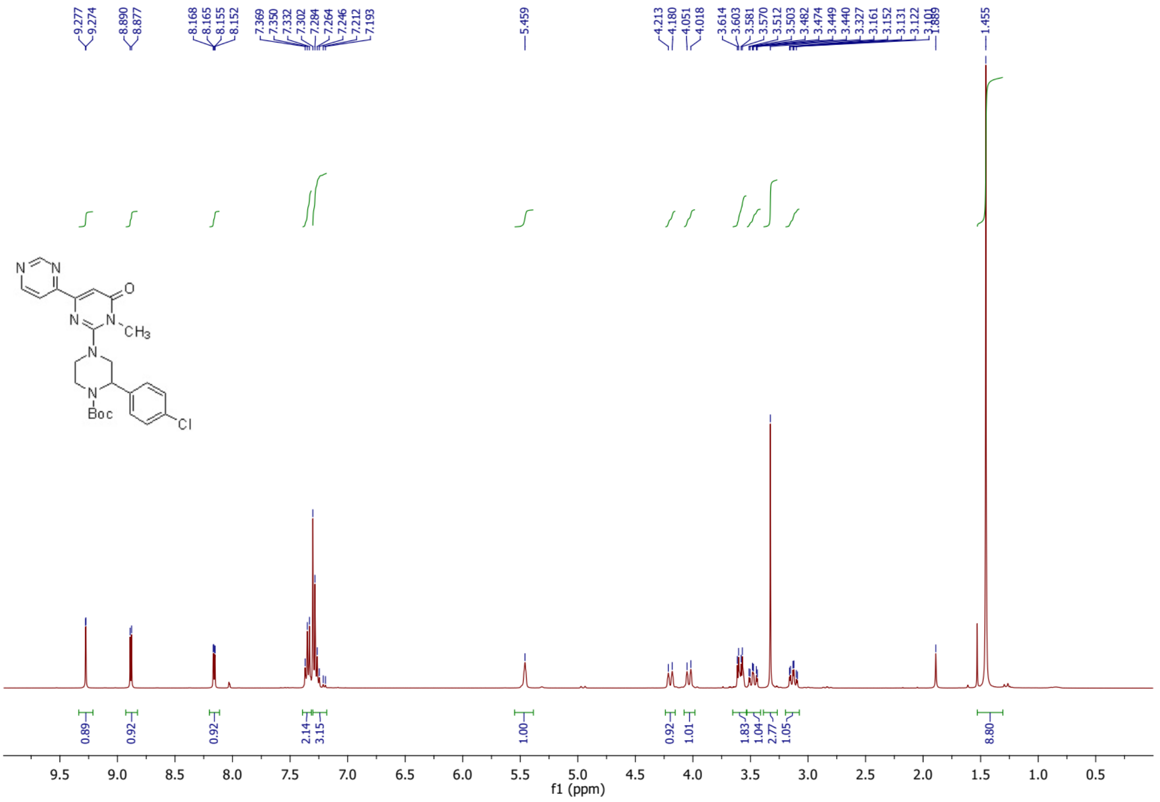

^{13}C {^{1}H} NMR spectrum ($CDCl_3$, 100 MHz) of N-Boc-**9:**

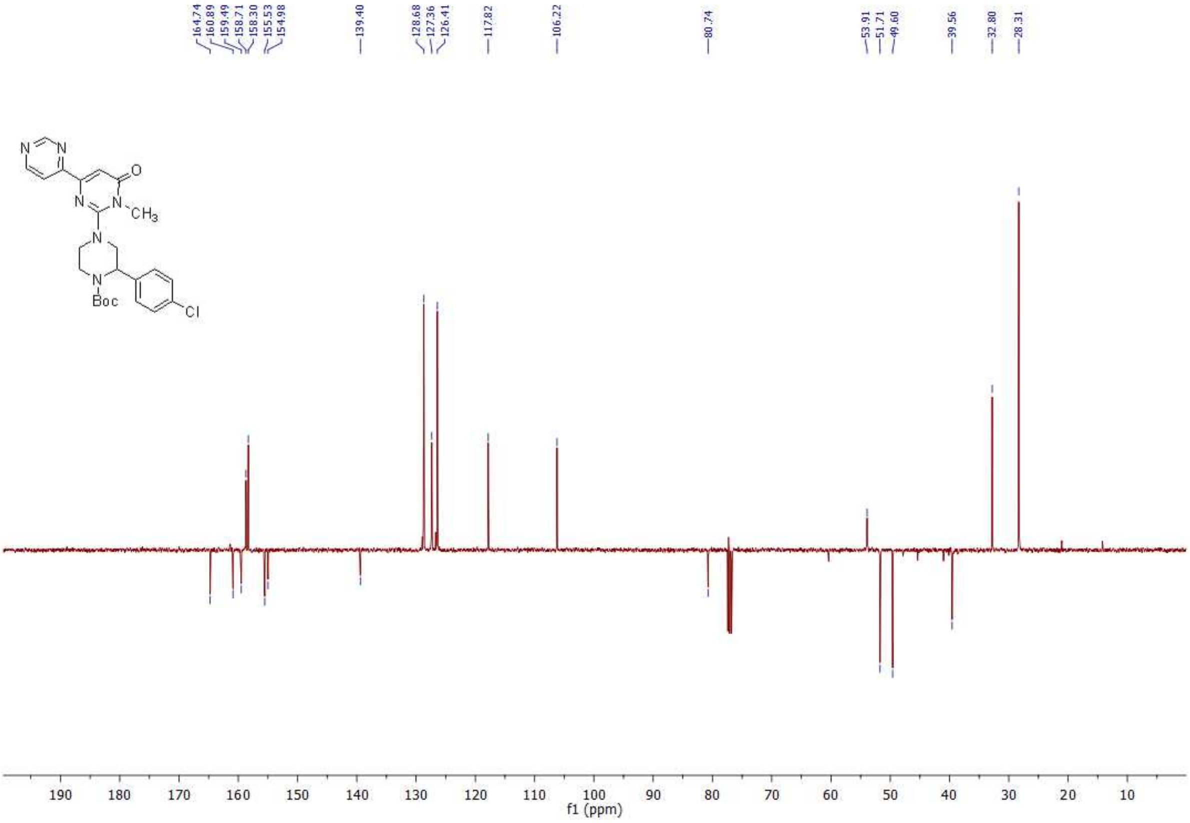

^{1}H NMR spectrum ($CDCl_3$, 400 MHz) of **9:**

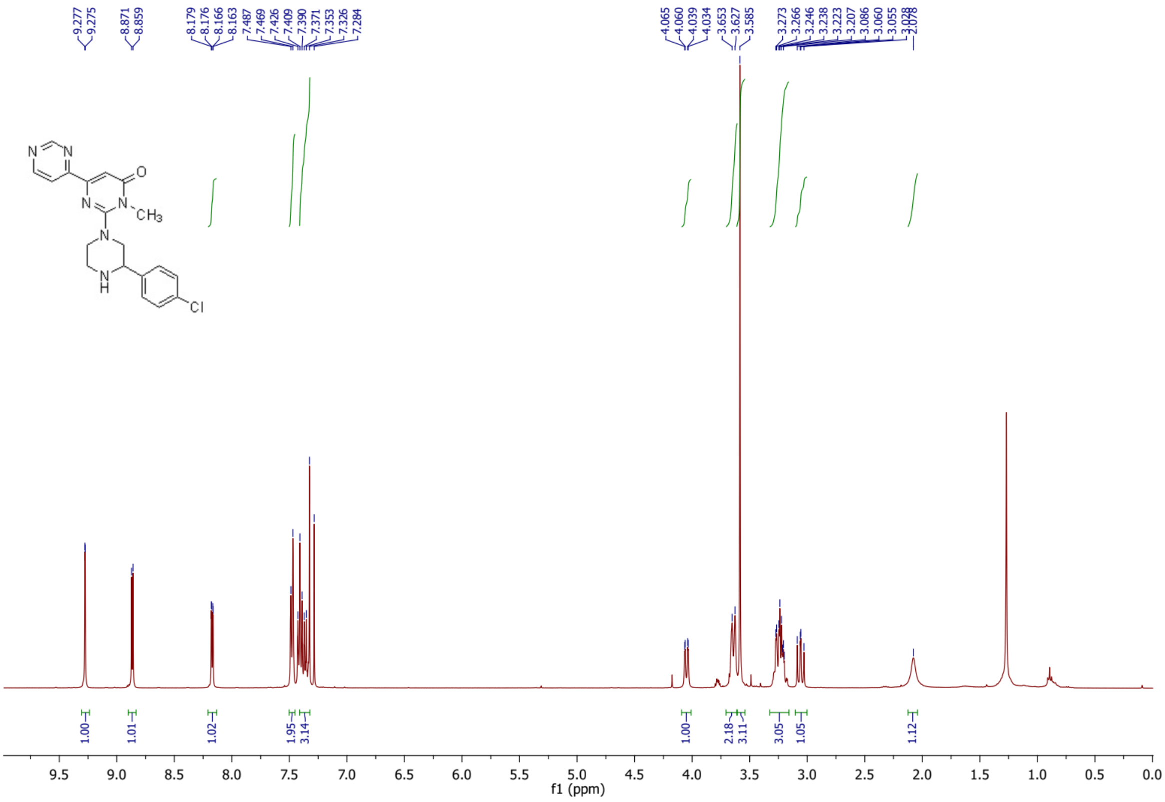

^{13}C {^{1}H} NMR spectrum ($CDCl_3$, 100 MHz) of **9:**

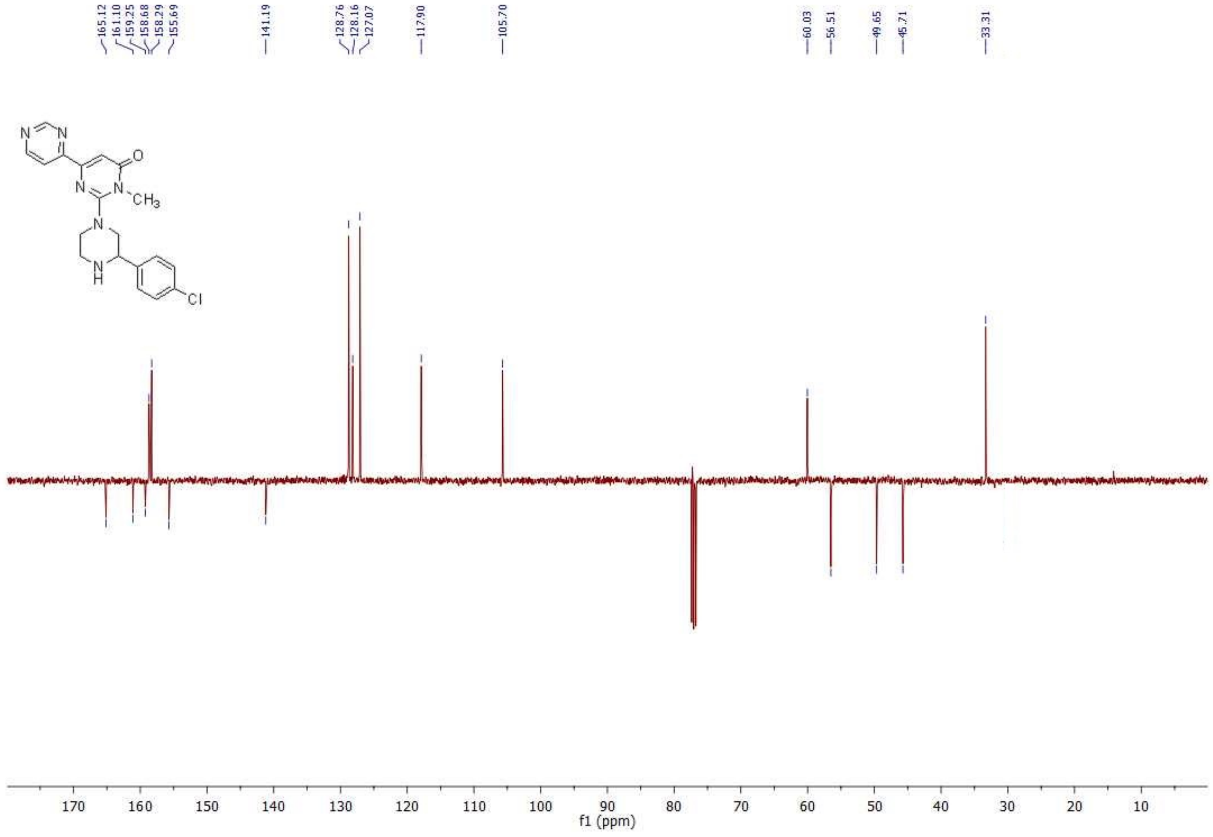

5. HPLC traces

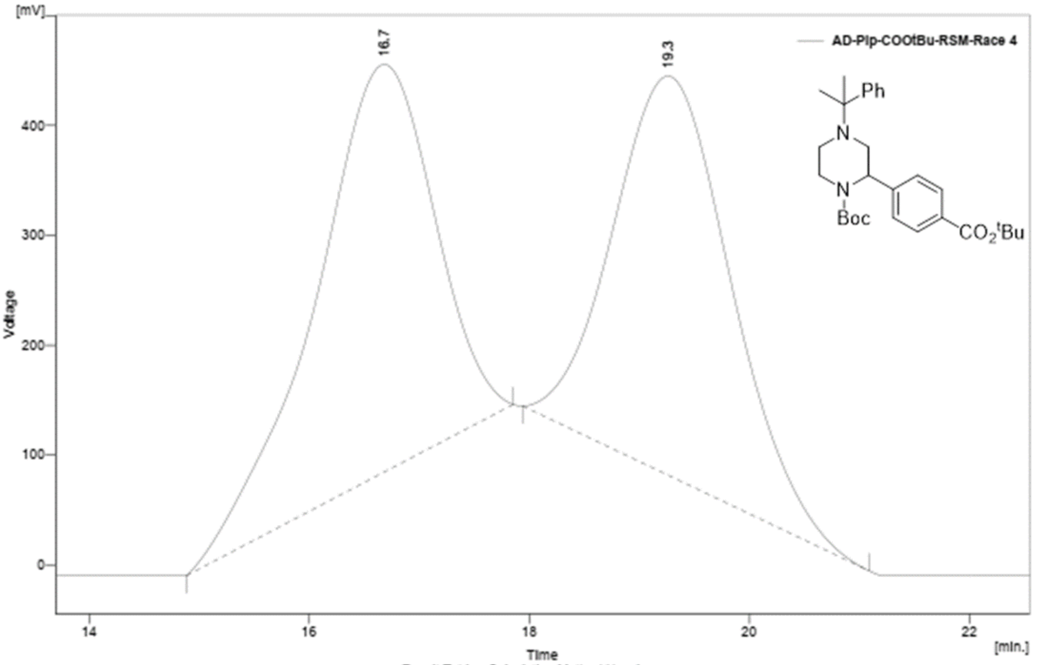

Result Table - Calculation Method Uncal

	Reten. Time [min]	Area [mV.s]	Height [mV]	Area [%]	Height [%]	W05 [min]
1	16.680	28046.437	371.145	50.8	50.5	1.12
2	19.260	27215.765	363.376	49.2	49.5	1.17
	Total	55262.202	734.521	100.0	100.0	

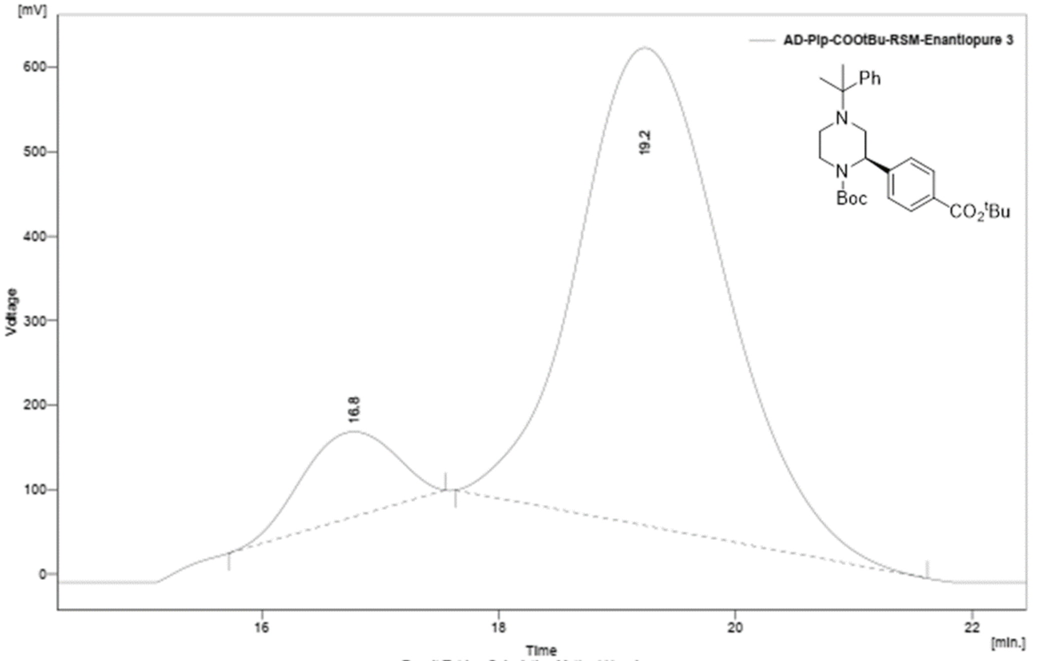

Result Table - Calculation Method Uncal

	Reten. Time [min]	Area [mV.s]	Height [mV]	Area [%]	Height [%]	W05 [min]
1	16.780	5577.203	100.583	10.2	15.1	0.94
2	19.233	49241.359	566.033	89.8	84.9	1.32
	Total	54818.562	666.615	100.0	100.0	

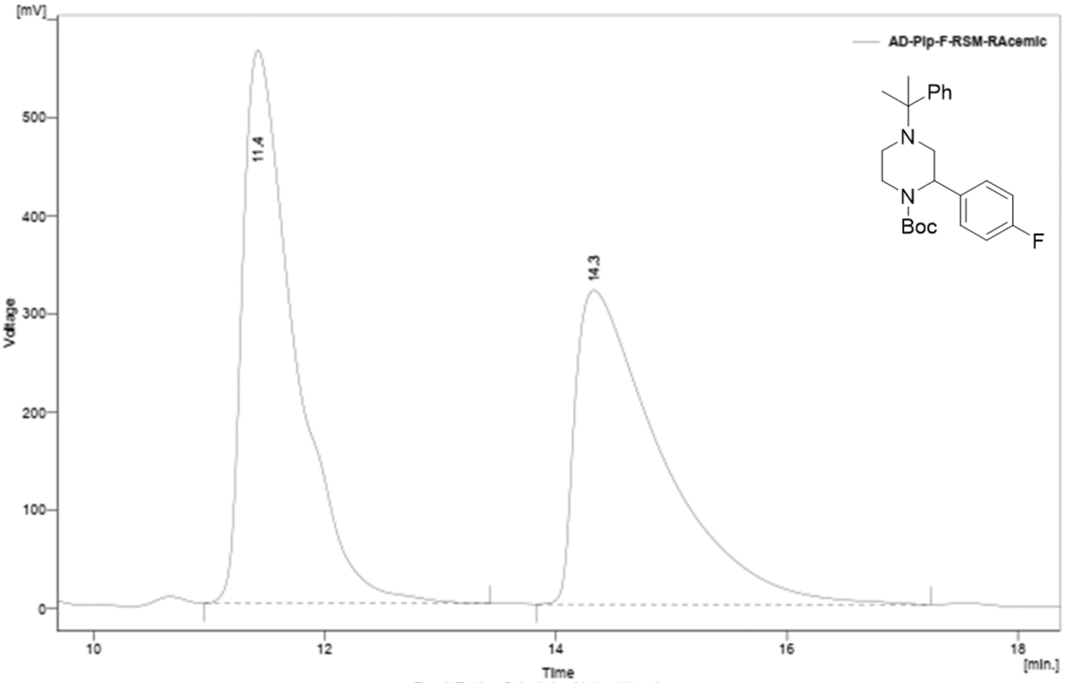

Result Table - Calculation Method Uncal

	Reten. Time [min]	Area [mV.s]	Height [mV]	Area [%]	Height [%]	W05 [min]
1	11.423	17833.996	563.804	51.8	63.8	0.45
2	14.330	16564.915	319.709	48.2	36.2	0.75
	Total	34398.911	883.513	100.0	100.0	

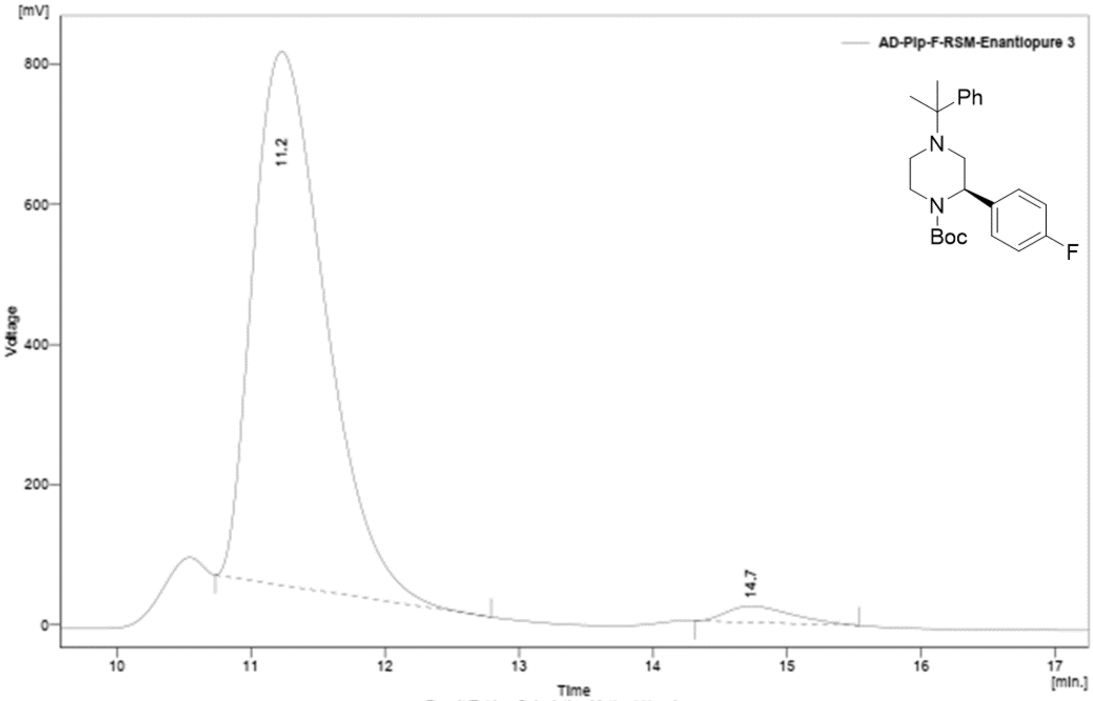

Result Table - Calculation Method Uncal

	Reten. Time [min]	Area [mV.s]	Height [mV]	Area [%]	Height [%]	W05 [min]
1	11.230	28550.717	761.679	97.3	97.0	0.59
2	14.730	806.484	23.509	2.7	3.0	0.52
	Total	29357.200	785.188	100.0	100.0	

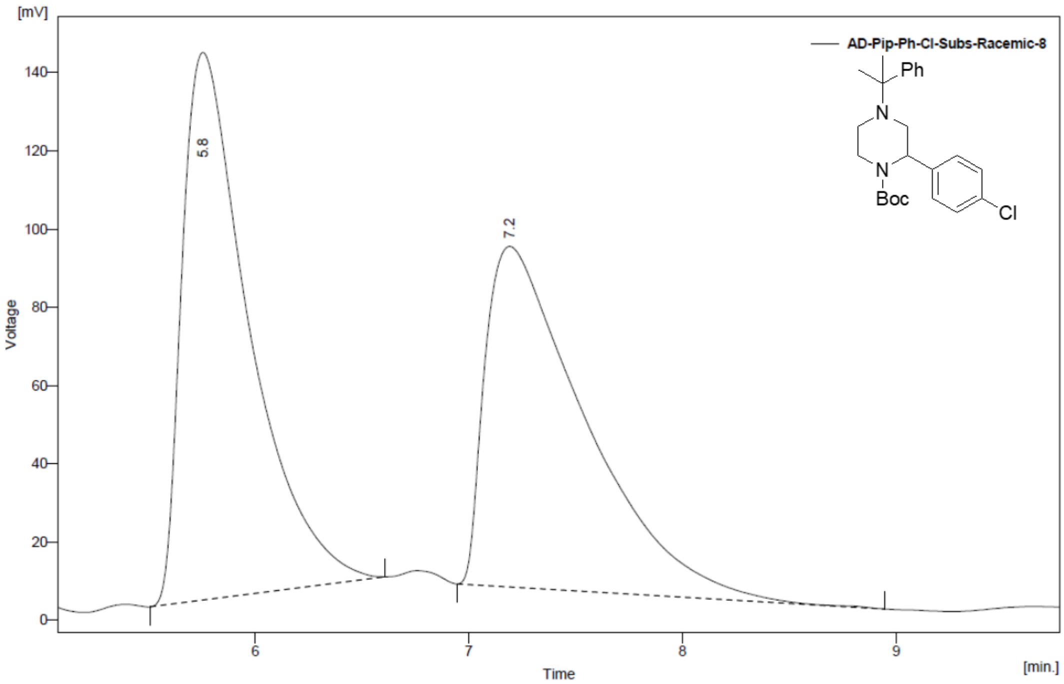

Result Table - Calculation Method Uncal

	Reten. Time [min]	Area [mV.s]	Height [mV]	Area [%]	Height [%]	W05 [min]
1	5.753	3054.674	139.986	51.7	61.7	0.33
2	7.190	2848.330	87.070	48.3	38.3	0.49
	Total	5903.004	227.056	100.0	100.0	

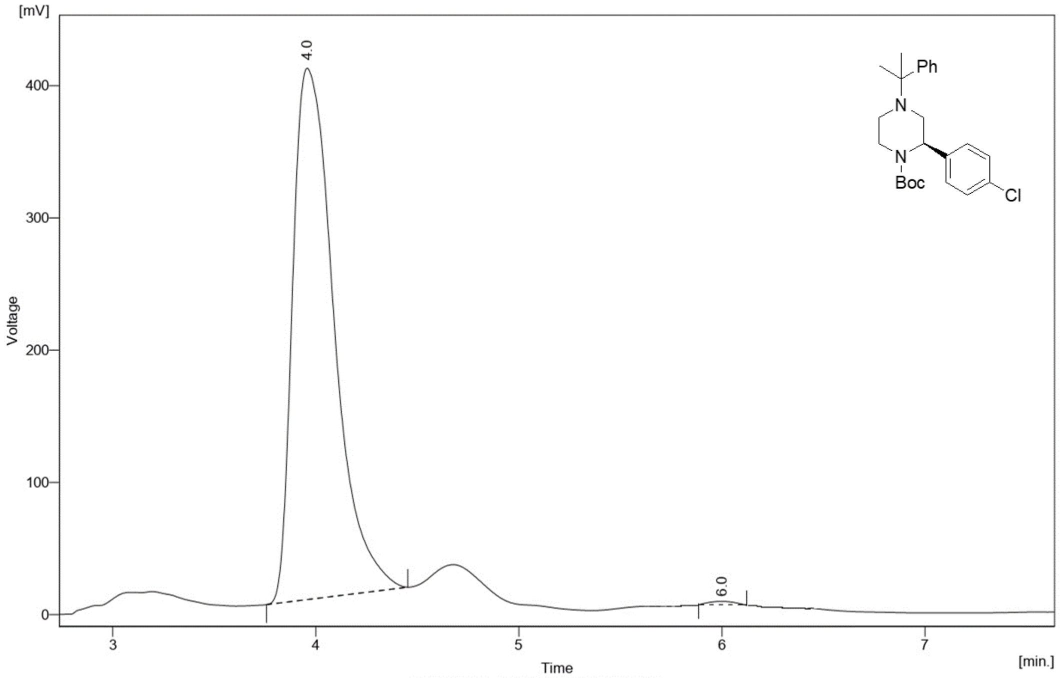

Result Table - Calculation Method Uncal

	Reten. Time [min]	Area [mV.s]	Height [mV]	Area [%]	Height [%]	W05 [min]
1	3.957	5681.100	401.929	98.7	97.1	0.22
2	6.023	257.636	11.980	1.3	2.9	0.37
	Total	5938.736	413.909	100.0	100.0	

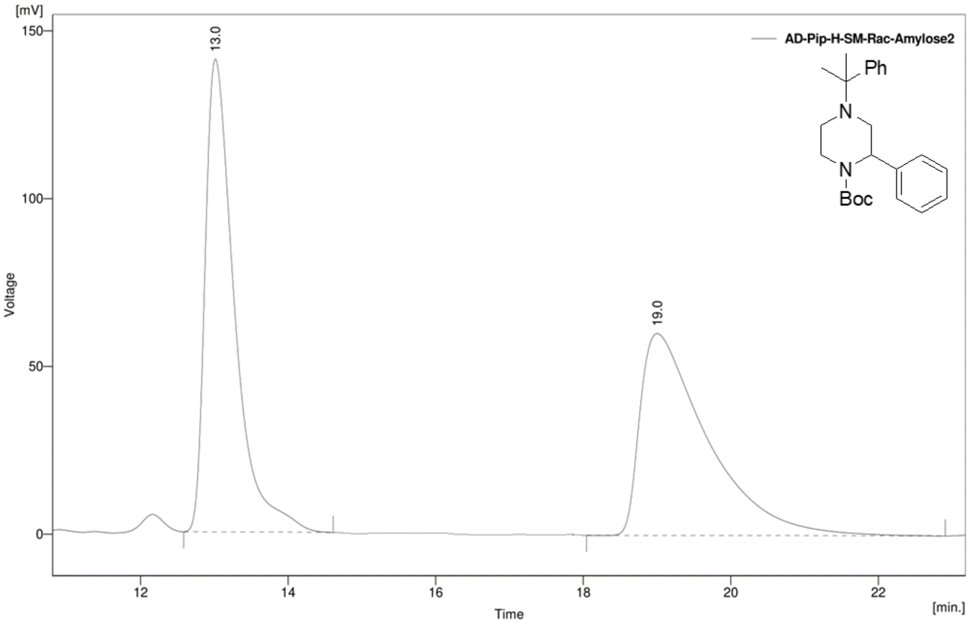

Result Table - Calculation Method Uncal

	Reten. Time [min]	Area [mV.s]	Height [mV]	Area [%]	Height [%]	W05 [min]
1	13.017	3893.827	140.937	50.5	70.1	0.40
2	19.003	3813.256	60.227	49.5	29.9	0.92
	Total	7707.083	201.164	100.0	100.0	

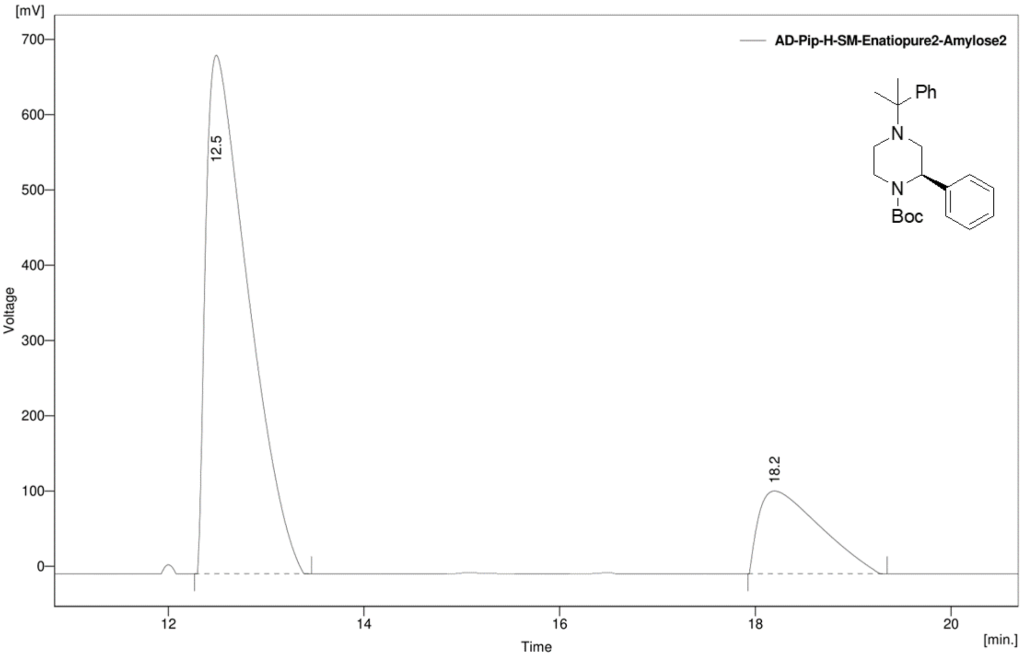

Result Table - Calculation Method Uncal

	Reten. Time [min]	Area [mV.s]	Height [mV]	Area [%]	Height [%]	W05 [min]
1	12.490	20727.952	689.025	81.2	86.2	0.48
2	18.197	4807.146	109.984	18.8	13.8	0.73
	Total	25535.098	799.010	100.0	100.0	

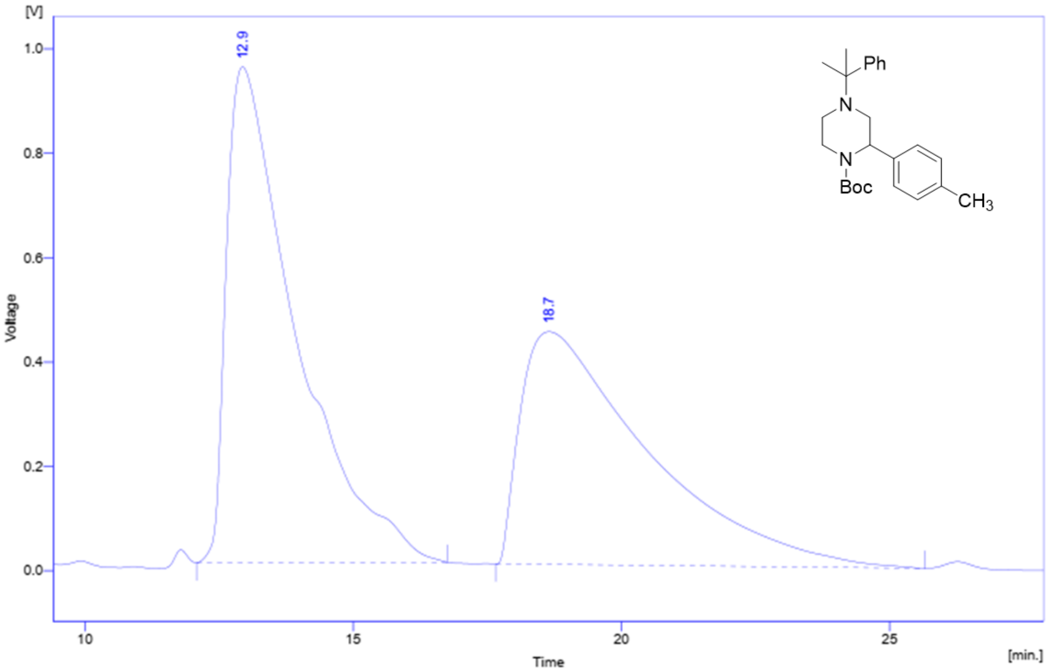

Result Table - Calculation Method Uncal

	Reten. Time [min]	Area [mV.s]	Height [mV]	Area [%]	Height [%]	W05 [min]
1	12.937	85247.406	949.959	53.7	68.0	1.26
2	18.650	73459.277	446.110	46.3	32.0	2.41
	Total	158706.683	1396.070	100.0	100.0	

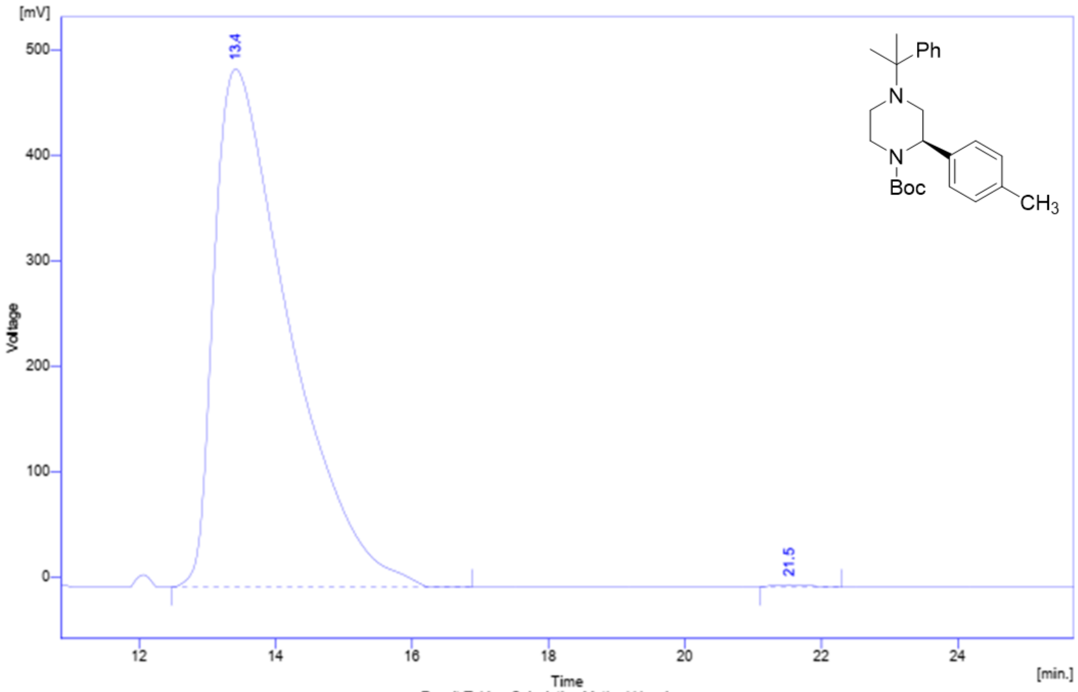

Result Table - Calculation Method Uncal

	Reten. Time [min]	Area [mV.s]	Height [mV]	Area [%]	Height [%]	W05 [min]
1	13.413	37601.857	491.686	99.7	99.5	1.15
2	21.527	103.556	2.660	0.3	0.5	0.69
	Total	37705.413	494.346	100.0	100.0	

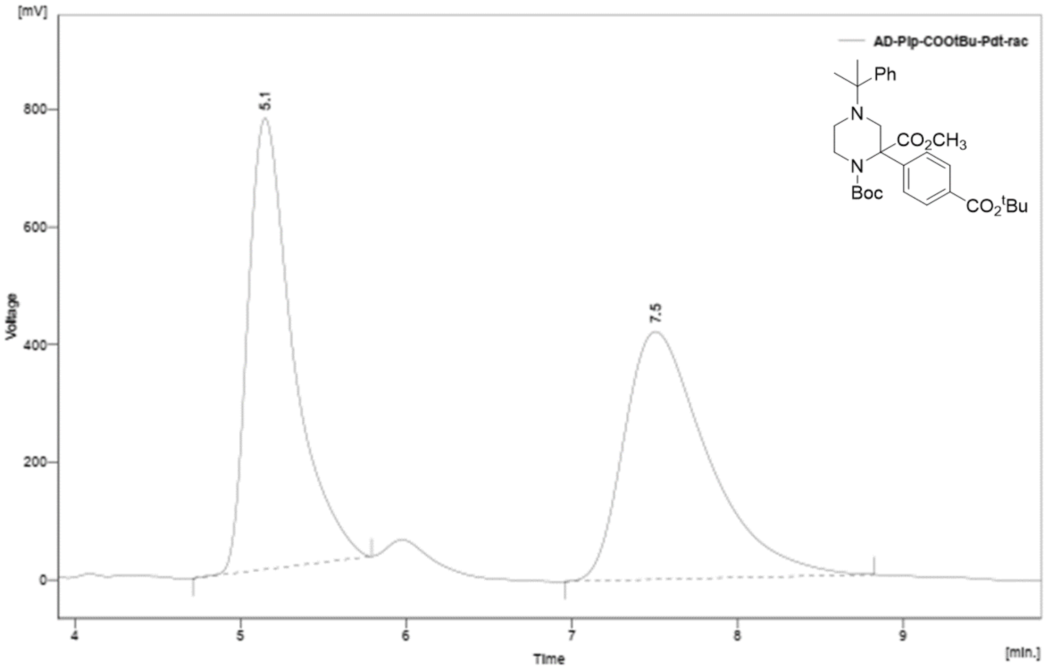

Result Table - Calculation Method Uncal

	Reten. Time [min]	Area [mV.s]	Height [mV]	Area [%]	Height [%]	W05 [min]
1	5.147	14555.523	767.520	49.6	64.6	0.28
2	7.503	14779.750	421.497	50.4	35.4	0.53
	Total	29335.273	1189.017	100.0	100.0	

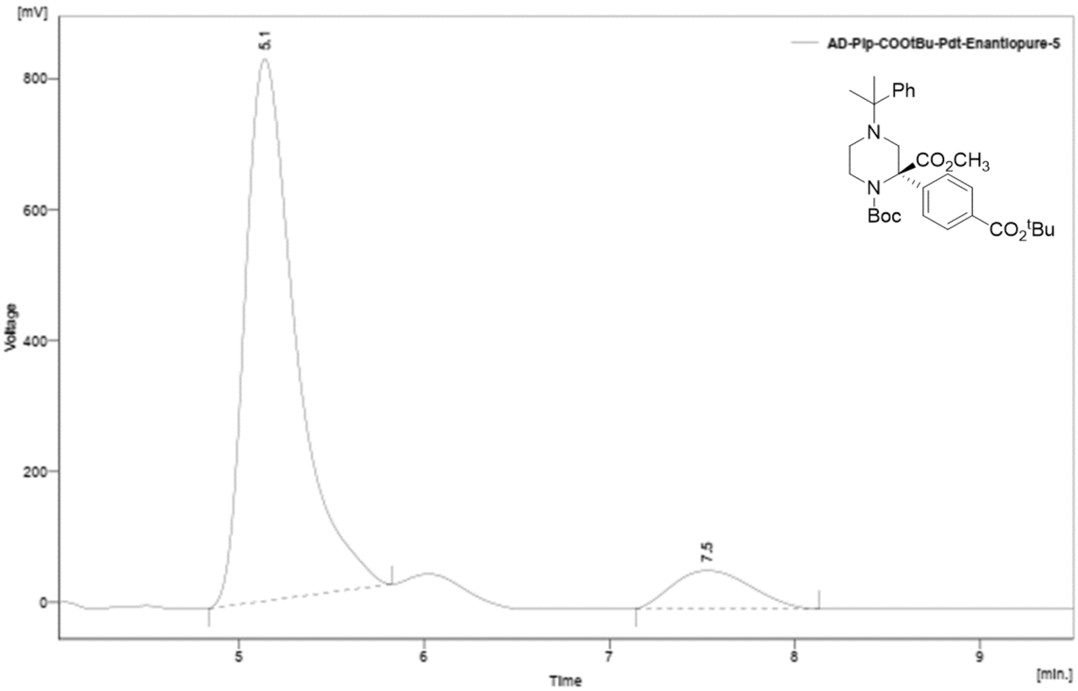

Result Table - Calculation Method Uncal

	Reten. Time [min]	Area [mV.s]	Height [mV]	Area [%]	Height [%]	W05 [min]
1	5.140	15816.978	829.848	90.3	93.4	0.28
2	7.527	1701.612	58.385	9.7	6.6	0.49
	Total	17518.590	888.233	100.0	100.0	

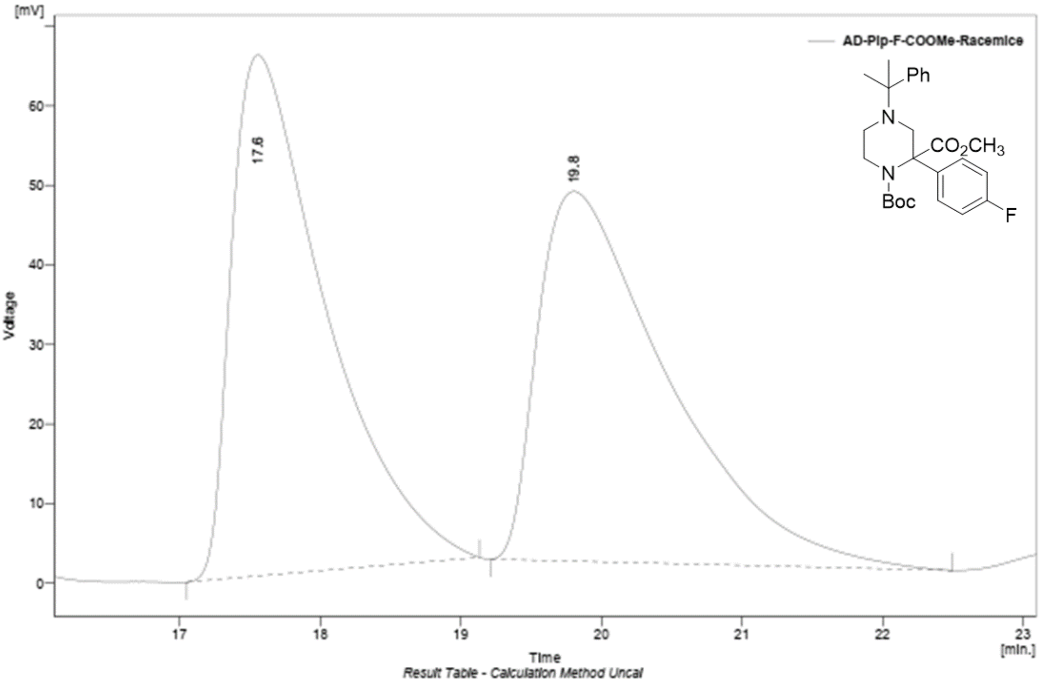

Result Table - Calculation Method Uncal

	Reten. Time [min]	Area [mV.s]	Height [mV]	Area [%]	Height [%]	W05 [min]
1	17.557	3055.515	65.492	50.5	58.5	0.72
2	19.803	2995.413	46.478	49.5	41.5	0.96
	Total	6050.928	111.970	100.0	100.0	

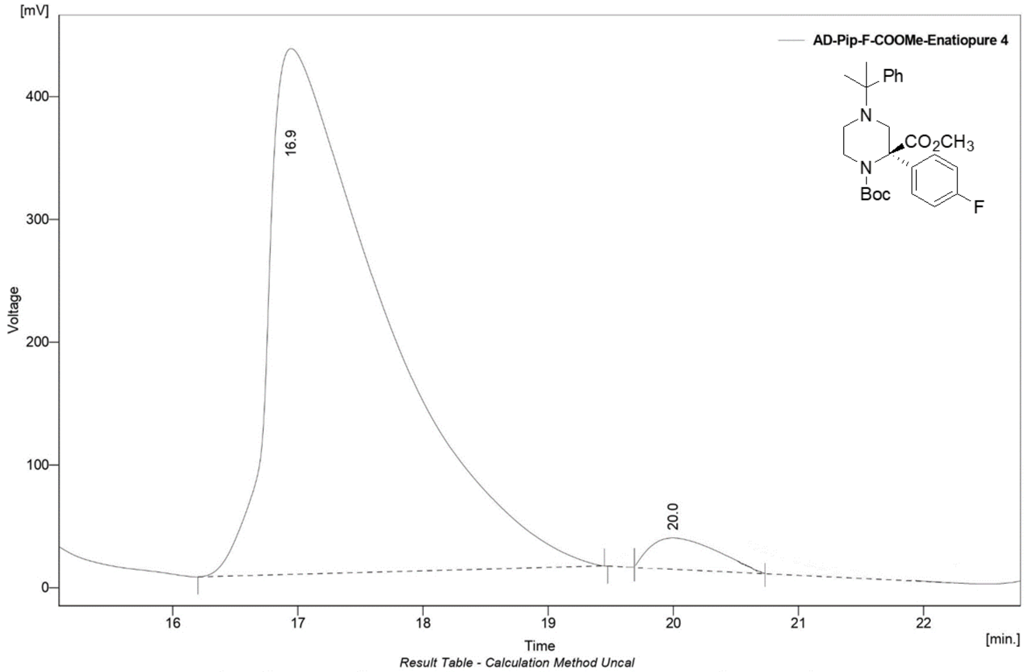

Result Table - Calculation Method Uncal

	Reten. Time [min]	Area [mV.s]	Height [mV]	Area [%]	Height [%]	W05 [min]
1	16.947	27626.956	428.153	97.3	90.8	0.94
2	20.000	2635.129	43.124	2.7	9.2	0.87
	Total	30262.085	471.277	100.0	100.0	

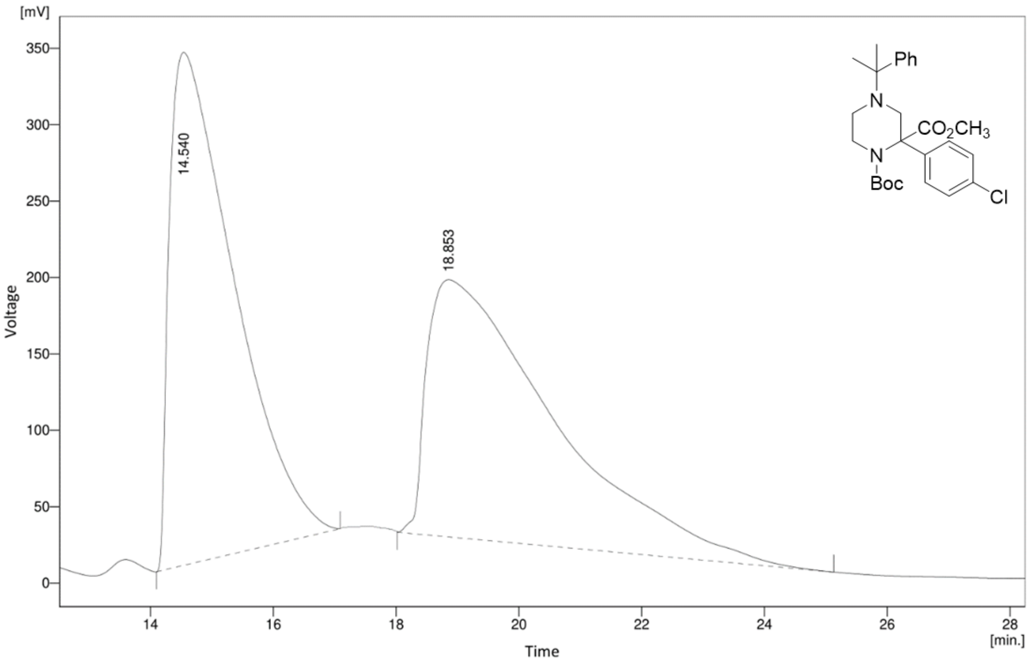

Result Table - Calculation Method Uncal

	Reten. Time [min]	Area [mV.s]	Height [mV]	Area [%]	Height [%]	W05 [min]
1	14.540	24603.020	335.749	50.4	66.6	1.20
2	18.853	24182.519	168.288	49.6	33.4	2.02
	Total	48785.539	504.037	100.0	100.0	

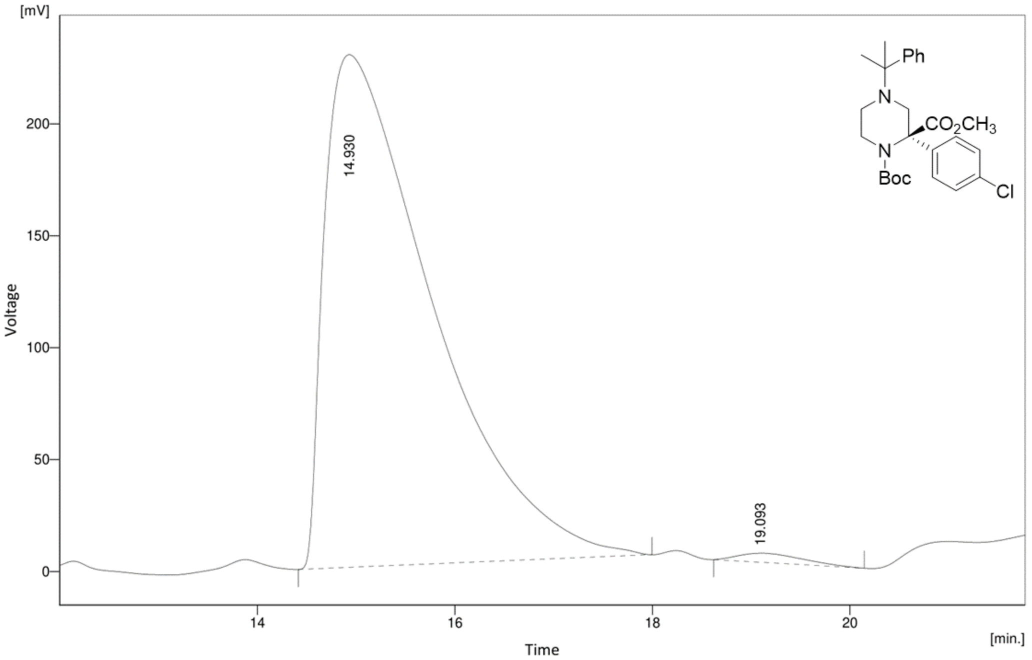

Result Table - Calculation Method Uncal

	Reten. Time [min]	Area [mV.s]	Height [mV]	Area [%]	Height [%]	W05 [min]
1	14.930	17384.240	229.065	98.9	98.3	1.17
2	19.093	188.612	4.032	1.1	1.7	0.69
	Total	17572.852	233.097	100.0	100.0	

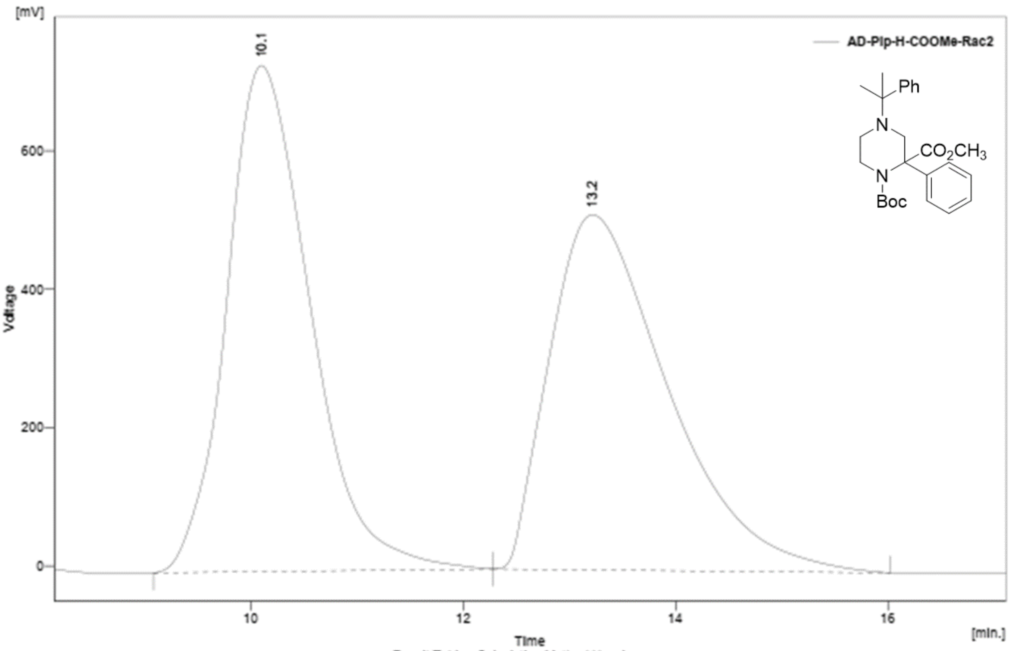

Result Table - Calculation Method Uncal

	Reten. Time [min]	Area [mV.s]	Height [mV]	Area [%]	Height [%]	W05 [min]
1	10.100	39960.034	731.340	50.1	58.8	0.82
2	13.217	39748.166	513.423	49.9	41.2	1.20
	Total	79708.200	1244.763	100.0	100.0	

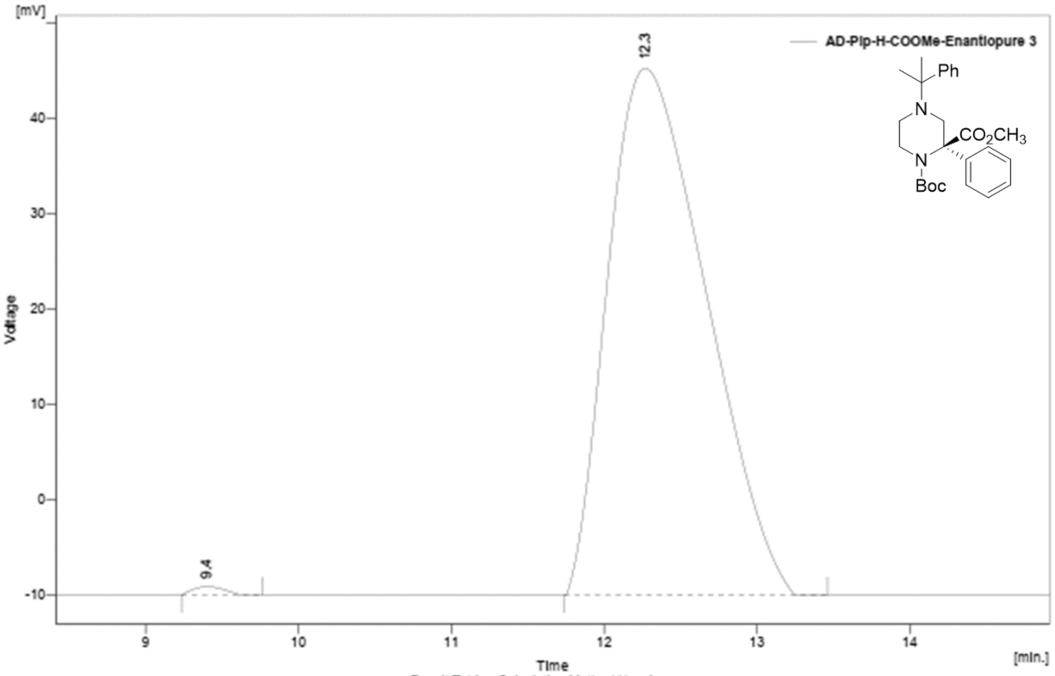

Result Table - Calculation Method Uncal

	Reten. Time [min]	Area [mV.s]	Height [mV]	Area [%]	Height [%]	W05 [min]
1	9.397	12.061	0.859	0.5	1.5	0.25
2	12.267	2457.651	55.180	99.5	98.5	0.73
	Total	2469.712	56.039	100.0	100.0	

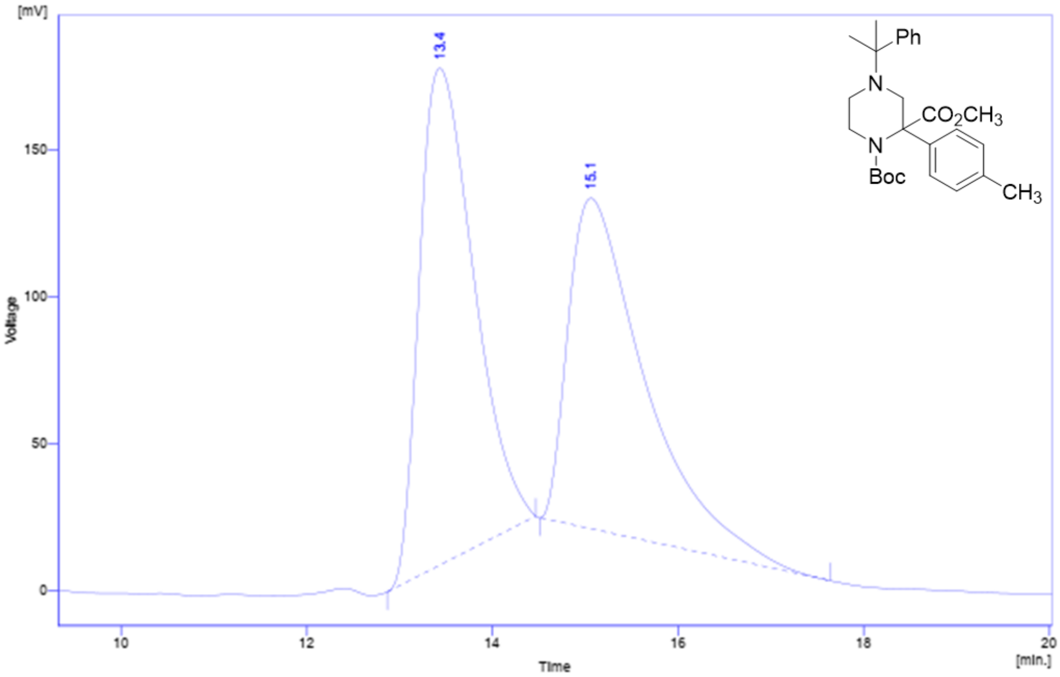

Result Table - Calculation Method Uncal

	Reten. Time [min]	Area [mV.s]	Height [mV]	Area [%]	Height [%]	W05 [min]
1	13.433	6905.849	169.036	51.5	60.0	0.66
2	15.060	6510.181	112.460	48.5	40.0	0.82
	Total	13416.030	281.496	100.0	100.0	

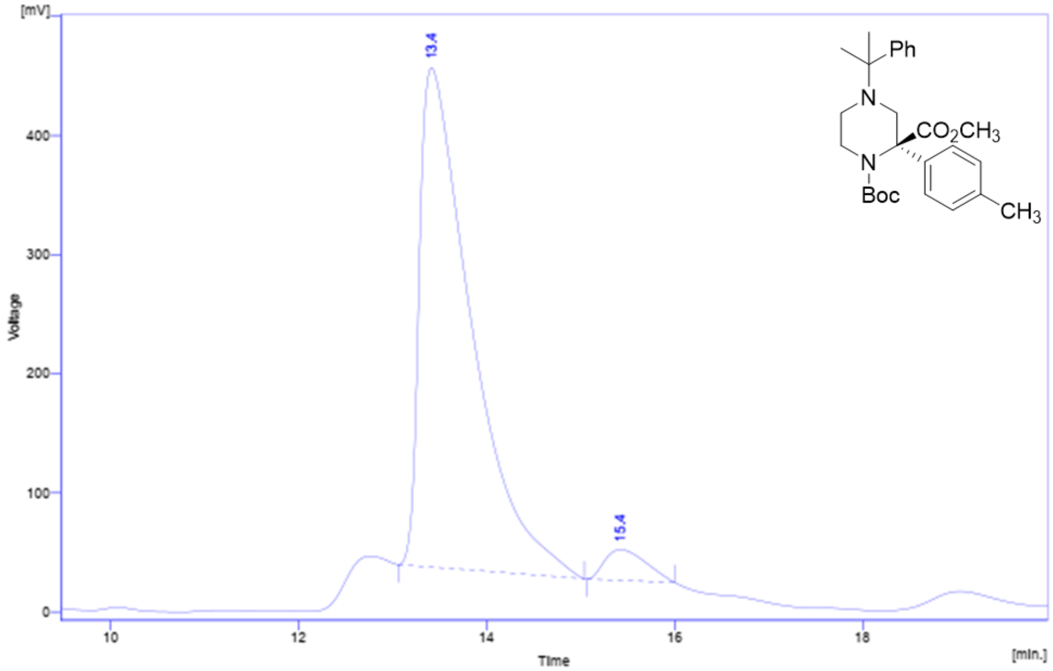

Result Table - Calculation Method Uncal

	Reten. Time [min]	Area [mV.s]	Height [mV]	Area [%]	Height [%]	W05 [min]
1	13.413	16074.727	419.222	95.3	94.2	0.56
2	15.423	790.206	25.835	4.7	5.8	0.50
	Total	16864.932	445.057	100.0	100.0	

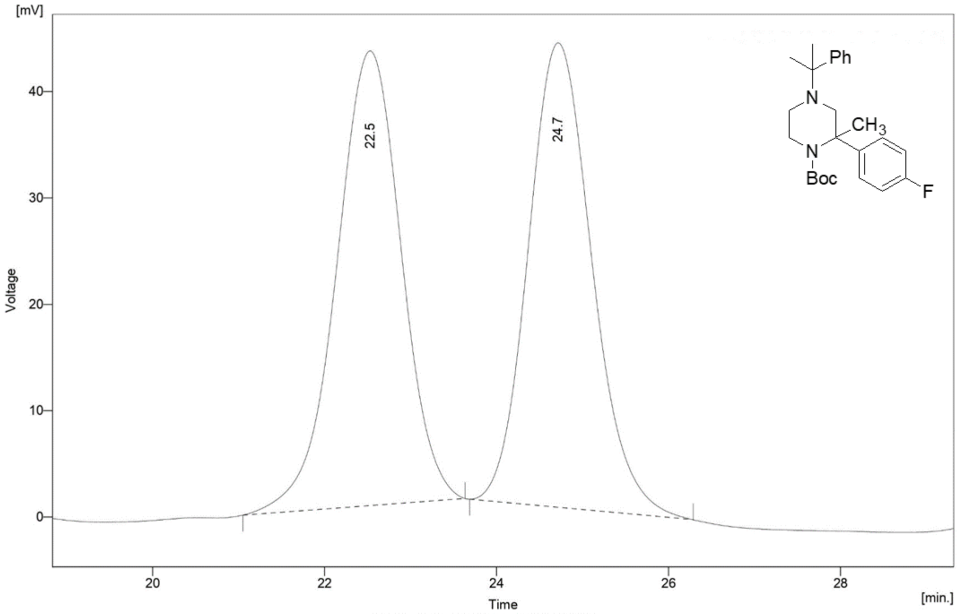

Result Table - Calculation Method Uncal

	Reten. Time [min]	Area [mV.s]	Height [mV]	Area [%]	Height [%]	W05 [min]
1	22.530	2236.886	42.764	49.9	49.5	0.81
2	24.713	2246.244	43.681	50.1	50.5	0.79
	Total	4483.130	86.445	100.0	100.0	

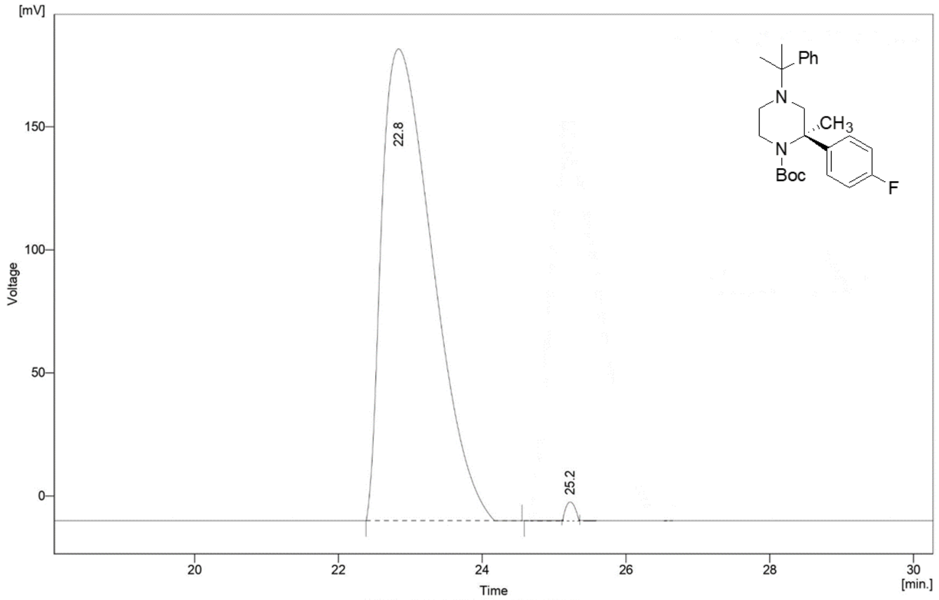

Result Table - Calculation Method Uncal

	Reten. Time [min]	Area [mV.s]	Height [mV]	Area [%]	Height [%]	W05 [min]
1	22.837	9419.386	191.550	96.0	91.5	0.79
2	25.207	7401.280	148.881	4.0	8.5	0.80
	Total	16820.666	340.431	100.0	100.0	

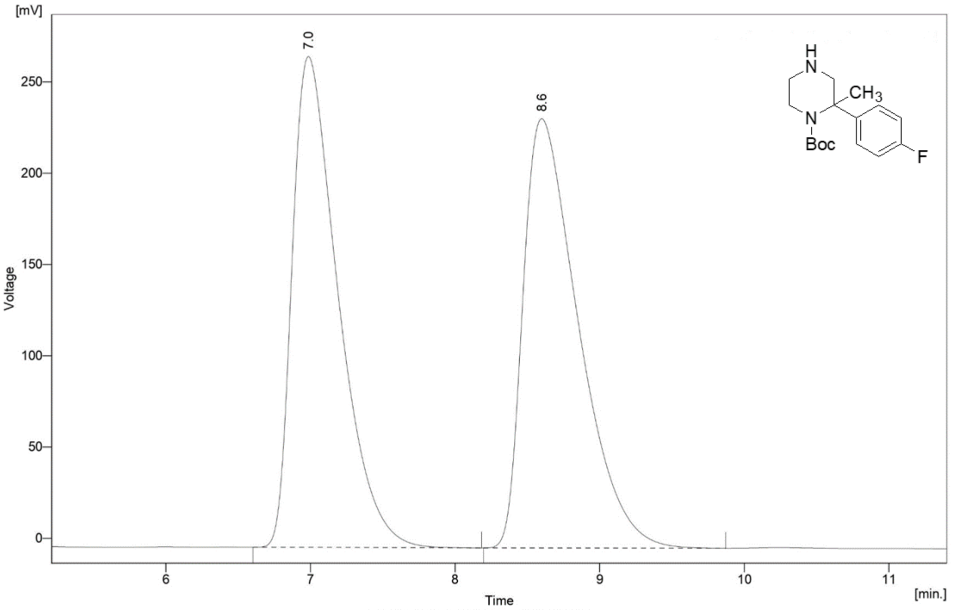

Result Table - Calculation Method Uncal

	Reten. Time [min]	Area [mV.s]	Height [mV]	Area [%]	Height [%]	W05 [min]
1	6.987	5935.376	268.932	48.8	53.3	0.34
2	8.600	6237.506	235.203	51.2	46.7	0.41
	Total	12172.883	504.135	100.0	100.0	

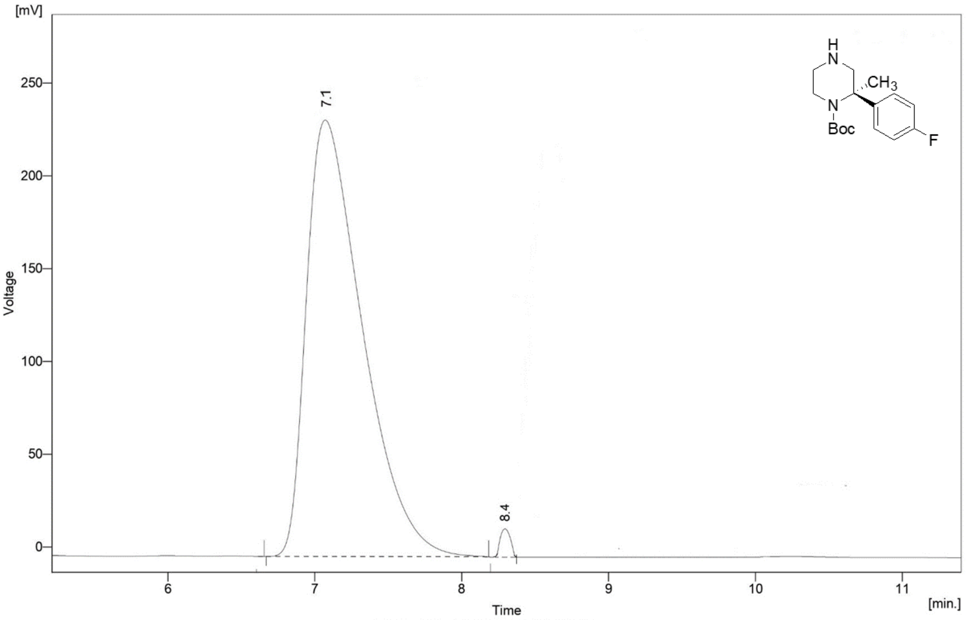

Result Table - Calculation Method Uncal

	Reten. Time [min]	Area [mV.s]	Height [mV]	Area [%]	Height [%]	W05 [min]
1	6.987	5935.376	268.932	93.8	93.1	0.34
2	8.600	6237.506	235.203	6.2	6.9	0.41
	Total	12172.883	504.135	100.0	100.0	

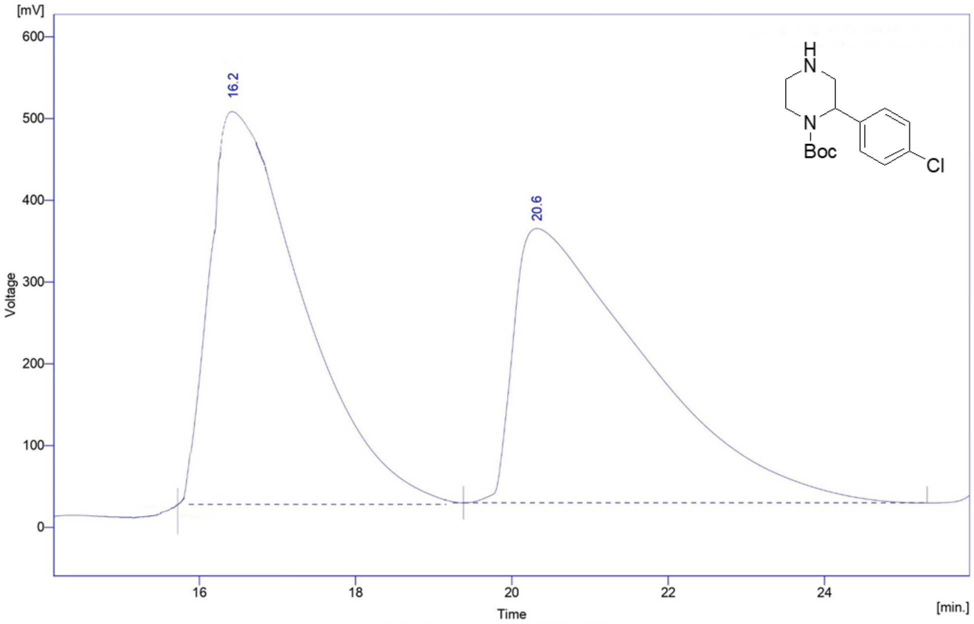

Result Table - Calculation Method Uncal

	Reten. Time [min]	Area [mV.s]	Height [mV]	Area [%]	Height [%]	W05 [min]
1	16.437	38848.456	545.558	49.6	61.9	1.07
2	20.323	39549.285	335.598	50.4	38.1	1.81
	Total	78397.742	881.156	100.0	100.0	

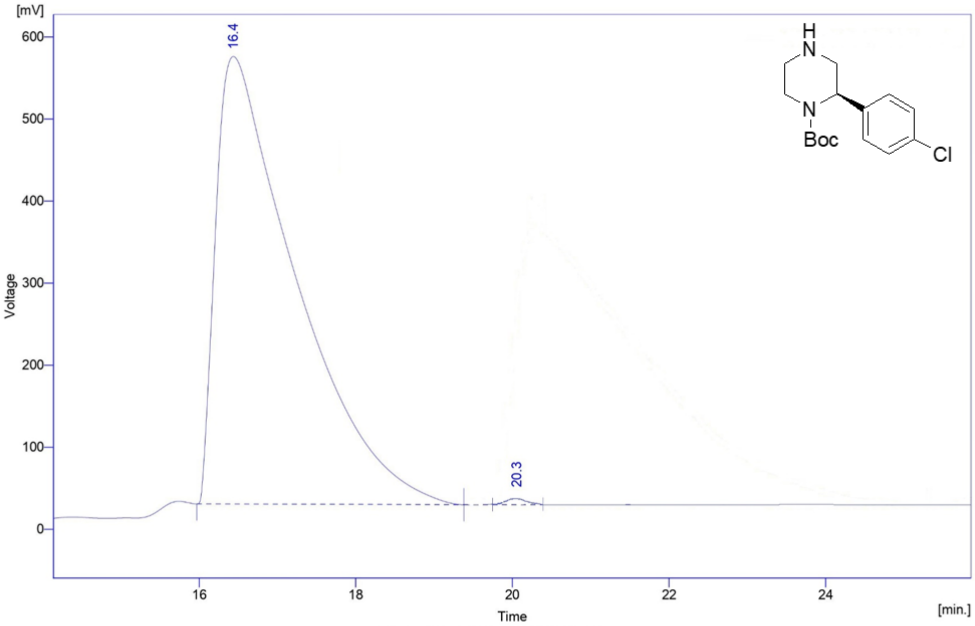

Result Table - Calculation Method Uncal

	Reten. Time [min]	Area [mV.s]	Height [mV]	Area [%]	Height [%]	W05 [min]
1	16.437	38848.456	545.558	98.6	97.7	1.07
2	20.323	39549.285	335.598	1.4	2.3	1.81
	Total	78397.742	881.156	100.0	100.0	

6. DFT calculations

All calculations on *tert*-butyl 2-phenyl-4-(2-phenylpropan-2-yl)piperazine-1-carboxylate **2g** were performed using density functional theory, employing the B3LYP[9] functional as implemented in the D.01 version of Gaussian 09.[10] Calculations included dispersion corrections using the GD3-BJ[11] method. Calculations used the def2TZVP[12] basis set. Solvent was included *via* the PCM method[13,14] as implemented in Gaussian with the default parameters for THF. Calculations were performed at 298 K and 195 K.

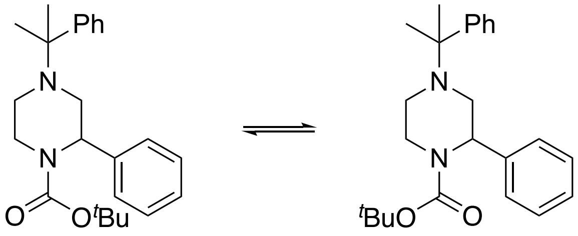

2g, 'left rotamer' **2g**, 'right rotamer'

The rotamers of piperazine **2g** were investigated, where the carbonyl of the Boc group could be pointing in the direction away from the phenyl group at C-2 [Left Rotamer] or towards the phenyl group at C-2 [Right Rotamer]. Comparing the axial and equatorial conformations the lower energy structures were found to be when the phenyl group at C-2 occupied an axial conformation.

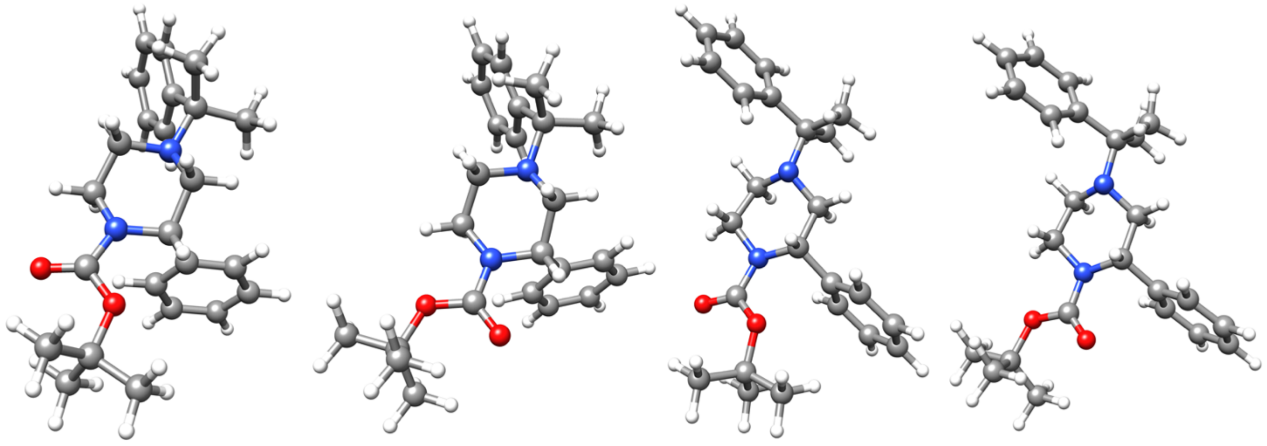

Phenyl Axial Phenyl Equatorial

Comparing the two rotamers, where the phenyl group at C-2 was axial, the lower energy rotamer was the left rotamer. At 195 K the difference in Gibbs energy between the two rotamers was calculated to be ~165 J/mol. This corresponded to a rotamer ratio of 53:47 in preference of the left rotamer.

The transition states for the clockwise and anticlockwise rotation of the Boc group were then investigated for both sets of rotamers with the different conformations of the C-2 phenyl group. The lower energy transitions states were found to be when the phenyl group adopted

an equatorial position. Closer analysis of the two transition states in which the phenyl group was equatorial found that the clockwise rotation of Boc group was lower in energy.

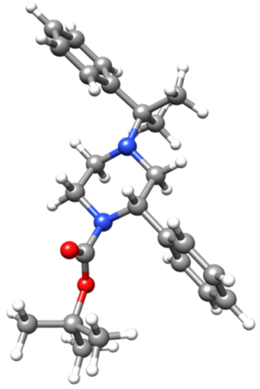

Phenyl Equatorial Clockwise Transition

The activation parameters for the Boc rotation at 195 K were calculated to give the following values:

$\Delta G^{\ddagger} \approx 50.2$ kJ/mol

$\Delta H^{\ddagger} \approx 46.1$ kJ/mol

$\Delta S^{\ddagger} \approx -20.9$ J/mol·K

The following pages show the DFT data for the rotamers and transition states:

Left Rotamer Axial

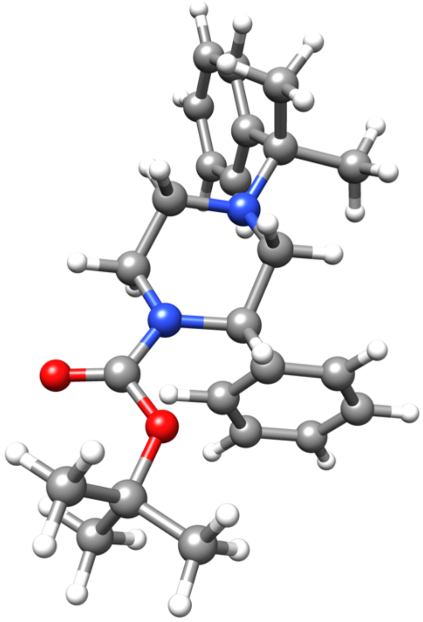

Route : # opt freq b3lyp scrf=(solvent=thf) geom=connectivity def2tzvp empiricaldispersion=gd3bj int=ultrafine pop=(regular,mk)

SMILES : CC(C)(C)OC(=O)N2CCN(C(C)(C)c1ccccc1)CC2c3ccccc3

Formula : $C_{24}H_{32}N_2O_2$

Charge : 0

Multiplicity : 1

Dipole : 3.4347 Debye

Energy : -1194.38643726 a.u.

Gibbs Energy : -1193.925156 a.u.

Number of imaginary frequencies : 0

Cartesian Coordinates (XYZ format)

60

C	-1.15452000	-1.70811700	-0.17176000
C	0.07325300	-1.38702600	-1.00242500
C	0.92574900	-0.03148300	0.91809400
C	-0.35595500	-0.44565800	1.65206000
H	0.38196400	-2.25255700	-1.58104200
H	-0.98063100	-2.63996800	0.39006100
H	-1.99249500	-1.87656900	-0.84587800
H	1.73941600	-0.11525900	1.63531100
H	-0.14506000	-1.38088800	2.19621500
H	-0.58715900	0.31371900	2.38967300
H	-0.17321200	-0.58101800	-1.70042200
N	1.19488700	-0.99211900	-0.15218000
N	-1.45092300	-0.59296700	0.71494400
C	-2.82745500	-0.57860300	1.26241800

C	-3.13009100	-1.83676500	2.09193900
H	-4.10148400	-1.76459400	2.58106600
H	-3.12366800	-2.74024200	1.48257700
H	-2.37845100	-1.94752000	2.87422900
C	-3.01551000	0.65718500	2.16140500
H	-2.64919200	1.55470800	1.66321700
H	-4.07633900	0.78823500	2.37229700
H	-2.50066000	0.54790000	3.11608600
C	-3.78129300	-0.40997200	0.07345500
C	-4.95717800	-1.14386100	-0.05981400
C	-3.49082200	0.55337600	-0.89688100
C	-5.81969500	-0.92633100	-1.13221100
H	-5.21470800	-1.89881100	0.66900400
C	-4.34675400	0.77311800	-1.96639500
H	-2.57190000	1.11666600	-0.81287200
C	-5.51890300	0.03177200	-2.09021900
H	-6.72575800	-1.51312500	-1.21618600
H	-4.09823300	1.52258300	-2.70743800
H	-6.18661900	0.19910700	-2.92556500
C	0.88496600	1.41357400	0.43242800
C	0.59873000	2.43629200	1.33866900
C	1.16990700	1.76320300	-0.88448700
C	0.57654300	3.76516300	0.93680600
H	0.39684000	2.19873500	2.37543700
C	1.14730800	3.09303300	-1.29337800
H	1.41678800	0.99816400	-1.60627000
C	0.84569000	4.10030200	-0.38631400
H	0.35116200	4.53992200	1.65858100
H	1.36775700	3.33826000	-2.32453200
H	0.82600700	5.13491700	-0.70319400
C	2.45936200	-1.26229500	-0.58677300
O	2.70843600	-1.92405400	-1.58171300
O	3.38138800	-0.71691800	0.22718900
C	4.81675900	-0.75786300	-0.08684100
C	5.31877100	-2.19708900	-0.08136800
H	4.89184800	-2.76571300	-0.90292400
H	6.40569300	-2.19913000	-0.17714300
H	5.05921500	-2.68316300	0.86041400
C	5.42738400	0.02912200	1.06615400
H	5.03446700	1.04608600	1.08483900
H	5.20115500	-0.45013900	2.01924100
H	6.51034000	0.07618500	0.95001700
C	5.07755300	-0.05225900	-1.41301900
H	6.15375900	0.04223600	-1.56442700
H	4.65461600	-0.60771100	-2.24594600
H	4.64540000	0.94943700	-1.39684900

Frequencies

Mode	IR frequency	IR intensity	Raman intensity
1	16.30880000	0.29740000	0.00000000
2	25.79900000	0.00770000	0.00000000
3	29.82410000	0.31890000	0.00000000
4	37.80720000	0.03400000	0.00000000
5	39.86080000	0.11870000	0.00000000

6	49.27010000	0.29750000	0.00000000
7	63.86680000	0.91880000	0.00000000
8	74.39300000	2.80820000	0.00000000
9	90.70150000	0.55650000	0.00000000
10	116.99960000	0.67460000	0.00000000
11	130.89640000	0.49700000	0.00000000
12	169.49480000	1.01940000	0.00000000
13	174.15550000	1.74810000	0.00000000
14	194.42220000	3.26720000	0.00000000
15	208.47960000	0.14250000	0.00000000
16	217.12330000	2.37210000	0.00000000
17	220.26140000	0.64380000	0.00000000
18	245.73340000	0.02960000	0.00000000
19	259.93110000	2.01910000	0.00000000
20	272.96660000	0.03060000	0.00000000
21	278.00360000	4.11230000	0.00000000
22	292.26400000	0.63660000	0.00000000
23	300.48920000	1.12720000	0.00000000
24	310.96420000	3.38280000	0.00000000
25	323.26530000	0.35820000	0.00000000
26	330.61400000	13.88790000	0.00000000
27	333.47500000	1.78160000	0.00000000
28	350.90830000	0.54130000	0.00000000
29	351.87350000	1.65790000	0.00000000
30	363.61550000	5.04180000	0.00000000
31	393.58140000	6.95390000	0.00000000
32	417.67330000	0.08020000	0.00000000
33	419.36900000	0.32000000	0.00000000
34	429.13510000	1.40610000	0.00000000
35	432.66730000	1.44180000	0.00000000
36	460.79780000	2.89580000	0.00000000
37	464.71770000	3.95650000	0.00000000
38	481.18490000	10.08600000	0.00000000
39	491.91100000	4.44620000	0.00000000
40	521.31930000	3.02790000	0.00000000
41	536.84600000	5.85710000	0.00000000
42	568.16510000	30.59950000	0.00000000
43	579.42210000	9.47590000	0.00000000
44	622.39310000	11.09740000	0.00000000
45	636.87140000	0.56490000	0.00000000
46	638.01980000	0.46440000	0.00000000
47	657.27070000	8.08750000	0.00000000
48	712.18000000	5.28360000	0.00000000

49	716.97880000	78.53800000	0.00000000
50	718.58490000	46.53950000	0.00000000
51	745.81010000	39.43620000	0.00000000
52	763.25570000	1.65960000	0.00000000
53	770.67880000	12.31020000	0.00000000
54	782.48470000	31.91240000	0.00000000
55	785.59280000	34.03320000	0.00000000
56	796.96150000	11.29090000	0.00000000
57	832.15170000	17.85820000	0.00000000
58	843.30540000	11.13050000	0.00000000
59	861.90540000	0.19740000	0.00000000
60	862.44570000	0.16390000	0.00000000
61	868.19080000	45.86870000	0.00000000
62	912.15630000	6.97080000	0.00000000
63	930.19830000	0.52880000	0.00000000
64	930.50230000	0.72510000	0.00000000
65	930.99830000	0.60770000	0.00000000
66	934.19200000	1.38470000	0.00000000
67	942.09240000	12.02050000	0.00000000
68	973.75160000	0.11490000	0.00000000
69	977.00050000	90.21600000	0.00000000
70	982.79800000	0.35460000	0.00000000
71	983.66680000	3.45800000	0.00000000
72	994.95710000	34.07800000	0.00000000
73	996.03740000	2.66520000	0.00000000
74	998.85740000	3.49790000	0.00000000
75	1005.22090000	34.62890000	0.00000000
76	1022.71870000	1.26740000	0.00000000
77	1024.98690000	0.45610000	0.00000000
78	1026.34320000	1.79220000	0.00000000
79	1032.12610000	27.76840000	0.00000000
80	1053.79550000	17.85250000	0.00000000
81	1054.26030000	1.03100000	0.00000000
82	1057.27960000	6.90040000	0.00000000
83	1061.13980000	0.80770000	0.00000000
84	1089.70620000	7.22210000	0.00000000
85	1101.70530000	12.61420000	0.00000000
86	1114.26190000	12.87060000	0.00000000
87	1124.47240000	72.15680000	0.00000000
88	1130.69810000	188.03120000	0.00000000
89	1135.97340000	74.85090000	0.00000000
90	1159.63200000	29.68620000	0.00000000
91	1180.33130000	0.57300000	0.00000000

92	1181.44020000	0.39270000	0.00000000
93	1190.79040000	272.87500000	0.00000000
94	1193.68780000	152.07030000	0.00000000
95	1198.84510000	40.46660000	0.00000000
96	1207.10040000	3.06820000	0.00000000
97	1211.04180000	5.89910000	0.00000000
98	1213.58130000	63.34760000	0.00000000
99	1244.96730000	9.85860000	0.00000000
100	1256.51310000	69.04100000	0.00000000
101	1269.28760000	27.47780000	0.00000000
102	1274.36490000	121.45790000	0.00000000
103	1282.86950000	10.70460000	0.00000000
104	1307.37340000	43.90840000	0.00000000
105	1316.05820000	84.61650000	0.00000000
106	1324.55040000	96.55410000	0.00000000
107	1330.38090000	2.80450000	0.00000000
108	1342.94210000	68.94960000	0.00000000
109	1360.03050000	7.03630000	0.00000000
110	1361.19740000	42.39390000	0.00000000
111	1367.73960000	71.07350000	0.00000000
112	1381.75540000	43.47390000	0.00000000
113	1397.29760000	1.09480000	0.00000000
114	1397.46110000	41.22850000	0.00000000
115	1399.90720000	29.83570000	0.00000000
116	1413.74370000	27.86810000	0.00000000
117	1415.49990000	8.40130000	0.00000000
118	1423.35370000	18.30050000	0.00000000
119	1429.91370000	109.18100000	0.00000000
120	1431.00630000	270.19010000	0.00000000
121	1467.22060000	0.39500000	0.00000000
122	1479.80000000	9.92900000	0.00000000
123	1484.24430000	16.99970000	0.00000000
124	1484.46450000	4.15170000	0.00000000
125	1484.47960000	2.22970000	0.00000000
126	1484.64660000	0.17620000	0.00000000
127	1488.02890000	21.28780000	0.00000000
128	1491.92390000	1.73370000	0.00000000
129	1493.44770000	0.55670000	0.00000000
130	1498.21010000	2.14590000	0.00000000
131	1499.33060000	13.67760000	0.00000000
132	1503.67800000	13.33500000	0.00000000
133	1508.57910000	11.12760000	0.00000000
134	1511.44100000	13.14400000	0.00000000

135	1517.25540000	28.75810000	0.00000000
136	1532.04750000	20.36240000	0.00000000
137	1535.37740000	17.51500000	0.00000000
138	1624.28450000	0.64580000	0.00000000
139	1626.99330000	1.62620000	0.00000000
140	1644.65520000	7.98700000	0.00000000
141	1647.07240000	13.91280000	0.00000000
142	1704.50350000	711.77600000	0.00000000
143	2925.01270000	92.96430000	0.00000000
144	2932.11190000	167.57250000	0.00000000
145	3021.30390000	55.94220000	0.00000000
146	3040.71810000	16.05450000	0.00000000
147	3042.24430000	27.17530000	0.00000000
148	3043.09440000	38.85820000	0.00000000
149	3047.35860000	28.78550000	0.00000000
150	3049.94840000	21.27390000	0.00000000
151	3099.07220000	8.93780000	0.00000000
152	3101.65940000	12.48270000	0.00000000
153	3103.89060000	19.97230000	0.00000000
154	3104.42600000	21.11650000	0.00000000
155	3105.14620000	3.96360000	0.00000000
156	3109.54260000	87.59150000	0.00000000
157	3111.03550000	45.73250000	0.00000000
158	3113.53060000	33.13710000	0.00000000
159	3117.00950000	66.30440000	0.00000000
160	3119.47370000	32.50760000	0.00000000
161	3138.23860000	14.49170000	0.00000000
162	3143.17840000	4.81680000	0.00000000
163	3147.68260000	21.43570000	0.00000000
164	3158.06920000	19.23890000	0.00000000
165	3166.72330000	0.48080000	0.00000000
166	3167.90390000	3.47830000	0.00000000
167	3174.37600000	1.21680000	0.00000000
168	3175.05020000	18.02930000	0.00000000
169	3182.99340000	37.25970000	0.00000000
170	3189.13660000	34.14940000	0.00000000
171	3194.00170000	32.77840000	0.00000000
172	3200.31450000	14.64760000	0.00000000
173	3204.39920000	11.68330000	0.00000000
174	3205.79690000	9.72200000	0.00000000

Right Rotamer Axial

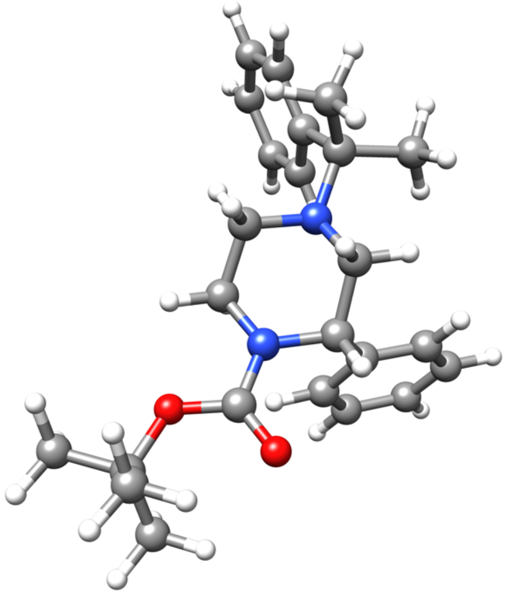

Route : # opt freq b3lyp scrf=(solvent=thf) geom=connectivity def2tzvp empiricaldispersion=gd3bj int=ultrafine pop=(regular,mk)
SMILES : CC(C)(C)OC(=O)N2CCN(C(C)(C)c1ccccc1)CC2c3ccccc3
Formula : $C_{24}H_{32}N_2O_2$
Charge : 0
Multiplicity : 1
Dipole : 2.9218 Debye
Energy : -1194.38576012 a.u.
Gibbs Energy : -1193.925528 a.u.
Number of imaginary frequencies : 0

Cartesian Coordinates (XYZ format)

60

C	0.65296000	-1.50775200	-0.34360600
C	-0.59771800	-1.04485800	0.38441400
C	-0.75140000	0.84411100	-1.23089400
C	0.49584800	0.25974100	-1.90309800
H	-1.17858700	-1.90055100	0.70949300
H	0.36906300	-2.23762300	-1.11911400
H	1.29978100	-2.01459800	0.37075400
H	-1.43853500	1.15357500	-2.01728300
H	0.16018700	-0.45398400	-2.67307300
H	1.02453000	1.05719100	-2.41107200
H	-0.30079000	-0.47384800	1.26894900
N	-1.42824700	-0.21681000	-0.48894800
N	1.34899000	-0.36317100	-0.91005300
C	2.76347400	-0.60980400	-1.27220100
C	2.90383400	-1.72845100	-2.31703500
H	3.93419700	-1.82341500	-2.65983200
H	2.58574000	-2.69523600	-1.92743500
H	2.29046600	-1.49296500	-3.18743900
C	3.38564600	0.67588800	-1.84697000

H	3.16056200	1.53084800	-1.20964100
H	4.46760000	0.55812300	-1.89643300
H	3.02989000	0.88346400	-2.85632800
C	3.51154000	-0.91824400	0.03071600
C	4.44275100	-1.94770300	0.14031100
C	3.29175900	-0.10494600	1.14634100
C	5.13549600	-2.16334400	1.32988400
H	4.63804200	-2.59841500	-0.69988800
C	3.97944400	-0.31615600	2.33264900
H	2.55831500	0.68657100	1.07794600
C	4.90725500	-1.34990800	2.43095600
H	5.85163700	-2.97309300	1.39148100
H	3.79004400	0.32540900	3.18420700
H	5.44284900	-1.51833900	3.35639500
C	-0.44439200	2.06595500	-0.36979500
C	0.32912000	3.10769800	-0.88522300
C	-0.95597000	2.20420100	0.91791000
C	0.60158500	4.24050600	-0.12961500
H	0.72294100	3.04480400	-1.89104200
C	-0.68465900	3.33587700	1.68023300
H	-1.57673500	1.42525900	1.33702600
C	0.10036200	4.35778100	1.16251800
H	1.20558400	5.03339200	-0.55219800
H	-1.09086800	3.41615900	2.68055900
H	0.31457800	5.23797900	1.75465600
C	-2.79078900	-0.23265600	-0.48060800
O	-3.48052000	0.51822000	-1.15229100
O	-3.26742200	-1.17637800	0.35275200
C	-4.71058100	-1.41039000	0.51378700
C	-5.38272300	-0.16591900	1.08286300
H	-5.36380300	0.65337800	0.36959000
H	-6.42057400	-0.39784100	1.32664000
H	-4.87996500	0.14833400	1.99888700
C	-4.74840000	-2.54902300	1.52569500
H	-4.23211200	-3.42658700	1.13516000
H	-4.26961000	-2.24875900	2.45828000
H	-5.78246700	-2.82094400	1.73845300
C	-5.32306900	-1.85058700	-0.81098900
H	-6.35764800	-2.15461700	-0.64506700
H	-5.30685400	-1.04491500	-1.53988900
H	-4.77736000	-2.70572600	-1.21286900

Frequencies

Mode	IR frequency	IR intensity	Raman intensity
1	13.90170000	0.09960000	0.00000000
2	20.16300000	0.09900000	0.00000000
3	26.60190000	0.67060000	0.00000000
4	36.78290000	0.03000000	0.00000000
5	37.30350000	0.23230000	0.00000000
6	44.37680000	0.19200000	0.00000000
7	59.33270000	0.94960000	0.00000000
8	68.25450000	3.10980000	0.00000000
9	96.17110000	0.92050000	0.00000000

10	117.21730000	0.50720000	0.00000000
11	129.34550000	1.48140000	0.00000000
12	166.80670000	1.99770000	0.00000000
13	175.18360000	2.58330000	0.00000000
14	198.03620000	1.05810000	0.00000000
15	204.97930000	0.16610000	0.00000000
16	215.54460000	0.92680000	0.00000000
17	224.23550000	1.71030000	0.00000000
18	245.26800000	0.29260000	0.00000000
19	264.18890000	1.89120000	0.00000000
20	270.21610000	3.72990000	0.00000000
21	272.71170000	0.66350000	0.00000000
22	282.90910000	1.27170000	0.00000000
23	292.04680000	1.24010000	0.00000000
24	305.37890000	0.57510000	0.00000000
25	322.27800000	1.13890000	0.00000000
26	330.60960000	11.13810000	0.00000000
27	338.07030000	5.92900000	0.00000000
28	349.07970000	2.12800000	0.00000000
29	353.51080000	0.75910000	0.00000000
30	357.20740000	2.66580000	0.00000000
31	411.49820000	2.94960000	0.00000000
32	417.13910000	0.14600000	0.00000000
33	418.52930000	0.56550000	0.00000000
34	425.32010000	1.58350000	0.00000000
35	446.42640000	9.51560000	0.00000000
36	460.56790000	2.84950000	0.00000000
37	462.60240000	4.51040000	0.00000000
38	477.11630000	7.78380000	0.00000000
39	491.15220000	4.43050000	0.00000000
40	524.19640000	3.43310000	0.00000000
41	557.57410000	32.64750000	0.00000000
42	561.44790000	2.28730000	0.00000000
43	581.38000000	10.29980000	0.00000000
44	623.07040000	12.71370000	0.00000000
45	636.35540000	4.10010000	0.00000000
46	638.15060000	0.71410000	0.00000000
47	642.90320000	6.80910000	0.00000000
48	705.89930000	1.76000000	0.00000000
49	715.88100000	78.42330000	0.00000000
50	716.54030000	44.29490000	0.00000000
51	744.50280000	37.64800000	0.00000000
52	747.89830000	8.53330000	0.00000000

53	779.57160000	40.13690000	0.00000000
54	780.98100000	19.12860000	0.00000000
55	782.67460000	12.45030000	0.00000000
56	795.94410000	12.70420000	0.00000000
57	830.68690000	4.98730000	0.00000000
58	841.51040000	3.77240000	0.00000000
59	860.39280000	0.35490000	0.00000000
60	861.83360000	0.19100000	0.00000000
61	871.53630000	65.80030000	0.00000000
62	911.37360000	5.81850000	0.00000000
63	928.66990000	1.46730000	0.00000000
64	930.53030000	0.06580000	0.00000000
65	931.05410000	0.45970000	0.00000000
66	933.97940000	1.62770000	0.00000000
67	941.57020000	9.38600000	0.00000000
68	972.15530000	0.09650000	0.00000000
69	976.60140000	102.78750000	0.00000000
70	981.39610000	8.87590000	0.00000000
71	982.35490000	1.20350000	0.00000000
72	992.57830000	4.94940000	0.00000000
73	993.72190000	19.15220000	0.00000000
74	997.32190000	7.65130000	0.00000000
75	1002.87590000	51.28740000	0.00000000
76	1022.39430000	0.31250000	0.00000000
77	1025.03560000	0.56730000	0.00000000
78	1026.14880000	0.24610000	0.00000000
79	1030.78070000	35.14670000	0.00000000
80	1052.85890000	7.92900000	0.00000000
81	1052.86820000	10.23190000	0.00000000
82	1057.55840000	10.24120000	0.00000000
83	1060.70920000	1.85680000	0.00000000
84	1087.36480000	9.68160000	0.00000000
85	1098.84870000	21.08310000	0.00000000
86	1113.26960000	9.38930000	0.00000000
87	1121.37970000	131.03510000	0.00000000
88	1129.76540000	64.70310000	0.00000000
89	1137.58850000	162.70590000	0.00000000
90	1158.32420000	17.93080000	0.00000000
91	1179.56890000	0.79500000	0.00000000
92	1181.00580000	2.59090000	0.00000000
93	1188.93140000	326.19020000	0.00000000
94	1195.38180000	56.78970000	0.00000000
95	1199.86290000	95.30260000	0.00000000

96	1204.32070000	1.41470000	0.00000000
97	1210.96080000	12.07050000	0.00000000
98	1213.35790000	51.93030000	0.00000000
99	1240.03810000	59.62940000	0.00000000
100	1260.28540000	52.55290000	0.00000000
101	1269.33850000	21.56970000	0.00000000
102	1277.64190000	72.88350000	0.00000000
103	1281.67850000	42.58560000	0.00000000
104	1298.24120000	98.27930000	0.00000000
105	1317.25600000	80.32080000	0.00000000
106	1323.25820000	60.42390000	0.00000000
107	1330.04500000	1.44150000	0.00000000
108	1341.89470000	84.05880000	0.00000000
109	1357.58820000	2.40190000	0.00000000
110	1362.02130000	26.24390000	0.00000000
111	1365.79250000	37.46880000	0.00000000
112	1384.46740000	30.32070000	0.00000000
113	1396.61780000	15.20090000	0.00000000
114	1396.86480000	47.01410000	0.00000000
115	1399.36330000	32.04350000	0.00000000
116	1408.54100000	42.93710000	0.00000000
117	1414.34660000	10.77030000	0.00000000
118	1423.23290000	21.64910000	0.00000000
119	1428.36150000	7.03960000	0.00000000
120	1437.17690000	422.86830000	0.00000000
121	1466.68400000	0.20920000	0.00000000
122	1479.71250000	11.62260000	0.00000000
123	1483.57560000	11.51540000	0.00000000
124	1484.02160000	2.54130000	0.00000000
125	1484.51030000	0.35950000	0.00000000
126	1484.74700000	3.28700000	0.00000000
127	1487.41660000	23.00520000	0.00000000
128	1491.65040000	1.00320000	0.00000000
129	1493.19840000	1.05020000	0.00000000
130	1497.61830000	7.13960000	0.00000000
131	1498.02250000	0.50670000	0.00000000
132	1503.08510000	2.80110000	0.00000000
133	1507.92590000	11.19420000	0.00000000
134	1512.04470000	17.19020000	0.00000000
135	1517.54040000	31.77450000	0.00000000
136	1530.80070000	19.76860000	0.00000000
137	1535.37590000	18.20990000	0.00000000
138	1624.08240000	0.59960000	0.00000000

139	1626.22880000	5.41230000	0.00000000
140	1644.21500000	7.82930000	0.00000000
141	1646.89200000	10.29060000	0.00000000
142	1702.69400000	678.36380000	0.00000000
143	2926.18330000	45.45510000	0.00000000
144	2929.91900000	218.51680000	0.00000000
145	3028.15440000	51.51550000	0.00000000
146	3040.95920000	16.05290000	0.00000000
147	3042.28530000	26.82380000	0.00000000
148	3043.23450000	39.88910000	0.00000000
149	3047.02350000	29.33560000	0.00000000
150	3049.98130000	21.87650000	0.00000000
151	3087.44510000	8.42560000	0.00000000
152	3100.39700000	22.75400000	0.00000000
153	3102.08360000	7.16040000	0.00000000
154	3103.56690000	28.81990000	0.00000000
155	3104.84060000	7.52810000	0.00000000
156	3109.87040000	81.77500000	0.00000000
157	3110.86980000	46.83640000	0.00000000
158	3113.03940000	33.97600000	0.00000000
159	3116.95020000	64.36850000	0.00000000
160	3119.97030000	32.20470000	0.00000000
161	3145.19750000	3.25890000	0.00000000
162	3148.77390000	22.28650000	0.00000000
163	3157.60400000	13.74950000	0.00000000
164	3160.90120000	18.08750000	0.00000000
165	3166.59560000	0.51960000	0.00000000
166	3168.67990000	1.96520000	0.00000000
167	3174.87400000	17.13660000	0.00000000
168	3175.65590000	2.96630000	0.00000000
169	3185.52330000	37.41940000	0.00000000
170	3188.78550000	32.79100000	0.00000000
171	3194.05930000	35.47900000	0.00000000
172	3198.13090000	15.83480000	0.00000000
173	3202.88250000	10.38690000	0.00000000
174	3204.04640000	13.16510000	0.00000000

Left Rotamer Equatorial

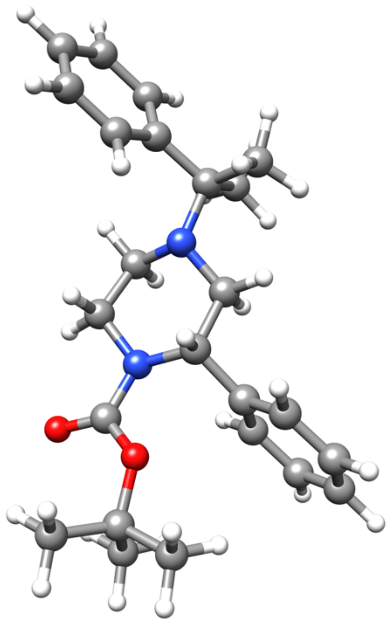

Route : # opt freq b3lyp scrf=(solvent=thf) geom=connectivity def2tzvp
empiricaldispersion=gd3bj int=ultrafine pop=(regular,mk)
SMILES : CC(C)(C)OC(=O)N2CCN(C(C)(C)c1ccccc1)CC2c3ccccc3
Formula : $C_{24}H_{32}N_2O_2$
Charge : 0
Multiplicity : 1
Dipole : 3.3178 Debye
Energy : -1194.38233605 a.u.
Gibbs Energy : -1193.920928 a.u.
Number of imaginary frequencies : 0

Cartesian Coordinates (XYZ format)

60

C	0.59771200	1.23472000	0.03045200
C	-0.64160200	0.40258200	-0.34747700
C	0.53809900	-1.49776800	0.79460700
C	1.64894000	-0.53527900	1.14461600
H	0.39469300	1.72272700	0.99694500
H	0.67842000	2.01761900	-0.71795400
H	0.35161800	-2.17547800	1.62239000
H	1.42572200	-0.04790500	2.10699600
H	2.56830800	-1.10555300	1.26563600
H	-0.54160900	0.11342300	-1.39921700
N	-0.72320700	-0.81010000	0.48366100
N	1.80441000	0.43621200	0.07275600
C	3.09163000	1.17839300	0.09251300
C	3.14420800	2.15131100	-1.09935100
H	4.16031300	2.52882500	-1.20960800
H	2.86354600	1.64499300	-2.02294200
H	2.48736200	3.00867800	-0.95293000
C	3.27295500	1.98656300	1.38742600
H	3.34992500	1.34482700	2.26475300

H	4.16845500	2.60590300	1.34107200
H	2.42336000	2.65500000	1.52692800
C	4.20979300	0.15040800	-0.11906200
C	4.07902100	-0.79807400	-1.13722700
C	5.38238400	0.14809000	0.63258700
C	5.08502900	-1.71831200	-1.39421800
H	3.16653700	-0.81900300	-1.71636600
C	6.39563900	-0.77348800	0.37789400
H	5.52088100	0.86289600	1.43065900
C	6.25240200	-1.71063200	-0.63558700
H	4.95796700	-2.44532900	-2.18658300
H	7.29582900	-0.75655100	0.97925300
H	7.03771900	-2.42908800	-0.83221600
C	-1.82084900	-1.63838500	0.44917900
O	-1.88167000	-2.69115800	1.06253400
O	-2.78831900	-1.15655200	-0.33967700
C	-4.16940600	-1.66247500	-0.28408300
C	-4.22590400	-3.08194100	-0.83524600
H	-3.69564800	-3.77519900	-0.18791700
H	-5.26776400	-3.39676800	-0.91419600
H	-3.78290300	-3.11688300	-1.83177700
C	-4.91352400	-0.69529400	-1.19575500
H	-4.83866900	0.32288700	-0.81625200
H	-4.49533100	-0.72059900	-2.20249100
H	-5.96567500	-0.97647800	-1.24982000
C	-4.70110200	-1.56708200	1.14128300
H	-5.76257400	-1.81850200	1.14409400
H	-4.18011500	-2.25133900	1.80623800
H	-4.58909200	-0.54888600	1.51543600
H	0.84973100	-2.09572900	-0.06999400
C	-1.83305800	1.32587300	-0.20635700
C	-2.38974600	1.60699700	1.03952100
C	-2.32923500	1.98198400	-1.32777100
C	-3.44078000	2.50682400	1.15608200
H	-2.01300800	1.09901300	1.91775000
C	-3.37464200	2.89173500	-1.21461200
H	-1.90578300	1.76632600	-2.30145800
C	-3.93837000	3.15354200	0.02844500
H	-3.87355300	2.70391900	2.12871500
H	-3.75536100	3.38646300	-2.09904000
H	-4.75937800	3.85291900	0.11896600

Frequencies

Mode	IR frequency	IR intensity	Raman intensity
1	19.90680000	0.18610000	0.00000000
2	25.90740000	0.32400000	0.00000000
3	37.16180000	0.16090000	0.00000000
4	38.39810000	0.21420000	0.00000000
5	51.89530000	2.18110000	0.00000000
6	62.96650000	0.31220000	0.00000000
7	70.42930000	0.74730000	0.00000000
8	78.56530000	2.53890000	0.00000000
9	96.83610000	1.00950000	0.00000000

10	101.04830000	0.57430000	0.00000000
11	137.86720000	0.98540000	0.00000000
12	146.61180000	1.53230000	0.00000000
13	172.89550000	3.07800000	0.00000000
14	190.25080000	2.02510000	0.00000000
15	205.76000000	0.26180000	0.00000000
16	213.31230000	0.66210000	0.00000000
17	233.49250000	3.46830000	0.00000000
18	244.66450000	2.08100000	0.00000000
19	248.24270000	0.58250000	0.00000000
20	258.45610000	0.69050000	0.00000000
21	271.51140000	5.02430000	0.00000000
22	276.71180000	0.83250000	0.00000000
23	285.20590000	1.31630000	0.00000000
24	300.05600000	0.24790000	0.00000000
25	310.51240000	2.60850000	0.00000000
26	325.78780000	1.41640000	0.00000000
27	328.43500000	6.92560000	0.00000000
28	337.59100000	5.51160000	0.00000000
29	353.84440000	1.93590000	0.00000000
30	362.54530000	2.96380000	0.00000000
31	384.91020000	10.33900000	0.00000000
32	417.79230000	0.07850000	0.00000000
33	418.67750000	0.29280000	0.00000000
34	419.50130000	0.45530000	0.00000000
35	431.29950000	5.08280000	0.00000000
36	462.17180000	2.38120000	0.00000000
37	465.78320000	4.50740000	0.00000000
38	483.58090000	3.11390000	0.00000000
39	500.52000000	0.99420000	0.00000000
40	522.50320000	4.78480000	0.00000000
41	541.44360000	7.72770000	0.00000000
42	566.87930000	10.92890000	0.00000000
43	585.31100000	26.60070000	0.00000000
44	615.75770000	25.98910000	0.00000000
45	636.39940000	0.00940000	0.00000000
46	638.03910000	0.50250000	0.00000000
47	648.59520000	0.43360000	0.00000000
48	698.62500000	2.05590000	0.00000000
49	716.81810000	63.45290000	0.00000000
50	718.09460000	83.64480000	0.00000000
51	735.77830000	2.09900000	0.00000000
52	762.77450000	13.81810000	0.00000000

53	777.34650000	12.92350000	0.00000000
54	787.00300000	46.06650000	0.00000000
55	794.05120000	54.94700000	0.00000000
56	815.35260000	16.41550000	0.00000000
57	835.90350000	3.27920000	0.00000000
58	860.88310000	29.31870000	0.00000000
59	861.38450000	1.13000000	0.00000000
60	862.55280000	0.45410000	0.00000000
61	863.56900000	22.04470000	0.00000000
62	913.17260000	4.66780000	0.00000000
63	930.75380000	2.38420000	0.00000000
64	931.18130000	5.29110000	0.00000000
65	931.82950000	1.18060000	0.00000000
66	933.44330000	0.88850000	0.00000000
67	942.30380000	11.67210000	0.00000000
68	949.63670000	7.52040000	0.00000000
69	973.37530000	0.20360000	0.00000000
70	983.16750000	0.13380000	0.00000000
71	985.07970000	0.27710000	0.00000000
72	994.92700000	88.32180000	0.00000000
73	998.69250000	2.00740000	0.00000000
74	1001.41940000	6.00830000	0.00000000
75	1021.84050000	3.62810000	0.00000000
76	1023.05550000	22.69370000	0.00000000
77	1025.71980000	0.73560000	0.00000000
78	1026.63230000	7.59220000	0.00000000
79	1052.54450000	4.63030000	0.00000000
80	1053.45730000	24.20150000	0.00000000
81	1053.69340000	2.73270000	0.00000000
82	1057.36360000	3.82430000	0.00000000
83	1068.48980000	3.73740000	0.00000000
84	1088.38000000	11.09800000	0.00000000
85	1101.25070000	10.81690000	0.00000000
86	1104.51080000	13.59910000	0.00000000
87	1124.05510000	4.30150000	0.00000000
88	1136.76460000	12.57990000	0.00000000
89	1143.79050000	256.66590000	0.00000000
90	1165.43750000	18.29450000	0.00000000
91	1179.23490000	2.01120000	0.00000000
92	1180.33870000	1.21110000	0.00000000
93	1184.76190000	51.77040000	0.00000000
94	1189.29250000	424.20090000	0.00000000
95	1200.48130000	9.06760000	0.00000000

96	1202.67330000	98.71260000	0.00000000
97	1206.99020000	3.04740000	0.00000000
98	1222.26170000	15.65790000	0.00000000
99	1238.56180000	24.42690000	0.00000000
100	1258.62840000	94.35770000	0.00000000
101	1270.46490000	22.32610000	0.00000000
102	1277.13390000	15.19820000	0.00000000
103	1281.64380000	57.71320000	0.00000000
104	1290.43410000	11.73390000	0.00000000
105	1306.92280000	268.01650000	0.00000000
106	1329.99110000	1.25180000	0.00000000
107	1331.03390000	37.67280000	0.00000000
108	1348.74090000	41.11430000	0.00000000
109	1354.99330000	77.31280000	0.00000000
110	1359.64770000	5.21830000	0.00000000
111	1367.14100000	126.43170000	0.00000000
112	1388.53590000	31.21200000	0.00000000
113	1396.48410000	26.53530000	0.00000000
114	1397.71440000	13.93140000	0.00000000
115	1400.31000000	45.87360000	0.00000000
116	1402.31280000	71.95190000	0.00000000
117	1415.12380000	22.65040000	0.00000000
118	1424.50180000	5.86760000	0.00000000
119	1427.05650000	118.61650000	0.00000000
120	1431.71610000	15.57300000	0.00000000
121	1467.14730000	0.36380000	0.00000000
122	1479.52220000	10.97100000	0.00000000
123	1484.18940000	3.98250000	0.00000000
124	1484.62530000	4.84450000	0.00000000
125	1485.61030000	0.64640000	0.00000000
126	1488.44990000	24.59300000	0.00000000
127	1490.22120000	1.58530000	0.00000000
128	1491.44270000	8.21910000	0.00000000
129	1494.05980000	0.13250000	0.00000000
130	1497.27450000	2.68690000	0.00000000
131	1498.22610000	5.35980000	0.00000000
132	1503.46060000	10.54520000	0.00000000
133	1508.82800000	11.08470000	0.00000000
134	1510.16910000	7.88190000	0.00000000
135	1518.25880000	23.34740000	0.00000000
136	1531.85850000	24.64210000	0.00000000
137	1533.11410000	14.74740000	0.00000000
138	1624.30420000	0.86050000	0.00000000

139	1629.05920000	1.48490000	0.00000000
140	1644.85420000	10.19930000	0.00000000
141	1648.00250000	13.91620000	0.00000000
142	1701.87980000	686.56200000	0.00000000
143	2933.87130000	36.18600000	0.00000000
144	2937.94310000	133.93720000	0.00000000
145	2998.26360000	74.86770000	0.00000000
146	3006.86900000	67.75680000	0.00000000
147	3042.46490000	18.54140000	0.00000000
148	3043.32770000	27.00580000	0.00000000
149	3044.84690000	31.15250000	0.00000000
150	3049.54830000	24.13960000	0.00000000
151	3051.71870000	27.31430000	0.00000000
152	3101.38450000	16.28720000	0.00000000
153	3103.52890000	23.58000000	0.00000000
154	3107.02950000	3.15790000	0.00000000
155	3108.05010000	15.33130000	0.00000000
156	3111.44150000	81.67150000	0.00000000
157	3113.72320000	62.59960000	0.00000000
158	3115.21130000	36.66760000	0.00000000
159	3117.92320000	34.65400000	0.00000000
160	3126.11390000	26.97640000	0.00000000
161	3133.05710000	26.53190000	0.00000000
162	3133.92880000	16.34810000	0.00000000
163	3141.12140000	6.65130000	0.00000000
164	3146.08190000	20.54590000	0.00000000
165	3162.69060000	8.85820000	0.00000000
166	3167.27780000	0.70440000	0.00000000
167	3169.63330000	0.00660000	0.00000000
168	3175.56470000	17.73040000	0.00000000
169	3178.78100000	19.35970000	0.00000000
170	3186.47530000	34.99630000	0.00000000
171	3189.78420000	36.12560000	0.00000000
172	3195.33280000	19.68930000	0.00000000
173	3200.63080000	14.37230000	0.00000000
174	3207.21090000	11.80830000	0.00000000

Right Rotamer Equatorial

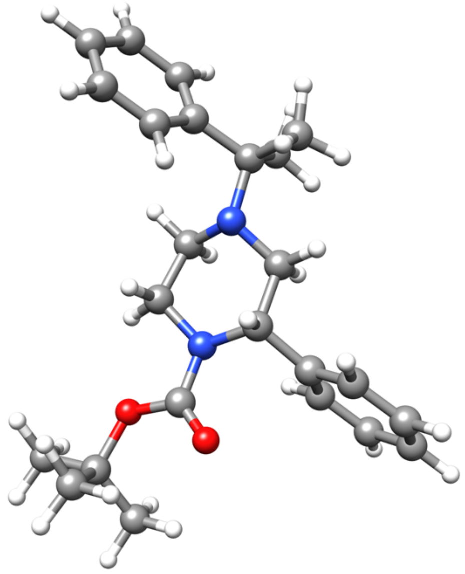

Route : # opt freq b3lyp scrf=(solvent=thf) geom=connectivity def2tzvp
empiricaldispersion=gd3bj int=ultrafine pop=(regular,mk)
SMILES : CC(C)(C)OC(=O)N2CCN(C(C)(C)c1ccccc1)CC2c3ccccc3
Formula : $C_{24}H_{32}N_2O_2$
Charge : 0
Multiplicity : 1
Dipole : 3.1397 Debye
Energy : -1194.37986331 a.u.
Gibbs Energy : -1193.919973 a.u.
Number of imaginary frequencies : 0

Cartesian Coordinates (XYZ format)

60

C	0.58254600	1.55735700	0.07935800
C	-0.71366600	0.95659900	-0.48887500
C	0.10553700	-1.22046200	0.47109600
C	1.29715800	-0.46443900	1.01361600
H	0.36632900	1.94446300	1.08773300
H	0.82561100	2.40844800	-0.55005100
H	-0.22717100	-1.94987600	1.20280200
H	1.05860700	-0.06926500	2.01400100
H	2.12191000	-1.16678300	1.12226900
H	-0.56651800	0.79553000	-1.56232700
N	-1.02346800	-0.33049800	0.15323100
N	1.66608000	0.59697700	0.09090900
C	3.03610300	1.13683000	0.28622500
C	3.31011700	2.23786600	-0.75397700
H	4.37323500	2.47641500	-0.75188000
H	3.03546300	1.90082700	-1.75352600
H	2.76530500	3.15444700	-0.52811600
C	3.22640800	1.73559200	1.68914700
H	3.15250300	0.98058400	2.47144600

H	4.19677300	2.22328200	1.77964500
H	2.46422100	2.49300800	1.87246500
C	4.01695000	-0.00602500	-0.00304400
C	3.83035800	-0.79064900	-1.14454000
C	5.12353600	-0.27005700	0.80052400
C	4.71836800	-1.80560800	-1.47063500
H	2.96598600	-0.60759400	-1.76735700
C	6.01834300	-1.28765000	0.47684700
H	5.30169700	0.31223400	1.69286700
C	5.82050200	-2.06018700	-0.65896200
H	4.54998100	-2.40130300	-2.35909900
H	6.86887700	-1.47489800	1.12012400
H	6.51360600	-2.85285000	-0.90970500
C	-2.21469000	-0.91579500	-0.21060200
O	-3.04637900	-0.38769600	-0.92377000
O	-2.34071200	-2.13647100	0.34139000
C	-3.53382400	-2.96696300	0.11710800
C	-4.76879000	-2.27148600	0.67889100
H	-5.00754200	-1.37450300	0.11413400
H	-5.61819800	-2.95462200	0.63199100
H	-4.60644600	-2.00120400	1.72343800
C	-3.21579700	-4.22367800	0.91788900
H	-2.30848200	-4.69805700	0.54249500
H	-3.07226700	-3.98067300	1.97119200
H	-4.03882600	-4.93375800	0.83453200
C	-3.67410200	-3.29839200	-1.36454800
H	-4.47405000	-4.02881200	-1.49437500
H	-3.91063500	-2.41264400	-1.94759400
H	-2.74875800	-3.73653800	-1.74186100
H	0.41322600	-1.76010400	-0.43186600
C	-1.79854000	1.99408400	-0.29580300
C	-2.41906200	2.16930600	0.93929900
C	-2.11177500	2.86437600	-1.33495100
C	-3.34946100	3.18370600	1.12468400
H	-2.18352400	1.49553500	1.75292900
C	-3.03738100	3.88570600	-1.15196900
H	-1.63660900	2.73392700	-2.29995800
C	-3.66226200	4.04742600	0.07935200
H	-3.83187500	3.30094800	2.08693400
H	-3.27501200	4.54999900	-1.97317100
H	-4.38842900	4.83708000	0.22317400

Frequencies

Mode	IR frequency	IR intensity	Raman intensity
1	16.92750000	0.14550000	0.00000000
2	25.48900000	0.00940000	0.00000000
3	28.80960000	1.26870000	0.00000000
4	37.93280000	0.17050000	0.00000000
5	39.50600000	0.68550000	0.00000000
6	48.12010000	0.23530000	0.00000000
7	67.70140000	1.71730000	0.00000000
8	70.74700000	0.88300000	0.00000000
9	92.50190000	1.06280000	0.00000000

10	109.26090000	0.18700000	0.00000000
11	121.45410000	0.84690000	0.00000000
12	145.45710000	2.67230000	0.00000000
13	174.05900000	4.00370000	0.00000000
14	191.68470000	0.60290000	0.00000000
15	203.19840000	0.33610000	0.00000000
16	209.48160000	4.27490000	0.00000000
17	232.37320000	2.42270000	0.00000000
18	242.43800000	2.99630000	0.00000000
19	245.65300000	1.64000000	0.00000000
20	261.74270000	2.74410000	0.00000000
21	268.90830000	5.64180000	0.00000000
22	272.43170000	3.86070000	0.00000000
23	287.32980000	1.85360000	0.00000000
24	300.89020000	1.56470000	0.00000000
25	304.47670000	0.87720000	0.00000000
26	316.64970000	0.12970000	0.00000000
27	330.37220000	0.42320000	0.00000000
28	334.47620000	20.38350000	0.00000000
29	352.69660000	1.55200000	0.00000000
30	353.50200000	0.85080000	0.00000000
31	406.45410000	4.31230000	0.00000000
32	416.23350000	3.51090000	0.00000000
33	417.63330000	0.10150000	0.00000000
34	417.85650000	0.41940000	0.00000000
35	428.76040000	2.59070000	0.00000000
36	458.72050000	2.86240000	0.00000000
37	462.78460000	5.72860000	0.00000000
38	483.74660000	0.80740000	0.00000000
39	503.75920000	1.75700000	0.00000000
40	529.80480000	6.03840000	0.00000000
41	548.22720000	31.75440000	0.00000000
42	560.58100000	10.09700000	0.00000000
43	588.99690000	8.89110000	0.00000000
44	612.29640000	16.10710000	0.00000000
45	636.94610000	0.25640000	0.00000000
46	637.72440000	2.26820000	0.00000000
47	641.20940000	5.17480000	0.00000000
48	691.44490000	1.25060000	0.00000000
49	715.42610000	58.02960000	0.00000000
50	716.71970000	81.61040000	0.00000000
51	734.82190000	2.90700000	0.00000000
52	770.63050000	17.55210000	0.00000000

53	776.16130000	11.14340000	0.00000000
54	786.91240000	48.38720000	0.00000000
55	797.95780000	31.06770000	0.00000000
56	815.40600000	20.49230000	0.00000000
57	834.89970000	7.77320000	0.00000000
58	852.84380000	12.89800000	0.00000000
59	858.95960000	0.05220000	0.00000000
60	861.51870000	0.29520000	0.00000000
61	875.45980000	39.18670000	0.00000000
62	913.04730000	4.31880000	0.00000000
63	926.93350000	9.98940000	0.00000000
64	930.11250000	0.03770000	0.00000000
65	930.48410000	0.02800000	0.00000000
66	933.48650000	1.24420000	0.00000000
67	942.08160000	11.11370000	0.00000000
68	953.48930000	2.36360000	0.00000000
69	971.73680000	0.08470000	0.00000000
70	975.69670000	0.20680000	0.00000000
71	983.36180000	0.14100000	0.00000000
72	990.30090000	8.88830000	0.00000000
73	997.35990000	54.33640000	0.00000000
74	999.59660000	31.40920000	0.00000000
75	1021.76930000	7.41480000	0.00000000
76	1024.13760000	18.07400000	0.00000000
77	1024.99940000	20.87450000	0.00000000
78	1026.21110000	5.48700000	0.00000000
79	1051.68300000	4.86270000	0.00000000
80	1052.41960000	3.02160000	0.00000000
81	1053.28040000	42.31610000	0.00000000
82	1054.06970000	7.57140000	0.00000000
83	1066.02900000	28.18050000	0.00000000
84	1088.63120000	49.41020000	0.00000000
85	1101.02930000	19.56360000	0.00000000
86	1103.99650000	5.80800000	0.00000000
87	1123.42850000	11.74270000	0.00000000
88	1136.49160000	6.83490000	0.00000000
89	1145.67640000	184.72930000	0.00000000
90	1164.37580000	69.31400000	0.00000000
91	1178.78230000	1.61340000	0.00000000
92	1180.19140000	3.13570000	0.00000000
93	1184.84320000	340.19420000	0.00000000
94	1192.62390000	212.89220000	0.00000000
95	1199.47640000	11.17520000	0.00000000

96	1202.42230000	117.23610000	0.00000000
97	1206.29830000	4.55780000	0.00000000
98	1221.97500000	11.82370000	0.00000000
99	1233.97630000	59.82840000	0.00000000
100	1267.56170000	164.13560000	0.00000000
101	1269.13560000	38.16960000	0.00000000
102	1274.15110000	45.82330000	0.00000000
103	1281.20250000	23.60070000	0.00000000
104	1291.12950000	37.69040000	0.00000000
105	1299.43410000	188.75550000	0.00000000
106	1330.16050000	2.08530000	0.00000000
107	1331.28830000	13.70900000	0.00000000
108	1348.45080000	72.90130000	0.00000000
109	1352.98030000	104.28470000	0.00000000
110	1359.09000000	6.28460000	0.00000000
111	1365.93870000	130.81290000	0.00000000
112	1389.48700000	11.09920000	0.00000000
113	1396.60660000	24.41250000	0.00000000
114	1397.28140000	31.60050000	0.00000000
115	1399.48710000	22.72600000	0.00000000
116	1404.62730000	71.26090000	0.00000000
117	1414.88830000	32.65560000	0.00000000
118	1416.90920000	27.79630000	0.00000000
119	1423.91160000	38.75990000	0.00000000
120	1431.58780000	2.61540000	0.00000000
121	1466.55970000	0.14140000	0.00000000
122	1479.49490000	11.02260000	0.00000000
123	1483.84450000	2.94620000	0.00000000
124	1484.08910000	4.81030000	0.00000000
125	1484.25730000	0.09690000	0.00000000
126	1487.87580000	19.98820000	0.00000000
127	1489.84390000	8.06770000	0.00000000
128	1491.18240000	2.51650000	0.00000000
129	1493.79430000	0.76240000	0.00000000
130	1497.17960000	2.42230000	0.00000000
131	1498.35640000	8.13860000	0.00000000
132	1503.33420000	7.64190000	0.00000000
133	1509.34920000	13.83660000	0.00000000
134	1510.53640000	3.79570000	0.00000000
135	1516.82020000	23.61880000	0.00000000
136	1531.85820000	26.37790000	0.00000000
137	1532.52410000	10.97630000	0.00000000
138	1624.42360000	0.77410000	0.00000000

139	1628.91130000	1.80050000	0.00000000
140	1644.66000000	10.82170000	0.00000000
141	1647.49020000	10.47060000	0.00000000
142	1721.45060000	567.32570000	0.00000000
143	2933.03900000	36.99960000	0.00000000
144	2937.45470000	134.59450000	0.00000000
145	3000.79430000	60.30940000	0.00000000
146	3006.02690000	76.24420000	0.00000000
147	3041.09430000	16.22530000	0.00000000
148	3043.20980000	39.07910000	0.00000000
149	3043.65620000	27.28190000	0.00000000
150	3049.53650000	25.54930000	0.00000000
151	3049.96820000	21.54960000	0.00000000
152	3102.12490000	4.65240000	0.00000000
153	3102.21630000	26.36480000	0.00000000
154	3103.72620000	29.02750000	0.00000000
155	3107.50350000	3.28270000	0.00000000
156	3110.89890000	44.00610000	0.00000000
157	3111.52020000	83.78020000	0.00000000
158	3115.21290000	34.78250000	0.00000000
159	3116.99710000	62.75520000	0.00000000
160	3118.19360000	33.27630000	0.00000000
161	3133.62370000	31.03300000	0.00000000
162	3136.49070000	14.85580000	0.00000000
163	3145.06810000	1.91620000	0.00000000
164	3148.39350000	24.76500000	0.00000000
165	3162.08420000	9.52310000	0.00000000
166	3167.13250000	0.73090000	0.00000000
167	3168.53020000	0.01020000	0.00000000
168	3175.38570000	16.94620000	0.00000000
169	3177.80800000	21.43640000	0.00000000
170	3185.47860000	39.58230000	0.00000000
171	3189.43940000	34.67580000	0.00000000
172	3194.34360000	20.41050000	0.00000000
173	3198.83140000	15.87910000	0.00000000
174	3206.55090000	12.59290000	0.00000000

Axial Clockwise Transition State

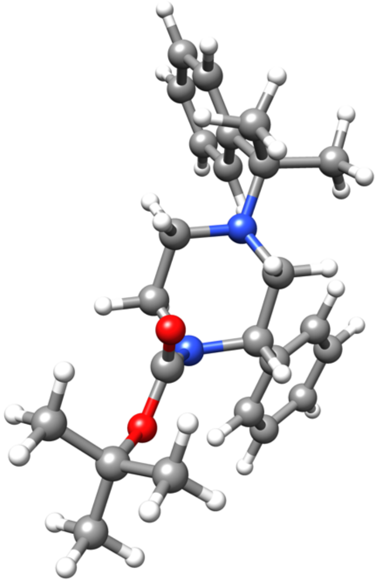

Route : # opt freq b3lyp scrf=(solvent=thf) geom=connectivity def2tzvp
empiricaldispersion=gd3bj int=ultrafine pop=(regular,mk)
SMILES : CC(C)(C)OC(=O)N2CCN(C(C)(C)c1ccccc1)CC2c3ccccc3
Formula : $C_{24}H_{32}N_2O_2$
Charge : 0
Multiplicity : 1
Dipole : 1.6544 Debye
Energy : -1194.35793774 a.u.
Gibbs Energy : -1193.895918 a.u.
Number of imaginary frequencies : 1

Cartesian Coordinates (XYZ format)

60

C	0.61246900	-1.22856400	0.52203800
C	-0.44294200	-0.39147400	1.22993400
C	-0.90368300	0.78122200	-0.86036600
C	0.16458200	-0.09235000	-1.53281300
H	-0.92258000	-0.96985400	2.02037100
H	0.15827600	-2.16526600	0.17222700
H	1.39523100	-1.48310000	1.23628900
H	-1.71902100	0.89910500	-1.58011200
H	-0.34391200	-0.96824900	-1.95796300
H	0.59732400	0.45918600	-2.36178200
H	0.03711800	0.47329300	1.69326900
N	-1.48220800	0.12581100	0.32936200
N	1.19882800	-0.46085400	-0.57533900
C	2.43600900	-1.06861200	-1.12525300
C	2.19408800	-2.49052100	-1.66022400
H	3.07670600	-2.87392700	-2.17200400
H	1.93223200	-3.18986200	-0.86726700
H	1.37813300	-2.47494900	-2.38325300

C	2.98681900	-0.21336200	-2.28083900
H	3.07947000	0.83082300	-1.98460500
H	3.97914900	-0.57605600	-2.54711600
H	2.36129200	-0.27818700	-3.17098900
C	3.49158100	-1.02821300	-0.01299500
C	4.34306600	-2.09619200	0.25813700
C	3.64832100	0.14567800	0.72949200
C	5.32323700	-1.99885800	1.24362100
H	4.25063500	-3.02090700	-0.29313900
C	4.62274000	0.24691300	1.71195700
H	2.97948100	0.97378800	0.53985800
C	5.46776900	-0.82816300	1.97500700
H	5.97024600	-2.84487200	1.43868900
H	4.72217200	1.16556800	2.27668500
H	6.22676800	-0.75267400	2.74308100
C	-0.44170200	2.18343800	-0.47225000
C	0.73448800	2.75362200	-0.95327700
C	-1.25323600	2.95120100	0.36503300
C	1.09399400	4.05239800	-0.60558900
H	1.39033700	2.18784400	-1.59561800
C	-0.90020200	4.24844600	0.71278600
H	-2.16083100	2.51115600	0.75474000
C	0.27972800	4.80585900	0.23001000
H	2.01765400	4.47021000	-0.98584400
H	-1.54609700	4.82447500	1.36357900
H	0.56081000	5.81466400	0.50385300
C	-2.51264600	-0.80524900	0.03098800
O	-2.37716100	-1.83534100	-0.59036600
O	-3.66198800	-0.36618300	0.54192600
C	-4.92835900	-1.11932900	0.38868300
C	-5.28934200	-1.22531500	-1.08775300
H	-4.58942800	-1.85988400	-1.62493000
H	-6.28879300	-1.65237500	-1.18045700
H	-5.29899200	-0.23556200	-1.54662200
C	-5.92560200	-0.23639600	1.12657200
H	-5.63824100	-0.12447800	2.17237800
H	-5.97323900	0.75263500	0.67010100
H	-6.91777700	-0.68606600	1.08511100
C	-4.80818800	-2.47982300	1.06396800
H	-5.78801100	-2.95915300	1.07520300
H	-4.11095200	-3.12645400	0.53794900
H	-4.47623100	-2.36152100	2.09646600

Frequencies

Mode	IR frequency	IR intensity	Raman intensity
1	-55.60110000	2.07050000	0.00000000
2	15.00210000	0.04520000	0.00000000
3	24.31270000	0.02760000	0.00000000
4	31.64830000	0.28500000	0.00000000
5	40.10130000	0.25690000	0.00000000
6	49.25880000	0.03500000	0.00000000
7	58.29700000	0.43240000	0.00000000
8	80.28660000	0.49030000	0.00000000

9	96.89920000	0.21500000	0.00000000
10	104.95340000	0.27990000	0.00000000
11	147.41730000	1.34690000	0.00000000
12	164.72260000	4.73330000	0.00000000
13	172.42360000	2.25640000	0.00000000
14	200.56410000	0.35180000	0.00000000
15	203.88290000	0.08620000	0.00000000
16	220.44500000	1.68360000	0.00000000
17	232.67250000	0.73070000	0.00000000
18	242.88760000	0.42070000	0.00000000
19	255.17720000	0.21610000	0.00000000
20	272.87350000	0.25480000	0.00000000
21	276.26580000	8.10970000	0.00000000
22	285.88120000	0.93880000	0.00000000
23	294.18350000	0.84460000	0.00000000
24	313.26670000	0.94690000	0.00000000
25	323.91740000	6.10470000	0.00000000
26	324.77070000	1.85580000	0.00000000
27	337.90110000	10.44650000	0.00000000
28	345.76090000	0.51960000	0.00000000
29	358.97000000	0.84990000	0.00000000
30	360.92220000	2.60300000	0.00000000
31	401.58570000	1.09850000	0.00000000
32	414.70290000	2.88770000	0.00000000
33	416.92740000	0.63760000	0.00000000
34	418.72410000	0.41170000	0.00000000
35	438.06890000	2.80200000	0.00000000
36	456.54960000	2.45310000	0.00000000
37	471.32930000	0.49880000	0.00000000
38	477.04450000	6.01730000	0.00000000
39	485.27830000	16.07920000	0.00000000
40	518.29740000	9.37050000	0.00000000
41	540.24140000	1.67980000	0.00000000
42	576.89810000	17.45930000	0.00000000
43	587.10010000	3.38160000	0.00000000
44	606.68860000	16.06280000	0.00000000
45	637.86350000	0.53750000	0.00000000
46	638.23990000	0.09970000	0.00000000
47	687.73260000	11.23230000	0.00000000
48	710.59950000	46.99210000	0.00000000
49	718.15270000	52.67330000	0.00000000
50	719.14920000	55.09870000	0.00000000
51	738.97150000	6.35180000	0.00000000

52	754.90250000	31.21800000	0.00000000
53	773.32120000	4.27770000	0.00000000
54	783.32850000	27.72650000	0.00000000
55	794.80640000	13.39010000	0.00000000
56	814.72520000	10.93940000	0.00000000
57	835.03110000	11.64760000	0.00000000
58	849.80950000	37.88930000	0.00000000
59	858.46280000	0.17840000	0.00000000
60	862.28320000	25.07740000	0.00000000
61	864.00620000	0.05530000	0.00000000
62	905.29230000	7.80120000	0.00000000
63	928.19720000	9.41390000	0.00000000
64	931.94200000	1.40340000	0.00000000
65	932.55900000	0.21260000	0.00000000
66	933.21740000	0.07960000	0.00000000
67	939.92300000	13.00820000	0.00000000
68	961.83930000	5.82990000	0.00000000
69	973.59730000	0.08550000	0.00000000
70	974.84520000	103.24930000	0.00000000
71	979.11010000	4.64480000	0.00000000
72	982.22230000	33.52370000	0.00000000
73	982.68490000	1.70740000	0.00000000
74	994.68530000	5.80540000	0.00000000
75	997.97010000	2.73390000	0.00000000
76	1021.59820000	7.83780000	0.00000000
77	1025.07280000	25.40630000	0.00000000
78	1025.53270000	1.51670000	0.00000000
79	1027.21740000	32.13840000	0.00000000
80	1053.04810000	5.56790000	0.00000000
81	1053.19650000	20.27470000	0.00000000
82	1054.24170000	3.02350000	0.00000000
83	1058.58890000	6.81640000	0.00000000
84	1066.36650000	11.52810000	0.00000000
85	1095.19360000	24.84150000	0.00000000
86	1103.33080000	19.03640000	0.00000000
87	1112.38420000	26.85310000	0.00000000
88	1124.46580000	5.57430000	0.00000000
89	1134.32930000	42.34890000	0.00000000
90	1151.94270000	22.09490000	0.00000000
91	1158.21320000	138.27200000	0.00000000
92	1175.82740000	672.56880000	0.00000000
93	1179.01810000	17.07450000	0.00000000
94	1179.57740000	1.93410000	0.00000000

95	1199.23900000	15.14130000	0.00000000
96	1203.63090000	67.33650000	0.00000000
97	1205.54800000	7.58110000	0.00000000
98	1207.88070000	20.18620000	0.00000000
99	1227.23410000	11.82710000	0.00000000
100	1249.43140000	226.53450000	0.00000000
101	1266.42200000	240.82550000	0.00000000
102	1269.67720000	8.65470000	0.00000000
103	1272.24760000	26.81630000	0.00000000
104	1283.46540000	18.10090000	0.00000000
105	1313.71400000	94.65010000	0.00000000
106	1316.67120000	71.06960000	0.00000000
107	1329.80230000	3.17810000	0.00000000
108	1340.06150000	9.94120000	0.00000000
109	1345.61310000	20.64770000	0.00000000
110	1358.47740000	0.50970000	0.00000000
111	1361.25940000	3.26290000	0.00000000
112	1377.61710000	22.23750000	0.00000000
113	1393.07330000	59.13230000	0.00000000
114	1396.26740000	12.23900000	0.00000000
115	1398.66050000	25.25120000	0.00000000
116	1401.61280000	42.11230000	0.00000000
117	1405.51370000	26.02580000	0.00000000
118	1413.12050000	11.89360000	0.00000000
119	1425.23540000	19.95970000	0.00000000
120	1427.08090000	3.26550000	0.00000000
121	1467.93560000	0.19730000	0.00000000
122	1479.10460000	6.22920000	0.00000000
123	1480.52860000	13.02210000	0.00000000
124	1483.06370000	9.73410000	0.00000000
125	1484.04820000	0.14670000	0.00000000
126	1484.52070000	2.47450000	0.00000000
127	1486.95690000	1.23660000	0.00000000
128	1489.23810000	17.81790000	0.00000000
129	1493.10530000	0.70100000	0.00000000
130	1497.52560000	2.39580000	0.00000000
131	1498.03630000	2.16120000	0.00000000
132	1503.68360000	3.40110000	0.00000000
133	1510.57460000	11.23690000	0.00000000
134	1516.76860000	26.89710000	0.00000000
135	1517.49730000	35.61960000	0.00000000
136	1531.29280000	20.40090000	0.00000000
137	1534.06120000	16.20940000	0.00000000

138	1624.09060000	0.59340000	0.00000000
139	1625.47950000	1.06550000	0.00000000
140	1644.35740000	8.31600000	0.00000000
141	1645.52360000	7.21370000	0.00000000
142	1726.29590000	395.72150000	0.00000000
143	2973.86810000	38.89560000	0.00000000
144	2977.10650000	106.73110000	0.00000000
145	3030.53410000	38.18050000	0.00000000
146	3041.37760000	34.42340000	0.00000000
147	3042.80300000	16.18080000	0.00000000
148	3045.28450000	32.07460000	0.00000000
149	3045.30610000	32.60470000	0.00000000
150	3050.47440000	30.32670000	0.00000000
151	3051.58530000	19.07330000	0.00000000
152	3087.64220000	36.20100000	0.00000000
153	3091.02250000	41.91370000	0.00000000
154	3104.87320000	6.65670000	0.00000000
155	3105.91100000	5.24350000	0.00000000
156	3106.04630000	21.72160000	0.00000000
157	3111.73520000	79.27180000	0.00000000
158	3113.33880000	42.98830000	0.00000000
159	3117.51820000	33.10190000	0.00000000
160	3118.51990000	58.30760000	0.00000000
161	3121.22690000	40.45160000	0.00000000
162	3132.83570000	35.15600000	0.00000000
163	3143.02000000	1.54950000	0.00000000
164	3146.04230000	26.45780000	0.00000000
165	3165.95950000	0.63350000	0.00000000
166	3166.84090000	0.30430000	0.00000000
167	3174.25880000	18.67210000	0.00000000
168	3175.51620000	20.53940000	0.00000000
169	3188.34950000	32.42830000	0.00000000
170	3189.24290000	31.22620000	0.00000000
171	3197.56500000	12.65000000	0.00000000
172	3197.98450000	17.51440000	0.00000000
173	3203.78520000	14.12930000	0.00000000
174	3228.74960000	9.50000000	0.00000000

Axial Anticlockwise Transition State

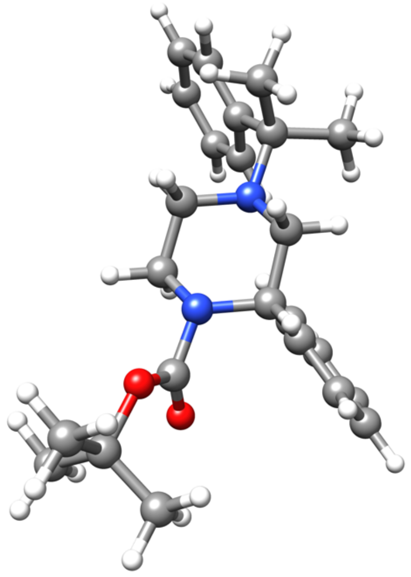

Route : # opt freq b3lyp scrf=(solvent=thf) geom=connectivity def2tzvp empiricaldispersion=gd3bj int=ultrafine pop=(regular,mk)
SMILES : CC(C)(C)OC(=O)N2CCN(C(C)(C)c1ccccc1)CC2c3ccccc3
Formula : $C_{24}H_{32}N_2O_2$
Charge : 0
Multiplicity : 1
Dipole : 2.6170 Debye
Energy : -1194.35848593 a.u.
Gibbs Energy : -1193.896321 a.u.
Number of imaginary frequencies : 1

Cartesian Coordinates (XYZ format)

60

C	-0.97462300	-1.48585000	0.04757600
C	0.29926600	-1.12107000	-0.69163600
C	0.84721200	0.48818400	1.11607700
C	-0.47208800	0.06942200	1.77845900
H	0.70317500	-2.01168200	-1.17642400
H	-0.76229500	-2.34316200	0.70551400
H	-1.72788400	-1.79523300	-0.67505800
H	1.58021900	0.55542200	1.92333300
H	-0.24034300	-0.74332900	2.48509000
H	-0.83703500	0.91735900	2.35054400
H	0.08741400	-0.38193600	-1.47294600
N	1.29342400	-0.63829600	0.27042700
N	-1.46608900	-0.33513400	0.79570400
C	-2.85843500	-0.48164000	1.29744600
C	-2.99457000	-1.64436900	2.29366500
H	-4.00097700	-1.68585800	2.71037700
H	-2.77736300	-2.60861100	1.83524000
H	-2.30344800	-1.50025000	3.12389700
C	-3.29160400	0.81426400	2.00784700

H	-3.05016900	1.68804800	1.40326400
H	-4.36919800	0.79230600	2.16764900
H	-2.81533400	0.92035300	2.98218600
C	-3.76788500	-0.65023800	0.07405400
C	-4.69806600	-1.68007500	-0.04136900
C	-3.69175400	0.28384400	-0.96296700
C	-5.52491700	-1.77923900	-1.15861600
H	-4.78746500	-2.42396200	0.73665700
C	-4.51128100	0.18962600	-2.07803500
H	-2.97334600	1.08770900	-0.89529100
C	-5.43493200	-0.84710400	-2.18245500
H	-6.23684100	-2.59228000	-1.22486600
H	-4.42951800	0.92548800	-2.86812200
H	-6.07444800	-0.92490800	-3.05221500
C	0.79336000	1.86303600	0.47021200
C	1.72525800	2.82133600	0.86894900
C	-0.15529800	2.22523400	-0.48874500
C	1.72152300	4.10149500	0.32830400
H	2.47052800	2.55804100	1.61049000
C	-0.16168600	3.50331000	-1.03439900
H	-0.89759200	1.50641900	-0.79323300
C	0.77627100	4.44732100	-0.63027400
H	2.45699200	4.82630100	0.65391900
H	-0.90530300	3.76303900	-1.77761800
H	0.76949200	5.44210000	-1.05705800
C	2.58372500	-0.48257400	-0.30642500
O	2.90022500	0.38395100	-1.08642300
O	3.38461500	-1.45100900	0.13818800
C	4.78696200	-1.57753200	-0.32062700
C	4.82066100	-1.81765700	-1.82487900
H	4.49216300	-0.94095400	-2.37667800
H	5.84217400	-2.05549000	-2.12452900
H	4.18404400	-2.66395900	-2.08718300
C	5.26981700	-2.80872600	0.43396100
H	5.19740400	-2.65122800	1.51043300
H	4.67193100	-3.68090900	0.16811900
H	6.31096100	-3.01122200	0.18203400
C	5.57884200	-0.34396200	0.09540900
H	6.63535400	-0.50947400	-0.11995500
H	5.24852300	0.54131100	-0.44130800
H	5.47356600	-0.17118300	1.16750600

Frequencies

Mode	IR frequency	IR intensity	Raman intensity
1	-52.34890000	1.49300000	0.00000000
2	22.48750000	0.14750000	0.00000000
3	24.82380000	0.08700000	0.00000000
4	36.19030000	0.19310000	0.00000000
5	41.61670000	0.31690000	0.00000000
6	48.03900000	0.24800000	0.00000000
7	58.63670000	0.08000000	0.00000000
8	74.59650000	0.86170000	0.00000000
9	100.67420000	0.15600000	0.00000000

10	111.85030000	1.94080000	0.00000000
11	130.01240000	1.49620000	0.00000000
12	160.61840000	1.68140000	0.00000000
13	176.53010000	3.11180000	0.00000000
14	193.56230000	2.83440000	0.00000000
15	199.38430000	0.12290000	0.00000000
16	205.58620000	0.89590000	0.00000000
17	236.21540000	3.63270000	0.00000000
18	240.03910000	0.67920000	0.00000000
19	250.33130000	2.89640000	0.00000000
20	256.46810000	2.46840000	0.00000000
21	269.82580000	0.10050000	0.00000000
22	280.40370000	0.24630000	0.00000000
23	296.72610000	1.32070000	0.00000000
24	307.66250000	2.42540000	0.00000000
25	310.15070000	2.99190000	0.00000000
26	324.16860000	9.40360000	0.00000000
27	327.02330000	1.02090000	0.00000000
28	328.54870000	0.42880000	0.00000000
29	357.64630000	0.44270000	0.00000000
30	361.67440000	2.85320000	0.00000000
31	379.82170000	1.62000000	0.00000000
32	411.87910000	0.23640000	0.00000000
33	417.95070000	0.22850000	0.00000000
34	420.55510000	1.55410000	0.00000000
35	422.67030000	4.07290000	0.00000000
36	451.94990000	0.78760000	0.00000000
37	474.58510000	6.71240000	0.00000000
38	485.51010000	3.78140000	0.00000000
39	498.98470000	8.47580000	0.00000000
40	523.19600000	12.11860000	0.00000000
41	538.36960000	6.81520000	0.00000000
42	577.91960000	19.98440000	0.00000000
43	584.14140000	5.16870000	0.00000000
44	618.50290000	15.76590000	0.00000000
45	637.10160000	0.81350000	0.00000000
46	638.82930000	0.44620000	0.00000000
47	676.70670000	27.76580000	0.00000000
48	715.10970000	111.92240000	0.00000000
49	716.75160000	12.38690000	0.00000000
50	718.19810000	23.69070000	0.00000000
51	755.94800000	24.95300000	0.00000000
52	773.12300000	5.47240000	0.00000000

53	775.76740000	20.86020000	0.00000000
54	793.73300000	11.36800000	0.00000000
55	795.27560000	39.08130000	0.00000000
56	814.45000000	2.03320000	0.00000000
57	825.55680000	1.24390000	0.00000000
58	849.59830000	34.68280000	0.00000000
59	859.29030000	21.65260000	0.00000000
60	859.91250000	13.24390000	0.00000000
61	861.69170000	0.87770000	0.00000000
62	909.52560000	8.65980000	0.00000000
63	931.99390000	11.42850000	0.00000000
64	932.79800000	0.20180000	0.00000000
65	933.17080000	0.04320000	0.00000000
66	934.10640000	1.13750000	0.00000000
67	940.80710000	12.91370000	0.00000000
68	960.47800000	55.85390000	0.00000000
69	974.22630000	0.04760000	0.00000000
70	978.93150000	1.55570000	0.00000000
71	983.36100000	0.12760000	0.00000000
72	990.42710000	30.04220000	0.00000000
73	993.74860000	1.65640000	0.00000000
74	995.30720000	11.84320000	0.00000000
75	998.57270000	5.24870000	0.00000000
76	1019.09910000	41.51840000	0.00000000
77	1022.15660000	0.41310000	0.00000000
78	1026.48370000	5.55070000	0.00000000
79	1029.18360000	9.06530000	0.00000000
80	1053.09180000	18.49240000	0.00000000
81	1053.30210000	0.75260000	0.00000000
82	1057.75880000	7.60410000	0.00000000
83	1062.04220000	7.42970000	0.00000000
84	1090.09970000	17.07210000	0.00000000
85	1100.40410000	10.08130000	0.00000000
86	1107.41090000	6.09750000	0.00000000
87	1120.11480000	6.88090000	0.00000000
88	1125.75570000	81.35850000	0.00000000
89	1130.56540000	34.29050000	0.00000000
90	1147.58790000	127.67380000	0.00000000
91	1164.42620000	10.40120000	0.00000000
92	1175.42710000	726.11590000	0.00000000
93	1179.52290000	0.47160000	0.00000000
94	1180.15330000	0.55230000	0.00000000
95	1198.21620000	71.96190000	0.00000000

96	1206.14380000	2.36300000	0.00000000
97	1207.97660000	13.77020000	0.00000000
98	1209.74860000	30.30770000	0.00000000
99	1227.72330000	30.07210000	0.00000000
100	1247.58050000	115.02500000	0.00000000
101	1271.82380000	20.70660000	0.00000000
102	1274.89940000	194.91280000	0.00000000
103	1280.01920000	40.09930000	0.00000000
104	1284.25360000	31.72890000	0.00000000
105	1296.20800000	223.91570000	0.00000000
106	1319.99830000	90.90690000	0.00000000
107	1330.29760000	2.64910000	0.00000000
108	1344.07730000	9.99850000	0.00000000
109	1352.04080000	10.10060000	0.00000000
110	1358.88910000	0.88330000	0.00000000
111	1363.41110000	21.21250000	0.00000000
112	1380.46920000	2.80260000	0.00000000
113	1397.26290000	31.44280000	0.00000000
114	1399.18690000	24.36780000	0.00000000
115	1401.56500000	24.07030000	0.00000000
116	1402.09700000	9.92820000	0.00000000
117	1413.08730000	2.49860000	0.00000000
118	1414.57280000	8.19280000	0.00000000
119	1425.27080000	18.23220000	0.00000000
120	1428.77070000	11.09050000	0.00000000
121	1467.57760000	0.17390000	0.00000000
122	1479.42620000	11.44770000	0.00000000
123	1483.34110000	0.14430000	0.00000000
124	1484.15490000	2.37700000	0.00000000
125	1484.35520000	2.23220000	0.00000000
126	1488.74590000	18.40350000	0.00000000
127	1488.94530000	0.46240000	0.00000000
128	1491.98330000	9.14300000	0.00000000
129	1493.77450000	0.79380000	0.00000000
130	1497.90760000	2.17880000	0.00000000
131	1499.13330000	4.63030000	0.00000000
132	1506.82670000	9.66540000	0.00000000
133	1508.01230000	11.82250000	0.00000000
134	1511.07840000	7.12450000	0.00000000
135	1516.83760000	20.78300000	0.00000000
136	1531.30970000	16.43260000	0.00000000
137	1532.66020000	19.44650000	0.00000000
138	1624.39210000	0.53800000	0.00000000

139	1624.72190000	0.59310000	0.00000000
140	1644.81530000	7.15520000	0.00000000
141	1645.72240000	3.08550000	0.00000000
142	1739.89550000	411.29300000	0.00000000
143	2930.87340000	79.91910000	0.00000000
144	2938.36440000	144.46310000	0.00000000
145	2999.41060000	67.57250000	0.00000000
146	3041.76810000	39.36460000	0.00000000
147	3042.53740000	15.50400000	0.00000000
148	3045.32640000	25.82090000	0.00000000
149	3045.38000000	37.23170000	0.00000000
150	3050.45670000	27.70000000	0.00000000
151	3051.39320000	19.44690000	0.00000000
152	3070.70680000	45.41310000	0.00000000
153	3100.73170000	24.57480000	0.00000000
154	3104.94700000	6.63560000	0.00000000
155	3105.84570000	22.17850000	0.00000000
156	3107.49650000	1.87850000	0.00000000
157	3111.96610000	76.17320000	0.00000000
158	3112.96280000	43.36510000	0.00000000
159	3116.19460000	39.99590000	0.00000000
160	3118.18380000	57.45790000	0.00000000
161	3120.35200000	31.85240000	0.00000000
162	3131.68020000	32.80060000	0.00000000
163	3143.60010000	1.42970000	0.00000000
164	3146.42030000	26.42460000	0.00000000
165	3158.28300000	11.74930000	0.00000000
166	3167.93260000	0.41520000	0.00000000
167	3168.21280000	1.46210000	0.00000000
168	3176.25890000	18.37280000	0.00000000
169	3178.03240000	40.04700000	0.00000000
170	3190.67770000	36.35600000	0.00000000
171	3191.44280000	32.52360000	0.00000000
172	3207.06320000	23.07640000	0.00000000
173	3208.62750000	0.33920000	0.00000000
174	3240.04970000	2.71440000	0.00000000

Equatorial Clockwise Transition State

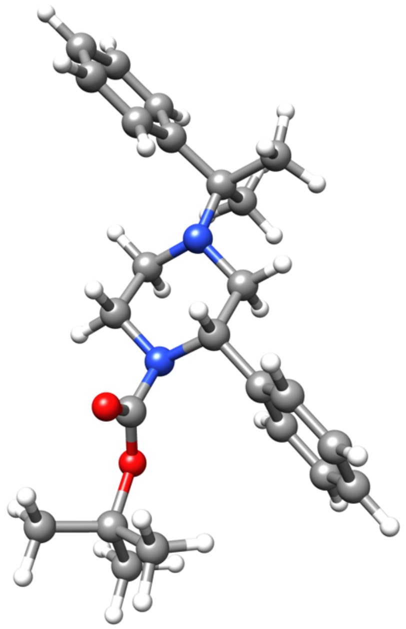

Route : # opt freq b3lyp scrf=(solvent=thf) geom=connectivity def2tzvp empiricaldispersion=gd3bj int=ultrafine pop=(regular,mk)
SMILES : CC(C)(C)OC(=O)N2CCN(C(C)(C)c1ccccc1)CC2c3ccccc3
Formula : $C_{24}H_{32}N_2O_2$
Charge : 0
Multiplicity : 1
Dipole : 2.3097 Debye
Energy : -1194.36728384 a.u.
Gibbs Energy : -1193.905163 a.u.
Number of imaginary frequencies : 1

Cartesian Coordinates (XYZ format)

60

C	0.69346100	1.31528100	0.24194500
C	-0.51652900	0.65391500	-0.42184600
C	0.39305800	-1.50896200	0.20940600
C	1.56680800	-0.81031400	0.86694500
H	0.42080500	1.57897200	1.27574200
H	0.89026900	2.23937200	-0.29507800
H	0.15910100	-2.42753400	0.74975100
H	1.33079400	-0.65031300	1.93020800
H	2.43996200	-1.45866100	0.81222800
H	-0.26091100	0.47646100	-1.47452900
N	-0.78451800	-0.63091100	0.25525200
N	1.85172900	0.44034600	0.17464700
C	3.16243700	1.04418900	0.52386800
C	3.36863000	2.34153400	-0.27933300
H	4.40661100	2.65904100	-0.18491700
H	3.15368100	2.18056500	-1.33581700
H	2.73944200	3.15133500	0.08986900
C	3.26142700	1.38320600	2.02051300
H	3.24325600	0.49139700	2.64615700

H	4.17724600	1.93193000	2.23966300
H	2.42433500	2.01930600	2.30864200
C	4.25231900	0.06430200	0.07108100
C	4.17011100	-0.50407100	-1.20308400
C	5.35708800	-0.24955000	0.85906000
C	5.15704400	-1.35880000	-1.67253300
H	3.30841600	-0.28331100	-1.81740800
C	6.35112700	-1.10666900	0.39182600
H	5.45620200	0.16748400	1.85073700
C	6.25625700	-1.66555900	-0.87493700
H	5.06816100	-1.78924400	-2.66218800
H	7.19797400	-1.33757300	1.02576100
H	7.02634000	-2.33413100	-1.23771000
C	-1.93839100	-1.26449100	-0.30624000
O	-2.04619200	-1.57507000	-1.46979500
O	-2.85429700	-1.44210500	0.63578400
C	-4.21528600	-1.93408300	0.31978200
C	-4.88662800	-0.96913800	-0.65069300
H	-4.42149200	-1.00170300	-1.63234000
H	-5.93735700	-1.24331000	-0.75389000
H	-4.83232500	0.05058200	-0.26782900
C	-4.90008400	-1.89478100	1.67883800
H	-4.38176200	-2.53994300	2.38882000
H	-4.90884000	-0.87805700	2.07226200
H	-5.92963800	-2.24009100	1.58326000
C	-4.14541500	-3.35872000	-0.21403800
H	-5.15799800	-3.75260900	-0.31237200
H	-3.66139900	-3.39507700	-1.18637400
H	-3.59838000	-3.99749400	0.48097500
H	0.65409000	-1.77022800	-0.82473900
C	-1.73397500	1.54522300	-0.36569900
C	-2.36835000	1.81679700	0.84723400
C	-2.24729300	2.10522700	-1.53260200
C	-3.49000000	2.63410300	0.88984400
H	-1.99327300	1.36350600	1.75440000
C	-3.36984000	2.92540500	-1.49405900
H	-1.77057200	1.88625200	-2.48015700
C	-3.99469800	3.19123700	-0.28149600
H	-3.97617300	2.83139500	1.83678000
H	-3.76002400	3.34802600	-2.41101100
H	-4.87277900	3.82332000	-0.24878600

Frequencies

Mode	IR frequency	IR intensity	Raman intensity
1	-34.02480000	1.93850000	0.00000000
2	26.55080000	0.01600000	0.00000000
3	30.26190000	0.20030000	0.00000000
4	32.97930000	0.02940000	0.00000000
5	39.47020000	0.06110000	0.00000000
6	51.35570000	0.19680000	0.00000000
7	58.12650000	0.66250000	0.00000000
8	78.42290000	1.08120000	0.00000000
9	101.09020000	0.48860000	0.00000000

10	109.52420000	0.86210000	0.00000000
11	127.88390000	1.38930000	0.00000000
12	144.97130000	3.49850000	0.00000000
13	176.37720000	2.62100000	0.00000000
14	187.97980000	1.41970000	0.00000000
15	205.58500000	0.18530000	0.00000000
16	209.25510000	0.07750000	0.00000000
17	218.35920000	3.76340000	0.00000000
18	243.32240000	0.34540000	0.00000000
19	253.91430000	0.68140000	0.00000000
20	260.62720000	1.06960000	0.00000000
21	272.53020000	1.59530000	0.00000000
22	272.81440000	5.84930000	0.00000000
23	297.51120000	1.80150000	0.00000000
24	309.43440000	2.15860000	0.00000000
25	313.53100000	0.38590000	0.00000000
26	324.01790000	8.74060000	0.00000000
27	327.62920000	0.27080000	0.00000000
28	337.93530000	0.14770000	0.00000000
29	356.58980000	0.48990000	0.00000000
30	358.83750000	1.69770000	0.00000000
31	382.46040000	1.19490000	0.00000000
32	415.49870000	0.44670000	0.00000000
33	415.67590000	1.82940000	0.00000000
34	417.62880000	0.30970000	0.00000000
35	423.16220000	3.81640000	0.00000000
36	453.81250000	2.31770000	0.00000000
37	474.89320000	5.68300000	0.00000000
38	483.61080000	2.87340000	0.00000000
39	512.24860000	2.12780000	0.00000000
40	526.80150000	5.07740000	0.00000000
41	530.60400000	13.13860000	0.00000000
42	552.02180000	11.54390000	0.00000000
43	582.00970000	22.89440000	0.00000000
44	612.04090000	12.11970000	0.00000000
45	636.89920000	0.60660000	0.00000000
46	638.02010000	0.47170000	0.00000000
47	647.74570000	2.10400000	0.00000000
48	714.54610000	66.67430000	0.00000000
49	716.66480000	76.62280000	0.00000000
50	733.12890000	2.08150000	0.00000000
51	749.45190000	30.40730000	0.00000000
52	759.96510000	7.71860000	0.00000000

53	778.95360000	9.52560000	0.00000000
54	786.69400000	51.38060000	0.00000000
55	807.14760000	10.63380000	0.00000000
56	811.71600000	8.20320000	0.00000000
57	841.24800000	16.18510000	0.00000000
58	854.65740000	40.95660000	0.00000000
59	860.84270000	1.30020000	0.00000000
60	861.59800000	0.45760000	0.00000000
61	875.49410000	13.18600000	0.00000000
62	911.07320000	8.39570000	0.00000000
63	932.80560000	1.06780000	0.00000000
64	933.19350000	0.42530000	0.00000000
65	933.85170000	1.76880000	0.00000000
66	936.49590000	10.08890000	0.00000000
67	941.23560000	15.34710000	0.00000000
68	947.82070000	12.27170000	0.00000000
69	974.46790000	0.09370000	0.00000000
70	982.75420000	0.22380000	0.00000000
71	983.15920000	0.08920000	0.00000000
72	989.48460000	36.00490000	0.00000000
73	998.56140000	0.41220000	0.00000000
74	1002.53630000	0.50610000	0.00000000
75	1014.65020000	87.93010000	0.00000000
76	1021.38120000	3.11550000	0.00000000
77	1025.91400000	0.43700000	0.00000000
78	1026.08110000	1.92000000	0.00000000
79	1050.30970000	6.73560000	0.00000000
80	1053.00120000	25.11460000	0.00000000
81	1053.75760000	1.91860000	0.00000000
82	1054.95420000	2.40650000	0.00000000
83	1066.53740000	9.18910000	0.00000000
84	1091.81820000	7.66800000	0.00000000
85	1099.97530000	17.70370000	0.00000000
86	1104.44000000	14.28190000	0.00000000
87	1122.94440000	13.56050000	0.00000000
88	1128.76240000	64.58560000	0.00000000
89	1133.40050000	41.72580000	0.00000000
90	1146.67540000	37.60910000	0.00000000
91	1161.56620000	4.64060000	0.00000000
92	1179.54190000	307.15110000	0.00000000
93	1179.72830000	135.32130000	0.00000000
94	1180.28070000	189.33710000	0.00000000
95	1198.82990000	3.34540000	0.00000000

96	1203.12210000	86.16310000	0.00000000
97	1206.12100000	18.38400000	0.00000000
98	1224.89430000	8.41830000	0.00000000
99	1229.91270000	21.49870000	0.00000000
100	1248.95850000	3.95900000	0.00000000
101	1269.18710000	371.46080000	0.00000000
102	1272.34420000	30.66370000	0.00000000
103	1278.63960000	119.16650000	0.00000000
104	1284.72680000	34.06240000	0.00000000
105	1286.11300000	50.34360000	0.00000000
106	1322.79770000	45.95800000	0.00000000
107	1329.96940000	2.64420000	0.00000000
108	1332.72440000	63.00810000	0.00000000
109	1349.52200000	1.42020000	0.00000000
110	1358.20900000	4.93970000	0.00000000
111	1359.70700000	15.59310000	0.00000000
112	1380.77170000	24.77440000	0.00000000
113	1392.86200000	11.84880000	0.00000000
114	1396.47720000	24.46170000	0.00000000
115	1399.55380000	24.51610000	0.00000000
116	1402.53350000	21.99970000	0.00000000
117	1410.27080000	11.75450000	0.00000000
118	1413.67840000	5.33270000	0.00000000
119	1425.91690000	20.62930000	0.00000000
120	1426.37160000	5.02970000	0.00000000
121	1468.30910000	0.38130000	0.00000000
122	1479.62380000	13.70930000	0.00000000
123	1484.06510000	2.14190000	0.00000000
124	1484.35460000	0.16300000	0.00000000
125	1485.30880000	1.98020000	0.00000000
126	1488.32540000	1.86920000	0.00000000
127	1490.00200000	5.04380000	0.00000000
128	1490.80170000	27.39970000	0.00000000
129	1492.68900000	0.10530000	0.00000000
130	1496.01300000	4.33010000	0.00000000
131	1497.99030000	2.66090000	0.00000000
132	1501.16210000	0.60620000	0.00000000
133	1507.13430000	17.10480000	0.00000000
134	1510.41360000	4.10490000	0.00000000
135	1517.42420000	23.08410000	0.00000000
136	1531.26250000	23.43550000	0.00000000
137	1532.56190000	12.08400000	0.00000000
138	1624.15700000	0.73380000	0.00000000

139	1627.82640000	1.28650000	0.00000000
140	1644.42130000	9.91480000	0.00000000
141	1648.00080000	3.03790000	0.00000000
142	1737.04140000	366.83860000	0.00000000
143	2940.96330000	49.50050000	0.00000000
144	2945.74930000	110.07400000	0.00000000
145	2979.99660000	38.11830000	0.00000000
146	2982.95610000	125.94270000	0.00000000
147	3043.05080000	15.25030000	0.00000000
148	3043.11020000	27.31130000	0.00000000
149	3045.83450000	29.18160000	0.00000000
150	3049.31260000	26.08110000	0.00000000
151	3052.24830000	22.53070000	0.00000000
152	3076.06320000	40.39440000	0.00000000
153	3098.97520000	29.73860000	0.00000000
154	3104.42330000	13.90900000	0.00000000
155	3106.54790000	3.47260000	0.00000000
156	3109.50500000	8.08770000	0.00000000
157	3110.53450000	79.26060000	0.00000000
158	3113.92900000	42.11870000	0.00000000
159	3115.85330000	34.70360000	0.00000000
160	3117.30240000	37.19500000	0.00000000
161	3119.40520000	53.68080000	0.00000000
162	3125.67110000	32.39430000	0.00000000
163	3143.21790000	0.19810000	0.00000000
164	3146.15720000	26.46330000	0.00000000
165	3166.75770000	0.84660000	0.00000000
166	3166.77470000	4.15760000	0.00000000
167	3172.71470000	1.76220000	0.00000000
168	3175.01130000	17.70120000	0.00000000
169	3182.05390000	29.88040000	0.00000000
170	3189.17590000	35.75830000	0.00000000
171	3192.46000000	31.99070000	0.00000000
172	3199.10100000	7.93540000	0.00000000
173	3199.30790000	15.09670000	0.00000000
174	3205.91800000	12.27370000	0.00000000

Equatorial Anticlockwise Transition State

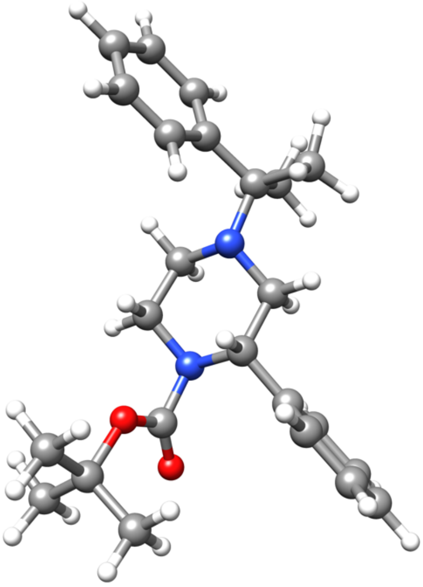

Route : # opt freq b3lyp scrf=(solvent=thf) geom=connectivity def2tzvp
empiricaldispersion=gd3bj int=ultrafine pop=(regular,mk)
SMILES : CC(C)(C)OC(=O)N2CCN(C(C)(C)c1ccccc1)CC2c3ccccc3
Formula : $C_{24}H_{32}N_2O_2$
Charge : 0
Multiplicity : 1
Dipole : 2.5981 Debye
Energy : -1194.36566296 a.u.
Gibbs Energy : -1193.903995 a.u.
Number of imaginary frequencies : 1

Cartesian Coordinates (XYZ format)

60

C	0.55574100	-1.52764500	-0.04376200
C	-0.74415200	-0.74140600	0.15097900
C	0.24702100	1.09522600	-1.08145500
C	1.50590700	0.27359500	-1.27761400
H	0.44303200	-2.16843200	-0.93183500
H	0.67022800	-2.17591200	0.82090400
H	0.09810000	1.75558200	-1.93661800
H	1.42916400	-0.27131500	-2.23081300
H	2.35986700	0.94609000	-1.34446400
H	-0.63853000	-0.16425000	1.07972100
N	-0.90925700	0.19213000	-0.98689900
N	1.68916600	-0.62485400	-0.14472000
C	3.03866500	-1.23827400	-0.06538400
C	3.12420300	-2.15225100	1.17065200
H	4.16520500	-2.42398800	1.34252800
H	2.75267900	-1.63913100	2.05782700
H	2.55922500	-3.07413500	1.03292600
C	3.36440600	-2.07990700	-1.31053600
H	3.43992800	-1.47027300	-2.21038300

H	4.30378500	-2.61857900	-1.18666300
H	2.58162100	-2.82244800	-1.46634500
C	4.04444300	-0.10073400	0.15328200
C	3.76643900	0.87865400	1.11072900
C	5.25666500	-0.02586100	-0.52839800
C	4.66796100	1.89937100	1.37598700
H	2.82095700	0.84176400	1.63359700
C	6.16565100	0.99662100	-0.26503800
H	5.50762000	-0.76267700	-1.27755700
C	5.87623000	1.96388900	0.68717900
H	4.42746900	2.64833000	2.12022200
H	7.09953200	1.03439500	-0.81163300
H	6.58032900	2.76052400	0.89025900
C	-2.16520900	0.86594800	-0.95982800
O	-3.08934900	0.56337600	-1.67202600
O	-2.17353900	1.84609400	-0.05340100
C	-3.36857000	2.67940900	0.20539400
C	-3.73979000	3.45671100	-1.05082300
H	-4.09253300	2.79552100	-1.83787500
H	-4.53239200	4.16628200	-0.80948700
H	-2.87930700	4.01917400	-1.41607500
C	-2.88539500	3.61850400	1.30155100
H	-2.57544600	3.05336900	2.18104400
H	-2.04034000	4.21286800	0.95279100
H	-3.69025500	4.29499000	1.58959500
C	-4.50314600	1.79553100	0.70796100
H	-5.33534800	2.42697500	1.02198200
H	-4.85191100	1.11756900	-0.06648100
H	-4.17422600	1.20966200	1.56705200
H	0.35327400	1.71196300	-0.18129500
C	-1.92458900	-1.66762300	0.30237100
C	-2.32679200	-2.50496900	-0.73868900
C	-2.62315100	-1.70903400	1.50606200
C	-3.39988700	-3.36948900	-0.57376800
H	-1.80922000	-2.46090000	-1.68710200
C	-3.69962100	-2.57385500	1.67558400
H	-2.32376800	-1.05494000	2.31599400
C	-4.08948100	-3.40775500	0.63516600
H	-3.70406400	-4.01104400	-1.39092500
H	-4.23381100	-2.59167900	2.61688000
H	-4.92840900	-4.07999300	0.76136500

Frequencies

Mode	IR frequency	IR intensity	Raman intensity
1	-36.76800000	1.71490000	0.00000000
2	24.67200000	0.15820000	0.00000000
3	27.96440000	0.47510000	0.00000000
4	32.01220000	0.00380000	0.00000000
5	39.12030000	0.12640000	0.00000000
6	44.14930000	0.08990000	0.00000000
7	57.60790000	0.92670000	0.00000000
8	82.40870000	0.56310000	0.00000000
9	93.99930000	0.98850000	0.00000000

10	111.27340000	0.57180000	0.00000000
11	119.81530000	1.18940000	0.00000000
12	147.45940000	4.16500000	0.00000000
13	175.86480000	1.38700000	0.00000000
14	187.21040000	3.69430000	0.00000000
15	202.73550000	0.26660000	0.00000000
16	208.70280000	0.08560000	0.00000000
17	226.45890000	0.36480000	0.00000000
18	243.74040000	0.41820000	0.00000000
19	249.57130000	0.16250000	0.00000000
20	262.66820000	2.69010000	0.00000000
21	271.00980000	0.32220000	0.00000000
22	282.82400000	2.00010000	0.00000000
23	292.12820000	0.00400000	0.00000000
24	304.86180000	0.34350000	0.00000000
25	310.44030000	0.35840000	0.00000000
26	324.17250000	7.27230000	0.00000000
27	326.57550000	0.22190000	0.00000000
28	343.50330000	1.40420000	0.00000000
29	354.05240000	0.31960000	0.00000000
30	373.32490000	2.11300000	0.00000000
31	381.33060000	4.44800000	0.00000000
32	415.18180000	1.09740000	0.00000000
33	417.45150000	0.19740000	0.00000000
34	417.60660000	0.08510000	0.00000000
35	421.03190000	0.83160000	0.00000000
36	456.62980000	6.60510000	0.00000000
37	472.86480000	4.90120000	0.00000000
38	486.23160000	0.81410000	0.00000000
39	507.90740000	3.74950000	0.00000000
40	521.25780000	5.48100000	0.00000000
41	545.93300000	3.10360000	0.00000000
42	559.52320000	31.90730000	0.00000000
43	583.51560000	15.46080000	0.00000000
44	615.89510000	14.31610000	0.00000000
45	636.66200000	0.13860000	0.00000000
46	637.90850000	0.96510000	0.00000000
47	644.27490000	5.25790000	0.00000000
48	684.87550000	4.75890000	0.00000000
49	713.65510000	63.91590000	0.00000000
50	716.89640000	76.30030000	0.00000000
51	735.65300000	4.85710000	0.00000000
52	774.60370000	25.97330000	0.00000000

53	784.83610000	17.96370000	0.00000000
54	786.95880000	33.09440000	0.00000000
55	805.39400000	6.74490000	0.00000000
56	811.71170000	12.76780000	0.00000000
57	840.76380000	3.91160000	0.00000000
58	855.68410000	53.80120000	0.00000000
59	858.55150000	0.66720000	0.00000000
60	861.89900000	0.31060000	0.00000000
61	880.03810000	8.70810000	0.00000000
62	910.75470000	6.26000000	0.00000000
63	932.53810000	1.69030000	0.00000000
64	933.03830000	2.65360000	0.00000000
65	933.11110000	0.26590000	0.00000000
66	934.34750000	7.35210000	0.00000000
67	941.56180000	13.95970000	0.00000000
68	950.01500000	18.34900000	0.00000000
69	974.24630000	0.08280000	0.00000000
70	979.15810000	0.16560000	0.00000000
71	983.02680000	0.18330000	0.00000000
72	989.38960000	27.92600000	0.00000000
73	995.54450000	0.11750000	0.00000000
74	998.06270000	1.42660000	0.00000000
75	1015.61300000	76.43530000	0.00000000
76	1021.17820000	3.22380000	0.00000000
77	1025.60490000	0.32160000	0.00000000
78	1025.89910000	2.34370000	0.00000000
79	1049.12460000	9.46740000	0.00000000
80	1053.24290000	26.45070000	0.00000000
81	1053.85520000	3.59210000	0.00000000
82	1054.25290000	5.41590000	0.00000000
83	1066.15600000	9.36280000	0.00000000
84	1091.25400000	39.99660000	0.00000000
85	1100.71810000	28.42800000	0.00000000
86	1105.39520000	7.05500000	0.00000000
87	1118.41050000	102.37080000	0.00000000
88	1121.76080000	20.26880000	0.00000000
89	1133.07940000	57.04820000	0.00000000
90	1139.89270000	171.00220000	0.00000000
91	1161.44820000	23.03590000	0.00000000
92	1169.06460000	545.99740000	0.00000000
93	1180.06710000	0.55760000	0.00000000
94	1180.58260000	0.22740000	0.00000000
95	1201.21410000	1.07180000	0.00000000

96	1203.20150000	113.55870000	0.00000000
97	1206.17880000	10.01460000	0.00000000
98	1224.51690000	6.03610000	0.00000000
99	1231.27710000	8.26200000	0.00000000
100	1245.54420000	135.58140000	0.00000000
101	1255.82050000	119.00620000	0.00000000
102	1272.18120000	23.91900000	0.00000000
103	1278.29970000	50.86580000	0.00000000
104	1284.12920000	46.25320000	0.00000000
105	1288.24790000	26.20900000	0.00000000
106	1324.60850000	43.51860000	0.00000000
107	1329.99720000	5.26070000	0.00000000
108	1332.17330000	17.91550000	0.00000000
109	1350.68750000	2.00670000	0.00000000
110	1358.61250000	1.03760000	0.00000000
111	1363.28750000	7.88170000	0.00000000
112	1384.26310000	12.75430000	0.00000000
113	1395.42090000	22.19640000	0.00000000
114	1395.88550000	13.55160000	0.00000000
115	1399.90850000	26.84260000	0.00000000
116	1402.24110000	26.06370000	0.00000000
117	1408.79110000	6.47850000	0.00000000
118	1412.97880000	8.91430000	0.00000000
119	1425.75500000	19.77430000	0.00000000
120	1426.71680000	0.63370000	0.00000000
121	1467.84030000	0.33170000	0.00000000
122	1479.57570000	13.05100000	0.00000000
123	1483.65710000	0.04660000	0.00000000
124	1483.92650000	2.49240000	0.00000000
125	1484.09050000	1.31260000	0.00000000
126	1488.32420000	2.16690000	0.00000000
127	1489.69740000	17.02840000	0.00000000
128	1490.91250000	17.13560000	0.00000000
129	1492.56680000	0.46140000	0.00000000
130	1495.21770000	7.33860000	0.00000000
131	1497.31460000	2.87600000	0.00000000
132	1501.25150000	0.98890000	0.00000000
133	1507.12070000	16.80120000	0.00000000
134	1509.23780000	4.00580000	0.00000000
135	1516.35260000	22.96790000	0.00000000
136	1531.35220000	22.79050000	0.00000000
137	1533.43960000	10.82070000	0.00000000
138	1624.20660000	0.73730000	0.00000000

139	1628.31760000	0.81720000	0.00000000
140	1644.31780000	10.08960000	0.00000000
141	1648.42530000	1.95740000	0.00000000
142	1761.59340000	517.21880000	0.00000000
143	2943.37760000	45.18490000	0.00000000
144	2948.11190000	108.34100000	0.00000000
145	2971.90180000	75.06930000	0.00000000
146	2998.84330000	78.34400000	0.00000000
147	3042.80700000	26.78270000	0.00000000
148	3042.87770000	16.13940000	0.00000000
149	3045.91450000	34.03690000	0.00000000
150	3049.23970000	25.61490000	0.00000000
151	3052.25120000	17.78820000	0.00000000
152	3081.67930000	39.90120000	0.00000000
153	3097.82850000	30.64430000	0.00000000
154	3105.42840000	10.37490000	0.00000000
155	3106.33470000	3.44170000	0.00000000
156	3108.14470000	16.18900000	0.00000000
157	3110.45970000	75.26220000	0.00000000
158	3112.58030000	45.49140000	0.00000000
159	3115.76810000	33.84810000	0.00000000
160	3117.28400000	40.21320000	0.00000000
161	3118.52070000	58.81570000	0.00000000
162	3125.54640000	33.03920000	0.00000000
163	3143.48370000	0.81860000	0.00000000
164	3146.44470000	24.07820000	0.00000000
165	3165.34170000	6.10330000	0.00000000
166	3166.60320000	0.81740000	0.00000000
167	3172.41250000	1.27230000	0.00000000
168	3174.87620000	17.50800000	0.00000000
169	3181.73120000	29.41800000	0.00000000
170	3189.11970000	35.65790000	0.00000000
171	3192.10640000	34.49390000	0.00000000
172	3199.04410000	16.28490000	0.00000000
173	3199.36850000	8.33040000	0.00000000
174	3205.87020000	12.46970000	0.00000000

Thermochemistry Values

Left Rotamer Axial:

	Temperature	
	298 K	195 K
ε_0	–1194.38643726 a.u.	–1194.38643726 a.u.
ε_{ZPE}	0.519464 a.u.	0.519464 a.u.
E_{tot}	0.546344 a.u.	0.531746 a.u.
H_{corr}	0.547288 a.u.	0.532363 a.u.
G_{corr}	0.461281 a.u.	0.487928 a.u.
S_{tot}	181.016 calmol^{-1}K^{-1}	142.994 calmol^{-1}K^{-1}
$\varepsilon_0 + \varepsilon_{ZPE}$	–1193.866973 a.u.	–1193.866973 a.u.
$\varepsilon_0 + E_{tot}$	–1193.840094 a.u.	–1193.854691 a.u.
$\varepsilon_0 + H_{corr}$	–1193.839149 a.u.	–1193.854074 a.u.
$\varepsilon_0 + G_{corr}$	–1193.925156 a.u.	–1193.898509 a.u.

Right Rotamer Axial:

	Temperature	
	298 K	195 K
ε_0	–1194.38576012 a.u.	–1194.38576012 a.u.
ε_{ZPE}	0.519236 a.u.	0.519236 a.u.
E_{tot}	0.546197 a.u.	0.531585 a.u.
H_{corr}	0.547141 a.u.	0.532203 a.u.
G_{corr}	0.460232 a.u.	0.487188 a.u.
S_{tot}	182.916 calmol^{-1}K^{-1}	144.858 calmol^{-1}K^{-1}
$\varepsilon_0 + \varepsilon_{ZPE}$	–1193.866525 a.u.	–1193.866524 a.u.
$\varepsilon_0 + E_{tot}$	–1193.839563 a.u.	–1193.854175 a.u.
$\varepsilon_0 + H_{corr}$	–1193.838619 a.u.	–1193.853557 a.u.
$\varepsilon_0 + G_{corr}$	–1193.925528 a.u.	–1193.898572 a.u.

Left Rotamer Equatorial:

	Temperature	
	298 K	195 K
ε_0	–1194.38233605 a.u.	–1194.38233605 a.u.
ε_{ZPE}	0.518995 a.u.	0.518995 a.u.
E_{tot}	0.545961 a.u.	0.531318 a.u.
H_{corr}	0.546905 a.u.	0.531935 a.u.
G_{corr}	0.461409 a.u.	0.487871 a.u.
S_{tot}	179.943 calmol^{-1}K^{-1}	141.800 calmol^{-1}K^{-1}
$\varepsilon_0 + \varepsilon_{ZPE}$	–1193.863341 a.u.	–1193.863341 a.u.
$\varepsilon_0 + E_{tot}$	–1193.836375 a.u.	–1193.851018 a.u.
$\varepsilon_0 + H_{corr}$	–1193.835431 a.u.	–1193.850401 a.u.
$\varepsilon_0 + G_{corr}$	–1193.920928 a.u.	–1193.894465 a.u.

Right Rotamer Equatorial:

	Temperature	
	298 K	195 K
ε_0	–1194.37986331 a.u.	–1194.37986331 a.u.
ε_{ZPE}	0.518668 a.u.	0.518668 a.u.
E_{tot}	0.545801 a.u.	0.531135 a.u.
H_{corr}	0.546745 a.u.	0.531752 a.u.
G_{corr}	0.45989 a.u.	0.486818 a.u.
S_{tot}	182.801 calmol^{-1}K^{-1}	144.598 calmol^{-1}K^{-1}
$\varepsilon_0 + \varepsilon_{ZPE}$	–1193.861195 a.u.	–1193.861195 a.u.
$\varepsilon_0 + E_{tot}$	–1193.834063 a.u.	–1193.848728 a.u.
$\varepsilon_0 + H_{corr}$	–1193.833118 a.u.	–1193.848111 a.u.
$\varepsilon_0 + G_{corr}$	–1193.919973 a.u.	–1193.893045 a.u.

Axial Clockwise Transition State:

	Temperature	
	298 K	195 K
ε_0	–1194.35793774 a.u.	–1194.35793774 a.u.
ε_{ZPE}	0.518653 a.u.	0.518653 a.u.
E_{tot}	0.544687 a.u.	0.530395 a.u.
H_{corr}	0.545632 a.u.	0.531012 a.u.
G_{corr}	0.462020 a.u.	0.487897 a.u.
S_{tot}	175.976 calmol^{-1}K^{-1}	138.745 calmol^{-1}K^{-1}
$\varepsilon_0 + \varepsilon_{ZPE}$	–1193.839285 a.u.	–1193.839285 a.u.
$\varepsilon_0 + E_{tot}$	–1193.813250 a.u.	–1193.827543 a.u.
$\varepsilon_0 + H_{corr}$	–1193.812306 a.u.	–1193.826926 a.u.
$\varepsilon_0 + G_{corr}$	–1193.895918 a.u.	–1193.870041 a.u.

Axial Anticlockwise Transition State:

	Temperature	
	298 K	195 K
ε_0	–1194.35848593 a.u.	–1194.35848593 a.u.
ε_{ZPE}	0.518536 a.u.	0.518536 a.u.
E_{tot}	0.544693 a.u.	0.530371 a.u.
H_{corr}	0.545637 a.u.	0.530989 a.u.
G_{corr}	0.462165 a.u.	0.487990 a.u.
S_{tot}	175.682 calmol^{-1}K^{-1}	138.371 calmol^{-1}K^{-1}
$\varepsilon_0 + \varepsilon_{ZPE}$	–1193.839950 a.u.	–1193.839950 a.u.
$\varepsilon_0 + E_{tot}$	–1193.813793 a.u.	–1193.828115 a.u.
$\varepsilon_0 + H_{corr}$	–1193.812849 a.u.	–1193.827497 a.u.
$\varepsilon_0 + G_{corr}$	–1193.896321 a.u.	–1193.870496 a.u.

Equatorial Clockwise Transition State:

	Temperature	
	298 K	195 K
ε_0	–1194.36728384 a.u.	–1194.36728384 a.u.
ε_{ZPE}	0.518307 a.u.	0.518307 a.u.
E_{tot}	0.544481 a.u.	0.530153 a.u.
H_{corr}	0.545426 a.u.	0.530771 a.u.
G_{corr}	0.462121 a.u.	0.487887 a.u.
S_{tot}	175.330 calmol^{-1}K^{-1}	138.002 calmol^{-1}K^{-1}
$\varepsilon_0 + \varepsilon_{ZPE}$	–1193.848977 a.u.	–1193.848977 a.u.
$\varepsilon_0 + E_{tot}$	–1193.822802 a.u.	–1193.837131 a.u.
$\varepsilon_0 + H_{corr}$	–1193.821858 a.u.	–1193.836513 a.u.
$\varepsilon_0 + G_{corr}$	–1193.905163 a.u.	–1193.879397 a.u.

Equatorial Anticlockwise Transition State:

	Temperature	
	298 K	195 K
ε_0	–1194.36566296 a.u.	–1194.36566296 a.u.
ε_{ZPE}	0.518207 a.u.	0.518207 a.u.
E_{tot}	0.544419 a.u.	0.530084 a.u.
H_{corr}	0.545363 a.u.	0.530702 a.u.
G_{corr}	0.461668 a.u.	0.487567 a.u.
S_{tot}	176.151 calmol^{-1}K^{-1}	138.806 calmol^{-1}K^{-1}
$\varepsilon_0 + \varepsilon_{ZPE}$	–1193.847456 a.u.	–1193.847456 a.u.
$\varepsilon_0 + E_{tot}$	–1193.821244 a.u.	–1193.835579 a.u.
$\varepsilon_0 + H_{corr}$	–1193.820300 a.u.	–1193.834961 a.u.
$\varepsilon_0 + G_{corr}$	–1193.903995 a.u.	–1193.878096 a.u.

7. References

1. Chak, B. C. M.; McAuley, A. *Can. J. Chem.* **2006**, *84*, 187–195.

2. Salituro, F. G.; Saunders, J. O.; Yan, S.; *WO 2012/092442 Al*

3. Firth, J. D.; O'Brien, P.; Ferris, L. *J. Org. Chem.* **2017**, *82*, 7023–7031.

4. O'Brien, C. J.; Nicewicz, D. A. *Synlett* **2021**, *32*, 814–816.

5. Shaw, M. H.; Shurtleff, V. W.; Terrett, J. A.; Cuthbertson, J. D.; MacMillan, D. W. C. *Science* **2016**, *352*, 1304–1308.

6. Zuo, Z.; Ahneman, D. T.; Chu, L.; Terrett, J. A.; Doyle, A. G.; MacMillan, D. W. C. *Science* **2014**, *345*, 437–440.

7. Yao, H.; Uras, G.; Zhang, P.; Shengtao, X.; Yin, Y.; Liu, J.; Qin, S.; Li, X.; Allen, S.; Bai, R.; Gong, Q.; Zhang, H.; Zhu, Z; Xu, J. *J. Med. Chem.* **2021**, *64*, 7483–7506.

8. For software, see iNMR (http://www.inmr.net).

9. Becke, A. D. *J. Chem. Phys.* **1993**, *98*, 5648–5652.

10. Frisch, M. J.; Trucks, G. W.; Schlegel, H. B.; Scuseria, G. E.; Robb, M. A.; Cheeseman, J. R.; Scalmani, G.; Barone, V.; Mennucci, B.; Petersson, G. A.; Nakatsuji, H.; Caricato, M.; Li, X.; Hratchian, H. P.; Izmaylov, A. F.; Bloino, J.; Zheng, G.; Sonnenberg, J. L.; Hada, M.; Ehara, M.; Toyota, K.; Fukuda, R.; Hasegawa, J.; Ishida, M.; Nakajima, T.; Honda, Y.; Kitao, O.; Nakai, H.; Vreven, T.; Montgomery, Jr., J. A.; Peralta, J. E.; Ogliaro, F.; Bearpark, M.; Heyd, J. J.; Brothers, E.; Kudin, K. N.; Staroverov, V. N.; Kobayashi, R.; Normand, J.; Raghavachari, K.; Rendell, A.; Burant, J. C.; Iyengar, S. S.; Tomasi, J.; Cossi, M.; Rega, N.; Millam, N. J.; Klene, M.; Knox, J. E.; Cross, J. B.; Bakken, V.; Adamo, C.; Jaramillo, J.; Gomperts, R.; Stratmann, R. E.; Yazyev, O.; Austin, A. J.; Cammi, R.; Pomelli, C.; Ochterski, J. W.; Martin, R. L.; Morokuma, K.; Zakrzewski, V. G.; Voth, G. A.; Salvador, P.; Dannenberg, J. J.; Dapprich, S.; Daniels, A. D.; Farkas, Ö.; Foresman, J. B.; Ortiz, J. V.; Cioslowski, J.; Fox, D. J. Gaussian, Inc., Wallingford CT, **2009**.

11. Grimme, S.; Ehrlich, S.; Goerigk, L. *J. Comput. Chem*. **2011**, *32*, 1456–1465.

12. Weigend, F.; Ahlrichs R. *Phys. Chem. Chem. Phys.* **2005**, *7*, 3297–3305.

13. Scalmani, G.; Frisch, M. J. *J. Chem. Phys.* **2010**, *132*, 114110.

14. Cossi, M.; Rega, N.; Scalmani, G.; Barone, V. *J. Comput. Chem.* **2003**, *24*, 669–681.
